# Supplementary material for: Non-invasive early detection of cancer four years before conventional diagnosis using a blood test
Source: Nat Commun. 2020 Jul 21;11:3475. doi: 10.1038/s41467-020-17316-z (PMC7374162; doi:10.1038/s41467-020-17316-z)
Supplement: Supplementary file 3 — Description of Additional Supplementary Files [file 41467_2020_17316_MOESM3_ESM.docx]

Description of Additional Supplementary Files

**Title:** Supplementary Data 1.

**Description:** (Microsoft Excel Format) Genomic regions and CpG sites targeted by the PanSeer assay (in the hg19 reference genome). Genes were associated with each target region using GREAT with default parameters.

**Title:** Supplementary Data 2.

**Description:** (Microsoft Excel Format) Clinical characteristics and Logistic Regression analysis scores/calls for all cancer patients and healthy controls assayed in this study.

**Title:** Supplementary Data 3.

**Description:** (Microsoft Excel Format) Logistic regression model for cancer detection (including coefficients, intercepts, and cutoffs).

**Title:** Supplementary Data 4.

**Description:** (Microsoft Excel Format) Cross-validation results for cancer detection model (including score and call for 10 sets).

**Title:** Supplementary Data 5.

**Description:** (TSV Format) Data matrix of average methylation levels across each target region and sample (in the hg19 reference genome). Supplementary Data 6. (TSV Format) Data matrix of methylation levels across each individual CpG site in limit of detection samples (in the hg19 reference genome).

**Title:** Supplementary Data 6.

**Description:** (TSV Format) Data matrix of methylation levels across each individual CpG site in limit of detection samples (in the hg19 reference genome).
