# Supplementary material for: Non-invasive early detection of cancer four years before conventional diagnosis using a blood test
Source: Nat Commun. 2020 Jul 21;11:3475. doi: 10.1038/s41467-020-17316-z (PMC7374162; doi:10.1038/s41467-020-17316-z)
Supplement: Supplementary file 10 — Reporting Summary [file 41467_2020_17316_MOESM10_ESM.pdf]

nature research

Corresponding author(s): Kun Zhang

Last updated by author(s): Jun 16, 2020

Reporting Summary

Nature Research wishes to improve the reproducibility of the work that we publish. This form provides structure for consistency and transparency in reporting. For further information on Nature Research policies, seeAuthors & Referees and theEditorial Policy Checklist .

Statistics

For all statistical analyses, confirm that the following items are present in the figure legend, table legend, main text, or Methods section.

n/a	Confirmed	
☐	☒	The exact sample size (n) for each experimental group/condition, given as a discrete number and unit of measurement
☐	☒	A statement on whether measurements were taken from distinct samples or whether the same sample was measured repeatedly
☐	☒	The statistical test(s) used AND whether they are one- or two-sided<br>*Only common tests should be described solely by name; describe more complex techniques in the Methods section.*
☐	☒	A description of all covariates tested
☐	☒	A description of any assumptions or corrections, such as tests of normality and adjustment for multiple comparisons
☐	☒	A full description of the statistical parameters including central tendency (e.g. means) or other basic estimates (e.g. regression coefficient) AND variation (e.g. standard deviation) or associated estimates of uncertainty (e.g. confidence intervals)
☐	☒	For null hypothesis testing, the test statistic (e.g. F, t, r) with confidence intervals, effect sizes, degrees of freedom and P value noted<br>*Give P values as exact values whenever suitable.*
☒	☐	For Bayesian analysis, information on the choice of priors and Markov chain Monte Carlo settings
☒	☐	For hierarchical and complex designs, identification of the appropriate level for tests and full reporting of outcomes
☒	☐	Estimates of effect sizes (e.g. Cohen's d, Pearson's r), indicating how they were calculated

Our web collection on statistics for biologists contains articles on many of the points above.

Software and code

Policy information about availability of computer code

Data collection	The standard Illumina software package for Illumina sequencers was used.
Data analysis	All software used in this study has been described in published literature and are publicly available, including:<br>Illumina bcl2fastq software v2.20.0.422<br>PEAR v0.9.6<br>trim_galore v0.4.0<br>UMI_tools v0.5.5<br>Bismark v0.17.0<br>Bowtie2 v2.3.1

For manuscripts utilizing custom algorithms or software that are central to the research but not yet described in published literature, software must be made available to editors/reviewers. We strongly encourage code deposition in a community repository (e.g. GitHub). See the Nature Research guidelines for submitting code & software for further information.

Data

Policy information about availability of data

All manuscripts must include a data availability statement. This statement should provide the following information, where applicable:

- Accession codes, unique identifiers, or web links for publicly available datasets
- A list of figures that have associated raw data
- A description of any restrictions on data availability

The full methylation data matrix for all samples is available publicly at GitHub (https://github.com/ncomms-20-10056-t/ncomms-20-10056-t). Fully open release of genetic sequencing data was not included in the informed consent, hence the raw sequencing data cannot be made publicly available.

Field-specific reporting

Please select the one below that is the best fit for your research. If you are not sure, read the appropriate sections before making your selection.

- [x] Life sciences
- [ ] Behavioural & social sciences
- [ ] Ecological, evolutionary & environmental sciences

For a reference copy of the document with all sections, see nature.com/documents/nr-reporting-summary-flat.pdf

Life sciences study design

All studies must disclose on these points even when the disclosure is negative.

Sample size

Group sizes of 144 control individuals and 144 case patients were chosen; this sample size would be sufficient to verify that the PanSeer assay had an expected sensitivity and specificity of 75% with a power of 1-β=90% and a significance level of α=0.05. Additional case and control patients were incorporated into the study due to their availability.

Data exclusions

Exclusion criteria were incomplete clinical information, plasma volume less than 1 mL, or presence of hemolysis in plasma. For all samples, after processing and sequencing, it was required that at least 200,000 unique mapped DNA molecules were observed in the sequencing data, as lower amounts indicate a low-quality sample.

Out of these 575 pre-diagnosis samples, 191 samples were selected that passed the study's inclusion/exclusion criteria, had confirmed cancer diagnosis by retrospective chart review of hospitalization records and biopsy pathology, and passed experimental quality control metrics.

Replication

Each dilution of the LOD study was performed 6 times to measure reproducibility. All replicates were successfully performed.

Due to the longitudinal nature of the main study and limited plasma available per patient, it was not possible to perform the assay more than once for each sample.

Randomization

Healthy samples passing the study's inclusion/exclusion criteria that matched by time of collection, sex, 10-year age group, and unique mapped DNA molecule count to the pre-diagnosis samples were then randomly selected from the 110,501 TLS healthy samples not diagnosed with cancer for at least five years. We ensured to control for the covariates listed above in addition to location of sample collection, smoking status, cardiovascular disease status, diabetes status, and hypertension status.

Blinding

Following analysis of the training set, the model was locked down and the test set samples were blinded in the analysis.

Reporting for specific materials, systems and methods

We require information from authors about some types of materials, experimental systems and methods used in many studies. Here, indicate whether each material, system or method listed is relevant to your study. If you are not sure if a list item applies to your research, read the appropriate section before selecting a response.

Materials & experimental systems

n/a	Involved in the study	
x		Antibodies
	x	Eukaryotic cell lines
x		Palaeontology
x		Animals and other organisms
	x	Human research participants
x		Clinical data

Methods

n/a	Involved in the study	
x		ChIP-seq
x		Flow cytometry
x		MRI-based neuroimaging

Eukaryotic cell lines

Policy information about cell lines

Cell line source(s)	DNA from HT-29 from ATCC
Authentication	Validated by ATCC
Mycoplasma contamination	DNA purchased from ATCC, not tested for mycoplasma by authors
Commonly misidentified lines (See ICLAC register)	*Name any commonly misidentified cell lines used in the study and provide a rationale for their use.*

Human research participants

Policy information about studies involving human research participants

Population characteristics	The Taizhou Longitudinal Study (TLS) started in July 2007, with a goal to recruit 200,000 people in the city of Taizhou, Jiangsu province, China, and follow the participants for at least 40 years. Taizhou sits at the center of China, downstream of the Yangtze River. While the regional population and economy are average for China, the incidence of digestive cancers in Taizhou is high, at the levels of 36.9, 29.8 and 30.0 per 100,000 person-years for esophageal, gastric, and liver cancer, respectively. According to the surveillance of cancer deaths in 2010, the cancer mortality was 154.05/100,000 person-years, nearly twice the mean incidence rate for China29.<br><br>Healthy samples that matched by time of collection, sex, 10-year age group, and unique mapped DNA molecule count to the pre-diagnosis samples were then randomly selected from the 110,501 TLS healthy samples not diagnosed with cancer for at least five years. We ensured to control for the covariates listed above in addition to location of sample collection, smoking status, cardiovascular disease status, diabetes status, and hypertension status.
Recruitment	The baseline survey of the Taizhou Longitudinal Study took place during 2007-2016 in the Taixing, Gaogang, and Hailing areas in Taizhou. All men and women aged 30-75 who were living in these districts were eligible. Potentially eligible citizens were delivered an invitation letter by local community leaders and health workers, with support from the government for extensive publicity campaigns and health promotions. A Regional Coordinating Centre (RCC) and survey teams were set up for the baseline survey, consisting of 40 full-time staff members with medical qualifications and fieldwork experience in the three studied districts. All participants were indefinitely monitored for cancer occurrence through linkages with local cancer registries and health insurance databases. Exposure data were collected through survey questionnaires and physical measurements. In addition, blood samples and other biological samples were collected. A total of 123,115 individuals have been recruited and the average follow-up time is 8.1 years.<br><br>Taizhou sits at the center of China, downstream of the Yangtze River. While the regional population and economy are average for China, the incidence of digestive cancers in Taizhou is high, at the levels of 36.9, 29.8 and 30.0 per 100,000 person-years for esophageal, gastric, and liver cancer, respectively (Ref. 61). According to the surveillance of cancer deaths in 2010, the cancer mortality was 154.05/100,000 person-years, nearly twice the mean incidence rate for China (Ref.62).
Ethics oversight	The study was approved by the Human Ethics Committee of Fudan University. Written informed consent was obtained from all study participants prior to inclusion in the TLS

Note that full information on the approval of the study protocol must also be provided in the manuscript.
