# Supplementary material for: Non-invasive early detection of cancer four years before conventional diagnosis using a blood test
Source: Nat Commun. 2020 Jul 21;11:3475. doi: 10.1038/s41467-020-17316-z (PMC7374162; doi:10.1038/s41467-020-17316-z)

Supplementary Materials for

Non-invasive early detection of cancer four years before conventional diagnosis using a blood test

Xingdong Chen, Jeffrey Gole, Athurva Gore, Qiye He, Ming Lu, Jun Min, Ziyu Yuan, Xiaorong Yang, Yanfeng Jiang, Tiejun Zhang, Chen Suo, Xiaojie Li, Lei Cheng, Zhenhua Zhang, Hongyu Niu, Zhe Li, Zhen Xie, Han Shi, Xiang Zhang, Min Fan, Xiaofeng Wang, Yajun Yang, Justin Dang, Catie McConnell, Juan Zhang, Jiucun Wang, Shunzhang Yu, Weimin Ye, Yuan Gao, Kun Zhang, Rui Liu, Li Jin

Correspondence to: rliu@singleragenomics.com

The following sections include:

Supplementary Figs. 1 to 23
Supplementary Table 1
Supplementary Note 1-6

The following files are available at Nature server as well as GitHub: https://github.com/ncomms-20-10056-t/ncomms-20-10056-t

Supplementary Data 1 [Genomic regions and CpG sites targeted by PanSeer]
Supplementary Data 2 [Clinical characteristics and model scores/calls]
Supplementary Data 3 [Model coefficients, intercepts, and cutoffs for cancer detection]
Supplementary Data 4 [Cross-validation results for cancer detection model]
Supplementary Data 5 [Data matrix of average methylation values]
Supplementary Data 6 [Data matrix for limit of detection experiment]

A

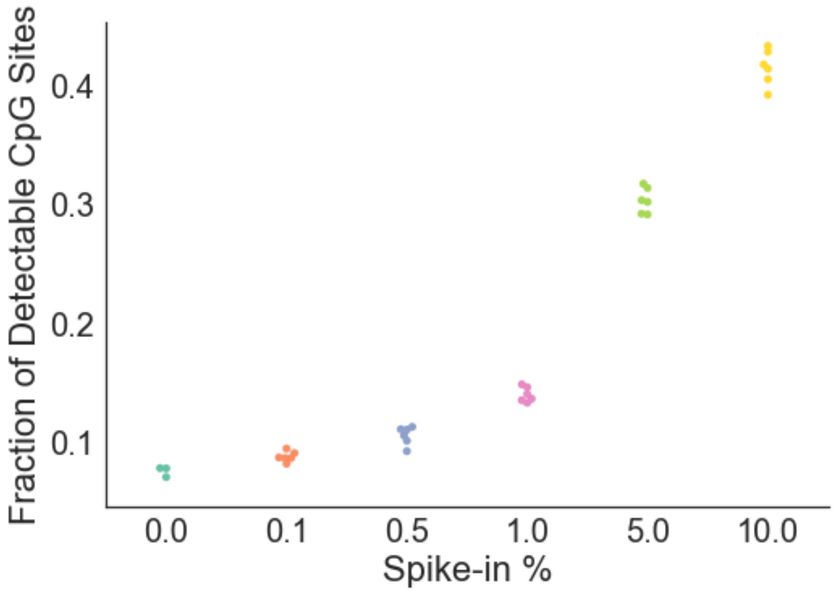

B

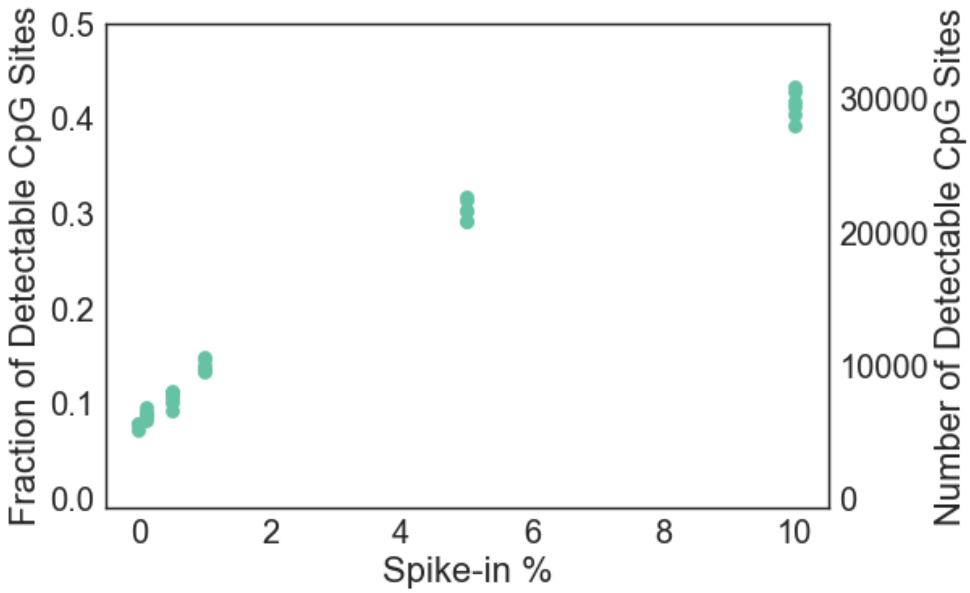

Supplementary Fig. 1.

Scatter plots showing limit of detection of PanSeer assay. Fragmented DNA from the cancer cell line HT29 was spiked-in to pooled healthy plasma (x-axis). The fraction of covered CpG sites showing values above the baseline cutoff value in each spike-in sample was plotted. As expected, the fraction of detectable CpG sites was elevated at higher spike-in amounts. (A) Spike-ins visualized as a swarm plot with a nonlinear x-axis. (B) Spike-ins visualized as a scatter plot with a linear x-axis.

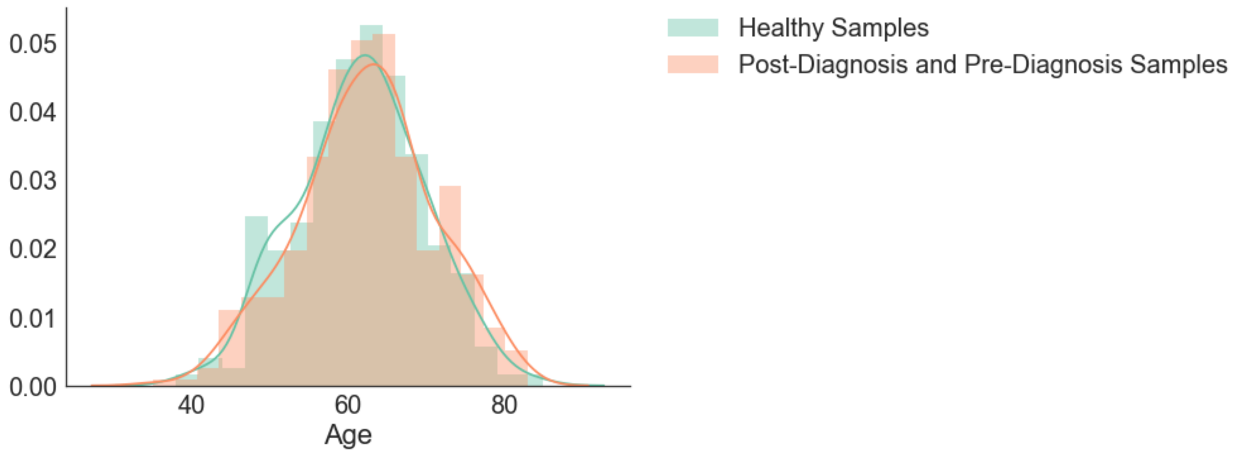

Supplementary Fig. 2.

Age distribution of included Taizhou Longitudinal Study samples for healthy participants and participants diagnosed with cancer. Healthy patient samples were selected for study inclusion by 10-year age window such that age distribution was matched between the two sample groups.

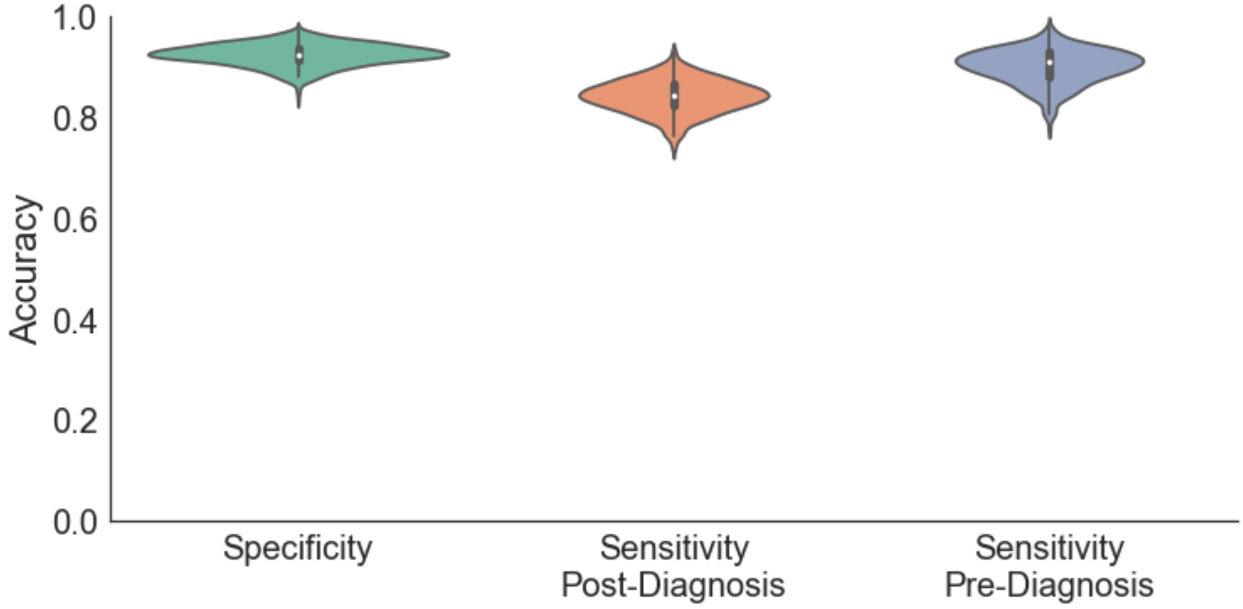

Supplementary Fig. 3.

Cross-validation performance of logistic regression classifier in leave-out samples (n=1,000 simulations). Accuracy remained high regardless of the individual training/test split.

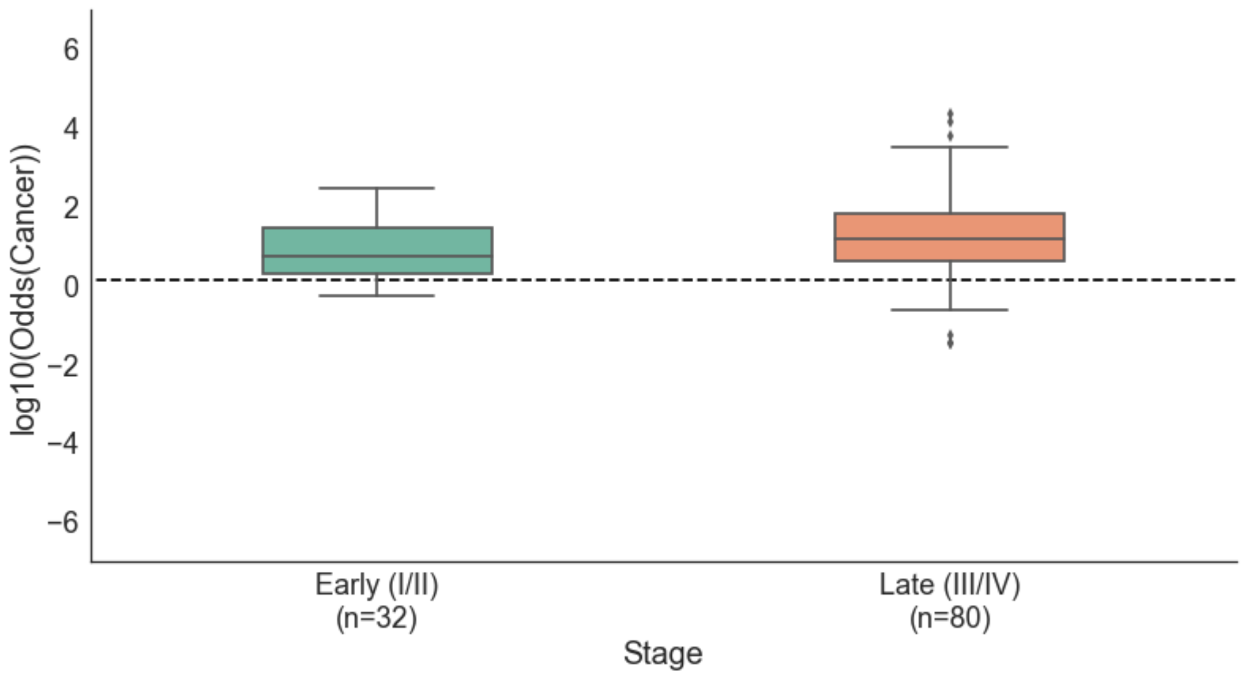

Supplementary Fig. 4.

Box plot of odds of each post-diagnosis sample being called as cancer by the PanSeer model split by cancer stage. Only the test set is presented here. The dashed line represents the cutoff. The distribution of scores for late-stage post-diagnosis cancer samples was similar to the early-stage post-diagnosis samples.

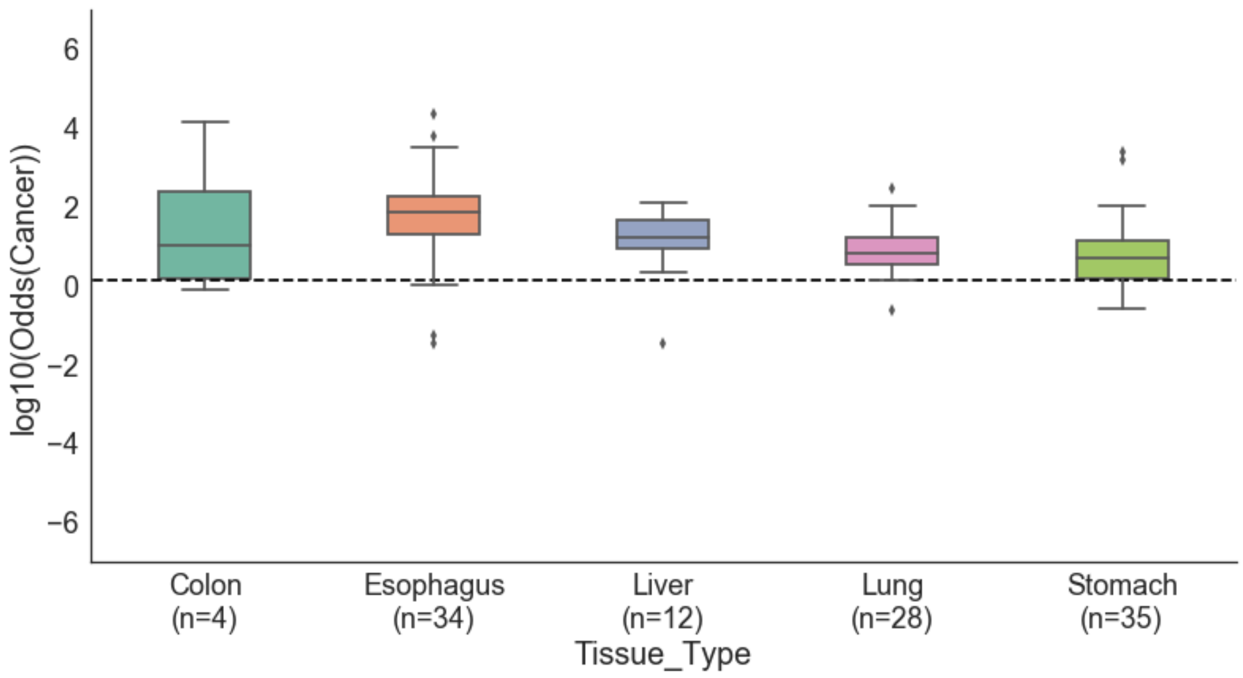

Supplementary Fig. 5.

Box plot of odds of each post-diagnosis sample being called as cancer by the PanSeer model split by tissue type. Only the test set is presented here. The dashed line represents the cutoff. The distribution of scores varied by tissue type.

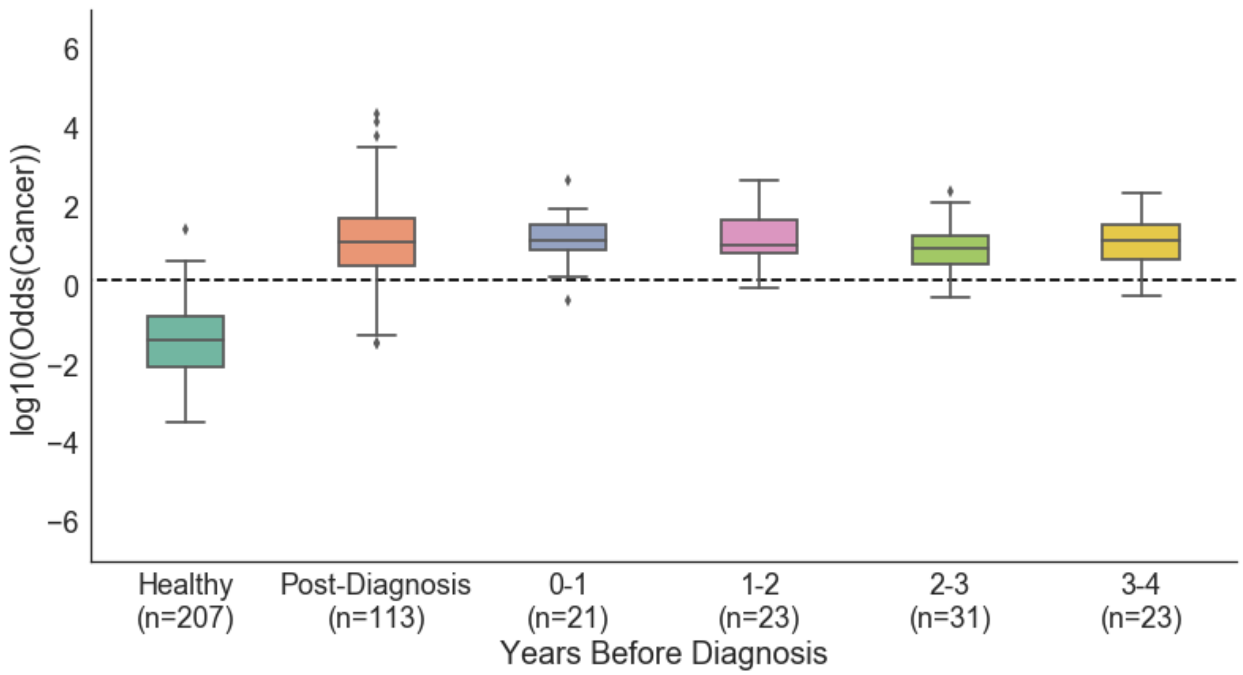

Supplementary Fig. 6.
Box plot of odds of each sample being called as cancer by the PanSeer model split by years before diagnosis. Only the test set is presented here. The dashed line represents the cutoff. The distribution of scores for cancer samples is much higher than the distribution for healthy samples.

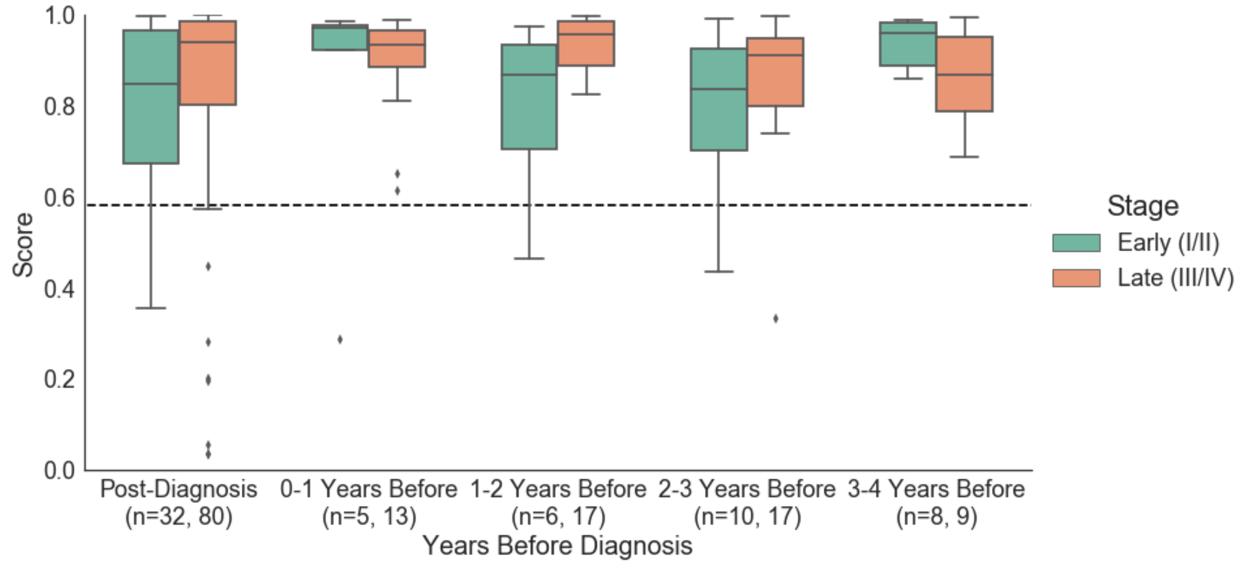

Supplementary Fig. 7
Box plot of PanSeer model score by years before diagnosis and cancer stage. All presented results used only the test set samples. No statistically significant difference was observed by the two-sided Kruskal-Wallis H test, regardless of cancer stage at time of conventional diagnosis; however, this may be due to the low pre-diagnosis sample count (caused by the low incidence rate of cancer in the general population).

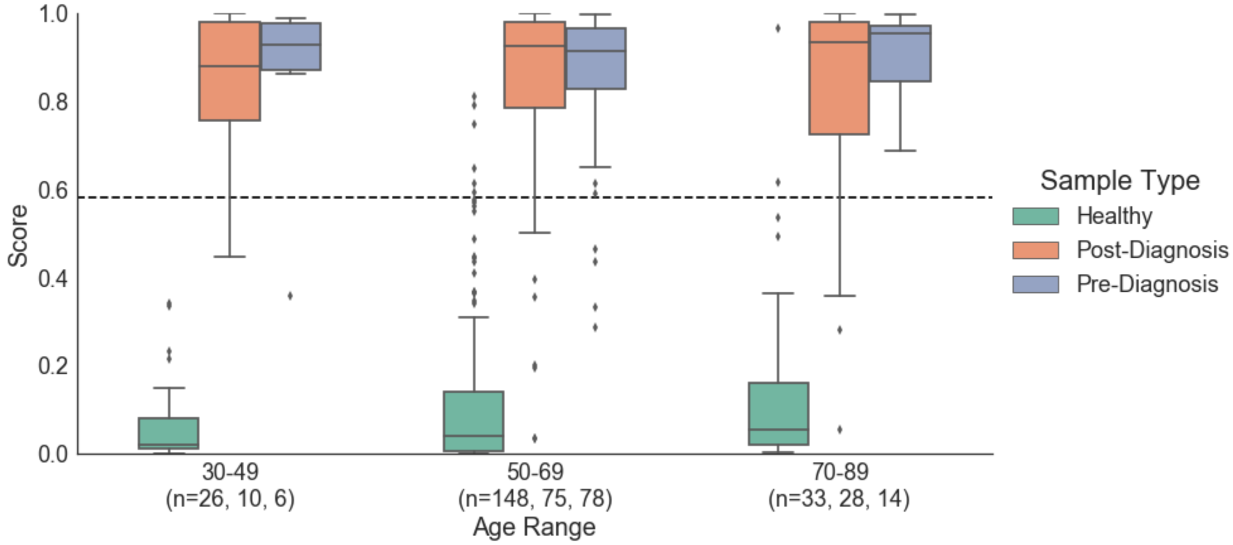

Supplementary Fig. 8
Box plot of PanSeer model score All presented results used only the test set samples. No statistically significant difference was observed by the two-sided Kruskal-Wallis H test.

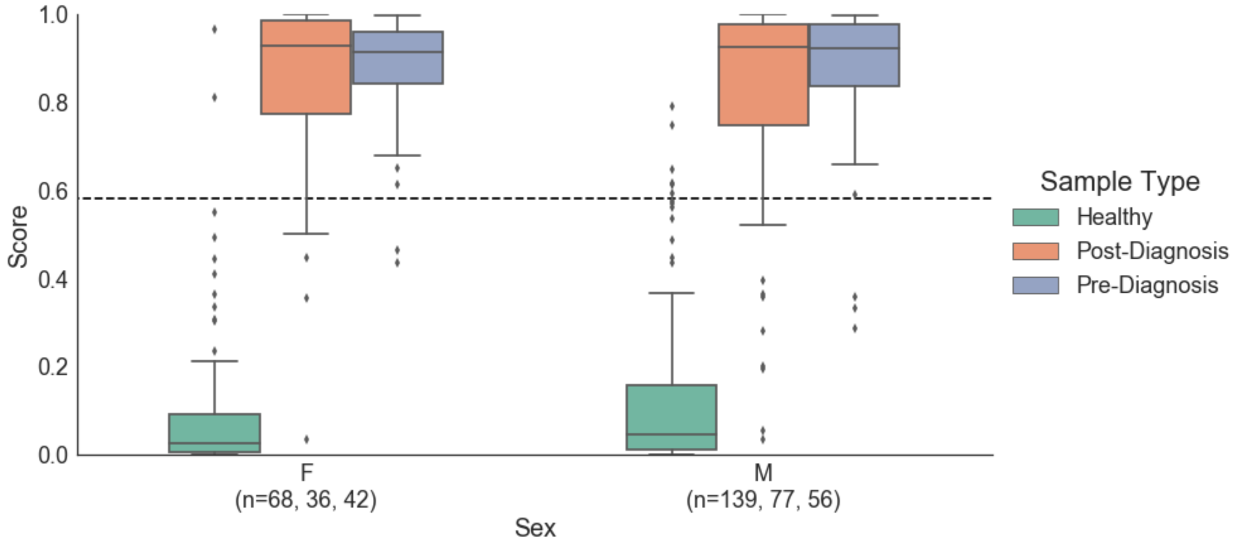

Supplementary Fig. 9
Box plot of PanSeer model score by sex. All presented results used only the test set samples. No statistically significant difference was observed by the two-sided Kruskal-Wallis H test.

A

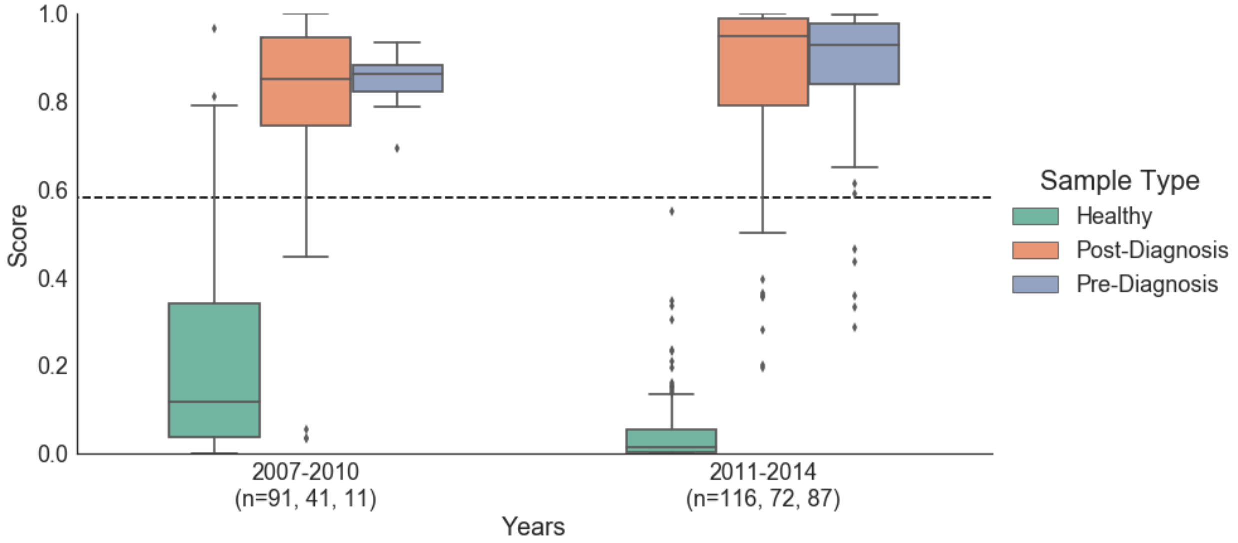

B

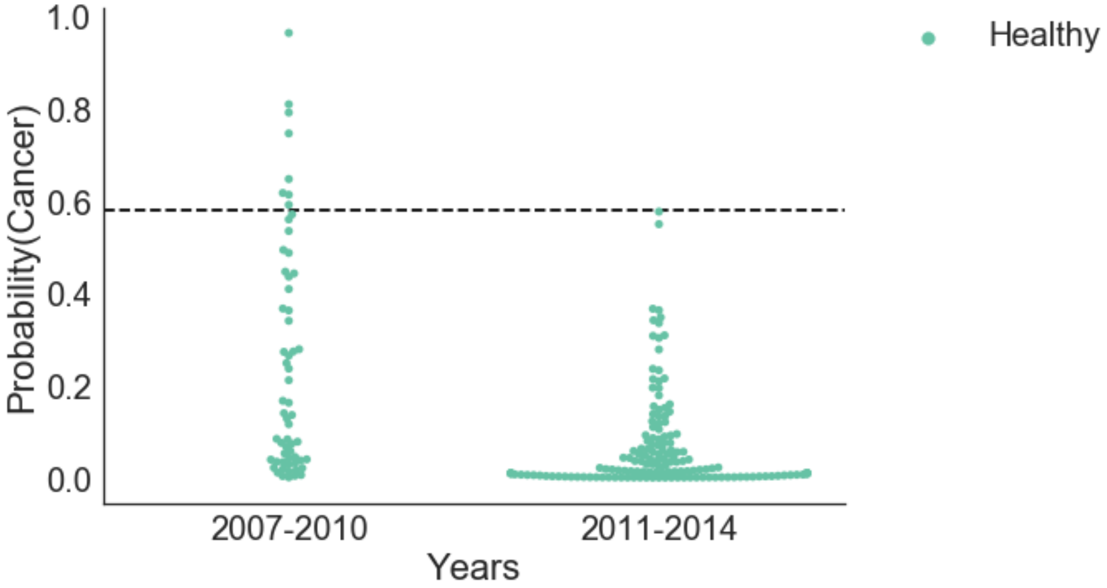

Supplementary Fig. 10
(a) Box plot of PanSeer model score by sample collection date. All presented results used only the test set samples. Statistical testing via the two-sided Kruskal-Wallis H test showed that specificity for healthy samples was reduced in older samples when compared to newer ones ($p = 2.34 \times 10^{-9}$). (b) Distribution of probability of cancer for healthy samples based on sample collection date. Post-hoc testing revealed that samples collected between 2007 and 2010 had a higher median probability of cancer, with a significant magnitude of difference (0.138 vs 0.020, respectively) and a measureable impact on specificity (86.7% vs 100%, respectively). It is possible that the longer storage time for these samples may have caused a reduced specificity due to DNA degradation (see Discussion).

A

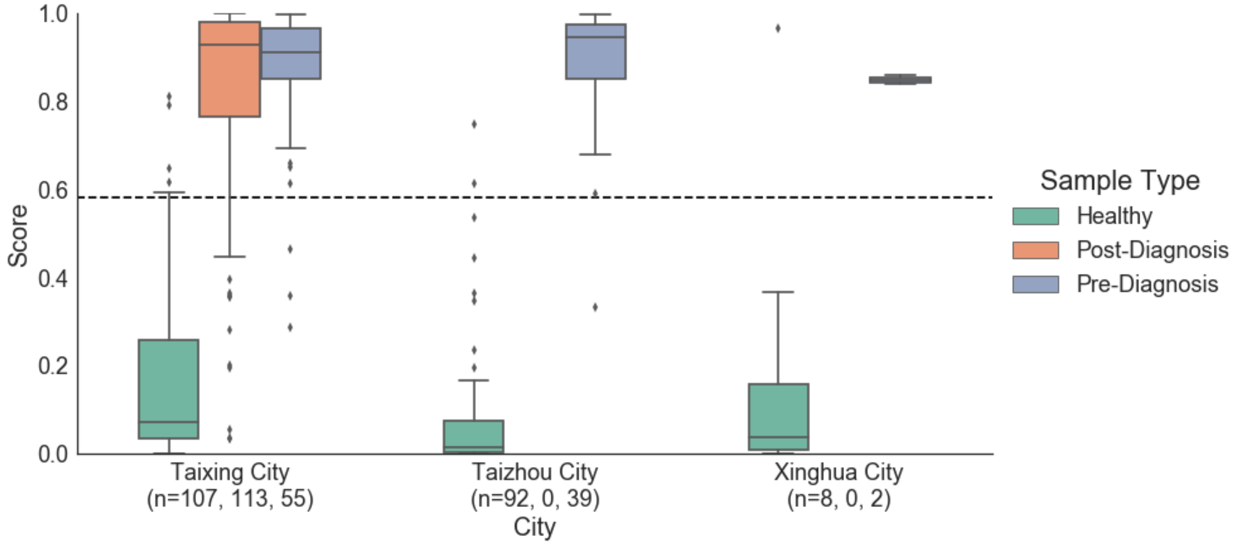

B

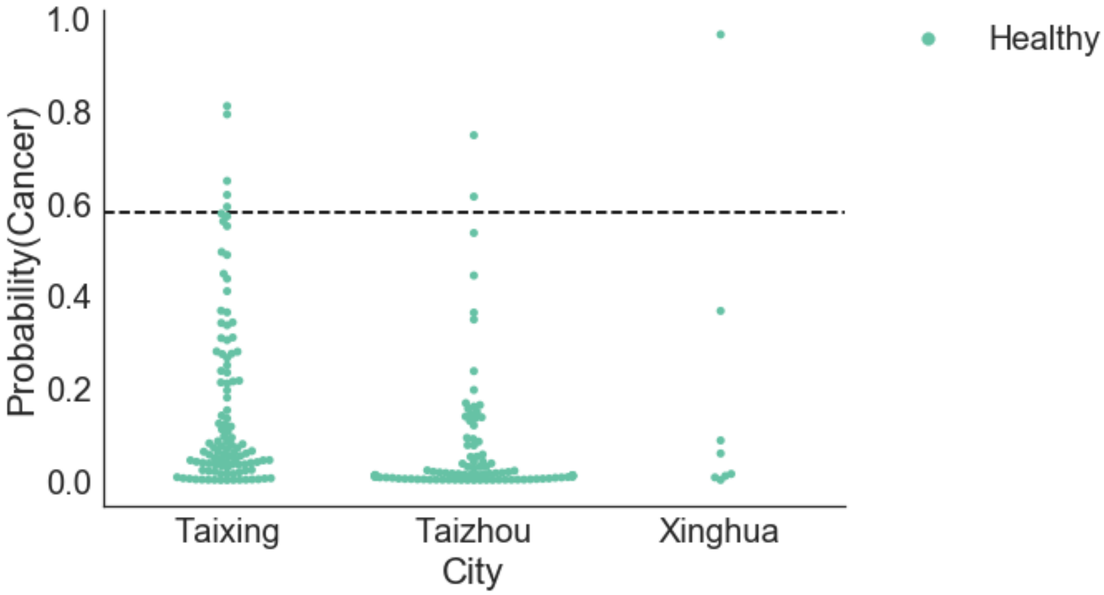

Supplementary Fig. 11

(a) Box plot of PanSeer model score by sample collection location. All presented results used only the test set samples. Statistical testing via the two-sided Kruskal-Wallis H test showed that the probability of cancer for healthy samples was related to collection site ($p = 4.1x10^{-6}$). (b) Distribution of probability of cancer for healthy samples based on sample collection location. Post-hoc testing revealed that samples from Taixing City had higher median probability of cancer; however, the magnitude of difference was small (0.070 vs 0.013 vs 0.036, respectively) and specificity remained high across all cities.

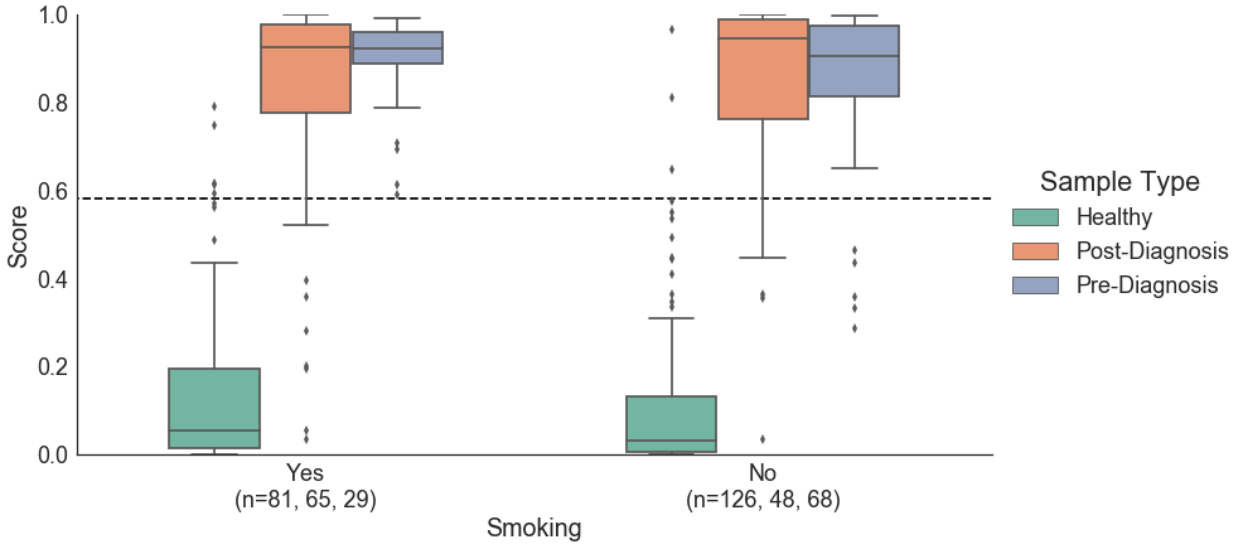

Supplementary Fig. 12

Box plot of PanSeer model score by smoking status. All presented results used only the test set samples. No statistically significant difference was observed by the two-sided Kruskal-Wallis H test.

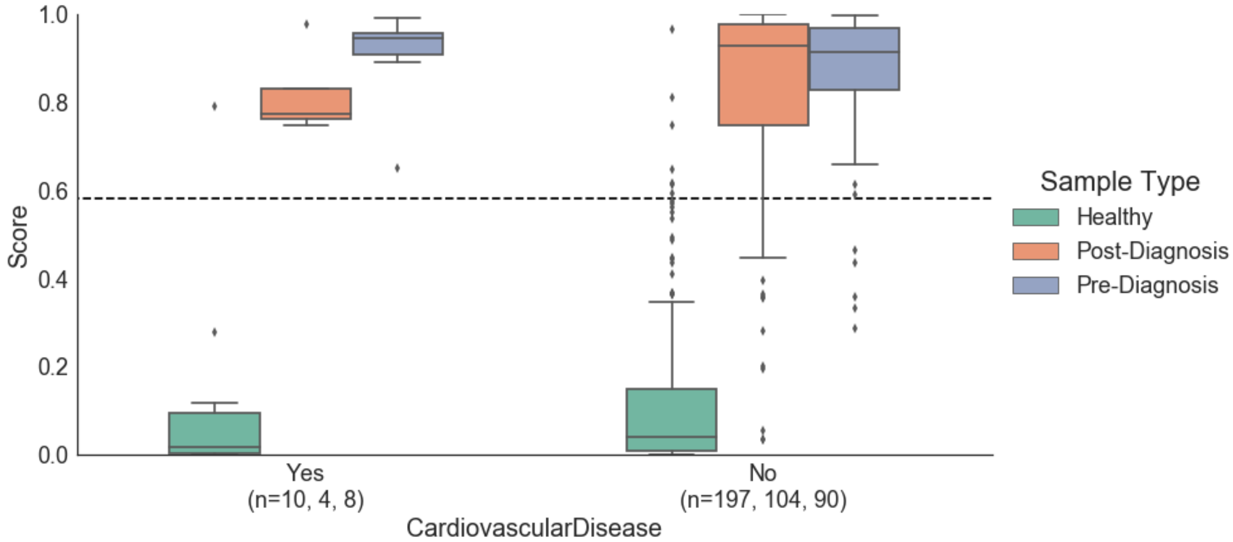

Supplementary Fig. 13
Box plot of PanSeer model score by cardiovascular disease status. All presented results used only the test set samples. No statistically significant difference was observed by the two-sided Kruskal-Wallis H test.

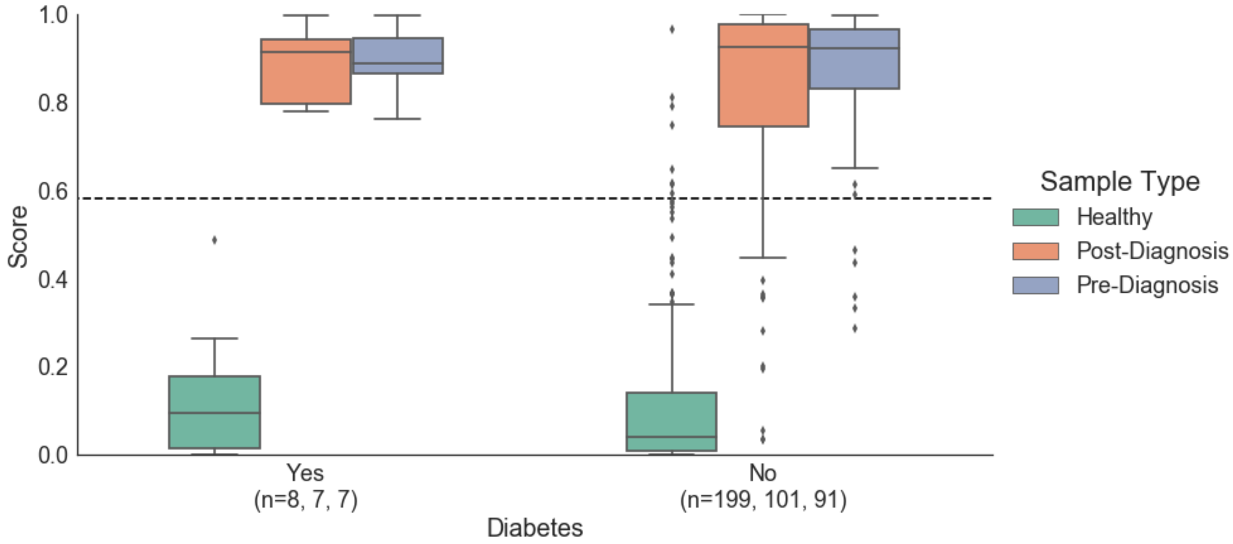

Supplementary Fig. 14

Box plot of PanSeer model score by diabetes status. All presented results used only the test set samples. No statistically significant difference was observed by the two-sided Kruskal-Wallis H test.

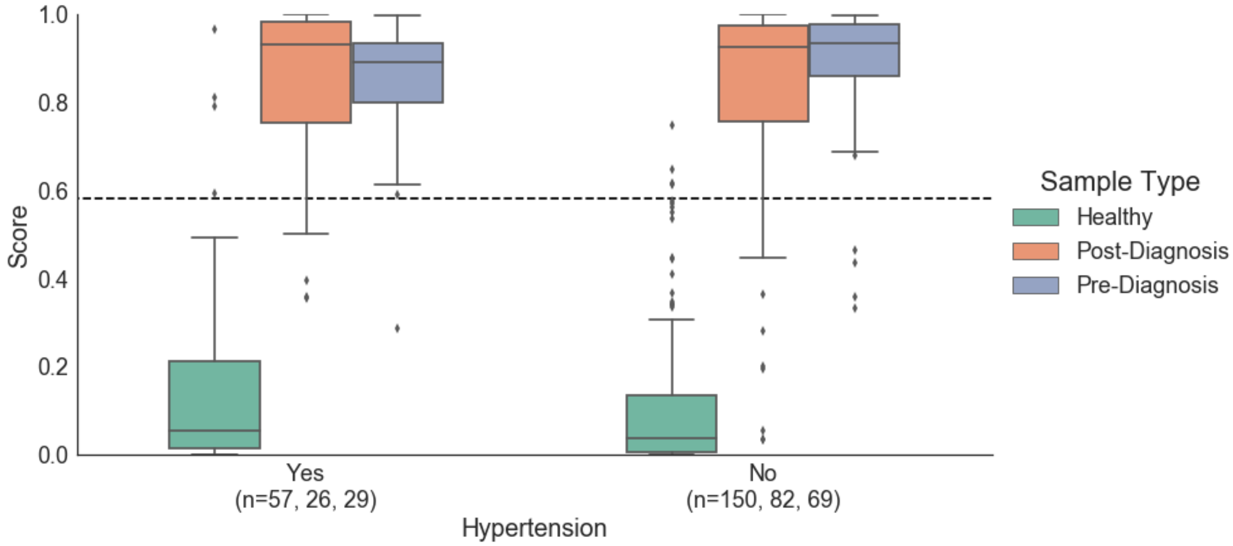

Supplementary Fig. 15

Box plot of PanSeer model score by hypertension status. All presented results used only the test set samples. No statistically significant difference was observed by the two-sided Kruskal-Wallis H test.

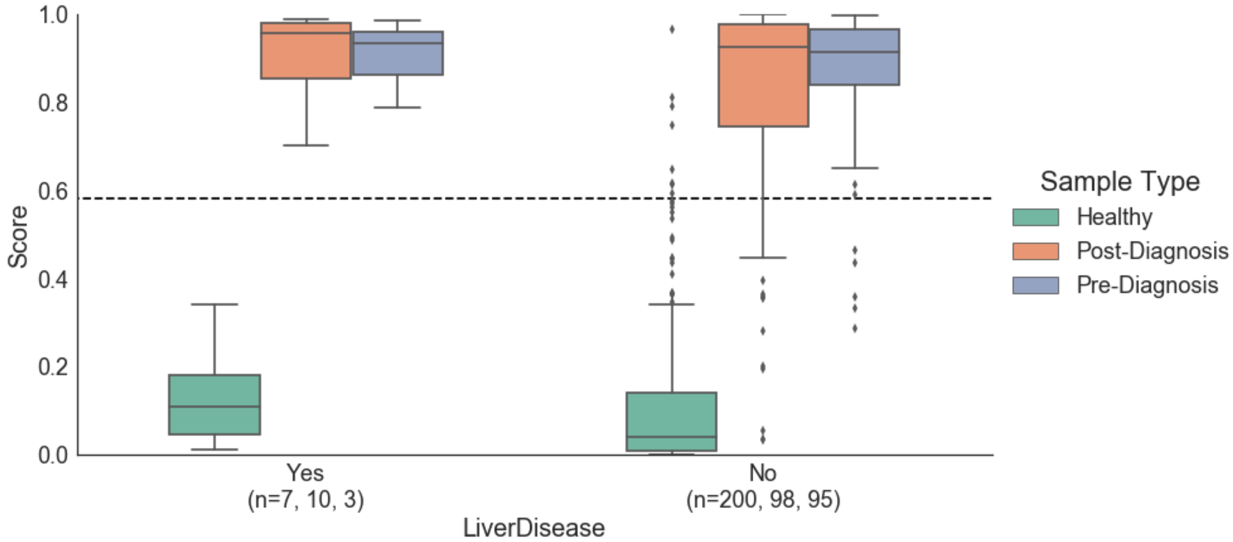

Supplementary Fig. 16
Box plot of PanSeer model score by liver disease status. All presented results used only the test set samples. No statistically significant difference was observed by the two-sided Kruskal-Wallis H test.

A

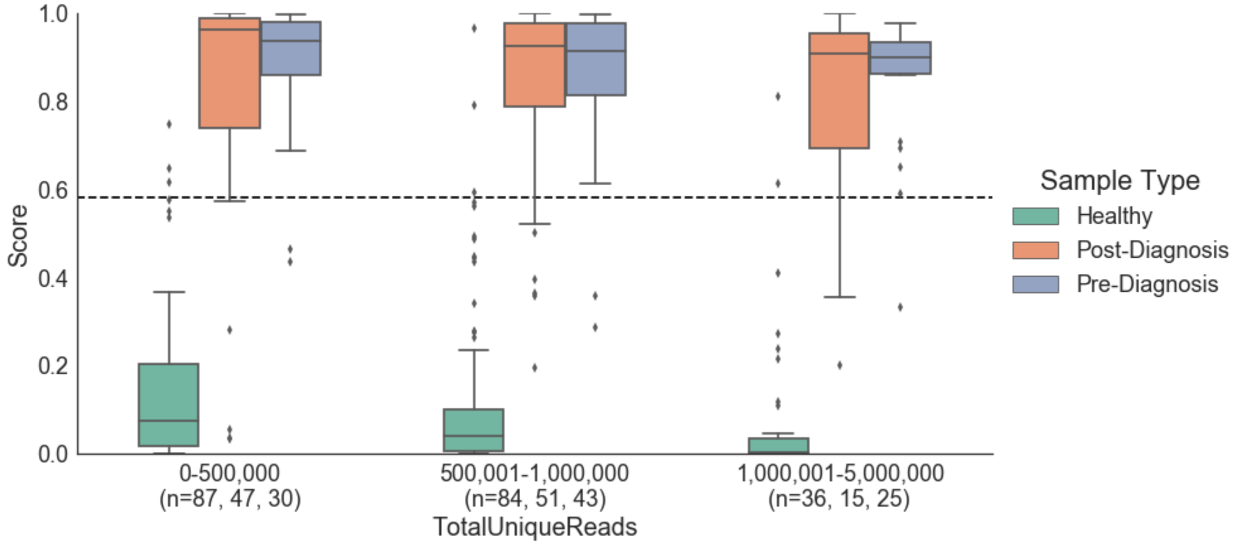

B

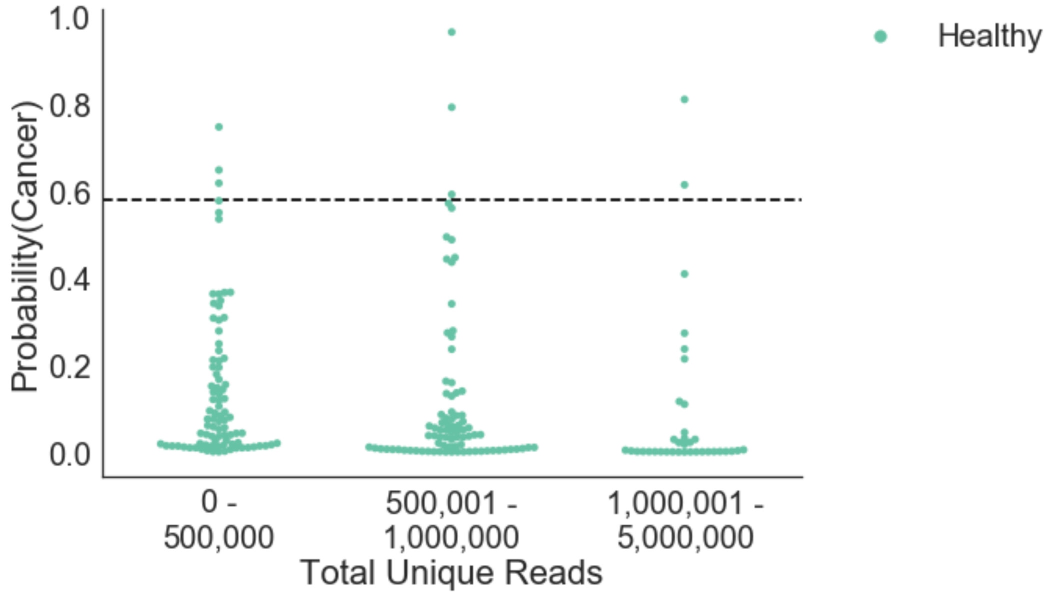

Supplementary Fig. 17

(a) Box plot of PanSeer model score by total unique mapped read count. All presented results used only the test set samples. Statistical testing via the two-sided Kruskal-Wallis H test showed that the probability of cancer for healthy samples was related to unique read count ($p = 2.18x10^{-5}$). (b) Distribution of probability of cancer for healthy samples based on total unique read count. Post-hoc testing revealed that samples with higher total unique read counts had lower median probability of cancer; however, the magnitude of difference was small (0.074 vs 0.039 vs 0.0031, respectively) and specificity remained high across all read counts.

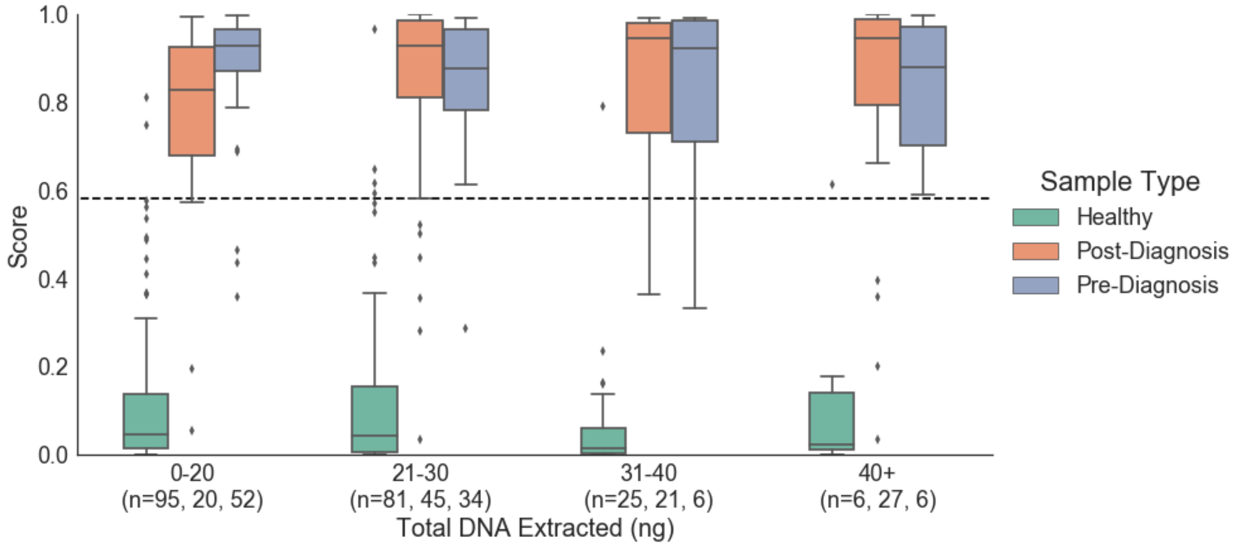

Supplementary Fig. 18
Box plot of PanSeer model score by sample DNA quantity. All presented results used only the test set samples. No statistically significant difference was observed by the two-sided Kruskal-Wallis H test.

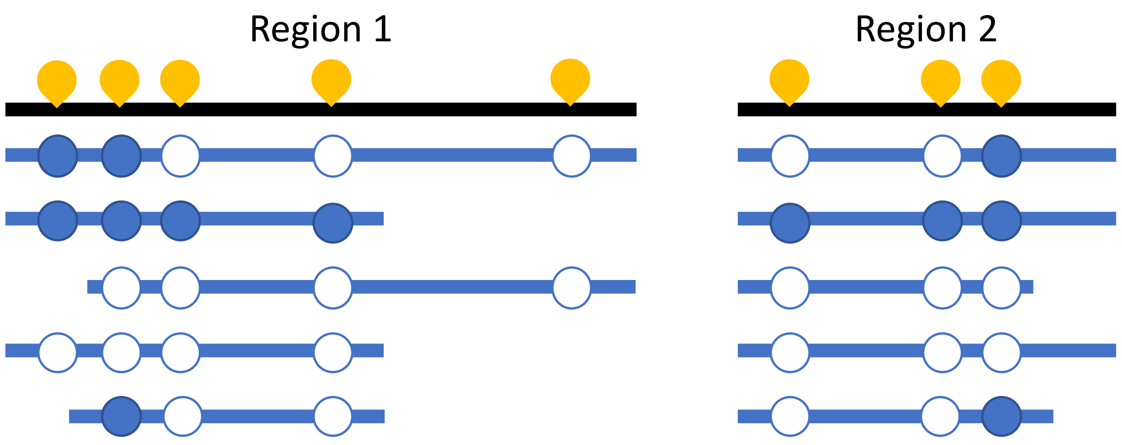

# of Cytosines: 7
# of Thymines: 13

# of Cytosines: 5
# of Thymines: 10

Average Methylation Fraction: 0.35

Average Methylation Fraction: 0.33

Data S1

Region	Num Cs	Num Ts	AMF
1	7	13	0.35
2	5	10	0.33
3	2	18	0.10
	…		

Summarized methylation in each genomic region:
- **AMF** – Average Methylation Fraction

We can now compute the LR equation for this sample. Suppose we had two coefficients (Table S4): 0.75 for Region 1's AMF value, and 0.25 for Region 2's AMF value.

Equation: $\Sigma Mi*Xi = M_1*AMF_1 + M_2*AMF_2 =$
$0.75*0.33 + 0.25*1.0 = 0.25 + 0.25 = 0.5$

Supplementary Fig. 19.
Diagram showing example computation of the Average Methylation Fraction (AMF) metric in targeted regions. AMF values were computed for each targeted genomic region by summing the number of observed cytosines (methylated bases) and dividing by the total number of observed cytosines and thymines (both methylated and unmethylated bases, respectively). These values were combined across all regions and samples into a single data matrix (**Supplementary Data 5**).

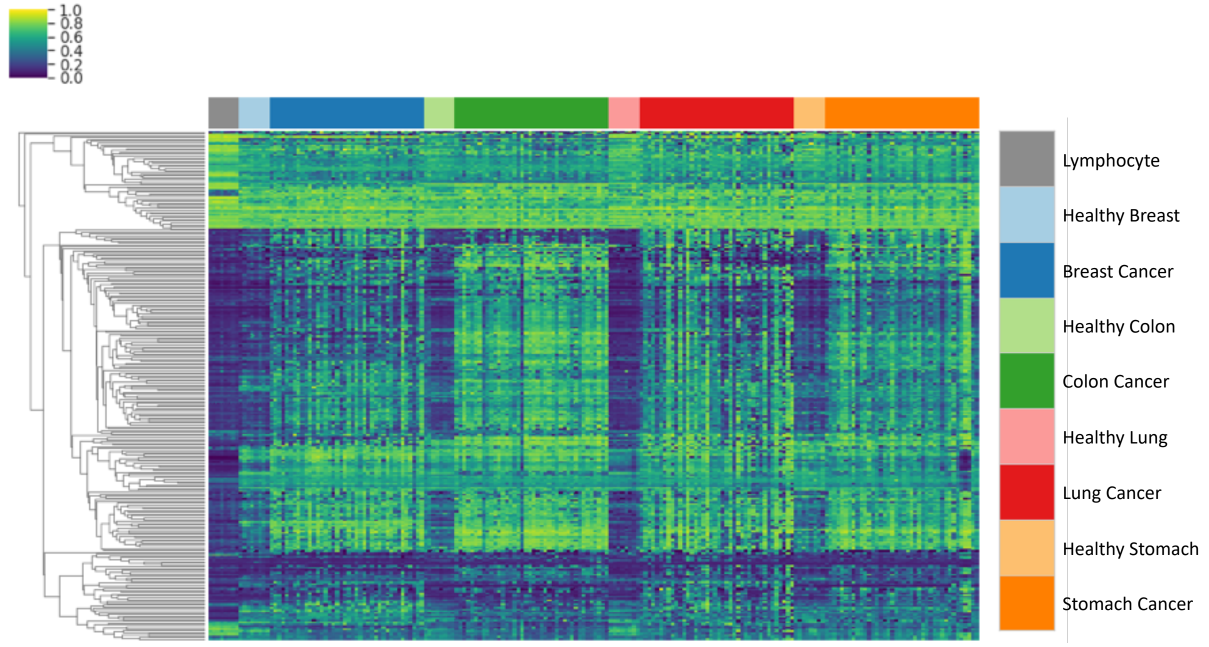

Supplementary Fig. 20

Heatmap of differential methylation between healthy and cancer tissues in PanSeer target regions. Primary healthy and cancer tissue samples were purchased from Biochain. The PanSeer target regions showed differential methylation bewteen cancer tissue and healthy tissue (as well as healthy lymphocytes).

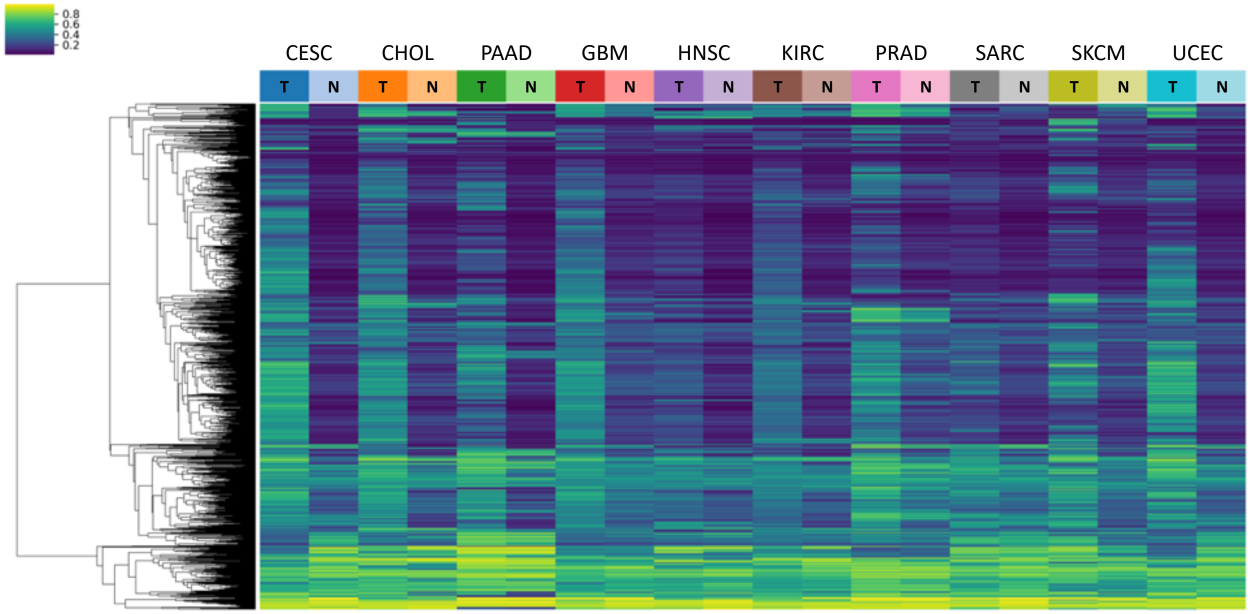

Supplementary Fig. 21

Heatmap of differential methylation between healthy and cancer tissues in the TCGA dataset across PanSeer target regions. PanSeer target regions were intersected with CpG sites covered by the TCGA 450k array data set, and the average methylation values across tissue types not present in the Biochain sample set (cervix CESC, cholangiocarcinoma CHOL, pancreatic PAAD, glioblastoma GBM, head/neck squamous cell HNSC, kidney KIRC, prostate PRAD, sarcoma SARC, skin SKCM, and uterine UCEC, respectively) were plotted; tumor samples were labeled "T" while healthy samples were labeled "N." The intersected CpG sites showed differential methylation bewteen cancer tissue and healthy tissue across many tissue types in the TCGA dataset.

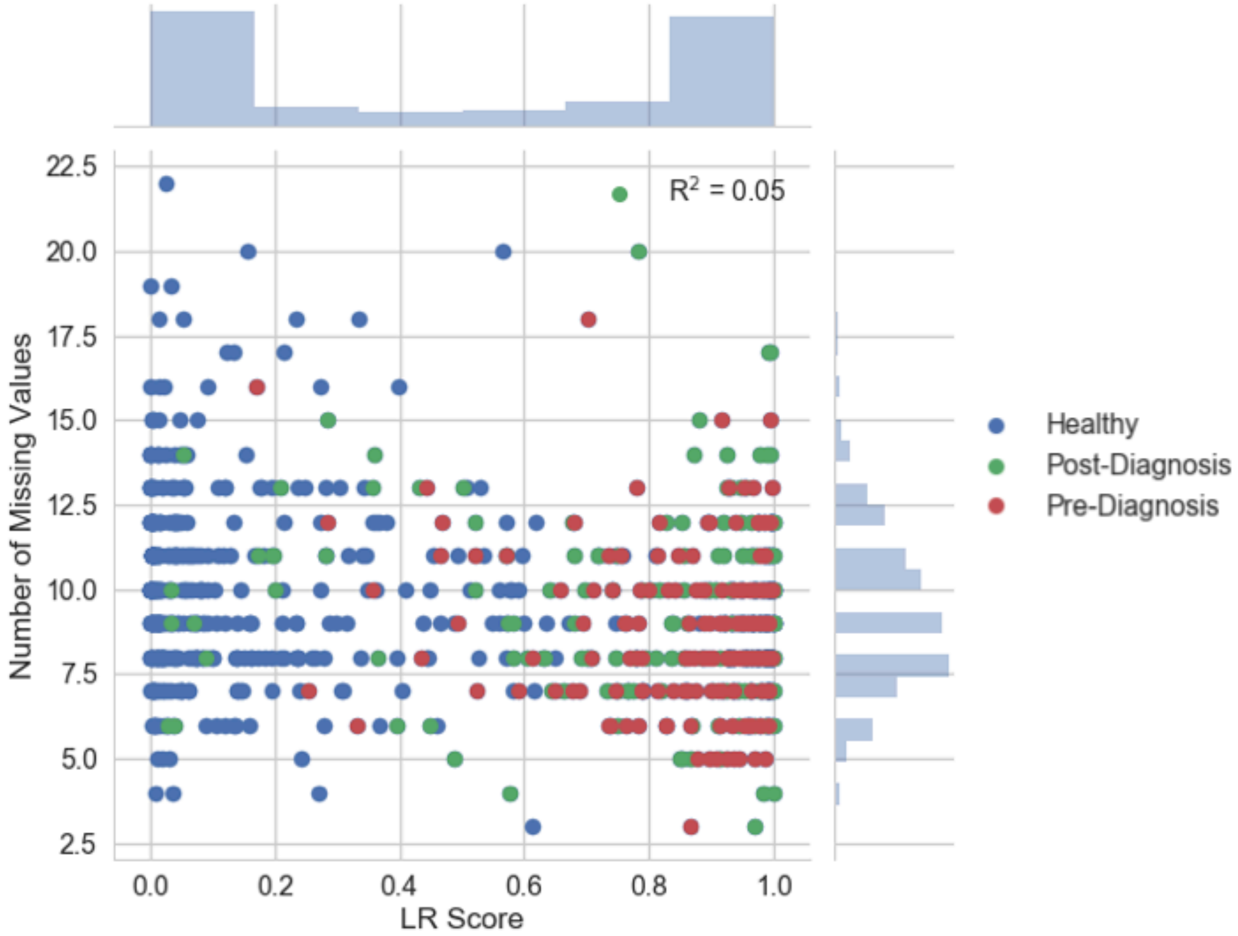

Supplementary Fig. 22
Comparison of number of missing values to logistic regression score. In order to further ensure that lack of coverage of certain genomic regions did not affect the model results, we plotted a comparison of the number of missing methylation values in each sample versus the logistic regression score. No correlation was observed between the number of NA values and the LR score, indicating that the cancer detection model is not biased by samples with missing values.

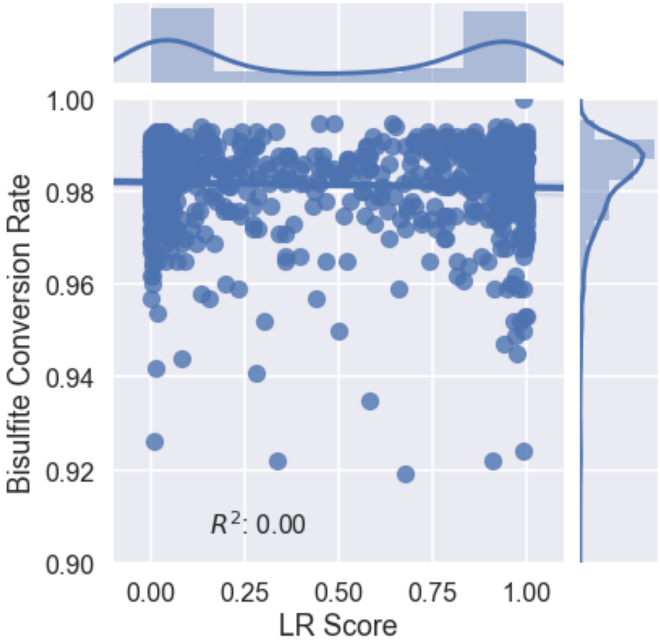

Supplementary Fig. 23
Comparison of bisulfite conversion rate to logistic regression score. In order to further ensure that the efficiency of bisulfite conversion in a given sample did not affect the model results, we we plotted a comparison of bisulfite conversion rate in each sample versus the logistic regression score. No correlation was observed between the bisulfite conversion rate and the LR score, indicating that the cancer detection model is not biased by variability in bisulfite conversion.

Tumor Type	Total Cases	Stage	Number of Patients	Years Before Diagnosis				
				Post-Diagnosis	0-1	1-2	2-3	3-4
**Colon**	42	I-II	21	2	3	4	7	5
		III-IV	16	5	3	6	0	2
		Unknown	5	0	1	0	1	3
**Esophagus**	113	I-II	46	29	3	4	5	5
		III-IV	63	39	8	5	7	4
		Unknown	4	0	1	0	1	2
**Stomach**	104	I-II	44	34	1	2	3	4
		III-IV	54	35	6	6	4	3
		Unknown	6	0	1	1	1	3
**Liver**	52	I-II	7	3	0	2	1	1
		III-IV	43	20	7	7	2	7
		Unknown	2	0	1	0	0	1
**Lung**	103	I-II	18	10	2	1	2	3
		III-IV	80	45	6	6	14	9
		Unknown	5	1	0	0	2	2

Supplementary Table 1

Summary of cancer tissues-of-origin and stages for post-diagnosis and pre-diagnosis samples.

Supplementary Note 1.
Code utilized to derive and implement a Logistic Regression classifier. A leave-in set of samples was used to build the classifier, and a leave-out set of samples was utilized to validate the classifier.

Supplementary Note 1

In [1]:
```python
from __future__ import division
import sys
import os
import glob
import random
```

In [2]:
```python
import pandas as pd
import numpy as np
import scipy
from sklearn.linear_model import LogisticRegression, LogisticRegressionCV
from sklearn.metrics import roc_curve, auc
from statsmodels.stats.multitest import multipletests
```

In [3]:
```python
%matplotlib inline
import seaborn as sns
import matplotlib.pyplot as plt
```

In [4]:
```python
sns.set()
sns.set_context('notebook', font_scale=1.2)
sns.set_style('white')
```

Sample Info

For this study, we processed two sets of samples; one set of plasma samples from the Taizhou Longitudinal Study, and a second set of commercially purchsed normal and cancer tissue samples from BioChain.

Taizhou Longitudinal Study

In [5]:
```python
pre_diagnosis_samples = ['TSH-PL180123001', 'TSH-PL180123006', 'TSH-PL180123007', 'TSH-PL180123008', 'TSH-PL180123010', 'TSH-PL180123013', 'TSH-PL180123014', 'TSH-PL180123016', 'TSH-PL180123017', 'TSH-PL180123018', 'TSH-PL180123021', 'TSH-PL180123022', 'TSH-PL180123025', 'TSH-PL180123027', 'TSH-PL180123029', 'TSH-PL180123030', 'TSH-PL180123031', 'TSH-PL180123033', 'TSH-PL180513004', 'TSH-PL180513005', 'TSH-PL180513006', 'TSH-PL180513007', 'TSH-PL180513011', 'TSH-PL180513012', 'TSH-PL180513013', 'TSH-PL180513014', 'TSH-PL180513016', 'TSH-PL180513017', 'TSH-PL180513020', 'TSH-PL180513021', 'TSH-PL180513023', 'TSH-PL180513024', 'TSH-PL180513025', 'TSH-PL180513026', 'TSH-PL180513027', 'TSH-PL180513029', 'TSH-PL180513030', 'TSH-PL180513034', 'TSH-PL180513036', 'TSH-PL180513037', 'TSH-PL180513038', 'TSH-PL180513039', 'TSH-PL180513040', 'TSH-PL180513041', 'TSH-PL180513043', 'TSH-PL180513045', 'TSH-PL180513049', 'TSH-PL180513050', 'TSH-PL180513051', 'TSH-PL180513055', 'TSH-PL180513056', 'TSH-PL180513057', 'TSH-PL180513060', 'TSH-PL180513062', 'TSH-PL180513066', 'TSH-PL180513067', 'TSH-PL180513068', 'TSH-PL180513069', 'TSH-PL180513070', 'TSH-PL180513071', 'TSH-PL180513072', 'TSH-PL180513073', 'TSH-PL180513075',
'TSH-PL180513076', 'TSH-PL180513077', 'TSH-PL180513078', 'TSH-PL180513079', 'TSH-PL180513080',
'TSH-PL180513081', 'TSH-PL180513084', 'TSH-PL180513085', 'TSH-PL180513087', 'TSH-PL180513088',
'TSH-PL180513089', 'TSH-PL180513091', 'TSH-PL180513092', 'TSH-PL180513093', 'TSH-PL180513095',
'TSH-PL180513097', 'TSH-PL180513098', 'TSH-PL180513099', 'TSH-PL180513100', 'TSH-PL180513101',
'TSH-PL180513102', 'TSH-PL180513104', 'TSH-PL180513106', 'TSH-PL180513108', 'TSH-PL180513109',
'TSH-PL180513110', 'TSH-PL180513111', 'TSH-PL180513114', 'TSH-PL180513115', 'TSH-PL180513117',
'TSH-PL180513118', 'TSH-PL180513119', 'TSH-PL180513121', 'TSH-PL180513122', 'TSH-PL180513123',
'TSH-PL180513124', 'TSH-PL180513127', 'TSH-PL180513128', 'TSH-PL180513129', 'TSH-PL180516001',
'TSH-PL180516002', 'TSH-PL180516003', 'TSH-PL180516004', 'TSH-PL180516006', 'TSH-PL180516007',
'TSH-PL180516008', 'TSH-PL180516009', 'TSH-PL180516011', 'TSH-PL180516012', 'TSH-PL180516013',
'TSH-PL180516014', 'TSH-PL180516016', 'TSH-PL180516018', 'TSH-PL180516019', 'TSH-PL180516020',
'TSH-PL180516021', 'TSH-PL180516023', 'TSH-PL180516027', 'TSH-PL180516028', 'TSH-PL180516030',
'TSH-PL180516031', 'TSH-PL180516032', 'TSH-PL180516036', 'TSH-PL180516038', 'TSH-PL180516039',
'TSH-PL180516040', 'TSH-PL180516042', 'TSH-PL180516043', 'TSH-PL180516044', 'TSH-PL180516045',
'TSH-PL180516049', 'TSH-PL180516051', 'TSH-PL180516053', 'TSH-PL180516054', 'TSH-PL180516055',
'TSH-PL180516056', 'TSH-PL180516057', 'TSH-PL180516059', 'TSH-PL180516062', 'TSH-PL180516063',
'TSH-PL180123002', 'TSH-PL180123004', 'TSH-PL180123005', 'TSH-PL180123012', 'TSH-PL180123024',
'TSH-PL180123032', 'TSH-PL180513001', 'TSH-PL180513002', 'TSH-PL180513003', 'TSH-PL180513009',
'TSH-PL180513010', 'TSH-PL180513018', 'TSH-PL180513019', 'TSH-PL180513022', 'TSH-PL180513028',
'TSH-PL180513031', 'TSH-PL180513032', 'TSH-PL180513033', 'TSH-PL180513035', 'TSH-PL180513044',
'TSH-PL180513058', 'TSH-PL180513064', 'TSH-PL180513083', 'TSH-PL180513086', 'TSH-PL180513090',
'TSH-PL180513096', 'TSH-PL180513103', 'TSH-PL180513105', 'TSH-PL180513112', 'TSH-PL180513116',
'TSH-PL180513125', 'TSH-PL180516005', 'TSH-PL180516010', 'TSH-PL180516015', 'TSH-PL180516017',
'TSH-PL180516025', 'TSH-PL180516026', 'TSH-PL180516029', 'TSH-PL180516033', 'TSH-PL180516034',
'TSH-PL180516046', 'TSH-PL180516047', 'TSH-PL180516048', 'TSH-PL180516050', 'TSH-PL180516052',
'TSH-PL180516058', 'TSH-PL180516060', 'TSH-PL180516061']
post_diagnosis_samples = ['TSH-PL171221101', 'TSH-PL171221112', 'TSH-PL171221149', 'TSH-PL171221150', 'TSH-PL171221160', 'TSH-PL171221172', 'TSH-PL171221179', 'TSH-PL171221180', 'TSH-PL180504001', 'TSH-PL180504002', 'TSH-PL180504003', 'TSH-PL180504004', 'TSH-PL180504005', 'TSH-PL180504009', 'TSH-PL180504010', 'TSH-PL180504011', 'TSH-PL180504013', 'TSH-PL180504014', 'TSH-PL180504015', 'TSH-PL180504016', 'TSH-PL180504017', 'TSH-PL180504018', 'TSH-PL180504019', 'TSH-PL180504021', 'TSH-PL180504022', 'TSH-PL180504027', 'TSH-PL180504028', 'TSH-PL180504030', 'TSH-PL180504033', 'TSH-PL180504034', 'TSH-PL180504041', 'TSH-PL180504042', 'TSH-PL180504044', 'TSH-PL180504046', 'TSH-PL180504047', 'TSH-PL180504049', 'TSH-PL180504050', 'TSH-PL180504053', 'TSH-PL180504054', 'TSH-PL180504058', 'TSH-PL180504061', 'TSH-PL180504062', 'TSH-PL180504063', 'TSH-PL180504065', 'TSH-PL180504068', 'TSH-PL180504070', 'TSH-PL180504074', 'TSH-PL180504081', 'TSH-PL180504083', 'TSH-PL180504087', 'TSH-PL180504088', 'TSH-PL180504089', 'TSH-PL180504091', 'TSH-PL180504094', 'TSH-PL180504097', 'TSH-PL180504101', 'TSH-PL180504104', 'TSH-PL180504108',
'TSH-PL180504111', 'TSH-PL180504118', 'TSH-PL180508001', 'TSH-PL180508004', 'TSH-PL180508005',
'TSH-PL180508006', 'TSH-PL180508008', 'TSH-PL180508009', 'TSH-PL180508011', 'TSH-PL180508012',
'TSH-PL180508013', 'TSH-PL180508014', 'TSH-PL180508015', 'TSH-PL180508019', 'TSH-PL180508020',
'TSH-PL180508021', 'TSH-PL180508022', 'TSH-PL180508025', 'TSH-PL180508030', 'TSH-PL180508031',
'TSH-PL180508033', 'TSH-PL180508034', 'TSH-PL180508036', 'TSH-PL180508037', 'TSH-PL180508038',
'TSH-PL180508039', 'TSH-PL180508040', 'TSH-PL180508041', 'TSH-PL180508042', 'TSH-PL180508043',
'TSH-PL180508049', 'TSH-PL180508050', 'TSH-PL180508060', 'TSH-PL180508061', 'TSH-PL180508064',
'TSH-PL180508068', 'TSH-PL180508076', 'TSH-PL180508078', 'TSH-PL180508083', 'TSH-PL180508086',
'TSH-PL180508089', 'TSH-PL180510005', 'TSH-PL180510006', 'TSH-PL180510007', 'TSH-PL180510008',
'TSH-PL180510010', 'TSH-PL180510011', 'TSH-PL180510012', 'TSH-PL180510014', 'TSH-PL180510015',
'TSH-PL180510016', 'TSH-PL180510017', 'TSH-PL180510018', 'TSH-PL180510019', 'TSH-PL180510021',
'TSH-PL180510023', 'TSH-PL180510024', 'TSH-PL180510025', 'TSH-PL180510026', 'TSH-PL180510030',
'TSH-PL180510031', 'TSH-PL180510033', 'TSH-PL180510038', 'TSH-PL180510040', 'TSH-PL180510044',
'TSH-PL180510047', 'TSH-PL180510050', 'TSH-PL180510052', 'TSH-PL180510053', 'TSH-PL180510054',
'TSH-PL180510055', 'TSH-PL180510057', 'TSH-PL180510061', 'TSH-PL180510063', 'TSH-PL180510066',
'TSH-PL180510071', 'TSH-PL180510072', 'TSH-PL180510073', 'TSH-PL180510074', 'TSH-PL180510075',
'TSH-PL180510076', 'TSH-PL180510077', 'TSH-PL180510078', 'TSH-PL180510079', 'TSH-PL180510080',
'TSH-PL180510085', 'TSH-PL180510089', 'TSH-PL180510092', 'TSH-PL180510095', 'TSH-PL180510101',
'TSH-PL180510104', 'TSH-PL180510105', 'TSH-PL180510107', 'TSH-PL180510112', 'TSH-PL180510121',
'TSH-PL180510122', 'TSH-PL180510125', 'TSH-PL180510139', 'TSH-PL180510140', 'TSH-PL180510146',
'TSH-PL180510147', 'TSH-PL180510162', 'TSH-PL181205168', 'TSH-PL181205170', 'TSH-PL181205173',
'TSH-PL181205175', 'TSH-PL181205182', 'TSH-PL171221151', 'TSH-PL180504006', 'TSH-PL180504012',
'TSH-PL180504024', 'TSH-PL180504026', 'TSH-PL180504029', 'TSH-PL180504037', 'TSH-PL180504043',
'TSH-PL180504045', 'TSH-PL180504051', 'TSH-PL180504052', 'TSH-PL180504055', 'TSH-PL180504057',
'TSH-PL180504064', 'TSH-PL180504076', 'TSH-PL180504084', 'TSH-PL180504110', 'TSH-PL180508003',
'TSH-PL180508007', 'TSH-PL180508023', 'TSH-PL180508026', 'TSH-PL180508028', 'TSH-PL180508029',
'TSH-PL180508032', 'TSH-PL180508044', 'TSH-PL180508052', 'TSH-PL180508065', 'TSH-PL180508067',
'TSH-PL180508088', 'TSH-PL180510002', 'TSH-PL180510004', 'TSH-PL180510013', 'TSH-PL180510020',
'TSH-PL180510022', 'TSH-PL180510032', 'TSH-PL180510034', 'TSH-PL180510035', 'TSH-PL180510043',
'TSH-PL180510048', 'TSH-PL180510068', 'TSH-PL180510088', 'TSH-PL180510090', 'TSH-PL180510098',
'TSH-PL180510100', 'TSH-PL180510103', 'TSH-PL180510108', 'TSH-PL180510109', 'TSH-PL180510113',
'TSH-PL180510117', 'TSH-PL180510124', 'TSH-PL180510143', 'TSH-PL181205169', 'TSH-PL181205172',
'TSH-PL181205174', 'TSH-PL181205177', 'TSH-PL181205179', 'TSH-PL171221153', 'TSH-PL171221113']
healthy_samples = ['TSH-PL180309087', 'TSH-PL180316003', 'TSH-PL180316005', 'TSH-PL180316016',
'TSH-PL180316017', 'TSH-PL180316018', 'TSH-PL180316022', 'TSH-PL180316024', 'TSH-PL180316027',
'TSH-PL180316033', 'TSH-PL180316037', 'TSH-PL180316038', 'TSH-PL180316039', 'TSH-PL180316049',
'TSH-PL180316061', 'TSH-PL180316065', 'TSH-PL180316079', 'TSH-PL180316092', 'TSH-PL180316095',
'TSH-PL180316099', 'TSH-PL180316101', 'TSH-PL180316106', 'TSH-PL180316115', 'TSH-PL180316119',
'TSH-PL180316126', 'TSH-PL180316129', 'TSH-PL180316157', 'TSH-PL180316161', 'TSH-PL180316163',
'TSH-PL180316164', 'TSH-PL180316173', 'TSH-PL180316174', 'TSH-PL180316181', 'TSH-PL180316183',
'TSH-PL180316187', 'TSH-PL180316190', 'TSH-PL180316195', 'TSH-PL180316196', 'TSH-PL180316198',
'TSH-PL180323034', 'TSH-PL180323038', 'TSH-PL180323051', 'TSH-PL180323052', 'TSH-PL180323054',
'TSH-PL180323059', 'TSH-PL180323065', 'TSH-PL180323067', 'TSH-PL180323072', 'TSH-PL180323073',
'TSH-PL180323091', 'TSH-PL180323106', 'TSH-PL180323107', 'TSH-PL180323109', 'TSH-PL180323110',
'TSH-PL180323111', 'TSH-PL180323119', 'TSH-PL180323121', 'TSH-PL180323124', 'TSH-PL180323139',
'TSH-PL180323141', 'TSH-PL180323142', 'TSH-PL180323151', 'TSH-PL180323155', 'TSH-PL180323166',
'TSH-PL180410003', 'TSH-PL180410023', 'TSH-PL180410043', 'TSH-PL180410049', 'TSH-PL180410059',
'TSH-PL180410063', 'TSH-PL180410076', 'TSH-PL180410087', 'TSH-PL180410115', 'TSH-PL180410116',
'TSH-PL180410133', 'TSH-PL180410140', 'TSH-PL180410144', 'TSH-PL180410160', 'TSH-PL180410162',
'TSH-PL180410198', 'TSH-PL180417003', 'TSH-PL180417007', 'TSH-PL180417035', 'TSH-PL180417060',
'TSH-PL180417074', 'TSH-PL180417087', 'TSH-PL180417095', 'TSH-PL180417101', 'TSH-PL180417109',
'TSH-PL180417117', 'TSH-PL180417135', 'TSH-PL180417138', 'TSH-PL180417143', 'TSH-PL180417152',
'TSH-PL180417156', 'TSH-PL180417165', 'TSH-PL180417170', 'TSH-PL180417179', 'TSH-PL180417180',
'TSH-PL180417181', 'TSH-PL180417200', 'TSH-PL180424003', 'TSH-PL180424030', 'TSH-PL180424036',
'TSH-PL180424058', 'TSH-PL180424090', 'TSH-PL180424097', 'TSH-PL180424104', 'TSH-PL180424115',
'TSH-PL180424146', 'TSH-PL180424149', 'TSH-PL180424155', 'TSH-PL180424162', 'TSH-PL180424166',
'TSH-PL180424171', 'TSH-PL180703037', 'TSH-PL180703136', 'TSH-PL180703153', 'TSH-PL180703157',
'TSH-PL180703185', 'TSH-PL180706034', 'TSH-PL180706037', 'TSH-PL180706095', 'TSH-PL180706096',
'TSH-PL180706104', 'TSH-PL180706113', 'TSH-PL180706118', 'TSH-PL180706145', 'TSH-PL180706147',
'TSH-PL180706150', 'TSH-PL180706153', 'TSH-PL180706156', 'TSH-PL181128001', 'TSH-PL181128003',
'TSH-PL181128005', 'TSH-PL181128007', 'TSH-PL181128008', 'TSH-PL181128009', 'TSH-PL181128011',
'TSH-PL181128012', 'TSH-PL181128014', 'TSH-PL181128015', 'TSH-PL181128019', 'TSH-PL181128021',
'TSH-PL181128022', 'TSH-PL181128023', 'TSH-PL181128025', 'TSH-PL181128027', 'TSH-PL181128029',
'TSH-PL181128030', 'TSH-PL181128033', 'TSH-PL181128035', 'TSH-PL181128036', 'TSH-PL181128038',
'TSH-PL181128039', 'TSH-PL181128040', 'TSH-PL181128041', 'TSH-PL181128042', 'TSH-PL181128043',
'TSH-PL181128045', 'TSH-PL181128047', 'TSH-PL181128048', 'TSH-PL181128049', 'TSH-PL181128050',
'TSH-PL181128051', 'TSH-PL181128054', 'TSH-PL181128055', 'TSH-PL181128056', 'TSH-PL181128057',
'TSH-PL181128058', 'TSH-PL181128061', 'TSH-PL181128062', 'TSH-PL181128064', 'TSH-PL181128065',
'TSH-PL181128066', 'TSH-PL181128070', 'TSH-PL181128073', 'TSH-PL181128077', 'TSH-PL181128080',
'TSH-PL181128082', 'TSH-PL181128083', 'TSH-PL181128085', 'TSH-PL181128086', 'TSH-PL181128093',
'TSH-PL181128096', 'TSH-PL181128099', 'TSH-PL181128101', 'TSH-PL181128102', 'TSH-PL181128103',
'TSH-PL181128104', 'TSH-PL181128107', 'TSH-PL181128109', 'TSH-PL181128110', 'TSH-PL181128111',
'TSH-PL181128112', 'TSH-PL181128114', 'TSH-PL181128115', 'TSH-PL181128117', 'TSH-PL181128118',
'TSH-PL181128120', 'TSH-PL181128122', 'TSH-PL181128123', 'TSH-PL181128124', 'TSH-PL181128125',
'TSH-PL181128126', 'TSH-PL181128127', 'TSH-PL181205002', 'TSH-PL181205003', 'TSH-PL181205005',
'TSH-PL181205006', 'TSH-PL181205008', 'TSH-PL181205009', 'TSH-PL181205013', 'TSH-PL181205014',
'TSH-PL181205016', 'TSH-PL181205020', 'TSH-PL181205022', 'TSH-PL181205024', 'TSH-PL181205026',
'TSH-PL181205027', 'TSH-PL181205028', 'TSH-PL181205030', 'TSH-PL181205033', 'TSH-PL181205035',
'TSH-PL181205040', 'TSH-PL181205041', 'TSH-PL181205042', 'TSH-PL181205043', 'TSH-PL181205044',
'TSH-PL181205045', 'TSH-PL181205046', 'TSH-PL181205047', 'TSH-PL181205048', 'TSH-PL181205049',
'TSH-PL181205051', 'TSH-PL181205053', 'TSH-PL181205054', 'TSH-PL181205055', 'TSH-PL181205057',
'TSH-PL181205058', 'TSH-PL181205059', 'TSH-PL181205060', 'TSH-PL181205061', 'TSH-PL181205062',
'TSH-PL181205063', 'TSH-PL181205064', 'TSH-PL181205065', 'TSH-PL181205066', 'TSH-PL181205068',
'TSH-PL181205069', 'TSH-PL181205072', 'TSH-PL181205074', 'TSH-PL181205075', 'TSH-PL181205076',
'TSH-PL181205077', 'TSH-PL181205078', 'TSH-PL181205079', 'TSH-PL181205081', 'TSH-PL181205082',
'TSH-PL181205083', 'TSH-PL181205085', 'TSH-PL181205086', 'TSH-PL181205087', 'TSH-PL181205088',
'TSH-PL181205090', 'TSH-PL181205091', 'TSH-PL181205093', 'TSH-PL181205095', 'TSH-PL181205096',
'TSH-PL181205097', 'TSH-PL181205098', 'TSH-PL181205099', 'TSH-PL181205102', 'TSH-PL181205103',
'TSH-PL181205104', 'TSH-PL181205107', 'TSH-PL181205108', 'TSH-PL181205110', 'TSH-PL181205113',
'TSH-PL181205115', 'TSH-PL181205116', 'TSH-PL181205117', 'TSH-PL181205121', 'TSH-PL181205122',
'TSH-PL181205125', 'TSH-PL181205128', 'TSH-PL181205129', 'TSH-PL181205140', 'TSH-PL181205141',
'TSH-PL181205142', 'TSH-PL181205143', 'TSH-PL181205144', 'TSH-PL181205145', 'TSH-PL181205146',
'TSH-PL181205147', 'TSH-PL181205148', 'TSH-PL181205149', 'TSH-PL181205150', 'TSH-PL181205151',
'TSH-PL181205152', 'TSH-PL181205155', 'TSH-PL181205156', 'TSH-PL181205157', 'TSH-PL181205158',
'TSH-PL181205160', 'TSH-PL181205161', 'TSH-PL181205162', 'TSH-PL181205164', 'TSH-PL181205165',
'TSH-PL181205166', 'TSH-PL180309082', 'TSH-PL180316013', 'TSH-PL180316029', 'TSH-PL180316075',
'TSH-PL180316102', 'TSH-PL180316112', 'TSH-PL180316134', 'TSH-PL180316138', 'TSH-PL180316175',
'TSH-PL180316178', 'TSH-PL180316200', 'TSH-PL180323029', 'TSH-PL180323032', 'TSH-PL180323053',
'TSH-PL180323068', 'TSH-PL180323074', 'TSH-PL180323088', 'TSH-PL180323097', 'TSH-PL180323103',
'TSH-PL180323115', 'TSH-PL180323120', 'TSH-PL180323160', 'TSH-PL180323175', 'TSH-PL180410058',
'TSH-PL180410070', 'TSH-PL180410081', 'TSH-PL180410148', 'TSH-PL180410161', 'TSH-PL180417017',
'TSH-PL180417023', 'TSH-PL180417045', 'TSH-PL180417077', 'TSH-PL180417104', 'TSH-PL180417111',
'TSH-PL180417124', 'TSH-PL180417162', 'TSH-PL180417169', 'TSH-PL180417199', 'TSH-PL180424042',
'TSH-PL180424088', 'TSH-PL180424138', 'TSH-PL180703012', 'TSH-PL180703127', 'TSH-PL180703134',
'TSH-PL180703141', 'TSH-PL180703168', 'TSH-PL180706117', 'TSH-PL181128004', 'TSH-PL181128006',
'TSH-PL181128013', 'TSH-PL181128018', 'TSH-PL181128020', 'TSH-PL181128031', 'TSH-PL181128037',
'TSH-PL181128067', 'TSH-PL181128071', 'TSH-PL181128078', 'TSH-PL181128084', 'TSH-PL181128095',
'TSH-PL181128097', 'TSH-PL181128098', 'TSH-PL181128105', 'TSH-PL181128106', 'TSH-PL181128108',
'TSH-PL181128113', 'TSH-PL181128116', 'TSH-PL181205001', 'TSH-PL181205004', 'TSH-PL181205007',
'TSH-PL181205010', 'TSH-PL181205011', 'TSH-PL181205012', 'TSH-PL181205015', 'TSH-PL181205018',
'TSH-PL181205019', 'TSH-PL181205023', 'TSH-PL181205031', 'TSH-PL181205032', 'TSH-PL181205036',
'TSH-PL181205037', 'TSH-PL181205052', 'TSH-PL181205056', 'TSH-PL181205067', 'TSH-PL181205070',
'TSH-PL181205071', 'TSH-PL181205073', 'TSH-PL181205080', 'TSH-PL181205084', 'TSH-PL181205089',
'TSH-PL181205092', 'TSH-PL181205094', 'TSH-PL181205100', 'TSH-PL181205101', 'TSH-PL181205106',
'TSH-PL181205112', 'TSH-PL181205118', 'TSH-PL181205119', 'TSH-PL181205120', 'TSH-PL181205123',
'TSH-PL181205124', 'TSH-PL181205153', 'TSH-PL181205154', 'TSH-PL181205159', 'TSH-PL181205163']
```

In [6]:
```python
healthy_samples = ['TSH-PL180309087', 'TSH-PL180316003', 'TSH-PL180316005', 'TSH-PL180316016',
'TSH-PL180316017', 'TSH-PL180316018', 'TSH-PL180316022', 'TSH-PL180316024', 'TSH-PL180316027',
'TSH-PL180316033', 'TSH-PL180316037', 'TSH-PL180316038', 'TSH-PL180316039', 'TSH-PL180316049',
'TSH-PL180316061', 'TSH-PL180316065', 'TSH-PL180316079', 'TSH-PL180316092', 'TSH-PL180316095',
'TSH-PL180316099', 'TSH-PL180316101', 'TSH-PL180316106', 'TSH-PL180316115', 'TSH-PL180316119',
'TSH-PL180316126', 'TSH-PL180316129', 'TSH-PL180316157', 'TSH-PL180316161', 'TSH-PL180316163',
'TSH-PL180316164', 'TSH-PL180316173', 'TSH-PL180316174', 'TSH-PL180316181', 'TSH-PL180316183',
'TSH-PL180316187', 'TSH-PL180316190', 'TSH-PL180316195', 'TSH-PL180316196', 'TSH-PL180316198',
'TSH-PL180323034', 'TSH-PL180323038', 'TSH-PL180323051', 'TSH-PL180323052', 'TSH-PL180323054',
'TSH-PL180323059', 'TSH-PL180323065', 'TSH-PL180323067', 'TSH-PL180323072', 'TSH-PL180323073',
'TSH-PL180323091', 'TSH-PL180323106', 'TSH-PL180323107', 'TSH-PL180323109', 'TSH-PL180323110',
'TSH-PL180323111', 'TSH-PL180323119', 'TSH-PL180323121', 'TSH-PL180323124', 'TSH-PL180323139',
'TSH-PL180323141', 'TSH-PL180323142', 'TSH-PL180323151', 'TSH-PL180323155', 'TSH-PL180323166',
'TSH-PL180410003', 'TSH-PL180410023', 'TSH-PL180410043', 'TSH-PL180410049', 'TSH-PL180410059',
'TSH-PL180410063', 'TSH-PL180410076', 'TSH-PL180410087', 'TSH-PL180410115', 'TSH-PL180410116',
'TSH-PL180410133', 'TSH-PL180410140', 'TSH-PL180410144', 'TSH-PL180410160', 'TSH-PL180410162',
'TSH-PL180410198', 'TSH-PL180417003', 'TSH-PL180417007', 'TSH-PL180417035', 'TSH-PL180417060',
'TSH-PL180417074', 'TSH-PL180417087', 'TSH-PL180417095', 'TSH-PL180417101', 'TSH-PL180417109',
'TSH-PL180417117', 'TSH-PL180417135', 'TSH-PL180417138', 'TSH-PL180417143', 'TSH-PL180417152',
'TSH-PL180417156', 'TSH-PL180417165', 'TSH-PL180417170', 'TSH-PL180417179', 'TSH-PL180417180',
'TSH-PL180417181', 'TSH-PL180417200', 'TSH-PL180424003', 'TSH-PL180424030', 'TSH-PL180424036',
'TSH-PL180424058', 'TSH-PL180424090', 'TSH-PL180424097', 'TSH-PL180424104', 'TSH-PL180424115',
'TSH-PL180424146', 'TSH-PL180424149', 'TSH-PL180424155', 'TSH-PL180424162', 'TSH-PL180424166',
'TSH-PL180424171', 'TSH-PL180703037', 'TSH-PL180703136', 'TSH-PL180703153', 'TSH-PL180703157',
'TSH-PL180703185', 'TSH-PL180706034', 'TSH-PL180706037', 'TSH-PL180706095', 'TSH-PL180706096',
'TSH-PL180706104', 'TSH-PL180706113', 'TSH-PL180706118', 'TSH-PL180706145', 'TSH-PL180706147',
'TSH-PL180706150', 'TSH-PL180706153', 'TSH-PL180706156', 'TSH-PL181128001', 'TSH-PL181128003',
'TSH-PL181128005', 'TSH-PL181128007', 'TSH-PL181128008', 'TSH-PL181128009', 'TSH-PL181128011',
'TSH-PL181128012', 'TSH-PL181128014', 'TSH-PL181128015', 'TSH-PL181128019', 'TSH-PL181128021',
'TSH-PL181128022', 'TSH-PL181128023', 'TSH-PL181128025', 'TSH-PL181128027', 'TSH-PL181128029',
'TSH-PL181128030', 'TSH-PL181128033', 'TSH-PL181128035', 'TSH-PL181128036', 'TSH-PL181128038',
'TSH-PL181128039', 'TSH-PL181128040', 'TSH-PL181128041', 'TSH-PL181128042', 'TSH-PL181128043',
'TSH-PL181128045', 'TSH-PL181128047', 'TSH-PL181128048', 'TSH-PL181128049', 'TSH-PL181128050',
'TSH-PL181128051', 'TSH-PL181128054', 'TSH-PL181128055', 'TSH-PL181128056', 'TSH-PL181128057',
'TSH-PL181128058', 'TSH-PL181128061', 'TSH-PL181128062', 'TSH-PL181128064', 'TSH-PL181128065',
'TSH-PL181128066', 'TSH-PL181128070', 'TSH-PL181128073', 'TSH-PL181128077', 'TSH-PL181128080',
'TSH-PL181128082', 'TSH-PL181128083', 'TSH-PL181128085', 'TSH-PL181128086', 'TSH-PL181128093',
'TSH-PL181128096', 'TSH-PL181128099', 'TSH-PL181128101', 'TSH-PL181128102', 'TSH-PL181128103',
'TSH-PL181128104', 'TSH-PL181128107', 'TSH-PL181128109', 'TSH-PL181128110', 'TSH-PL181128111',
'TSH-PL181128112', 'TSH-PL181128114', 'TSH-PL181128115', 'TSH-PL181128117', 'TSH-PL181128118',
'TSH-PL181128120', 'TSH-PL181128122', 'TSH-PL181128123', 'TSH-PL181128124', 'TSH-PL181128125',
'TSH-PL181128126', 'TSH-PL181128127', 'TSH-PL181205002', 'TSH-PL181205003', 'TSH-PL181205005',
'TSH-PL181205006', 'TSH-PL181205008', 'TSH-PL181205009', 'TSH-PL181205013', 'TSH-PL181205014',
'TSH-PL181205016', 'TSH-PL181205020', 'TSH-PL181205022', 'TSH-PL181205024', 'TSH-PL181205026',
'TSH-PL181205027', 'TSH-PL181205028', 'TSH-PL181205030', 'TSH-PL181205033', 'TSH-PL181205035',
'TSH-PL181205040', 'TSH-PL181205041', 'TSH-PL181205042', 'TSH-PL181205043', 'TSH-PL181205044',
'TSH-PL181205045', 'TSH-PL181205046', 'TSH-PL181205047', 'TSH-PL181205048', 'TSH-PL181205049',
'TSH-PL181205051', 'TSH-PL181205053', 'TSH-PL181205054', 'TSH-PL181205055', 'TSH-PL181205057',
'TSH-PL181205058', 'TSH-PL181205059', 'TSH-PL181205060', 'TSH-PL181205061', 'TSH-PL181205062',
'TSH-PL181205063', 'TSH-PL181205064', 'TSH-PL181205065', 'TSH-PL181205066', 'TSH-PL181205068',
'TSH-PL181205069', 'TSH-PL181205072', 'TSH-PL181205074', 'TSH-PL181205075', 'TSH-PL181205076',
'TSH-PL181205077', 'TSH-PL181205078', 'TSH-PL181205079', 'TSH-PL181205081', 'TSH-PL181205082',
'TSH-PL181205083', 'TSH-PL181205085', 'TSH-PL181205086', 'TSH-PL181205087', 'TSH-PL181205088',
'TSH-PL181205090', 'TSH-PL181205091', 'TSH-PL181205093', 'TSH-PL181205095', 'TSH-PL181205096',
'TSH-PL181205097', 'TSH-PL181205098', 'TSH-PL181205099', 'TSH-PL181205102', 'TSH-PL181205103',
'TSH-PL181205104', 'TSH-PL181205107', 'TSH-PL181205108', 'TSH-PL181205110', 'TSH-PL181205113',
'TSH-PL181205115', 'TSH-PL181205116', 'TSH-PL181205117', 'TSH-PL181205121', 'TSH-PL181205122',
'TSH-PL181205125', 'TSH-PL181205128', 'TSH-PL181205129', 'TSH-PL181205140', 'TSH-PL181205141',
'TSH-PL181205142', 'TSH-PL181205143', 'TSH-PL181205144', 'TSH-PL181205145', 'TSH-PL181205146',
'TSH-PL181205147', 'TSH-PL181205148', 'TSH-PL181205149', 'TSH-PL181205150', 'TSH-PL181205151',
'TSH-PL181205152', 'TSH-PL181205155', 'TSH-PL181205156', 'TSH-PL181205157', 'TSH-PL181205158',
'TSH-PL181205160', 'TSH-PL181205161', 'TSH-PL181205162', 'TSH-PL181205164', 'TSH-PL181205165',
'TSH-PL181205166', 'TSH-PL180309082', 'TSH-PL180316013', 'TSH-PL180316029', 'TSH-PL180316075',
'TSH-PL180316102', 'TSH-PL180316112', 'TSH-PL180316134', 'TSH-PL180316138', 'TSH-PL180316175',
'TSH-PL180316178', 'TSH-PL180316200', 'TSH-PL180323029', 'TSH-PL180323032', 'TSH-PL180323053',
'TSH-PL180323068', 'TSH-PL180323074', 'TSH-PL180323088', 'TSH-PL180323097', 'TSH-PL180323103',
'TSH-PL180323115', 'TSH-PL180323120', 'TSH-PL180323160', 'TSH-PL180323175', 'TSH-PL180410058',
'TSH-PL180410070', 'TSH-PL180410081', 'TSH-PL180410148', 'TSH-PL180410161', 'TSH-PL180417017',
'TSH-PL180417023', 'TSH-PL180417045', 'TSH-PL180417077', 'TSH-PL180417104', 'TSH-PL180417111',
'TSH-PL180417124', 'TSH-PL180417162', 'TSH-PL180417169', 'TSH-PL180417199', 'TSH-PL180424042',
'TSH-PL180424088', 'TSH-PL180424138', 'TSH-PL180703012', 'TSH-PL180703127', 'TSH-PL180703134',
'TSH-PL180703141', 'TSH-PL180703168', 'TSH-PL180706117', 'TSH-PL181128004', 'TSH-PL181128006',
'TSH-PL181128013', 'TSH-PL181128018', 'TSH-PL181128020', 'TSH-PL181128031', 'TSH-PL181128037',
'TSH-PL181128067', 'TSH-PL181128071', 'TSH-PL181128078', 'TSH-PL181128084', 'TSH-PL181128095',
'TSH-PL181128097', 'TSH-PL181128098', 'TSH-PL181128105', 'TSH-PL181128106', 'TSH-PL181128108',
'TSH-PL181128113', 'TSH-PL181128116', 'TSH-PL181205001', 'TSH-PL181205004', 'TSH-PL181205007',
'TSH-PL181205010', 'TSH-PL181205011', 'TSH-PL181205012', 'TSH-PL181205015', 'TSH-PL181205018',
'TSH-PL181205019', 'TSH-PL181205023', 'TSH-PL181205031', 'TSH-PL181205032', 'TSH-PL181205036',
'TSH-PL181205037', 'TSH-PL181205052', 'TSH-PL181205056', 'TSH-PL181205067', 'TSH-PL181205070',
'TSH-PL181205071', 'TSH-PL181205073', 'TSH-PL181205080', 'TSH-PL181205084', 'TSH-PL181205089',
'TSH-PL181205092', 'TSH-PL181205094', 'TSH-PL181205100', 'TSH-PL181205101', 'TSH-PL181205106',
'TSH-PL181205112', 'TSH-PL181205118', 'TSH-PL181205119', 'TSH-PL181205120', 'TSH-PL181205123',
'TSH-PL181205124', 'TSH-PL181205153', 'TSH-PL181205154', 'TSH-PL181205159', 'TSH-PL181205163']

post_diagnosis_samples_colon = ['TSH-PL180510139', 'TSH-PL180510140', 'TSH-PL180510146', 'TSH-PL180510147', 'TSH-PL180510162', 'TSH-PL181205175', 'TSH-PL180510143']
post_diagnosis_samples_liver = ['TSH-PL180510085', 'TSH-PL180510089', 'TSH-PL180510092', 'TSH-PL180510095', 'TSH-PL180510101', 'TSH-PL180510104', 'TSH-PL180510105', 'TSH-PL180510107', 'TSH-PL180510112', 'TSH-PL180510121', 'TSH-PL180510122', 'TSH-PL180510125', 'TSH-PL181205173', 'TSH-PL180510088', 'TSH-PL180510090', 'TSH-PL180510098', 'TSH-PL180510100', 'TSH-PL180510103', 'TSH-PL180510108', 'TSH-PL180510109', 'TSH-PL180510113', 'TSH-PL180510117', 'TSH-PL180510124']
post_diagnosis_samples_esophagus = ['TSH-PL171221101', 'TSH-PL171221112', 'TSH-PL171221149', 'TSH-PL171221150', 'TSH-PL171221160', 'TSH-PL171221172', 'TSH-PL171221179', 'TSH-PL171221180',
'TSH-PL180508001', 'TSH-PL180508004', 'TSH-PL180508005', 'TSH-PL180508006', 'TSH-PL180508008',
'TSH-PL180508009', 'TSH-PL180508011', 'TSH-PL180508012', 'TSH-PL180508013', 'TSH-PL180508014',
'TSH-PL180508015', 'TSH-PL180508019', 'TSH-PL180508020', 'TSH-PL180508021', 'TSH-PL180508022',
'TSH-PL180508025', 'TSH-PL180508030', 'TSH-PL180508031', 'TSH-PL180508033', 'TSH-PL180508034',
'TSH-PL180508036', 'TSH-PL180508037', 'TSH-PL180508038', 'TSH-PL180508039', 'TSH-PL180508040',
'TSH-PL180508041', 'TSH-PL180508042', 'TSH-PL180508043', 'TSH-PL180508049', 'TSH-PL180508050',
'TSH-PL180508060', 'TSH-PL180508061', 'TSH-PL180508064', 'TSH-PL180508068', 'TSH-PL180508076',
'TSH-PL180508078', 'TSH-PL180508083', 'TSH-PL180508086', 'TSH-PL180508089', 'TSH-PL180510044',
'TSH-PL180510061', 'TSH-PL181205168', 'TSH-PL181205170', 'TSH-PL171221151', 'TSH-PL180508003',
'TSH-PL180508007', 'TSH-PL180508023', 'TSH-PL180508026', 'TSH-PL180508028', 'TSH-PL180508029',
'TSH-PL180508032', 'TSH-PL180508044', 'TSH-PL180508052', 'TSH-PL180508065', 'TSH-PL180508067',
'TSH-PL180508088', 'TSH-PL181205172', 'TSH-PL181205174', 'TSH-PL171221153', 'TSH-PL171221113']
post_diagnosis_samples_lung = ['TSH-PL180510005', 'TSH-PL180510006', 'TSH-PL180510007', 'TSH-PL180510008', 'TSH-PL180510010', 'TSH-PL180510011', 'TSH-PL180510012', 'TSH-PL180510014', 'TSH-PL180510015', 'TSH-PL180510016', 'TSH-PL180510017', 'TSH-PL180510018', 'TSH-PL180510019', 'TSH-PL180510021', 'TSH-PL180510023', 'TSH-PL180510024', 'TSH-PL180510025', 'TSH-PL180510026', 'TSH-PL180510030', 'TSH-PL180510031', 'TSH-PL180510033', 'TSH-PL180510038', 'TSH-PL180510040', 'TSH-PL180510047', 'TSH-PL180510050', 'TSH-PL180510052', 'TSH-PL180510053', 'TSH-PL180510054', 'TSH-PL180510055', 'TSH-PL180510057', 'TSH-PL180510063', 'TSH-PL180510066', 'TSH-PL180510071',
'TSH-PL180510072', 'TSH-PL180510073', 'TSH-PL180510074', 'TSH-PL180510075', 'TSH-PL180510076',
'TSH-PL180510077', 'TSH-PL180510078', 'TSH-PL180510079', 'TSH-PL180510080', 'TSH-PL181205182',
'TSH-PL180510002', 'TSH-PL180510004', 'TSH-PL180510013', 'TSH-PL180510020', 'TSH-PL180510022',
'TSH-PL180510032', 'TSH-PL180510034', 'TSH-PL180510035', 'TSH-PL180510043', 'TSH-PL180510048',
'TSH-PL180510068', 'TSH-PL181205177', 'TSH-PL181205179']
post_diagnosis_samples_stomach = ['TSH-PL180504001', 'TSH-PL180504002', 'TSH-PL180504003', 'TSH-PL180504004', 'TSH-PL180504005', 'TSH-PL180504009', 'TSH-PL180504010', 'TSH-PL180504011', 'TSH-PL180504013', 'TSH-PL180504014', 'TSH-PL180504015', 'TSH-PL180504016', 'TSH-PL180504017', 'TSH-PL180504018', 'TSH-PL180504019', 'TSH-PL180504021', 'TSH-PL180504022', 'TSH-PL180504027',
'TSH-PL180504028', 'TSH-PL180504030', 'TSH-PL180504033', 'TSH-PL180504034', 'TSH-PL180504041',
'TSH-PL180504042', 'TSH-PL180504044', 'TSH-PL180504046', 'TSH-PL180504047', 'TSH-PL180504049',
'TSH-PL180504050', 'TSH-PL180504053', 'TSH-PL180504054', 'TSH-PL180504058', 'TSH-PL180504061',
'TSH-PL180504062', 'TSH-PL180504063', 'TSH-PL180504065', 'TSH-PL180504068', 'TSH-PL180504070',
'TSH-PL180504074', 'TSH-PL180504081', 'TSH-PL180504083', 'TSH-PL180504087', 'TSH-PL180504088',
'TSH-PL180504089', 'TSH-PL180504091', 'TSH-PL180504094', 'TSH-PL180504097', 'TSH-PL180504101',
'TSH-PL180504104', 'TSH-PL180504108', 'TSH-PL180504111', 'TSH-PL180504118', 'TSH-PL180504006',
'TSH-PL180504012', 'TSH-PL180504024', 'TSH-PL180504026', 'TSH-PL180504029', 'TSH-PL180504037',
'TSH-PL180504043', 'TSH-PL180504045', 'TSH-PL180504051', 'TSH-PL180504052', 'TSH-PL180504055',
'TSH-PL180504057', 'TSH-PL180504064', 'TSH-PL180504076', 'TSH-PL180504084', 'TSH-PL180504110',
'TSH-PL181205169']

pre_diagnosis_samples_colon = ['TSH-PL180123001', 'TSH-PL180123010', 'TSH-PL180513013', 'TSH-PL180513014', 'TSH-PL180513020', 'TSH-PL180513027', 'TSH-PL180513029', 'TSH-PL180513030', 'TSH-PL180513034', 'TSH-PL180513037', 'TSH-PL180513049', 'TSH-PL180513062', 'TSH-PL180513077', 'TSH-PL180513078', 'TSH-PL180513085', 'TSH-PL180513114', 'TSH-PL180516003', 'TSH-PL180516011', 'TSH-PL180516012', 'TSH-PL180516014', 'TSH-PL180516018', 'TSH-PL180516021', 'TSH-PL180516028', 'TSH-PL180516039', 'TSH-PL180516044', 'TSH-PL180516053', 'TSH-PL180516057', 'TSH-PL180513001', 'TSH-PL180513002', 'TSH-PL180513064', 'TSH-PL180513116', 'TSH-PL180516015', 'TSH-PL180516017',
'TSH-PL180516026', 'TSH-PL180516033']
pre_diagnosis_samples_liver = ['TSH-PL180123014', 'TSH-PL180123031', 'TSH-PL180513006', 'TSH-PL180513011', 'TSH-PL180513017', 'TSH-PL180513021', 'TSH-PL180513026', 'TSH-PL180513041', 'TSH-PL180513043', 'TSH-PL180513071', 'TSH-PL180513088', 'TSH-PL180513095', 'TSH-PL180513098', 'TSH-PL180513108', 'TSH-PL180513109', 'TSH-PL180513111', 'TSH-PL180513117', 'TSH-PL180516006', 'TSH-PL180516038', 'TSH-PL180516040', 'TSH-PL180516051', 'TSH-PL180516054', 'TSH-PL180516063', 'TSH-PL180123004', 'TSH-PL180123005', 'TSH-PL180513009', 'TSH-PL180513033', 'TSH-PL180513090',
'TSH-PL180516025']
pre_diagnosis_samples_esophagus = ['TSH-PL180123006', 'TSH-PL180123007', 'TSH-PL180123008', 'TSH-PL180123016', 'TSH-PL180123025', 'TSH-PL180513004', 'TSH-PL180513005', 'TSH-PL180513016', 'TSH-PL180513051', 'TSH-PL180513068', 'TSH-PL180513072', 'TSH-PL180513075', 'TSH-PL180513080',
'TSH-PL180513081', 'TSH-PL180513091', 'TSH-PL180513093', 'TSH-PL180513097', 'TSH-PL180513100',
'TSH-PL180513101', 'TSH-PL180513110', 'TSH-PL180513115', 'TSH-PL180513118', 'TSH-PL180513121',
'TSH-PL180513122', 'TSH-PL180513124', 'TSH-PL180513128', 'TSH-PL180513129', 'TSH-PL180516013',
'TSH-PL180516020', 'TSH-PL180516030', 'TSH-PL180516031', 'TSH-PL180516045', 'TSH-PL180516049',
'TSH-PL180516059', 'TSH-PL180123024', 'TSH-PL180123032', 'TSH-PL180513028', 'TSH-PL180513032',
'TSH-PL180513058', 'TSH-PL180513083', 'TSH-PL180513096', 'TSH-PL180516005', 'TSH-PL180516047',
'TSH-PL180516050', 'TSH-PL180516061']
pre_diagnosis_samples_lung = ['TSH-PL180123013', 'TSH-PL180123021', 'TSH-PL180123022', 'TSH-PL180123029', 'TSH-PL180123030', 'TSH-PL180513007', 'TSH-PL180513012', 'TSH-PL180513023', 'TSH-PL180513024', 'TSH-PL180513036', 'TSH-PL180513040', 'TSH-PL180513045', 'TSH-PL180513055', 'TSH-PL180513056', 'TSH-PL180513057', 'TSH-PL180513067', 'TSH-PL180513069', 'TSH-PL180513079', 'TSH-PL180513084', 'TSH-PL180513087', 'TSH-PL180513092', 'TSH-PL180513106', 'TSH-PL180516001', 'TSH-PL180516002', 'TSH-PL180516004', 'TSH-PL180516016', 'TSH-PL180516023', 'TSH-PL180516032', 'TSH-PL180516055', 'TSH-PL180516056', 'TSH-PL180516062', 'TSH-PL180123002', 'TSH-PL180123012', 'TSH-PL180513010', 'TSH-PL180513019', 'TSH-PL180513022', 'TSH-PL180513031', 'TSH-PL180513035',
'TSH-PL180513044', 'TSH-PL180513086', 'TSH-PL180513112', 'TSH-PL180516029', 'TSH-PL180516034',
'TSH-PL180516046', 'TSH-PL180516048', 'TSH-PL180516058', 'TSH-PL180516060']
pre_diagnosis_samples_stomach = ['TSH-PL180123017', 'TSH-PL180123018', 'TSH-PL180123027', 'TSH-PL180123033', 'TSH-PL180513025', 'TSH-PL180513038', 'TSH-PL180513039', 'TSH-PL180513050', 'TSH-PL180513060', 'TSH-PL180513066', 'TSH-PL180513070', 'TSH-PL180513073', 'TSH-PL180513076', 'TSH-PL180513089', 'TSH-PL180513099', 'TSH-PL180513102', 'TSH-PL180513104', 'TSH-PL180513119', 'TSH-PL180513123', 'TSH-PL180513127', 'TSH-PL180516007', 'TSH-PL180516008', 'TSH-PL180516009',
'TSH-PL180516019', 'TSH-PL180516027', 'TSH-PL180516036', 'TSH-PL180516042', 'TSH-PL180516043',
'TSH-PL180513003', 'TSH-PL180513018', 'TSH-PL180513103', 'TSH-PL180513105', 'TSH-PL180513125',
'TSH-PL180516010', 'TSH-PL180516052']
```

BioChain Tissue Samples

In [7]:
```python
healthy_lung_tissue = ['BC_Lung_ND1', 'BC_Lung_ND2', 'BC_Lung_ND3', 'BC_Lung_ND4', 'BC_Lung_ND5', 'BC_Lung_ND6', 'BC_Lung_ND7', 'BC_Lung_ND8']
healthy_colon_tissue = ['BC_CRC_ND1', 'BC_CRC_ND2', 'BC_CRC_ND3', 'BC_CRC_ND4', 'BC_CRC_ND5', 'BC_CRC_ND6', 'BC_CRC_ND7', 'BC_CRC_ND8']
healthy_stomach_tissue = ['BC_Stomach_ND1', 'BC_Stomach_ND2', 'BC_Stomach_ND3', 'BC_Stomach_ND4', 'BC_Stomach_ND5', 'BC_Stomach_ND6', 'BC_Stomach_ND7', 'BC_Stomach_ND8']
healthy_breast_tissue = ['BC_Breast_ND1', 'BC_Breast_ND2', 'BC_Breast_ND3', 'BC_Breast_ND4', 'BC_Breast_ND5', 'BC_Breast_ND6', 'BC_Breast_ND7', 'BC_Breast_ND8']

cancer_lung_tissue = ['BC_Lung_TD1', 'BC_Lung_TD10', 'BC_Lung_TD11', 'BC_Lung_TD12', 'BC_Lung_TD13', 'BC_Lung_TD14', 'BC_Lung_TD15', 'BC_Lung_TD16', 'BC_Lung_TD17', 'BC_Lung_TD18', 'BC_Lung_TD19', 'BC_Lung_TD2', 'BC_Lung_TD20', 'BC_Lung_TD21', 'BC_Lung_TD22', 'BC_Lung_TD23', 'BC_Lung_TD24', 'BC_Lung_TD25', 'BC_Lung_TD26', 'BC_Lung_TD27', 'BC_Lung_TD28', 'BC_Lung_TD29', 'BC_Lung_TD3', 'BC_Lung_TD30', 'BC_Lung_TD31', 'BC_Lung_TD32', 'BC_Lung_TD33', 'BC_Lung_TD34', 'BC_Lung_TD35', 'BC_Lung_TD36', 'BC_Lung_TD37', 'BC_Lung_TD38', 'BC_Lung_TD39', 'BC_Lung_TD4', 'BC_Lung_TD40', 'BC_Lung_TD5', 'BC_Lung_TD6', 'BC_Lung_TD7', 'BC_Lung_TD8', 'BC_Lung_TD9']
cancer_colon_tissue = ['BC_CRC_TD1', 'BC_CRC_TD10', 'BC_CRC_TD11', 'BC_CRC_TD12', 'BC_CRC_TD13', 'BC_CRC_TD14', 'BC_CRC_TD15', 'BC_CRC_TD16', 'BC_CRC_TD17', 'BC_CRC_TD18', 'BC_CRC_TD19', 'BC_CRC_TD2', 'BC_CRC_TD20', 'BC_CRC_TD21', 'BC_CRC_TD22', 'BC_CRC_TD23', 'BC_CRC_TD24', 'BC_CRC_TD25', 'BC_CRC_TD26', 'BC_CRC_TD27', 'BC_CRC_TD28', 'BC_CRC_TD29', 'BC_CRC_TD3', 'BC_CRC_TD30', 'BC_CRC_TD31', 'BC_CRC_TD32', 'BC_CRC_TD33', 'BC_CRC_TD34', 'BC_CRC_TD35', 'BC_CRC_TD36', 'BC_CRC_TD37', 'BC_CRC_TD38', 'BC_CRC_TD39', 'BC_CRC_TD4', 'BC_CRC_TD40', 'BC_CRC_TD5', 'BC_CRC_TD6', 'BC_CRC_TD7', 'BC_CRC_TD8', 'BC_CRC_TD9']
cancer_stomach_tissue = ['BC_Stomach_TD1', 'BC_Stomach_TD10', 'BC_Stomach_TD11', 'BC_Stomach_TD12', 'BC_Stomach_TD13', 'BC_Stomach_TD14', 'BC_Stomach_TD15', 'BC_Stomach_TD16', 'BC_Stomach_TD17', 'BC_Stomach_TD18', 'BC_Stomach_TD19', 'BC_Stomach_TD2', 'BC_Stomach_TD20', 'BC_Stomach_TD21', 'BC_Stomach_TD22', 'BC_Stomach_TD23', 'BC_Stomach_TD24', 'BC_Stomach_TD25', 'BC_Stomach_TD26', 'BC_Stomach_TD27', 'BC_Stomach_TD28', 'BC_Stomach_TD29', 'BC_Stomach_TD3', 'BC_Stomach_TD30', 'BC_Stomach_TD31', 'BC_Stomach_TD32', 'BC_Stomach_TD33', 'BC_Stomach_TD34', 'BC_Stomach_TD35', 'BC_Stomach_TD36', 'BC_Stomach_TD37', 'BC_Stomach_TD38', 'BC_Stomach_TD39', 'BC_Stomach_TD4', 'BC_Stomach_TD40', 'BC_Stomach_TD5', 'BC_Stomach_TD6', 'BC_Stomach_TD7', 'BC_Stomach_TD8', 'BC_Stomach_TD9']
cancer_breast_tissue = ['BC_Breast_TD1', 'BC_Breast_TD10', 'BC_Breast_TD11', 'BC_Breast_TD12', 'BC_Breast_TD13', 'BC_Breast_TD14', 'BC_Breast_TD15', 'BC_Breast_TD16', 'BC_Breast_TD17', 'BC_Breast_TD18', 'BC_Breast_TD19', 'BC_Breast_TD2', 'BC_Breast_TD20', 'BC_Breast_TD21', 'BC_Breast_TD22', 'BC_Breast_TD23', 'BC_Breast_TD24', 'BC_Breast_TD25', 'BC_Breast_TD26', 'BC_Breast_TD27', 'BC_Breast_TD28', 'BC_Breast_TD29', 'BC_Breast_TD3', 'BC_Breast_TD30', 'BC_Breast_TD31', 'BC_Breast_TD32', 'BC_Breast_TD33', 'BC_Breast_TD34', 'BC_Breast_TD35', 'BC_Breast_TD36', 'BC_Breast_TD37', 'BC_Breast_TD38', 'BC_Breast_TD39', 'BC_Breast_TD4', 'BC_Breast_TD40', 'BC_Breast_TD5', 'BC_Breast_TD6', 'BC_Breast_TD7', 'BC_Breast_TD8', 'BC_Breast_TD9']

all_tissue_samples = healthy_lung_tissue+healthy_colon_tissue+healthy_stomach_tissue+healthy_breast_tissue+cancer_lung_tissue+cancer_colon_tissue+cancer_stomach_tissue+cancer_breast_tissue
```

Load Data

Let's load a data matrix listing the average methylation across each target region for each sample.

In [8]:
```python
amf_df_orig = pd.read_csv('SupplementaryData5.tsv', sep="\t", header=0, index_col=0)
```

Marker Selection

Let's first remove markers that are not covered in all plasma samples, so we don't have to deal with any missing values.

In [9]:
```python
amf_df = amf_df_orig.copy()
amf_df = amf_df.loc[amf_df[healthy_samples+post_diagnosis_samples+pre_diagnosis_samples].dropna(how='any').index]
```

Next, let's choose markers that separate healthy tissue from cancer tissue.

For each tissue type we purchased from BioChain, we will use a T-test to identify target regions that separate normal tissue from cancer tissue (applying a Benjamini-Hochberg multiple testing correction).

In [10]:
```python
tissue_t_tests = pd.concat(
    [
        pd.Series(amf_df.index),
        pd.Series(scipy.stats.ttest_ind(amf_df.loc[:, healthy_colon_tissue].T,
                                        amf_df.loc[:, cancer_colon_tissue].T,
                                        axis=0, equal_var=True, nan_policy='omit').pvalue),
        pd.Series(scipy.stats.ttest_ind(amf_df.loc[:, healthy_lung_tissue].T,
                                        amf_df.loc[:, cancer_lung_tissue].T,
                                        axis=0, equal_var=True, nan_policy='omit').pvalue),
        pd.Series(scipy.stats.ttest_ind(amf_df.loc[:, healthy_stomach_tissue].T,
                                        amf_df.loc[:, cancer_stomach_tissue].T,
                                        axis=0, equal_var=True, nan_policy='omit').pvalue),
        pd.Series(scipy.stats.ttest_ind(amf_df.loc[:, healthy_breast_tissue].T,
                                        amf_df.loc[:, cancer_breast_tissue].T,
                                        axis=0, equal_var=True, nan_policy='omit').pvalue),
    ], axis=1
)

tissue_t_tests.columns = ['Locus',
                          'ColonM', 'LungM', 'StomachM', 'BreastM',
                         ]
tissue_t_tests = tissue_t_tests.set_index('Locus')

# Benjamini-Hochberg Correction
from statsmodels.stats.multitest import multipletests
_, pvals_corrected, _, _ = multipletests(tissue_t_tests.min(axis=1).fillna(1).values, alpha=0.05, method='fdr_bh')

tissue_t_tests_min_by_marker = tissue_t_tests.loc[pvals_corrected <= 0.05].iloc[:, :4].min(axis=1).copy()
```

In [11]:
```python
print len(tissue_t_tests_min_by_marker)
```

```
477
```

We can then use these target regions in our plasma samples to build a classifier.

Divide Samples Into Leave-In and Leave-Out

Let's first split the Taizhou samples into two sets: leave-in (1/2) and leave-out (1/2). The leave-in set will be used to build a model.

We want to make sure that equal proportions of healthy/cancer samples are included in the leave-in/leave-out set. We also want balance within each type of cancer (so we don't end up with no samples for one cancer type in the leave-out set, for example).

In [12]:
```python
random.seed(128)

# Healthy
random.shuffle(healthy_samples)
# Post-Diagnosis
random.shuffle(post_diagnosis_samples_colon)
random.shuffle(post_diagnosis_samples_liver)
random.shuffle(post_diagnosis_samples_esophagus)
random.shuffle(post_diagnosis_samples_lung)
random.shuffle(post_diagnosis_samples_stomach)
# Pre-Diagnosis
random.shuffle(pre_diagnosis_samples_colon)
random.shuffle(pre_diagnosis_samples_liver)
random.shuffle(pre_diagnosis_samples_esophagus)
random.shuffle(pre_diagnosis_samples_lung)
random.shuffle(pre_diagnosis_samples_stomach)

negative_leavein = list(healthy_samples[:int(len(healthy_samples)/2)])
negative_leaveout = list(healthy_samples[int(len(healthy_samples)/2):])

positive_post_leavein = post_diagnosis_samples_colon[:int(len(post_diagnosis_samples_colon)/2)] + post_diagnosis_samples_liver[:int(len(post_diagnosis_samples_liver)/2)] + post_diagnosis_samples_esophagus[:int(len(post_diagnosis_samples_esophagus)/2)] + post_diagnosis_samples_lung[:int(len(post_diagnosis_samples_lung)/2)] + post_diagnosis_samples_stomach[:int(len(post_diagnosis_samples_stomach)/2)]
positive_post_leaveout = post_diagnosis_samples_colon[int(len(post_diagnosis_samples_colon)/2):] + post_diagnosis_samples_liver[int(len(post_diagnosis_samples_liver)/2):] + post_diagnosis_samples_esophagus[int(len(post_diagnosis_samples_esophagus)/2):] + post_diagnosis_samples_lung[int(len(post_diagnosis_samples_lung)/2):] + post_diagnosis_samples_stomach[int(len(post_diagnosis_samples_stomach)/2):]

positive_pre_leavein = pre_diagnosis_samples_colon[:int(len(pre_diagnosis_samples_colon)/2)] + pre_diagnosis_samples_liver[:int(len(pre_diagnosis_samples_liver)/2)] + pre_diagnosis_samples_esophagus[:int(len(pre_diagnosis_samples_esophagus)/2)] + pre_diagnosis_samples_lung[:int(len(pre_diagnosis_samples_lung)/2)] + pre_diagnosis_samples_stomach[:int(len(pre_diagnosis_samples_stomach)/2)]
positive_pre_leaveout = pre_diagnosis_samples_colon[int(len(pre_diagnosis_samples_colon)/2):] + pre_diagnosis_samples_liver[int(len(pre_diagnosis_samples_liver)/2):] + pre_diagnosis_samples_esophagus[int(len(pre_diagnosis_samples_esophagus)/2):] + pre_diagnosis_samples_lung[int(len(pre_diagnosis_samples_lung)/2):] + pre_diagnosis_samples_stomach[int(len(pre_diagnosis_samples_stomach)/2):]
```

Build a Logistic Regression Classifier

Now, let's build an LR classifier from the leave-in set. We will use the method described in Teschendorff et al, Nature Materials (2019). Briefly:

1. We will randomly split the leave-in set into 50% training, 50% test.
2. We will train an LR classifier on the training set, and compute the results on the test set.
3. We will repeat steps 1 and 2 using 1000 different random splits.
4. We will then compute the ensemble performance of these 1000 models across the ten separate test sets, and report this as the "leave-in set" accuracy.
5. We will then apply all 1000 models to the "leave-out set," compute the ensemble performance, and report this as the "leave-out set" accuracy.

Let's build each LR classifier using 1000 different random splits:

In [13]:
```python
negative_test_list = []
positive_post_test_list = []
positive_pre_test_list = []
z_prob_list = []

for random_state in range(1000):
    random.seed(random_state)

    # Set up LR classifier that will automatically fit the best C value using the training set
    clf = LogisticRegressionCV(penalty='l1', solver='liblinear', random_state=random_state,
                               Cs=[1.0, 5.0, 10.0, 50.0, 100.0], cv=3)

    # Split the leave-in set into training and test set
    negative = list(negative_leavein)
    positive_post = list(positive_post_leavein)
    positive_pre = list(positive_pre_leavein)
    random.shuffle(negative)
    random.shuffle(positive_post)
    random.shuffle(positive_pre)
    negative_training, negative_test = negative[:1*int(len(negative)/2)], negative[int(1*len(negative)/2):]
    positive_post_training, positive_post_test = positive_post[:1*int(len(positive_post)/2)], positive_post[int(1*len(positive_post)/2):]
    positive_pre_training, positive_pre_test = positive_pre[:1*int(len(positive_pre)/2)], positive_pre[int(1*len(positive_pre)/2):]
    positive_training = positive_post_training+positive_pre_training
    positive_test = positive_post_test+positive_pre_test

    # Build the model and set parameters using the training set
    x = amf_df.loc[tissue_t_tests_min_by_marker.index, negative_training+positive_training].transpose()
    y = ['Healthy']*len(negative_training) + ['Cancer']*len(positive_training)
    clf.fit(x,y)

    # Compute the accuracy of this classifier on the leave-in and leave-out sets
    z = pd.Series(clf.predict(amf_df.loc[tissue_t_tests_min_by_marker.index, healthy_samples+post_diagnosis_samples+pre_diagnosis_samples].transpose()),
                  index=healthy_samples+post_diagnosis_samples+pre_diagnosis_samples)
    z_prob = pd.Series(clf.predict_proba(amf_df.loc[tissue_t_tests_min_by_marker.index, healthy_samples+post_diagnosis_samples+pre_diagnosis_samples].transpose())[:, 0],
                       index=healthy_samples+post_diagnosis_samples+pre_diagnosis_samples)

    # Store the results
    negative_test_list.append(negative_test)
    positive_post_test_list.append(positive_post_test)
    positive_pre_test_list.append(positive_pre_test)
    z_prob_list.append(z_prob)
```

Next, let's compute the ensemble performance across the leave-in test sets and the leave-out sets.

In [14]:
```python
ensemble_score_leavein = pd.concat([z_prob_list[index].loc[negative_test_list[index]+positive_pre_test_list[index]+positive_post_test_list[index]]
                                    for index in range(1000)],
                                   axis=1, sort=True).mean(axis=1)
ensemble_score_leaveout = pd.concat([z_prob_list[index].loc[negative_leaveout+positive_post_leaveout+positive_pre_leaveout]
                                     for index in range(1000)],
                                    axis=1, sort=True).mean(axis=1)
```

We can now compute accuracy metrics for the two sets; as per Teschendorff et al 2019, we will choose the model cutoff using the leave-in set.

Leave-In Set Accuracy

Let's choose a cutoff that aims for ~95% specificity in the leave-in set, and compute the associated sensitivity.

In [15]:
```python
sns.set_style('whitegrid')

y_true = ['Healthy']*len(negative_leavein) + ['Cancer']*len(positive_post_leavein)
y_score = ensemble_score_leavein.loc[negative_leavein+positive_post_leavein]
cur_fpr, cur_tpr, cur_cutoff = roc_curve(y_true, y_score, pos_label='Cancer')
cur_auc = auc(cur_fpr, cur_tpr)
plt.plot(cur_fpr, cur_tpr, label=r'Post-Diagnosis ROC (AUC = %0.2f)' % cur_auc)

y_true = ['Healthy']*len(negative_leavein) + ['Cancer']*len(positive_pre_leavein)
y_score = ensemble_score_leavein.loc[negative_leavein+positive_pre_leavein]
cur_fpr, cur_tpr, cur_cutoff = roc_curve(y_true, y_score, pos_label='Cancer')
cur_auc = auc(cur_fpr, cur_tpr)
plt.plot(cur_fpr, cur_tpr, label=r'Pre-Diagnosis ROC (AUC = %0.2f)' % cur_auc)

plt.xlim([-0.0, 1.0])
plt.ylim([-0.0, 1.0])
plt.xticks([0, 0.05, 0.1, 0.2, 0.3, 0.4, 0.5, 0.6, 0.7, 0.8, 0.9, 1.0])
plt.yticks([0, 0.05, 0.1, 0.2, 0.3, 0.4, 0.5, 0.6, 0.7, 0.8, 0.9, 1.0])
plt.xlabel('False Positive Rate')
plt.ylabel('True Positive Rate')
plt.legend(loc="lower right");

print "Leave-In Results"
print "Specificity: %s" % (sum(ensemble_score_leavein.loc[negative_leavein] < 0.581) / len(negative_leavein))
print "Post-Diagnosis Sensitivity: %s" % (sum(ensemble_score_leavein.loc[positive_post_leavein] >= 0.581) / len(positive_post_leavein))
print "Pre-Diagnosis Sensitivity: %s" % (sum(ensemble_score_leavein.loc[positive_pre_leavein] >= 0.581) / len(positive_pre_leavein))

plt.figure()
sns.distplot(ensemble_score_leavein.loc[negative_leavein])
sns.distplot(ensemble_score_leavein.loc[positive_post_leavein])
sns.distplot(ensemble_score_leavein.loc[positive_pre_leavein])
plt.legend(['Healthy', 'Post-Diagnosis', 'Pre-Diagnosis']);
plt.xlim(0, 1)
```

```
Leave-In Results
Specificity: 0.946859903382
Post-Diagnosis Sensitivity: 0.881818181818
Pre-Diagnosis Sensitivity: 0.913978494624
```

Out[15]: (0, 1)

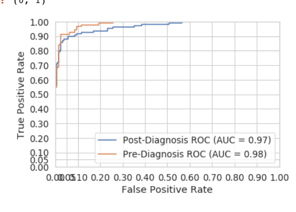

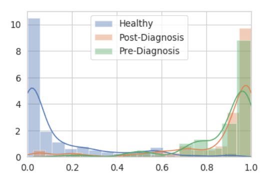

Leave-Out Set Accuracy

Let's compute the leave-out set sensitivity and specificity using the previously chosen cutoff.

In [16]:
```python
sns.set_style('whitegrid')

y_true = ['Healthy']*len(negative_leaveout) + ['Cancer']*len(positive_post_leaveout)
y_score = ensemble_score_leaveout.loc[negative_leaveout+positive_post_leaveout]
cur_fpr, cur_tpr, cur_cutoff = roc_curve(y_true, y_score, pos_label='Cancer')
cur_auc = auc(cur_fpr, cur_tpr)
plt.plot(cur_fpr, cur_tpr, label=r'Post-Diagnosis ROC (AUC = %0.2f)' % cur_auc)

y_true = ['Healthy']*len(negative_leaveout) + ['Cancer']*len(positive_pre_leaveout)
y_score = ensemble_score_leaveout.loc[negative_leaveout+positive_pre_leaveout]
cur_fpr, cur_tpr, cur_cutoff = roc_curve(y_true, y_score, pos_label='Cancer')
cur_auc = auc(cur_fpr, cur_tpr)
plt.plot(cur_fpr, cur_tpr, label=r'Pre-Diagnosis ROC (AUC = %0.2f)' % cur_auc)

plt.xlim([-0.0, 1.0])
plt.ylim([-0.0, 1.0])
plt.xticks([0, 0.05, 0.1, 0.2, 0.3, 0.4, 0.5, 0.6, 0.7, 0.8, 0.9, 1.0])
plt.yticks([0, 0.05, 0.1, 0.2, 0.3, 0.4, 0.5, 0.6, 0.7, 0.8, 0.9, 1.0])
plt.xlabel('False Positive Rate')
plt.ylabel('True Positive Rate')
plt.legend(loc="lower right");

print "Leave-Out Results"
print "Specificity: %s" % (sum(ensemble_score_leaveout.loc[negative_leaveout] < 0.581) / len(negative_leaveout))
print "Post-Diagnosis Sensitivity: %s" % (sum(ensemble_score_leaveout.loc[positive_post_leaveout] >= 0.581) / len(positive_post_leaveout))
print "Pre-Diagnosis Sensitivity: %s" % (sum(ensemble_score_leaveout.loc[positive_pre_leaveout] >= 0.581) / len(positive_pre_leaveout))

plt.figure()
sns.distplot(ensemble_score_leaveout.loc[negative_leaveout])
sns.distplot(ensemble_score_leaveout.loc[positive_post_leaveout])
sns.distplot(ensemble_score_leaveout.loc[positive_pre_leaveout])
plt.legend(['Healthy', 'Post-Diagnosis', 'Pre-Diagnosis']);
plt.xlim(0, 1)
```

```
Leave-Out Results
Specificity: 0.961352657005
Post-Diagnosis Sensitivity: 0.87610619469
Pre-Diagnosis Sensitivity: 0.948979591837
```

Out[16]: (0, 1)

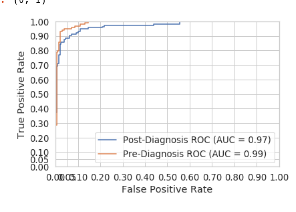

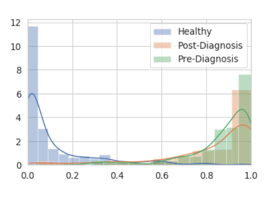

As per Teschendorff et al 2019, the accuracy is similar for the leave-in and leave-out data sets.

Output Data Table

Let's output a data table containing the scores for each cross-validation.

In [17]:
```python
all_scores_leavein = pd.concat([z_prob_list[index].loc[negative_test_list[index]+positive_pre_test_list[index]+positive_post_test_list[index]]
                                for index in range(1000)],
                               axis=1, sort=True).fillna('Training')
all_scores_leavein['Category'] = ['Leave-In']*all_scores_leavein.shape[0]

all_scores_leaveout = pd.concat([z_prob_list[index].loc[negative_leaveout+positive_post_leaveout+positive_pre_leaveout]
                                 for index in range(1000)],
                                axis=1, sort=True)
all_scores_leaveout['Category'] = ['Leave-Out']*all_scores_leaveout.shape[0]

all_scores_merged = pd.concat([all_scores_leavein, all_scores_leaveout], axis=0).replace('Training', np.nan).mean(axis=1)
```

In [18]:
```python
pd.concat([all_scores_leavein, all_scores_leaveout], axis=0).to_csv('tsh_analysis_20191107_cxval_score_table.tsv')
```

Let's also compute the final merged score for each sample by taking the average across all 1000 cross-validations:

In [19]:
```python
pd.DataFrame(pd.concat([all_scores_leavein, all_scores_leaveout], axis=0).replace('Training', np.nan).mean(axis=1)).to_csv('tsh_analysis_20191107_final_score_table.tsv')
```

These scores were utilized to generate the other figures in the manuscript.

Supplementary Note 2.
Code utilized to derive and implement a Linear Discriminant Analysis classifier, as well as a comparison to the Logistic Regression classifier. The LDA-based model provided similar classification accuracy as the LR model, showing that cancer signals can be identified up to four years before diagnosis with multiple machine learning approaches.

Supplementary Note 2

The LDA-based model provided similar classification accuracy as the LR model, showing that cancer signals can be identified up to four years before diagnosis with multiple machine learning approaches.

Approach	Leave-out Specificity	Leave-out Post-Diagnosis Sensitivity	Leave-out Pre-Diagnosis Sensitivity
LR	96.1%	87.6%	94.9%
LDA	94.7%	86.7%	96.9%

The derivation of the LDA classifier was done using the same procedure as the LR model, and is shown below.

Imports

In [1]:
```
from __future__ import division
import sys
import os
import glob
import random
```

In [2]:
```
import pandas as pd
import numpy as np
import scipy
from sklearn.discriminant_analysis import LinearDiscriminantAnalysis
from sklearn.metrics import roc_curve, auc
from statsmodels.stats.multitest import multipletests
import warnings
```

In [3]:
```
%matplotlib inline
import seaborn as sns
import matplotlib.pyplot as plt
```

In [4]:
```
sns.set()
sns.set_context('notebook', font_scale=1.2)
sns.set_style('white')
```

Sample Info

For this study, we processed two sets of samples; one set of plasma samples from the Taizhou Longitudinal Study, and a second set of commercially purchsed normal and cancer tissue samples from BioChain.

Taizhou Longitudinal Study

In [5]:
```
pre_diagnosis_samples = ['TSH-PL180123001', 'TSH-PL180123006', 'TSH-PL180123007', 'TSH-PL18012
3008', 'TSH-PL180123010', 'TSH-PL180123013', 'TSH-PL180123014', 'TSH-PL180123016', 'TSH-PL1801
23017', 'TSH-PL180123018', 'TSH-PL180123021', 'TSH-PL180123022', 'TSH-PL180123025', 'TSH-PL180
123027', 'TSH-PL180123029', 'TSH-PL180123030', 'TSH-PL180123031', 'TSH-PL180123033', 'TSH-PL18
0513004', 'TSH-PL180513005', 'TSH-PL180513006', 'TSH-PL180513007', 'TSH-PL180513011', 'TSH-PL1
80513012', 'TSH-PL180513013', 'TSH-PL180513014', 'TSH-PL180513016', 'TSH-PL180513017', 'TSH-PL
180513020', 'TSH-PL180513021', 'TSH-PL180513023', 'TSH-PL180513024', 'TSH-PL180513025', 'TSH-P
L180513026', 'TSH-PL180513027', 'TSH-PL180513029', 'TSH-PL180513030', 'TSH-PL180513034', 'TSH-
PL180513036', 'TSH-PL180513037', 'TSH-PL180513038', 'TSH-PL180513039', 'TSH-PL180513040', 'TSH
-PL180513041', 'TSH-PL180513043', 'TSH-PL180513045', 'TSH-PL180513049', 'TSH-PL180513050', 'TS
H-PL180513051', 'TSH-PL180513055', 'TSH-PL180513056', 'TSH-PL180513057', 'TSH-PL180513060', 'T
SH-PL180513062', 'TSH-PL180513066', 'TSH-PL180513067', 'TSH-PL180513068', 'TSH-PL180513069', '
TSH-PL180513070', 'TSH-PL180513071', 'TSH-PL180513072', 'TSH-PL180513073', 'TSH-PL180513075',
'TSH-PL180513076', 'TSH-PL180513077', 'TSH-PL180513078', 'TSH-PL180513079', 'TSH-PL180513080',
'TSH-PL180513081', 'TSH-PL180513084', 'TSH-PL180513085', 'TSH-PL180513087', 'TSH-PL180513088',
'TSH-PL180513089', 'TSH-PL180513091', 'TSH-PL180513092', 'TSH-PL180513093', 'TSH-PL180513095',
'TSH-PL180513097', 'TSH-PL180513098', 'TSH-PL180513099', 'TSH-PL180513100', 'TSH-PL180513101',
'TSH-PL180513102', 'TSH-PL180513104', 'TSH-PL180513106', 'TSH-PL180513108', 'TSH-PL180513109',
'TSH-PL180513110', 'TSH-PL180513111', 'TSH-PL180513114', 'TSH-PL180513115', 'TSH-PL180513117',
'TSH-PL180513118', 'TSH-PL180513119', 'TSH-PL180513121', 'TSH-PL180513122', 'TSH-PL180513123',
'TSH-PL180513124', 'TSH-PL180513127', 'TSH-PL180513128', 'TSH-PL180513129', 'TSH-PL180516001',
'TSH-PL180516002', 'TSH-PL180516003', 'TSH-PL180516004', 'TSH-PL180516006', 'TSH-PL180516007',
'TSH-PL180516008', 'TSH-PL180516009', 'TSH-PL180516011', 'TSH-PL180516012', 'TSH-PL180516013',
'TSH-PL180516014', 'TSH-PL180516016', 'TSH-PL180516018', 'TSH-PL180516019', 'TSH-PL180516020',
'TSH-PL180516021', 'TSH-PL180516023', 'TSH-PL180516027', 'TSH-PL180516028', 'TSH-PL180516030',
'TSH-PL180516031', 'TSH-PL180516032', 'TSH-PL180516036', 'TSH-PL180516038', 'TSH-PL180516039',
'TSH-PL180516040', 'TSH-PL180516042', 'TSH-PL180516043', 'TSH-PL180516044', 'TSH-PL180516045',
'TSH-PL180516049', 'TSH-PL180516051', 'TSH-PL180516053', 'TSH-PL180516054', 'TSH-PL180516055',
'TSH-PL180516056', 'TSH-PL180516057', 'TSH-PL180516059', 'TSH-PL180516062', 'TSH-PL180516063',
'TSH-PL180123002', 'TSH-PL180123004', 'TSH-PL180123005', 'TSH-PL180123012', 'TSH-PL180123024',
'TSH-PL180123032', 'TSH-PL180513001', 'TSH-PL180513002', 'TSH-PL180513003', 'TSH-PL180513009',
'TSH-PL180513010', 'TSH-PL180513018', 'TSH-PL180513019', 'TSH-PL180513022', 'TSH-PL180513028',
'TSH-PL180513031', 'TSH-PL180513032', 'TSH-PL180513033', 'TSH-PL180513035', 'TSH-PL180513044',
'TSH-PL180513058', 'TSH-PL180513064', 'TSH-PL180513083', 'TSH-PL180513086', 'TSH-PL180513090',
'TSH-PL180513096', 'TSH-PL180513103', 'TSH-PL180513105', 'TSH-PL180513112', 'TSH-PL180513116',
'TSH-PL180513125', 'TSH-PL180516005', 'TSH-PL180516010', 'TSH-PL180516015', 'TSH-PL180516017',
'TSH-PL180516025', 'TSH-PL180516026', 'TSH-PL180516029', 'TSH-PL180516033', 'TSH-PL180516034',
'TSH-PL180516046', 'TSH-PL180516047', 'TSH-PL180516048', 'TSH-PL180516050', 'TSH-PL180516052',
'TSH-PL180516058', 'TSH-PL180516060', 'TSH-PL180516061']
post_diagnosis_samples = ['TSH-PL171221101', 'TSH-PL171221112', 'TSH-PL171221149', 'TSH-PL1712
21150', 'TSH-PL171221160', 'TSH-PL171221172', 'TSH-PL171221179', 'TSH-PL171221180', 'TSH-PL180
504001', 'TSH-PL180504002', 'TSH-PL180504003', 'TSH-PL180504004', 'TSH-PL180504005', 'TSH-PL18
0504009', 'TSH-PL180504010', 'TSH-PL180504011', 'TSH-PL180504013', 'TSH-PL180504014', 'TSH-PL1
80504015', 'TSH-PL180504016', 'TSH-PL180504017', 'TSH-PL180504018', 'TSH-PL180504019', 'TSH-PL
180504021', 'TSH-PL180504022', 'TSH-PL180504027', 'TSH-PL180504028', 'TSH-PL180504030', 'TSH-P
L180504033', 'TSH-PL180504034', 'TSH-PL180504041', 'TSH-PL180504042', 'TSH-PL180504044', 'TSH-
PL180504046', 'TSH-PL180504047', 'TSH-PL180504049', 'TSH-PL180504050', 'TSH-PL180504053', 'TSH
-PL180504054', 'TSH-PL180504058', 'TSH-PL180504061', 'TSH-PL180504062', 'TSH-PL180504063', 'TS
H-PL180504065', 'TSH-PL180504068', 'TSH-PL180504070', 'TSH-PL180504074', 'TSH-PL180504081', 'T
SH-PL180504083', 'TSH-PL180504087', 'TSH-PL180504088', 'TSH-PL180504089', 'TSH-PL180504091', '
TSH-PL180504094', 'TSH-PL180504097', 'TSH-PL180504101', 'TSH-PL180504104', 'TSH-PL180504108',
'TSH-PL180504111', 'TSH-PL180504118', 'TSH-PL180508001', 'TSH-PL180508004', 'TSH-PL180508005',
'TSH-PL180508006', 'TSH-PL180508008', 'TSH-PL180508009', 'TSH-PL180508011', 'TSH-PL180508012',
'TSH-PL180508013', 'TSH-PL180508014', 'TSH-PL180508015', 'TSH-PL180508019', 'TSH-PL180508020',
'TSH-PL180508021', 'TSH-PL180508022', 'TSH-PL180508025', 'TSH-PL180508030', 'TSH-PL180508031',
'TSH-PL180508033', 'TSH-PL180508034', 'TSH-PL180508036', 'TSH-PL180508037', 'TSH-PL180508038',
'TSH-PL180508039', 'TSH-PL180508040', 'TSH-PL180508041', 'TSH-PL180508042', 'TSH-PL180508043',
'TSH-PL180508049', 'TSH-PL180508050', 'TSH-PL180508060', 'TSH-PL180508061', 'TSH-PL180508064',
'TSH-PL180508068', 'TSH-PL180508076', 'TSH-PL180508078', 'TSH-PL180508083', 'TSH-PL180508086',
'TSH-PL180508089', 'TSH-PL180510005', 'TSH-PL180510006', 'TSH-PL180510007', 'TSH-PL180510008',
'TSH-PL180510010', 'TSH-PL180510011', 'TSH-PL180510012', 'TSH-PL180510014', 'TSH-PL180510015',
'TSH-PL180510016', 'TSH-PL180510017', 'TSH-PL180510018', 'TSH-PL180510019', 'TSH-PL180510021',
'TSH-PL180510023', 'TSH-PL180510024', 'TSH-PL180510025', 'TSH-PL180510026', 'TSH-PL180510030',
'TSH-PL180510031', 'TSH-PL180510033', 'TSH-PL180510038', 'TSH-PL180510040', 'TSH-PL180510044',
'TSH-PL180510047', 'TSH-PL180510050', 'TSH-PL180510052', 'TSH-PL180510053', 'TSH-PL180510054',
'TSH-PL180510055', 'TSH-PL180510057', 'TSH-PL180510061', 'TSH-PL180510063', 'TSH-PL180510066',
'TSH-PL180510071', 'TSH-PL180510072', 'TSH-PL180510073', 'TSH-PL180510074', 'TSH-PL180510075',
'TSH-PL180510076', 'TSH-PL180510077', 'TSH-PL180510078', 'TSH-PL180510079', 'TSH-PL180510080',
'TSH-PL180510085', 'TSH-PL180510089', 'TSH-PL180510092', 'TSH-PL180510095', 'TSH-PL180510101',
'TSH-PL180510104', 'TSH-PL180510105', 'TSH-PL180510107', 'TSH-PL180510112', 'TSH-PL180510121',
'TSH-PL180510122', 'TSH-PL180510125', 'TSH-PL180510139', 'TSH-PL180510140', 'TSH-PL180510146',
'TSH-PL180510147', 'TSH-PL180510162', 'TSH-PL181205168', 'TSH-PL181205170', 'TSH-PL181205173',
'TSH-PL181205175', 'TSH-PL181205182', 'TSH-PL171221151', 'TSH-PL180504006', 'TSH-PL180504012',
'TSH-PL180504024', 'TSH-PL180504026', 'TSH-PL180504029', 'TSH-PL180504037', 'TSH-PL180504043',
'TSH-PL180504045', 'TSH-PL180504051', 'TSH-PL180504052', 'TSH-PL180504055', 'TSH-PL180504057',
'TSH-PL180504064', 'TSH-PL180504076', 'TSH-PL180504084', 'TSH-PL180504110', 'TSH-PL180508003',
'TSH-PL180508007', 'TSH-PL180508023', 'TSH-PL180508026', 'TSH-PL180508028', 'TSH-PL180508029',
'TSH-PL180508032', 'TSH-PL180508044', 'TSH-PL180508052', 'TSH-PL180508065', 'TSH-PL180508067',
'TSH-PL180508088', 'TSH-PL180510002', 'TSH-PL180510004', 'TSH-PL180510013', 'TSH-PL180510020',
'TSH-PL180510022', 'TSH-PL180510032', 'TSH-PL180510034', 'TSH-PL180510035', 'TSH-PL180510043',
'TSH-PL180510048', 'TSH-PL180510068', 'TSH-PL180510088', 'TSH-PL180510090', 'TSH-PL180510098',
'TSH-PL180510100', 'TSH-PL180510103', 'TSH-PL180510108', 'TSH-PL180510109', 'TSH-PL180510113',
'TSH-PL180510117', 'TSH-PL180510124', 'TSH-PL180510143', 'TSH-PL181205169', 'TSH-PL181205172',
'TSH-PL181205174', 'TSH-PL181205177', 'TSH-PL181205179', 'TSH-PL171221153', 'TSH-PL171221113']
healthy_samples = ['TSH-PL180309087', 'TSH-PL180316003', 'TSH-PL180316005', 'TSH-PL180316016',
'TSH-PL180316017', 'TSH-PL180316018', 'TSH-PL180316022', 'TSH-PL180316024', 'TSH-PL180316027',
'TSH-PL180316033', 'TSH-PL180316037', 'TSH-PL180316038', 'TSH-PL180316039', 'TSH-PL180316049',
'TSH-PL180316061', 'TSH-PL180316065', 'TSH-PL180316079', 'TSH-PL180316092', 'TSH-PL180316095',
'TSH-PL180316099', 'TSH-PL180316101', 'TSH-PL180316106', 'TSH-PL180316115', 'TSH-PL180316119',
'TSH-PL180316126', 'TSH-PL180316129', 'TSH-PL180316157', 'TSH-PL180316161', 'TSH-PL180316163',
'TSH-PL180316164', 'TSH-PL180316173', 'TSH-PL180316174', 'TSH-PL180316181', 'TSH-PL180316183',
'TSH-PL180316187', 'TSH-PL180316190', 'TSH-PL180316195', 'TSH-PL180316196', 'TSH-PL180316198',
'TSH-PL180323034', 'TSH-PL180323038', 'TSH-PL180323051', 'TSH-PL180323052', 'TSH-PL180323054',
'TSH-PL180323059', 'TSH-PL180323065', 'TSH-PL180323067', 'TSH-PL180323072', 'TSH-PL180323073',
'TSH-PL180323091', 'TSH-PL180323106', 'TSH-PL180323107', 'TSH-PL180323109', 'TSH-PL180323110',
'TSH-PL180323111', 'TSH-PL180323119', 'TSH-PL180323121', 'TSH-PL180323124', 'TSH-PL180323139',
'TSH-PL180323141', 'TSH-PL180323142', 'TSH-PL180323151', 'TSH-PL180323155', 'TSH-PL180323166',
'TSH-PL180410003', 'TSH-PL180410023', 'TSH-PL180410043', 'TSH-PL180410049', 'TSH-PL180410059',
'TSH-PL180410063', 'TSH-PL180410076', 'TSH-PL180410087', 'TSH-PL180410115', 'TSH-PL180410116',
'TSH-PL180410133', 'TSH-PL180410140', 'TSH-PL180410144', 'TSH-PL180410160', 'TSH-PL180410162',
'TSH-PL180410198', 'TSH-PL180417003', 'TSH-PL180417007', 'TSH-PL180417035', 'TSH-PL180417060',
'TSH-PL180417074', 'TSH-PL180417087', 'TSH-PL180417095', 'TSH-PL180417101', 'TSH-PL180417109',
'TSH-PL180417117', 'TSH-PL180417135', 'TSH-PL180417138', 'TSH-PL180417143', 'TSH-PL180417152',
'TSH-PL180417156', 'TSH-PL180417165', 'TSH-PL180417170', 'TSH-PL180417179', 'TSH-PL180417180',
'TSH-PL180417181', 'TSH-PL180417200', 'TSH-PL180424003', 'TSH-PL180424030', 'TSH-PL180424036',
'TSH-PL180424058', 'TSH-PL180424090', 'TSH-PL180424097', 'TSH-PL180424104', 'TSH-PL180424115',
'TSH-PL180424146', 'TSH-PL180424149', 'TSH-PL180424155', 'TSH-PL180424162', 'TSH-PL180424166',
'TSH-PL180424171', 'TSH-PL180703037', 'TSH-PL180703136', 'TSH-PL180703153', 'TSH-PL180703157',
'TSH-PL180703185', 'TSH-PL180706034', 'TSH-PL180706037', 'TSH-PL180706095', 'TSH-PL180706096',
'TSH-PL180706104', 'TSH-PL180706113', 'TSH-PL180706118', 'TSH-PL180706145', 'TSH-PL180706147',
'TSH-PL180706150', 'TSH-PL180706153', 'TSH-PL180706156', 'TSH-PL181128001', 'TSH-PL181128003',
'TSH-PL181128005', 'TSH-PL181128007', 'TSH-PL181128008', 'TSH-PL181128009', 'TSH-PL181128011',
'TSH-PL181128012', 'TSH-PL181128014', 'TSH-PL181128015', 'TSH-PL181128019', 'TSH-PL181128021',
'TSH-PL181128022', 'TSH-PL181128023', 'TSH-PL181128025', 'TSH-PL181128027', 'TSH-PL181128029',
'TSH-PL181128030', 'TSH-PL181128033', 'TSH-PL181128035', 'TSH-PL181128036', 'TSH-PL181128038',
'TSH-PL181128039', 'TSH-PL181128040', 'TSH-PL181128041', 'TSH-PL181128042', 'TSH-PL181128043',
'TSH-PL181128045', 'TSH-PL181128047', 'TSH-PL181128048', 'TSH-PL181128049', 'TSH-PL181128050',
'TSH-PL181128051', 'TSH-PL181128054', 'TSH-PL181128055', 'TSH-PL181128056', 'TSH-PL181128057',
'TSH-PL181128058', 'TSH-PL181128061', 'TSH-PL181128062', 'TSH-PL181128064', 'TSH-PL181128065',
'TSH-PL181128066', 'TSH-PL181128070', 'TSH-PL181128073', 'TSH-PL181128077', 'TSH-PL181128080',
'TSH-PL181128082', 'TSH-PL181128083', 'TSH-PL181128085', 'TSH-PL181128086', 'TSH-PL181128093',
'TSH-PL181128096', 'TSH-PL181128099', 'TSH-PL181128101', 'TSH-PL181128102', 'TSH-PL181128103',
'TSH-PL181128104', 'TSH-PL181128107', 'TSH-PL181128109', 'TSH-PL181128110', 'TSH-PL181128111',
'TSH-PL181128112', 'TSH-PL181128114', 'TSH-PL181128115', 'TSH-PL181128117', 'TSH-PL181128118',
'TSH-PL181128120', 'TSH-PL181128122', 'TSH-PL181128123', 'TSH-PL181128124', 'TSH-PL181128125',
'TSH-PL181128126', 'TSH-PL181128127', 'TSH-PL181205002', 'TSH-PL181205003', 'TSH-PL181205005',
'TSH-PL181205006', 'TSH-PL181205008', 'TSH-PL181205009', 'TSH-PL181205013', 'TSH-PL181205014',
'TSH-PL181205016', 'TSH-PL181205020', 'TSH-PL181205022', 'TSH-PL181205024', 'TSH-PL181205026',
'TSH-PL181205027', 'TSH-PL181205028', 'TSH-PL181205030', 'TSH-PL181205033', 'TSH-PL181205035',
'TSH-PL181205040', 'TSH-PL181205041', 'TSH-PL181205042', 'TSH-PL181205043', 'TSH-PL181205044',
'TSH-PL181205045', 'TSH-PL181205046', 'TSH-PL181205047', 'TSH-PL181205048', 'TSH-PL181205049',
'TSH-PL181205051', 'TSH-PL181205053', 'TSH-PL181205054', 'TSH-PL181205055', 'TSH-PL181205057',
'TSH-PL181205058', 'TSH-PL181205059', 'TSH-PL181205060', 'TSH-PL181205061', 'TSH-PL181205062',
'TSH-PL181205063', 'TSH-PL181205064', 'TSH-PL181205065', 'TSH-PL181205066', 'TSH-PL181205068',
'TSH-PL181205069', 'TSH-PL181205072', 'TSH-PL181205074', 'TSH-PL181205075', 'TSH-PL181205076',
'TSH-PL181205077', 'TSH-PL181205078', 'TSH-PL181205079', 'TSH-PL181205081', 'TSH-PL181205082',
'TSH-PL181205083', 'TSH-PL181205085', 'TSH-PL181205086', 'TSH-PL181205087', 'TSH-PL181205088',
'TSH-PL181205090', 'TSH-PL181205091', 'TSH-PL181205093', 'TSH-PL181205095', 'TSH-PL181205096',
'TSH-PL181205097', 'TSH-PL181205098', 'TSH-PL181205099', 'TSH-PL181205102', 'TSH-PL181205103',
'TSH-PL181205104', 'TSH-PL181205107', 'TSH-PL181205108', 'TSH-PL181205110', 'TSH-PL181205113',
'TSH-PL181205115', 'TSH-PL181205116', 'TSH-PL181205117', 'TSH-PL181205121', 'TSH-PL181205122',
'TSH-PL181205125', 'TSH-PL181205128', 'TSH-PL181205129', 'TSH-PL181205140', 'TSH-PL181205141',
'TSH-PL181205142', 'TSH-PL181205143', 'TSH-PL181205144', 'TSH-PL181205145', 'TSH-PL181205146',
'TSH-PL181205147', 'TSH-PL181205148', 'TSH-PL181205149', 'TSH-PL181205150', 'TSH-PL181205151',
'TSH-PL181205152', 'TSH-PL181205155', 'TSH-PL181205156', 'TSH-PL181205157', 'TSH-PL181205158',
'TSH-PL181205160', 'TSH-PL181205161', 'TSH-PL181205162', 'TSH-PL181205164', 'TSH-PL181205165',
'TSH-PL181205166', 'TSH-PL180309082', 'TSH-PL180316013', 'TSH-PL180316029', 'TSH-PL180316075',
'TSH-PL180316102', 'TSH-PL180316112', 'TSH-PL180316134', 'TSH-PL180316138', 'TSH-PL180316175',
'TSH-PL180316178', 'TSH-PL180316200', 'TSH-PL180323029', 'TSH-PL180323032', 'TSH-PL180323053',
'TSH-PL180323068', 'TSH-PL180323074', 'TSH-PL180323088', 'TSH-PL180323097', 'TSH-PL180323103',
'TSH-PL180323115', 'TSH-PL180323120', 'TSH-PL180323160', 'TSH-PL180323175', 'TSH-PL180410058',
'TSH-PL180410070', 'TSH-PL180410081', 'TSH-PL180410148', 'TSH-PL180410161', 'TSH-PL180417017',
'TSH-PL180417023', 'TSH-PL180417045', 'TSH-PL180417077', 'TSH-PL180417104', 'TSH-PL180417111',
'TSH-PL180417124', 'TSH-PL180417162', 'TSH-PL180417169', 'TSH-PL180417199', 'TSH-PL180424042',
'TSH-PL180424088', 'TSH-PL180424138', 'TSH-PL180703012', 'TSH-PL180703127', 'TSH-PL180703134',
'TSH-PL180703141', 'TSH-PL180703168', 'TSH-PL180706117', 'TSH-PL181128004', 'TSH-PL181128006',
'TSH-PL181128013', 'TSH-PL181128018', 'TSH-PL181128020', 'TSH-PL181128031', 'TSH-PL181128037',
'TSH-PL181128067', 'TSH-PL181128071', 'TSH-PL181128078', 'TSH-PL181128084', 'TSH-PL181128095',
'TSH-PL181128097', 'TSH-PL181128098', 'TSH-PL181128105', 'TSH-PL181128106', 'TSH-PL181128108',
'TSH-PL181128113', 'TSH-PL181128116', 'TSH-PL181205001', 'TSH-PL181205004', 'TSH-PL181205007',
'TSH-PL181205010', 'TSH-PL181205011', 'TSH-PL181205012', 'TSH-PL181205015', 'TSH-PL181205018',
'TSH-PL181205019', 'TSH-PL181205023', 'TSH-PL181205031', 'TSH-PL181205032', 'TSH-PL181205036',
'TSH-PL181205037', 'TSH-PL181205052', 'TSH-PL181205056', 'TSH-PL181205067', 'TSH-PL181205070',
'TSH-PL181205071', 'TSH-PL181205073', 'TSH-PL181205080', 'TSH-PL181205084', 'TSH-PL181205089',
'TSH-PL181205092', 'TSH-PL181205094', 'TSH-PL181205100', 'TSH-PL181205101', 'TSH-PL181205106',
'TSH-PL181205112', 'TSH-PL181205118', 'TSH-PL181205119', 'TSH-PL181205120', 'TSH-PL181205123',
'TSH-PL181205124', 'TSH-PL181205153', 'TSH-PL181205154', 'TSH-PL181205159', 'TSH-PL181205163']
```

In [6]:
```
healthy_samples = ['TSH-PL180309087', 'TSH-PL180316003', 'TSH-PL180316005', 'TSH-PL180316016',
'TSH-PL180316017', 'TSH-PL180316018', 'TSH-PL180316022', 'TSH-PL180316024', 'TSH-PL180316027',
'TSH-PL180316033', 'TSH-PL180316037', 'TSH-PL180316038', 'TSH-PL180316039', 'TSH-PL180316049',
'TSH-PL180316061', 'TSH-PL180316065', 'TSH-PL180316079', 'TSH-PL180316092', 'TSH-PL180316095',
'TSH-PL180316099', 'TSH-PL180316101', 'TSH-PL180316106', 'TSH-PL180316115', 'TSH-PL180316119',
'TSH-PL180316126', 'TSH-PL180316129', 'TSH-PL180316157', 'TSH-PL180316161', 'TSH-PL180316163',
'TSH-PL180316164', 'TSH-PL180316173', 'TSH-PL180316174', 'TSH-PL180316181', 'TSH-PL180316183',
'TSH-PL180316187', 'TSH-PL180316190', 'TSH-PL180316195', 'TSH-PL180316196', 'TSH-PL180316198',
'TSH-PL180323034', 'TSH-PL180323038', 'TSH-PL180323051', 'TSH-PL180323052', 'TSH-PL180323054',
'TSH-PL180323059', 'TSH-PL180323065', 'TSH-PL180323067', 'TSH-PL180323072', 'TSH-PL180323073',
'TSH-PL180323091', 'TSH-PL180323106', 'TSH-PL180323107', 'TSH-PL180323109', 'TSH-PL180323110',
'TSH-PL180323111', 'TSH-PL180323119', 'TSH-PL180323121', 'TSH-PL180323124', 'TSH-PL180323139',
'TSH-PL180323141', 'TSH-PL180323142', 'TSH-PL180323151', 'TSH-PL180323155', 'TSH-PL180323166',
'TSH-PL180410003', 'TSH-PL180410023', 'TSH-PL180410043', 'TSH-PL180410049', 'TSH-PL180410059',
'TSH-PL180410063', 'TSH-PL180410076', 'TSH-PL180410087', 'TSH-PL180410115', 'TSH-PL180410116',
'TSH-PL180410133', 'TSH-PL180410140', 'TSH-PL180410144', 'TSH-PL180410160', 'TSH-PL180410162',
'TSH-PL180410198', 'TSH-PL180417003', 'TSH-PL180417007', 'TSH-PL180417035', 'TSH-PL180417060',
'TSH-PL180417074', 'TSH-PL180417087', 'TSH-PL180417095', 'TSH-PL180417101', 'TSH-PL180417109',
'TSH-PL180417117', 'TSH-PL180417135', 'TSH-PL180417138', 'TSH-PL180417143', 'TSH-PL180417152',
'TSH-PL180417156', 'TSH-PL180417165', 'TSH-PL180417170', 'TSH-PL180417179', 'TSH-PL180417180',
'TSH-PL180417181', 'TSH-PL180417200', 'TSH-PL180424003', 'TSH-PL180424030', 'TSH-PL180424036',
'TSH-PL180424058', 'TSH-PL180424090', 'TSH-PL180424097', 'TSH-PL180424104', 'TSH-PL180424115',
'TSH-PL180424146', 'TSH-PL180424149', 'TSH-PL180424155', 'TSH-PL180424162', 'TSH-PL180424166',
'TSH-PL180424171', 'TSH-PL180703037', 'TSH-PL180703136', 'TSH-PL180703153', 'TSH-PL180703157',
'TSH-PL180703185', 'TSH-PL180706034', 'TSH-PL180706037', 'TSH-PL180706095', 'TSH-PL180706096',
'TSH-PL180706104', 'TSH-PL180706113', 'TSH-PL180706118', 'TSH-PL180706145', 'TSH-PL180706147',
'TSH-PL180706150', 'TSH-PL180706153', 'TSH-PL180706156', 'TSH-PL181128001', 'TSH-PL181128003',
'TSH-PL181128005', 'TSH-PL181128007', 'TSH-PL181128008', 'TSH-PL181128009', 'TSH-PL181128011',
'TSH-PL181128012', 'TSH-PL181128014', 'TSH-PL181128015', 'TSH-PL181128019', 'TSH-PL181128021',
'TSH-PL181128022', 'TSH-PL181128023', 'TSH-PL181128025', 'TSH-PL181128027', 'TSH-PL181128029',
'TSH-PL181128030', 'TSH-PL181128033', 'TSH-PL181128035', 'TSH-PL181128036', 'TSH-PL181128038',
'TSH-PL181128039', 'TSH-PL181128040', 'TSH-PL181128041', 'TSH-PL181128042', 'TSH-PL181128043',
'TSH-PL181128045', 'TSH-PL181128047', 'TSH-PL181128048', 'TSH-PL181128049', 'TSH-PL181128050',
'TSH-PL181128051', 'TSH-PL181128054', 'TSH-PL181128055', 'TSH-PL181128056', 'TSH-PL181128057',
'TSH-PL181128058', 'TSH-PL181128061', 'TSH-PL181128062', 'TSH-PL181128064', 'TSH-PL181128065',
'TSH-PL181128066', 'TSH-PL181128070', 'TSH-PL181128073', 'TSH-PL181128077', 'TSH-PL181128080',
'TSH-PL181128082', 'TSH-PL181128083', 'TSH-PL181128085', 'TSH-PL181128086', 'TSH-PL181128093',
'TSH-PL181128096', 'TSH-PL181128099', 'TSH-PL181128101', 'TSH-PL181128102', 'TSH-PL181128103',
'TSH-PL181128104', 'TSH-PL181128107', 'TSH-PL181128109', 'TSH-PL181128110', 'TSH-PL181128111',
'TSH-PL181128112', 'TSH-PL181128114', 'TSH-PL181128115', 'TSH-PL181128117', 'TSH-PL181128118',
'TSH-PL181128120', 'TSH-PL181128122', 'TSH-PL181128123', 'TSH-PL181128124', 'TSH-PL181128125',
'TSH-PL181128126', 'TSH-PL181128127', 'TSH-PL181205002', 'TSH-PL181205003', 'TSH-PL181205005',
'TSH-PL181205006', 'TSH-PL181205008', 'TSH-PL181205009', 'TSH-PL181205013', 'TSH-PL181205014',
'TSH-PL181205016', 'TSH-PL181205020', 'TSH-PL181205022', 'TSH-PL181205024', 'TSH-PL181205026',
'TSH-PL181205027', 'TSH-PL181205028', 'TSH-PL181205030', 'TSH-PL181205033', 'TSH-PL181205035',
'TSH-PL181205040', 'TSH-PL181205041', 'TSH-PL181205042', 'TSH-PL181205043', 'TSH-PL181205044',
'TSH-PL181205045', 'TSH-PL181205046', 'TSH-PL181205047', 'TSH-PL181205048', 'TSH-PL181205049',
'TSH-PL181205051', 'TSH-PL181205053', 'TSH-PL181205054', 'TSH-PL181205055', 'TSH-PL181205057',
'TSH-PL181205058', 'TSH-PL181205059', 'TSH-PL181205060', 'TSH-PL181205061', 'TSH-PL181205062',
'TSH-PL181205063', 'TSH-PL181205064', 'TSH-PL181205065', 'TSH-PL181205066', 'TSH-PL181205068',
'TSH-PL181205069', 'TSH-PL181205072', 'TSH-PL181205074', 'TSH-PL181205075', 'TSH-PL181205076',
'TSH-PL181205077', 'TSH-PL181205078', 'TSH-PL181205079', 'TSH-PL181205081', 'TSH-PL181205082',
'TSH-PL181205083', 'TSH-PL181205085', 'TSH-PL181205086', 'TSH-PL181205087', 'TSH-PL181205088',
'TSH-PL181205090', 'TSH-PL181205091', 'TSH-PL181205093', 'TSH-PL181205095', 'TSH-PL181205096',
'TSH-PL181205097', 'TSH-PL181205098', 'TSH-PL181205099', 'TSH-PL181205102', 'TSH-PL181205103',
'TSH-PL181205104', 'TSH-PL181205107', 'TSH-PL181205108', 'TSH-PL181205110', 'TSH-PL181205113',
'TSH-PL181205115', 'TSH-PL181205116', 'TSH-PL181205117', 'TSH-PL181205121', 'TSH-PL181205122',
'TSH-PL181205125', 'TSH-PL181205128', 'TSH-PL181205129', 'TSH-PL181205140', 'TSH-PL181205141',
'TSH-PL181205142', 'TSH-PL181205143', 'TSH-PL181205144', 'TSH-PL181205145', 'TSH-PL181205146',
'TSH-PL181205147', 'TSH-PL181205148', 'TSH-PL181205149', 'TSH-PL181205150', 'TSH-PL181205151',
'TSH-PL181205152', 'TSH-PL181205155', 'TSH-PL181205156', 'TSH-PL181205157', 'TSH-PL181205158',
'TSH-PL181205160', 'TSH-PL181205161', 'TSH-PL181205162', 'TSH-PL181205164', 'TSH-PL181205165',
'TSH-PL181205166', 'TSH-PL180309082', 'TSH-PL180316013', 'TSH-PL180316029', 'TSH-PL180316075',
'TSH-PL180316102', 'TSH-PL180316112', 'TSH-PL180316134', 'TSH-PL180316138', 'TSH-PL180316175',
'TSH-PL180316178', 'TSH-PL180316200', 'TSH-PL180323029', 'TSH-PL180323032', 'TSH-PL180323053',
'TSH-PL180323068', 'TSH-PL180323074', 'TSH-PL180323088', 'TSH-PL180323097', 'TSH-PL180323103',
'TSH-PL180323115', 'TSH-PL180323120', 'TSH-PL180323160', 'TSH-PL180323175', 'TSH-PL180410058',
'TSH-PL180410070', 'TSH-PL180410081', 'TSH-PL180410148', 'TSH-PL180410161', 'TSH-PL180417017',
'TSH-PL180417023', 'TSH-PL180417045', 'TSH-PL180417077', 'TSH-PL180417104', 'TSH-PL180417111',
'TSH-PL180417124', 'TSH-PL180417162', 'TSH-PL180417169', 'TSH-PL180417199', 'TSH-PL180424042',
'TSH-PL180424088', 'TSH-PL180424138', 'TSH-PL180703012', 'TSH-PL180703127', 'TSH-PL180703134',
'TSH-PL180703141', 'TSH-PL180703168', 'TSH-PL180706117', 'TSH-PL181128004', 'TSH-PL181128006',
'TSH-PL181128013', 'TSH-PL181128018', 'TSH-PL181128020', 'TSH-PL181128031', 'TSH-PL181128037',
'TSH-PL181128067', 'TSH-PL181128071', 'TSH-PL181128078', 'TSH-PL181128084', 'TSH-PL181128095',
'TSH-PL181128097', 'TSH-PL181128098', 'TSH-PL181128105', 'TSH-PL181128106', 'TSH-PL181128108',
'TSH-PL181128113', 'TSH-PL181128116', 'TSH-PL181205001', 'TSH-PL181205004', 'TSH-PL181205007',
'TSH-PL181205010', 'TSH-PL181205011', 'TSH-PL181205012', 'TSH-PL181205015', 'TSH-PL181205018',
'TSH-PL181205019', 'TSH-PL181205023', 'TSH-PL181205031', 'TSH-PL181205032', 'TSH-PL181205036',
'TSH-PL181205037', 'TSH-PL181205052', 'TSH-PL181205056', 'TSH-PL181205067', 'TSH-PL181205070',
'TSH-PL181205071', 'TSH-PL181205073', 'TSH-PL181205080', 'TSH-PL181205084', 'TSH-PL181205089',
'TSH-PL181205092', 'TSH-PL181205094', 'TSH-PL181205100', 'TSH-PL181205101', 'TSH-PL181205106',
'TSH-PL181205112', 'TSH-PL181205118', 'TSH-PL181205119', 'TSH-PL181205120', 'TSH-PL181205123',
'TSH-PL181205124', 'TSH-PL181205153', 'TSH-PL181205154', 'TSH-PL181205159', 'TSH-PL181205163']

post_diagnosis_samples_colon = ['TSH-PL180510139', 'TSH-PL180510140', 'TSH-PL180510146', 'TSH-
PL180510147', 'TSH-PL180510162', 'TSH-PL181205175', 'TSH-PL180510143']
post_diagnosis_samples_liver = ['TSH-PL180510085', 'TSH-PL180510089', 'TSH-PL180510092', 'TSH-
PL180510095', 'TSH-PL180510101', 'TSH-PL180510104', 'TSH-PL180510105', 'TSH-PL180510107', 'TSH
-PL180510112', 'TSH-PL180510121', 'TSH-PL180510122', 'TSH-PL180510125', 'TSH-PL181205173', 'TS
H-PL180510088', 'TSH-PL180510090', 'TSH-PL180510098', 'TSH-PL180510100', 'TSH-PL180510103', 'T
SH-PL180510108', 'TSH-PL180510109', 'TSH-PL180510113', 'TSH-PL180510117', 'TSH-PL180510124']
post_diagnosis_samples_esophagus = ['TSH-PL171221101', 'TSH-PL171221112', 'TSH-PL171221149', '
TSH-PL171221150', 'TSH-PL171221160', 'TSH-PL171221172', 'TSH-PL171221179', 'TSH-PL171221180',
'TSH-PL180508001', 'TSH-PL180508004', 'TSH-PL180508005', 'TSH-PL180508006', 'TSH-PL180508008',
'TSH-PL180508009', 'TSH-PL180508011', 'TSH-PL180508012', 'TSH-PL180508013', 'TSH-PL180508014',
'TSH-PL180508015', 'TSH-PL180508019', 'TSH-PL180508020', 'TSH-PL180508021', 'TSH-PL180508022',
'TSH-PL180508025', 'TSH-PL180508030', 'TSH-PL180508031', 'TSH-PL180508033', 'TSH-PL180508034',
'TSH-PL180508036', 'TSH-PL180508037', 'TSH-PL180508038', 'TSH-PL180508039', 'TSH-PL180508040',
'TSH-PL180508041', 'TSH-PL180508042', 'TSH-PL180508043', 'TSH-PL180508049', 'TSH-PL180508050',
'TSH-PL180508060', 'TSH-PL180508061', 'TSH-PL180508064', 'TSH-PL180508068', 'TSH-PL180508076',
'TSH-PL180508078', 'TSH-PL180508083', 'TSH-PL180508086', 'TSH-PL180508089', 'TSH-PL180510044',
'TSH-PL180510061', 'TSH-PL181205168', 'TSH-PL181205170', 'TSH-PL171221151', 'TSH-PL180508003',
'TSH-PL180508007', 'TSH-PL180508023', 'TSH-PL180508026', 'TSH-PL180508028', 'TSH-PL180508029',
'TSH-PL180508032', 'TSH-PL180508044', 'TSH-PL180508052', 'TSH-PL180508065', 'TSH-PL180508067',
'TSH-PL180508088', 'TSH-PL181205172', 'TSH-PL181205174', 'TSH-PL171221153', 'TSH-PL171221113']
post_diagnosis_samples_lung = ['TSH-PL180510005', 'TSH-PL180510006', 'TSH-PL180510007', 'TSH-P
L180510008', 'TSH-PL180510010', 'TSH-PL180510011', 'TSH-PL180510012', 'TSH-PL180510014', 'TSH-
PL180510015', 'TSH-PL180510016', 'TSH-PL180510017', 'TSH-PL180510018', 'TSH-PL180510019', 'TSH
-PL180510021', 'TSH-PL180510023', 'TSH-PL180510024', 'TSH-PL180510025', 'TSH-PL180510026', 'TS
H-PL180510030', 'TSH-PL180510031', 'TSH-PL180510033', 'TSH-PL180510038', 'TSH-PL180510040', 'T
SH-PL180510047', 'TSH-PL180510050', 'TSH-PL180510052', 'TSH-PL180510053', 'TSH-PL180510054', '
TSH-PL180510055', 'TSH-PL180510057', 'TSH-PL180510063', 'TSH-PL180510066', 'TSH-PL180510071',
'TSH-PL180510072', 'TSH-PL180510073', 'TSH-PL180510074', 'TSH-PL180510075', 'TSH-PL180510076',
'TSH-PL180510077', 'TSH-PL180510078', 'TSH-PL180510079', 'TSH-PL180510080', 'TSH-PL181205182',
'TSH-PL180510002', 'TSH-PL180510004', 'TSH-PL180510013', 'TSH-PL180510020', 'TSH-PL180510022',
'TSH-PL180510032', 'TSH-PL180510034', 'TSH-PL180510035', 'TSH-PL180510043', 'TSH-PL180510048',
'TSH-PL180510068', 'TSH-PL181205177', 'TSH-PL181205179']
post_diagnosis_samples_stomach = ['TSH-PL180504001', 'TSH-PL180504002', 'TSH-PL180504003', 'TS
H-PL180504004', 'TSH-PL180504005', 'TSH-PL180504009', 'TSH-PL180504010', 'TSH-PL180504011', 'T
SH-PL180504013', 'TSH-PL180504014', 'TSH-PL180504015', 'TSH-PL180504016', 'TSH-PL180504017', '
TSH-PL180504018', 'TSH-PL180504019', 'TSH-PL180504021', 'TSH-PL180504022', 'TSH-PL180504027',
'TSH-PL180504028', 'TSH-PL180504030', 'TSH-PL180504033', 'TSH-PL180504034', 'TSH-PL180504041',
'TSH-PL180504042', 'TSH-PL180504044', 'TSH-PL180504046', 'TSH-PL180504047', 'TSH-PL180504049',
'TSH-PL180504050', 'TSH-PL180504053', 'TSH-PL180504054', 'TSH-PL180504058', 'TSH-PL180504061',
'TSH-PL180504062', 'TSH-PL180504063', 'TSH-PL180504065', 'TSH-PL180504068', 'TSH-PL180504070',
'TSH-PL180504074', 'TSH-PL180504081', 'TSH-PL180504083', 'TSH-PL180504087', 'TSH-PL180504088',
'TSH-PL180504089', 'TSH-PL180504091', 'TSH-PL180504094', 'TSH-PL180504097', 'TSH-PL180504101',
'TSH-PL180504104', 'TSH-PL180504108', 'TSH-PL180504111', 'TSH-PL180504118', 'TSH-PL180504006',
'TSH-PL180504012', 'TSH-PL180504024', 'TSH-PL180504026', 'TSH-PL180504029', 'TSH-PL180504037',
'TSH-PL180504043', 'TSH-PL180504045', 'TSH-PL180504051', 'TSH-PL180504052', 'TSH-PL180504055',
'TSH-PL180504057', 'TSH-PL180504064', 'TSH-PL180504076', 'TSH-PL180504084', 'TSH-PL180504110',
'TSH-PL181205169']

pre_diagnosis_samples_colon = ['TSH-PL180123001', 'TSH-PL180123010', 'TSH-PL180513013', 'TSH-P
L180513014', 'TSH-PL180513020', 'TSH-PL180513027', 'TSH-PL180513029', 'TSH-PL180513030', 'TSH-
PL180513034', 'TSH-PL180513037', 'TSH-PL180513049', 'TSH-PL180513062', 'TSH-PL180513077', 'TSH
-PL180513078', 'TSH-PL180513085', 'TSH-PL180513114', 'TSH-PL180516003', 'TSH-PL180516011', 'TS
H-PL180516012', 'TSH-PL180516014', 'TSH-PL180516018', 'TSH-PL180516021', 'TSH-PL180516028', 'T
SH-PL180516039', 'TSH-PL180516044', 'TSH-PL180516053', 'TSH-PL180516057', 'TSH-PL180513001', '
TSH-PL180513002', 'TSH-PL180513064', 'TSH-PL180513116', 'TSH-PL180516015', 'TSH-PL180516017',
'TSH-PL180516026', 'TSH-PL180516033']
pre_diagnosis_samples_liver = ['TSH-PL180123014', 'TSH-PL180123031', 'TSH-PL180513006', 'TSH-P
L180513011', 'TSH-PL180513017', 'TSH-PL180513021', 'TSH-PL180513026', 'TSH-PL180513041', 'TSH-
PL180513043', 'TSH-PL180513071', 'TSH-PL180513088', 'TSH-PL180513095', 'TSH-PL180513098', 'TSH
-PL180513108', 'TSH-PL180513109', 'TSH-PL180513111', 'TSH-PL180513117', 'TSH-PL180516006', 'TS
H-PL180516038', 'TSH-PL180516040', 'TSH-PL180516051', 'TSH-PL180516054', 'TSH-PL180516063', 'T
SH-PL180123004', 'TSH-PL180123005', 'TSH-PL180513009', 'TSH-PL180513033', 'TSH-PL180513090', '
TSH-PL180516025']
pre_diagnosis_samples_esophagus = ['TSH-PL180123006', 'TSH-PL180123007', 'TSH-PL180123008', 'T
SH-PL180123016', 'TSH-PL180123025', 'TSH-PL180513004', 'TSH-PL180513005', 'TSH-PL180513016', '
TSH-PL180513051', 'TSH-PL180513068', 'TSH-PL180513072', 'TSH-PL180513075', 'TSH-PL180513080',
'TSH-PL180513081', 'TSH-PL180513091', 'TSH-PL180513093', 'TSH-PL180513097', 'TSH-PL180513100',
'TSH-PL180513101', 'TSH-PL180513110', 'TSH-PL180513115', 'TSH-PL180513118', 'TSH-PL180513121',
'TSH-PL180513122', 'TSH-PL180513124', 'TSH-PL180513128', 'TSH-PL180513129', 'TSH-PL180516013',
'TSH-PL180516020', 'TSH-PL180516030', 'TSH-PL180516031', 'TSH-PL180516045', 'TSH-PL180516049',
'TSH-PL180516059', 'TSH-PL180123024', 'TSH-PL180123032', 'TSH-PL180513028', 'TSH-PL180513032',
'TSH-PL180513058', 'TSH-PL180513083', 'TSH-PL180513096', 'TSH-PL180516005', 'TSH-PL180516047',
'TSH-PL180516050', 'TSH-PL180516061']
pre_diagnosis_samples_lung = ['TSH-PL180123013', 'TSH-PL180123021', 'TSH-PL180123022', 'TSH-PL
180123029', 'TSH-PL180123030', 'TSH-PL180513007', 'TSH-PL180513012', 'TSH-PL180513023', 'TSH-P
L180513024', 'TSH-PL180513036', 'TSH-PL180513040', 'TSH-PL180513045', 'TSH-PL180513055', 'TSH-
PL180513056', 'TSH-PL180513057', 'TSH-PL180513067', 'TSH-PL180513069', 'TSH-PL180513079', 'TSH
-PL180513084', 'TSH-PL180513087', 'TSH-PL180513092', 'TSH-PL180513106', 'TSH-PL180516001', 'TS
H-PL180516002', 'TSH-PL180516004', 'TSH-PL180516016', 'TSH-PL180516023', 'TSH-PL180516032', 'T
SH-PL180516055', 'TSH-PL180516056', 'TSH-PL180516062', 'TSH-PL180123002', 'TSH-PL180123012', '
TSH-PL180513010', 'TSH-PL180513019', 'TSH-PL180513022', 'TSH-PL180513031', 'TSH-PL180513035',
'TSH-PL180513044', 'TSH-PL180513086', 'TSH-PL180513112', 'TSH-PL180516029', 'TSH-PL180516034',
'TSH-PL180516046', 'TSH-PL180516048', 'TSH-PL180516058', 'TSH-PL180516060']
pre_diagnosis_samples_stomach = ['TSH-PL180123017', 'TSH-PL180123018', 'TSH-PL180123027', 'TSH
-PL180123033', 'TSH-PL180513025', 'TSH-PL180513038', 'TSH-PL180513039', 'TSH-PL180513050', 'TS
H-PL180513060', 'TSH-PL180513066', 'TSH-PL180513070', 'TSH-PL180513073', 'TSH-PL180513076', 'T
SH-PL180513089', 'TSH-PL180513099', 'TSH-PL180513102', 'TSH-PL180513104', 'TSH-PL180513119', '
TSH-PL180513123', 'TSH-PL180513127', 'TSH-PL180516007', 'TSH-PL180516008', 'TSH-PL180516009',
'TSH-PL180516019', 'TSH-PL180516027', 'TSH-PL180516036', 'TSH-PL180516042', 'TSH-PL180516043',
'TSH-PL180513003', 'TSH-PL180513018', 'TSH-PL180513103', 'TSH-PL180513105', 'TSH-PL180513125',
'TSH-PL180516010', 'TSH-PL180516052']
```

BioChain Tissue Samples

In [7]:
```
healthy_lung_tissue = ['BC_Lung_ND1', 'BC_Lung_ND2', 'BC_Lung_ND3', 'BC_Lung_ND4', 'BC_Lung_ND
5', 'BC_Lung_ND6', 'BC_Lung_ND7', 'BC_Lung_ND8']
healthy_colon_tissue = ['BC_CRC_ND1', 'BC_CRC_ND2', 'BC_CRC_ND3', 'BC_CRC_ND4', 'BC_CRC_ND5',
'BC_CRC_ND6', 'BC_CRC_ND7', 'BC_CRC_ND8']
healthy_stomach_tissue = ['BC_Stomach_ND1', 'BC_Stomach_ND2', 'BC_Stomach_ND3', 'BC_Stomach_ND
4', 'BC_Stomach_ND5', 'BC_Stomach_ND6', 'BC_Stomach_ND7', 'BC_Stomach_ND8']
healthy_breast_tissue = ['BC_Breast_ND1', 'BC_Breast_ND2', 'BC_Breast_ND3', 'BC_Breast_ND4', '
BC_Breast_ND5', 'BC_Breast_ND6', 'BC_Breast_ND7', 'BC_Breast_ND8']

cancer_lung_tissue = ['BC_Lung_TD1', 'BC_Lung_TD10', 'BC_Lung_TD11', 'BC_Lung_TD12', 'BC_Lung_
TD13', 'BC_Lung_TD14', 'BC_Lung_TD15', 'BC_Lung_TD16', 'BC_Lung_TD17', 'BC_Lung_TD18', 'BC_Lun
g_TD19', 'BC_Lung_TD2', 'BC_Lung_TD20', 'BC_Lung_TD21', 'BC_Lung_TD22', 'BC_Lung_TD23', 'BC_Lu
ng_TD24', 'BC_Lung_TD25', 'BC_Lung_TD26', 'BC_Lung_TD27', 'BC_Lung_TD28', 'BC_Lung_TD29', 'BC_
Lung_TD3', 'BC_Lung_TD30', 'BC_Lung_TD31', 'BC_Lung_TD32', 'BC_Lung_TD33', 'BC_Lung_TD34', 'BC
_Lung_TD35', 'BC_Lung_TD36', 'BC_Lung_TD37', 'BC_Lung_TD38', 'BC_Lung_TD39', 'BC_Lung_TD4', 'B
C_Lung_TD40', 'BC_Lung_TD5', 'BC_Lung_TD6', 'BC_Lung_TD7', 'BC_Lung_TD8', 'BC_Lung_TD9']
cancer_colon_tissue = ['BC_CRC_TD1', 'BC_CRC_TD10', 'BC_CRC_TD11', 'BC_CRC_TD12', 'BC_CRC_TD13
', 'BC_CRC_TD14', 'BC_CRC_TD15', 'BC_CRC_TD16', 'BC_CRC_TD17', 'BC_CRC_TD18', 'BC_CRC_TD19', '
BC_CRC_TD2', 'BC_CRC_TD20', 'BC_CRC_TD21', 'BC_CRC_TD22', 'BC_CRC_TD23', 'BC_CRC_TD24', 'BC_CR
C_TD25', 'BC_CRC_TD26', 'BC_CRC_TD27', 'BC_CRC_TD28', 'BC_CRC_TD29', 'BC_CRC_TD3', 'BC_CRC_TD3
0', 'BC_CRC_TD31', 'BC_CRC_TD32', 'BC_CRC_TD33', 'BC_CRC_TD34', 'BC_CRC_TD35', 'BC_CRC_TD36',
'BC_CRC_TD37', 'BC_CRC_TD38', 'BC_CRC_TD39', 'BC_CRC_TD4', 'BC_CRC_TD40', 'BC_CRC_TD5', 'BC_CR
C_TD6', 'BC_CRC_TD7', 'BC_CRC_TD8', 'BC_CRC_TD9']
cancer_stomach_tissue = ['BC_Stomach_TD1', 'BC_Stomach_TD10', 'BC_Stomach_TD11', 'BC_Stomach_T
D12', 'BC_Stomach_TD13', 'BC_Stomach_TD14', 'BC_Stomach_TD15', 'BC_Stomach_TD16', 'BC_Stomach_
TD17', 'BC_Stomach_TD18', 'BC_Stomach_TD19', 'BC_Stomach_TD2', 'BC_Stomach_TD20', 'BC_Stomach_
TD21', 'BC_Stomach_TD22', 'BC_Stomach_TD23', 'BC_Stomach_TD24', 'BC_Stomach_TD25', 'BC_Stomach
_TD26', 'BC_Stomach_TD27', 'BC_Stomach_TD28', 'BC_Stomach_TD29', 'BC_Stomach_TD3', 'BC_Stomach
_TD30', 'BC_Stomach_TD31', 'BC_Stomach_TD32', 'BC_Stomach_TD33', 'BC_Stomach_TD34', 'BC_Stomac
h_TD35', 'BC_Stomach_TD36', 'BC_Stomach_TD37', 'BC_Stomach_TD38', 'BC_Stomach_TD39', 'BC_Stoma
ch_TD4', 'BC_Stomach_TD40', 'BC_Stomach_TD5', 'BC_Stomach_TD6', 'BC_Stomach_TD7', 'BC_Stomach_
TD8', 'BC_Stomach_TD9']
cancer_breast_tissue = ['BC_Breast_TD1', 'BC_Breast_TD10', 'BC_Breast_TD11', 'BC_Breast_TD12',
'BC_Breast_TD13', 'BC_Breast_TD14', 'BC_Breast_TD15', 'BC_Breast_TD16', 'BC_Breast_TD17', 'BC_
Breast_TD18', 'BC_Breast_TD19', 'BC_Breast_TD2', 'BC_Breast_TD20', 'BC_Breast_TD21', 'BC_Breas
t_TD22', 'BC_Breast_TD23', 'BC_Breast_TD24', 'BC_Breast_TD25', 'BC_Breast_TD26', 'BC_Breast_TD
27', 'BC_Breast_TD28', 'BC_Breast_TD29', 'BC_Breast_TD3', 'BC_Breast_TD30', 'BC_Breast_TD31',
'BC_Breast_TD32', 'BC_Breast_TD33', 'BC_Breast_TD34', 'BC_Breast_TD35', 'BC_Breast_TD36', 'BC_
Breast_TD37', 'BC_Breast_TD38', 'BC_Breast_TD39', 'BC_Breast_TD4', 'BC_Breast_TD40', 'BC_Breas
t_TD5', 'BC_Breast_TD6', 'BC_Breast_TD7', 'BC_Breast_TD8', 'BC_Breast_TD9']

all_tissue_samples = healthy_lung_tissue+healthy_colon_tissue+healthy_stomach_tissue+healthy_b
reast_tissue+cancer_lung_tissue+cancer_colon_tissue+cancer_stomach_tissue+cancer_breast_tissue
```

Load Data

Let's load a data matrix listing the average methylation across each target region for each sample.

In [8]:
```
amf_df_orig = pd.read_csv('SupplementaryData5.tsv', sep="\t", header=0, index_col=0)
```

Marker Selection

Let's first remove markers that are not covered in all plasma samples, so we don't have to deal with any missing values.

In [9]:
```
amf_df = amf_df_orig.copy()
amf_df = amf_df.loc[amf_df[healthy_samples+post_diagnosis_samples+pre_diagnosis_samples].dropn
a(how='any').index]
```

Next, let's choose markers that separate healthy tissue from cancer tissue.

For each tissue type we purchased from BioChain, we will use a T-test to identify target regions that separate normal tissue from cancer tissue (applying a Benjamini-Hochberg multiple testing correction).

In [10]:
```
tissue_t_tests = pd.concat(
    [
        pd.Series(amf_df.index),
        pd.Series(scipy.stats.ttest_ind(amf_df.loc[:, healthy_colon_tissue].T,
                                        amf_df.loc[:, cancer_colon_tissue].T,
                                        axis=0, equal_var=True, nan_policy='omit').pvalue),
        pd.Series(scipy.stats.ttest_ind(amf_df.loc[:, healthy_lung_tissue].T,
                                        amf_df.loc[:, cancer_lung_tissue].T,
                                        axis=0, equal_var=True, nan_policy='omit').pvalue),
        pd.Series(scipy.stats.ttest_ind(amf_df.loc[:, healthy_stomach_tissue].T,
                                        amf_df.loc[:, cancer_stomach_tissue].T,
                                        axis=0, equal_var=True, nan_policy='omit').pvalue),
        pd.Series(scipy.stats.ttest_ind(amf_df.loc[:, healthy_breast_tissue].T,
                                        amf_df.loc[:, cancer_breast_tissue].T,
                                        axis=0, equal_var=True, nan_policy='omit').pvalue),
    ], axis=1
)

tissue_t_tests.columns = ['Locus',
                          'ColonM', 'LungM', 'StomachM', 'BreastM',
                         ]
tissue_t_tests = tissue_t_tests.set_index('Locus')

# Benjamini-Hochberg Correction
from statsmodels.stats.multitest import multipletests
_, pvals_corrected, _, _ = multipletests(tissue_t_tests.min(axis=1).fillna(1).values, alpha=0.
05, method='fdr_bh')

tissue_t_tests_min_by_marker = tissue_t_tests.loc[pvals_corrected <= 0.05].iloc[:, :4].min(axi
s=1).copy()
```

In [11]:
```
print len(tissue_t_tests_min_by_marker)
```

```
477
```

We can then use these target regions in our plasma samples to build a classifier.

Divide Samples Into Leave-In and Leave-Out

Let's first split the Taizhou samples into two sets: leave-in (1/2) and leave-out (1/2). The leave-in set will be used to build a model.

We want to make sure that equal proportions of healthy/cancer samples are included in the leave-in/leave-out set. We also want balance within each type of cancer (so we don't end up with no samples for one cancer type in the leave-out set, for example).

In [12]:
```
random.seed(128)

# Healthy
random.shuffle(healthy_samples)
# Post-Diagnosis
random.shuffle(post_diagnosis_samples_colon)
random.shuffle(post_diagnosis_samples_liver)
random.shuffle(post_diagnosis_samples_esophagus)
random.shuffle(post_diagnosis_samples_lung)
random.shuffle(post_diagnosis_samples_stomach)
# Pre-Diagnosis
random.shuffle(pre_diagnosis_samples_colon)
random.shuffle(pre_diagnosis_samples_liver)
random.shuffle(pre_diagnosis_samples_esophagus)
random.shuffle(pre_diagnosis_samples_lung)
random.shuffle(pre_diagnosis_samples_stomach)

negative_leavein = list(healthy_samples[:int(len(healthy_samples)/2)])
negative_leaveout = list(healthy_samples[int(len(healthy_samples)/2):])

positive_post_leavein = post_diagnosis_samples_colon[:int(len(post_diagnosis_samples_colon)/2)
] + post_diagnosis_samples_liver[:int(len(post_diagnosis_samples_liver)/2)] + post_diagnosis_s
amples_esophagus[:int(len(post_diagnosis_samples_esophagus)/2)] + post_diagnosis_samples_lung[
:int(len(post_diagnosis_samples_lung)/2)] + post_diagnosis_samples_stomach[:int(len(post_diagn
osis_samples_stomach)/2)]
positive_post_leaveout = post_diagnosis_samples_colon[int(len(post_diagnosis_samples_colon)/2)
:] + post_diagnosis_samples_liver[int(len(post_diagnosis_samples_liver)/2):] + post_diagnosis_
samples_esophagus[int(len(post_diagnosis_samples_esophagus)/2):] + post_diagnosis_samples_lung
[int(len(post_diagnosis_samples_lung)/2):] + post_diagnosis_samples_stomach[int(len(post_diagn
osis_samples_stomach)/2):]

positive_pre_leavein = pre_diagnosis_samples_colon[:int(len(pre_diagnosis_samples_colon)/2)] +
pre_diagnosis_samples_liver[:int(len(pre_diagnosis_samples_liver)/2)] + pre_diagnosis_samples_
esophagus[:int(len(pre_diagnosis_samples_esophagus)/2)] + pre_diagnosis_samples_lung[:int(len(
pre_diagnosis_samples_lung)/2)] + pre_diagnosis_samples_stomach[:int(len(pre_diagnosis_samples
_stomach)/2)]
positive_pre_leaveout = pre_diagnosis_samples_colon[int(len(pre_diagnosis_samples_colon)/2):]
+ pre_diagnosis_samples_liver[int(len(pre_diagnosis_samples_liver)/2):] + pre_diagnosis_sample
s_esophagus[int(len(pre_diagnosis_samples_esophagus)/2):] + pre_diagnosis_samples_lung[int(len
(pre_diagnosis_samples_lung)/2):] + pre_diagnosis_samples_stomach[int(len(pre_diagnosis_sample
s_stomach)/2):]
```

Build a Linear Discriminant Analysis Classifier

Now, let's build an LDA classifier from the leave-in set. We will use the method described in Teschendorff et al, Nature Materials (2019). Briefly:

1. We will randomly split the leave-in set into 50% training, 50% test.
2. We will train an LDA classifier on the training set, and compute the results on the test set.
3. We will repeat steps 1 and 2 using 1000 different random splits.
4. We will then compute the ensemble performance of these 1000 models across the ten separate test sets, and report this as the "leave-in set" accuracy.
5. We will then apply all 1000 models to the "leave-out set," compute the ensemble performance, and report this as the "leave-out set" accuracy.

Let's build each LDA classifier using 1000 different random splits:

In [13]:
```
negative_test_list = []
positive_post_test_list = []
positive_pre_test_list = []
z_prob_list = []

for random_state in range(1000):
    random.seed(random_state)

    # Set up LR classifier that will automatically fit the best C value using the training set
    clf = LinearDiscriminantAnalysis()

    # Split the leave-in set into training and test set
    negative = list(negative_leavein)
    positive_post = list(positive_post_leavein)
    positive_pre = list(positive_pre_leavein)
    random.shuffle(negative)
    random.shuffle(positive_post)
    random.shuffle(positive_pre)
    negative_training, negative_test = negative[:1*int(len(negative)/2)], negative[int(1*len(n
egative)/2):]
    positive_post_training, positive_post_test = positive_post[:1*int(len(positive_post)/2)],
positive_post[int(1*len(positive_post)/2):]
    positive_pre_training, positive_pre_test = positive_pre[:1*int(len(positive_pre)/2)], posi
tive_pre[int(1*len(positive_pre)/2):]
    positive_training = positive_post_training+positive_pre_training
    positive_test = positive_post_test+positive_pre_test

    # Build the model and set parameters using the training set
    x = amf_df.loc[tissue_t_tests_min_by_marker.index, negative_training+positive_training].tr
anspose()
    y = ['Healthy']*len(negative_training) + ['Cancer']*len(positive_training)
    with warnings.catch_warnings():
        warnings.simplefilter("ignore")
        clf.fit(x,y)

    # Compute the accuracy of this classifier on the leave-in and leave-out sets
    z = pd.Series(clf.predict(amf_df.loc[tissue_t_tests_min_by_marker.index, healthy_samples+p
ost_diagnosis_samples+pre_diagnosis_samples].transpose()),
                  index=healthy_samples+post_diagnosis_samples+pre_diagnosis_samples)
    z_prob = pd.Series(clf.predict_proba(amf_df.loc[tissue_t_tests_min_by_marker.index, health
y_samples+post_diagnosis_samples+pre_diagnosis_samples].transpose())[:, 0],
                       index=healthy_samples+post_diagnosis_samples+pre_diagnosis_samples)

    # Store the results
    negative_test_list.append(negative_test)
    positive_post_test_list.append(positive_post_test)
    positive_pre_test_list.append(positive_pre_test)
    z_prob_list.append(z_prob)
```

Next, let's compute the ensemble performance across the leave-in test sets and the leave-out sets.

In [14]:
```
ensemble_score_leavein = pd.concat([z_prob_list[index].loc[negative_test_list[index]+positive_
pre_test_list[index]+positive_post_test_list[index]]
                                    for index in range(1000)],
                                   axis=1, sort=True).mean(axis=1)
ensemble_score_leaveout = pd.concat([z_prob_list[index].loc[negative_leaveout+positive_post_le
aveout+positive_pre_leaveout]
                                     for index in range(1000)],
                                    axis=1, sort=True).mean(axis=1)
```

We can now compute accuracy metrics for the two sets; as per Teschendorff et al 2019, we will choose the model cutoff using the leave-in set.

Leave-In Set Accuracy

Let's choose a cutoff that aims for ~95% specificity in the leave-in set, and compute the associated sensitivity.

In [15]:
```
##### sns.set_style('whitegrid')

y_true = ['Healthy']*len(negative_leavein) + ['Cancer']*len(positive_post_leavein)
y_score = ensemble_score_leavein.loc[negative_leavein+positive_post_leavein]
cur_fpr, cur_tpr, cur_cutoff = roc_curve(y_true, y_score, pos_label='Cancer')
cur_auc = auc(cur_fpr, cur_tpr)
plt.plot(cur_fpr, cur_tpr, label=r'Post-Diagnosis ROC (AUC = %0.2f)' % cur_auc)

y_true = ['Healthy']*len(negative_leavein) + ['Cancer']*len(positive_pre_leavein)
y_score = ensemble_score_leavein.loc[negative_leavein+positive_pre_leavein]
cur_fpr, cur_tpr, cur_cutoff = roc_curve(y_true, y_score, pos_label='Cancer')
cur_auc = auc(cur_fpr, cur_tpr)
plt.plot(cur_fpr, cur_tpr, label=r'Pre-Diagnosis ROC (AUC = %0.2f)' % cur_auc)

plt.xlim([-0.0, 1.0])
plt.ylim([-0.0, 1.0])
plt.xticks([0, 0.05, 0.1, 0.2, 0.3, 0.4, 0.5, 0.6, 0.7, 0.8, 0.9, 1.0])
plt.yticks([0, 0.05, 0.1, 0.2, 0.3, 0.4, 0.5, 0.6, 0.7, 0.8, 0.9, 1.0])
plt.xlabel('False Positive Rate')
plt.ylabel('True Positive Rate')
plt.legend(loc="lower right");

print "Leave-In Results"
print "Specificity: %s" % (sum(ensemble_score_leavein.loc[negative_leavein] < 0.654) / len(neg
ative_leavein))
print "Post-Diagnosis Sensitivity: %s" % (sum(ensemble_score_leavein.loc[positive_post_leavein
] >= 0.654) / len(positive_post_leavein))
print "Pre-Diagnosis Sensitivity: %s" % (sum(ensemble_score_leavein.loc[positive_pre_leavein]
>= 0.654) / len(positive_pre_leavein))

plt.figure()
sns.distplot(ensemble_score_leavein.loc[negative_leavein])
sns.distplot(ensemble_score_leavein.loc[positive_post_leavein])
sns.distplot(ensemble_score_leavein.loc[positive_pre_leavein])
plt.legend(['Healthy', 'Post-Diagnosis', 'Pre-Diagnosis']);
plt.xlim(0, 1)
```

```
Leave-In Results
Specificity: 0.946859903382
Post-Diagnosis Sensitivity: 0.890909090909
Pre-Diagnosis Sensitivity: 0.956989247312
```

Out[15]: (0, 1)

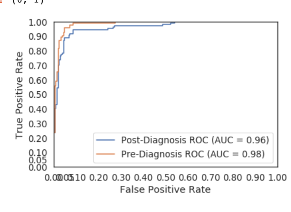

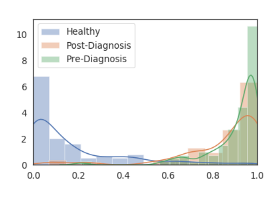

Leave-Out Set Accuracy

Let's compute the leave-out set sensitivity and specificity using the previously chosen cutoff.

In [16]:
```
sns.set_style('whitegrid')

y_true = ['Healthy']*len(negative_leaveout) + ['Cancer']*len(positive_post_leaveout)
y_score = ensemble_score_leaveout.loc[negative_leaveout+positive_post_leaveout]
cur_fpr, cur_tpr, cur_cutoff = roc_curve(y_true, y_score, pos_label='Cancer')
cur_auc = auc(cur_fpr, cur_tpr)
plt.plot(cur_fpr, cur_tpr, label=r'Post-Diagnosis ROC (AUC = %0.2f)' % cur_auc)

y_true = ['Healthy']*len(negative_leaveout) + ['Cancer']*len(positive_pre_leaveout)
y_score = ensemble_score_leaveout.loc[negative_leaveout+positive_pre_leaveout]
cur_fpr, cur_tpr, cur_cutoff = roc_curve(y_true, y_score, pos_label='Cancer')
cur_auc = auc(cur_fpr, cur_tpr)
plt.plot(cur_fpr, cur_tpr, label=r'Pre-Diagnosis ROC (AUC = %0.2f)' % cur_auc)

plt.xlim([-0.0, 1.0])
plt.ylim([-0.0, 1.0])
plt.xticks([0, 0.05, 0.1, 0.2, 0.3, 0.4, 0.5, 0.6, 0.7, 0.8, 0.9, 1.0])
plt.yticks([0, 0.05, 0.1, 0.2, 0.3, 0.4, 0.5, 0.6, 0.7, 0.8, 0.9, 1.0])
plt.xlabel('False Positive Rate')
plt.ylabel('True Positive Rate')
plt.legend(loc="lower right");

print "Leave-Out Results"
print "Specificity: %s" % (sum(ensemble_score_leaveout.loc[negative_leaveout] < 0.654) / len(n
egative_leaveout))
print "Post-Diagnosis Sensitivity: %s" % (sum(ensemble_score_leaveout.loc[positive_post_leaveo
ut] >= 0.654) / len(positive_post_leaveout))
print "Pre-Diagnosis Sensitivity: %s" % (sum(ensemble_score_leaveout.loc[positive_pre_leaveout
] >= 0.654) / len(positive_pre_leaveout))

plt.figure()
sns.distplot(ensemble_score_leaveout.loc[negative_leaveout])
sns.distplot(ensemble_score_leaveout.loc[positive_post_leaveout])
sns.distplot(ensemble_score_leaveout.loc[positive_pre_leaveout])
plt.legend(['Healthy', 'Post-Diagnosis', 'Pre-Diagnosis']);
plt.xlim(0, 1)
```

```
Leave-Out Results
Specificity: 0.946859903382
Post-Diagnosis Sensitivity: 0.867256637168
Pre-Diagnosis Sensitivity: 0.969387755102
```

Out[16]: (0, 1)

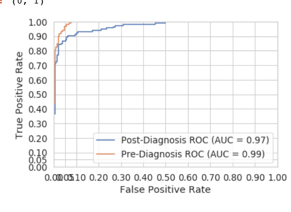

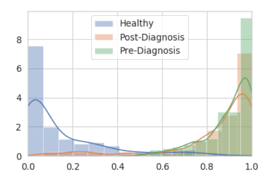

As per Teschendorff et al 2019, the accuracy is similar for the leave-in and leave-out data sets.

Output Data Table

Let's compute the final merged score for each sample by taking the average across all 1000 cross-validations:

In [17]:
```
all_scores_leavein = pd.concat([z_prob_list[index].loc[negative_test_list[index]+positive_pre_
test_list[index]+positive_post_test_list[index]]
                                for index in range(1000)],
                               axis=1, sort=True).fillna('Training')
all_scores_leavein['Category'] = ['Leave-In']*all_scores_leavein.shape[0]

all_scores_leaveout = pd.concat([z_prob_list[index].loc[negative_leaveout+positive_post_leaveo
ut+positive_pre_leaveout]
                                 for index in range(1000)],
                                axis=1, sort=True)
all_scores_leaveout['Category'] = ['Leave-Out']*all_scores_leaveout.shape[0]

all_scores_merged = pd.concat([all_scores_leavein, all_scores_leaveout], axis=0).replace('Trai
ning', np.nan).mean(axis=1)
```

In [18]:
```
pd.DataFrame(pd.concat([all_scores_leavein, all_scores_leaveout], axis=0).replace('Training',
np.nan).mean(axis=1)).to_csv('tsh_analysis_20181029_final_score_table_lda.tsv')
```

```
np.nan).mean(axis=1)).to_csv('tsh_analysis_20191029_final_score_table_lda.tsv')
```

Supplementary Note 3.
Code utilized to derive and implement an alternate Logistic Regression classifier that only compares healthy and pre-diagnosis samples. A leave-in set of samples was used to build the classifier, and a leave-out set of samples was utilized to validate the classifier.

Supplementary Note 3

This analysis is similar to that of Supplementary Note 1; however, in this case all post-diagnosis samples were excluded.

The leave-out set sensitivity and specificity were the same as in Supplementary Note 1.

In [1]:
```
from __future__ import division
import sys
import os
import glob
import random
```

In [2]:
```
import pandas as pd
import numpy as np
import scipy
from sklearn.linear_model import LogisticRegression, LogisticRegressionCV
from sklearn.metrics import roc_curve, auc
from statsmodels.stats.multitest import multipletests
```

In [3]:
```
%matplotlib inline
import seaborn as sns
import matplotlib.pyplot as plt
```

In [4]:
```
sns.set()
sns.set_context('notebook', font_scale=1.2)
sns.set_style('white')
```

Sample Info

For this study, we processed two sets of samples; one set of plasma samples from the Taizhou Longitudinal Study, and a second set of commercially purchsed normal and cancer tissue samples from BioChain.

Taizhou Longitudinal Study

In [5]:
```
pre_diagnosis_samples = ['TSH-PL180123001', 'TSH-PL180123006', 'TSH-PL180123007', 'TSH-PL180123008', 'TSH-PL180123010', 'TSH-PL180123013', 'TSH-PL180123014', 'TSH-PL180123016', 'TSH-PL180123017', 'TSH-PL180123018', 'TSH-PL180123021', 'TSH-PL180123022', 'TSH-PL180123025', 'TSH-PL180123027', 'TSH-PL180123029', 'TSH-PL180123030', 'TSH-PL180123031', 'TSH-PL180123033', 'TSH-PL180513004', 'TSH-PL180513005', 'TSH-PL180513006', 'TSH-PL180513007', 'TSH-PL180513011', 'TSH-PL180513012', 'TSH-PL180513013', 'TSH-PL180513014', 'TSH-PL180513016', 'TSH-PL180513017', 'TSH-PL180513020', 'TSH-PL180513021', 'TSH-PL180513023', 'TSH-PL180513024', 'TSH-PL180513025', 'TSH-PL180513026', 'TSH-PL180513027', 'TSH-PL180513029', 'TSH-PL180513030', 'TSH-PL180513034', 'TSH-PL180513036', 'TSH-PL180513037', 'TSH-PL180513038', 'TSH-PL180513039', 'TSH-PL180513040', 'TSH-PL180513041', 'TSH-PL180513043', 'TSH-PL180513045', 'TSH-PL180513049', 'TSH-PL180513050', 'TSH-PL180513051', 'TSH-PL180513055', 'TSH-PL180513056', 'TSH-PL180513057', 'TSH-PL180513060', 'TSH-PL180513062', 'TSH-PL180513066', 'TSH-PL180513067', 'TSH-PL180513068', 'TSH-PL180513069', 'TSH-PL180513070', 'TSH-PL180513071', 'TSH-PL180513072', 'TSH-PL180513073', 'TSH-PL180513075',
'TSH-PL180513076', 'TSH-PL180513077', 'TSH-PL180513078', 'TSH-PL180513079', 'TSH-PL180513080',
'TSH-PL180513081', 'TSH-PL180513084', 'TSH-PL180513085', 'TSH-PL180513087', 'TSH-PL180513088',
'TSH-PL180513089', 'TSH-PL180513091', 'TSH-PL180513092', 'TSH-PL180513093', 'TSH-PL180513095',
'TSH-PL180513097', 'TSH-PL180513098', 'TSH-PL180513099', 'TSH-PL180513100', 'TSH-PL180513101',
'TSH-PL180513102', 'TSH-PL180513104', 'TSH-PL180513106', 'TSH-PL180513108', 'TSH-PL180513109',
'TSH-PL180513110', 'TSH-PL180513111', 'TSH-PL180513114', 'TSH-PL180513115', 'TSH-PL180513117',
'TSH-PL180513118', 'TSH-PL180513119', 'TSH-PL180513121', 'TSH-PL180513122', 'TSH-PL180513123',
'TSH-PL180513124', 'TSH-PL180513127', 'TSH-PL180513128', 'TSH-PL180513129', 'TSH-PL180516001',
'TSH-PL180516002', 'TSH-PL180516003', 'TSH-PL180516004', 'TSH-PL180516006', 'TSH-PL180516007',
'TSH-PL180516008', 'TSH-PL180516009', 'TSH-PL180516011', 'TSH-PL180516012', 'TSH-PL180516013',
'TSH-PL180516014', 'TSH-PL180516016', 'TSH-PL180516018', 'TSH-PL180516019', 'TSH-PL180516020',
'TSH-PL180516021', 'TSH-PL180516023', 'TSH-PL180516027', 'TSH-PL180516028', 'TSH-PL180516030',
'TSH-PL180516031', 'TSH-PL180516032', 'TSH-PL180516036', 'TSH-PL180516038', 'TSH-PL180516039',
'TSH-PL180516040', 'TSH-PL180516042', 'TSH-PL180516043', 'TSH-PL180516044', 'TSH-PL180516045',
'TSH-PL180516049', 'TSH-PL180516051', 'TSH-PL180516053', 'TSH-PL180516054', 'TSH-PL180516055',
'TSH-PL180516056', 'TSH-PL180516057', 'TSH-PL180516059', 'TSH-PL180516062', 'TSH-PL180516063',
'TSH-PL180123002', 'TSH-PL180123004', 'TSH-PL180123005', 'TSH-PL180123012', 'TSH-PL180123024',
'TSH-PL180123032', 'TSH-PL180513001', 'TSH-PL180513002', 'TSH-PL180513003', 'TSH-PL180513009',
'TSH-PL180513010', 'TSH-PL180513018', 'TSH-PL180513019', 'TSH-PL180513022', 'TSH-PL180513028',
'TSH-PL180513031', 'TSH-PL180513032', 'TSH-PL180513033', 'TSH-PL180513035', 'TSH-PL180513044',
'TSH-PL180513058', 'TSH-PL180513064', 'TSH-PL180513083', 'TSH-PL180513086', 'TSH-PL180513090',
'TSH-PL180513096', 'TSH-PL180513103', 'TSH-PL180513105', 'TSH-PL180513112', 'TSH-PL180513116',
'TSH-PL180513125', 'TSH-PL180516005', 'TSH-PL180516010', 'TSH-PL180516015', 'TSH-PL180516017',
'TSH-PL180516025', 'TSH-PL180516026', 'TSH-PL180516029', 'TSH-PL180516033', 'TSH-PL180516034',
'TSH-PL180516046', 'TSH-PL180516047', 'TSH-PL180516048', 'TSH-PL180516050', 'TSH-PL180516052',
'TSH-PL180516058', 'TSH-PL180516060', 'TSH-PL180516061']
post_diagnosis_samples = ['TSH-PL171221101', 'TSH-PL171221112', 'TSH-PL171221149', 'TSH-PL171221150', 'TSH-PL171221160', 'TSH-PL171221172', 'TSH-PL171221179', 'TSH-PL171221180', 'TSH-PL180504001', 'TSH-PL180504002', 'TSH-PL180504003', 'TSH-PL180504004', 'TSH-PL180504005', 'TSH-PL180504009', 'TSH-PL180504010', 'TSH-PL180504011', 'TSH-PL180504013', 'TSH-PL180504014', 'TSH-PL180504015', 'TSH-PL180504016', 'TSH-PL180504017', 'TSH-PL180504018', 'TSH-PL180504019', 'TSH-PL180504021', 'TSH-PL180504022', 'TSH-PL180504027', 'TSH-PL180504028', 'TSH-PL180504030', 'TSH-PL180504033', 'TSH-PL180504034', 'TSH-PL180504041', 'TSH-PL180504042', 'TSH-PL180504044', 'TSH-PL180504046', 'TSH-PL180504047', 'TSH-PL180504049', 'TSH-PL180504050', 'TSH-PL180504053', 'TSH-PL180504054', 'TSH-PL180504058', 'TSH-PL180504061', 'TSH-PL180504062', 'TSH-PL180504063', 'TSH-PL180504065', 'TSH-PL180504068', 'TSH-PL180504070', 'TSH-PL180504074', 'TSH-PL180504081', 'TSH-PL180504083', 'TSH-PL180504087', 'TSH-PL180504088', 'TSH-PL180504089', 'TSH-PL180504091', 'TSH-PL180504094', 'TSH-PL180504097', 'TSH-PL180504101', 'TSH-PL180504104', 'TSH-PL180504108',
'TSH-PL180504111', 'TSH-PL180504118', 'TSH-PL180508001', 'TSH-PL180508004', 'TSH-PL180508005',
'TSH-PL180508006', 'TSH-PL180508008', 'TSH-PL180508009', 'TSH-PL180508011', 'TSH-PL180508012',
'TSH-PL180508013', 'TSH-PL180508014', 'TSH-PL180508015', 'TSH-PL180508019', 'TSH-PL180508020',
'TSH-PL180508021', 'TSH-PL180508022', 'TSH-PL180508025', 'TSH-PL180508030', 'TSH-PL180508031',
'TSH-PL180508033', 'TSH-PL180508034', 'TSH-PL180508036', 'TSH-PL180508037', 'TSH-PL180508038',
'TSH-PL180508039', 'TSH-PL180508040', 'TSH-PL180508041', 'TSH-PL180508042', 'TSH-PL180508043',
'TSH-PL180508049', 'TSH-PL180508050', 'TSH-PL180508060', 'TSH-PL180508061', 'TSH-PL180508064',
'TSH-PL180508068', 'TSH-PL180508076', 'TSH-PL180508078', 'TSH-PL180508083', 'TSH-PL180508086',
'TSH-PL180508089', 'TSH-PL180510005', 'TSH-PL180510006', 'TSH-PL180510007', 'TSH-PL180510008',
'TSH-PL180510010', 'TSH-PL180510011', 'TSH-PL180510012', 'TSH-PL180510014', 'TSH-PL180510015',
'TSH-PL180510016', 'TSH-PL180510017', 'TSH-PL180510018', 'TSH-PL180510019', 'TSH-PL180510021',
'TSH-PL180510023', 'TSH-PL180510024', 'TSH-PL180510025', 'TSH-PL180510026', 'TSH-PL180510030',
'TSH-PL180510031', 'TSH-PL180510033', 'TSH-PL180510038', 'TSH-PL180510040', 'TSH-PL180510044',
'TSH-PL180510047', 'TSH-PL180510050', 'TSH-PL180510052', 'TSH-PL180510053', 'TSH-PL180510054',
'TSH-PL180510055', 'TSH-PL180510057', 'TSH-PL180510061', 'TSH-PL180510063', 'TSH-PL180510066',
'TSH-PL180510071', 'TSH-PL180510072', 'TSH-PL180510073', 'TSH-PL180510074', 'TSH-PL180510075',
'TSH-PL180510076', 'TSH-PL180510077', 'TSH-PL180510078', 'TSH-PL180510079', 'TSH-PL180510080',
'TSH-PL180510085', 'TSH-PL180510089', 'TSH-PL180510092', 'TSH-PL180510095', 'TSH-PL180510101',
'TSH-PL180510104', 'TSH-PL180510105', 'TSH-PL180510107', 'TSH-PL180510112', 'TSH-PL180510121',
'TSH-PL180510122', 'TSH-PL180510125', 'TSH-PL180510139', 'TSH-PL180510140', 'TSH-PL180510146',
'TSH-PL180510147', 'TSH-PL180510162', 'TSH-PL181205168', 'TSH-PL181205170', 'TSH-PL181205173',
'TSH-PL181205175', 'TSH-PL181205182', 'TSH-PL171221151', 'TSH-PL180504006', 'TSH-PL180504012',
'TSH-PL180504024', 'TSH-PL180504026', 'TSH-PL180504029', 'TSH-PL180504037', 'TSH-PL180504043',
'TSH-PL180504045', 'TSH-PL180504051', 'TSH-PL180504052', 'TSH-PL180504055', 'TSH-PL180504057',
'TSH-PL180504064', 'TSH-PL180504076', 'TSH-PL180504084', 'TSH-PL180504110', 'TSH-PL180508003',
'TSH-PL180508007', 'TSH-PL180508023', 'TSH-PL180508026', 'TSH-PL180508028', 'TSH-PL180508029',
'TSH-PL180508032', 'TSH-PL180508044', 'TSH-PL180508052', 'TSH-PL180508065', 'TSH-PL180508067',
'TSH-PL180508088', 'TSH-PL180510002', 'TSH-PL180510004', 'TSH-PL180510013', 'TSH-PL180510020',
'TSH-PL180510022', 'TSH-PL180510032', 'TSH-PL180510034', 'TSH-PL180510035', 'TSH-PL180510043',
'TSH-PL180510048', 'TSH-PL180510068', 'TSH-PL180510088', 'TSH-PL180510090', 'TSH-PL180510098',
'TSH-PL180510100', 'TSH-PL180510103', 'TSH-PL180510108', 'TSH-PL180510109', 'TSH-PL180510113',
'TSH-PL180510117', 'TSH-PL180510124', 'TSH-PL180510143', 'TSH-PL181205169', 'TSH-PL181205172',
'TSH-PL181205174', 'TSH-PL181205177', 'TSH-PL181205179', 'TSH-PL171221153', 'TSH-PL171221113']
healthy_samples = ['TSH-PL180309087', 'TSH-PL180316003', 'TSH-PL180316005', 'TSH-PL180316016',
'TSH-PL180316017', 'TSH-PL180316018', 'TSH-PL180316022', 'TSH-PL180316024', 'TSH-PL180316027',
'TSH-PL180316033', 'TSH-PL180316037', 'TSH-PL180316038', 'TSH-PL180316039', 'TSH-PL180316049',
'TSH-PL180316061', 'TSH-PL180316065', 'TSH-PL180316079', 'TSH-PL180316092', 'TSH-PL180316095',
'TSH-PL180316099', 'TSH-PL180316101', 'TSH-PL180316106', 'TSH-PL180316115', 'TSH-PL180316119',
'TSH-PL180316126', 'TSH-PL180316129', 'TSH-PL180316157', 'TSH-PL180316161', 'TSH-PL180316163',
'TSH-PL180316164', 'TSH-PL180316173', 'TSH-PL180316174', 'TSH-PL180316181', 'TSH-PL180316183',
'TSH-PL180316187', 'TSH-PL180316190', 'TSH-PL180316195', 'TSH-PL180316196', 'TSH-PL180316198',
'TSH-PL180323034', 'TSH-PL180323038', 'TSH-PL180323051', 'TSH-PL180323052', 'TSH-PL180323054',
'TSH-PL180323059', 'TSH-PL180323065', 'TSH-PL180323067', 'TSH-PL180323072', 'TSH-PL180323073',
'TSH-PL180323091', 'TSH-PL180323106', 'TSH-PL180323107', 'TSH-PL180323109', 'TSH-PL180323110',
'TSH-PL180323111', 'TSH-PL180323119', 'TSH-PL180323121', 'TSH-PL180323124', 'TSH-PL180323139',
'TSH-PL180323141', 'TSH-PL180323142', 'TSH-PL180323151', 'TSH-PL180323155', 'TSH-PL180323166',
'TSH-PL180410003', 'TSH-PL180410023', 'TSH-PL180410043', 'TSH-PL180410049', 'TSH-PL180410059',
'TSH-PL180410063', 'TSH-PL180410076', 'TSH-PL180410087', 'TSH-PL180410115', 'TSH-PL180410116',
'TSH-PL180410133', 'TSH-PL180410140', 'TSH-PL180410144', 'TSH-PL180410160', 'TSH-PL180410162',
'TSH-PL180410198', 'TSH-PL180417003', 'TSH-PL180417007', 'TSH-PL180417035', 'TSH-PL180417060',
'TSH-PL180417074', 'TSH-PL180417087', 'TSH-PL180417095', 'TSH-PL180417101', 'TSH-PL180417109',
'TSH-PL180417117', 'TSH-PL180417135', 'TSH-PL180417138', 'TSH-PL180417143', 'TSH-PL180417152',
'TSH-PL180417156', 'TSH-PL180417165', 'TSH-PL180417170', 'TSH-PL180417179', 'TSH-PL180417180',
'TSH-PL180417181', 'TSH-PL180417200', 'TSH-PL180424003', 'TSH-PL180424030', 'TSH-PL180424036',
'TSH-PL180424058', 'TSH-PL180424090', 'TSH-PL180424097', 'TSH-PL180424104', 'TSH-PL180424115',
'TSH-PL180424146', 'TSH-PL180424149', 'TSH-PL180424155', 'TSH-PL180424162', 'TSH-PL180424166',
'TSH-PL180424171', 'TSH-PL180703037', 'TSH-PL180703136', 'TSH-PL180703153', 'TSH-PL180703157',
'TSH-PL180703185', 'TSH-PL180706034', 'TSH-PL180706037', 'TSH-PL180706095', 'TSH-PL180706096',
'TSH-PL180706104', 'TSH-PL180706113', 'TSH-PL180706118', 'TSH-PL180706145', 'TSH-PL180706147',
'TSH-PL180706150', 'TSH-PL180706153', 'TSH-PL180706156', 'TSH-PL181128001', 'TSH-PL181128003',
'TSH-PL181128005', 'TSH-PL181128007', 'TSH-PL181128008', 'TSH-PL181128009', 'TSH-PL181128011',
'TSH-PL181128012', 'TSH-PL181128014', 'TSH-PL181128015', 'TSH-PL181128019', 'TSH-PL181128021',
'TSH-PL181128022', 'TSH-PL181128023', 'TSH-PL181128025', 'TSH-PL181128027', 'TSH-PL181128029',
'TSH-PL181128030', 'TSH-PL181128033', 'TSH-PL181128035', 'TSH-PL181128036', 'TSH-PL181128038',
'TSH-PL181128039', 'TSH-PL181128040', 'TSH-PL181128041', 'TSH-PL181128042', 'TSH-PL181128043',
'TSH-PL181128045', 'TSH-PL181128047', 'TSH-PL181128048', 'TSH-PL181128049', 'TSH-PL181128050',
'TSH-PL181128051', 'TSH-PL181128054', 'TSH-PL181128055', 'TSH-PL181128056', 'TSH-PL181128057',
'TSH-PL181128058', 'TSH-PL181128061', 'TSH-PL181128062', 'TSH-PL181128064', 'TSH-PL181128065',
'TSH-PL181128066', 'TSH-PL181128070', 'TSH-PL181128073', 'TSH-PL181128077', 'TSH-PL181128080',
'TSH-PL181128082', 'TSH-PL181128083', 'TSH-PL181128085', 'TSH-PL181128086', 'TSH-PL181128093',
'TSH-PL181128096', 'TSH-PL181128099', 'TSH-PL181128101', 'TSH-PL181128102', 'TSH-PL181128103',
'TSH-PL181128104', 'TSH-PL181128107', 'TSH-PL181128109', 'TSH-PL181128110', 'TSH-PL181128111',
'TSH-PL181128112', 'TSH-PL181128114', 'TSH-PL181128115', 'TSH-PL181128117', 'TSH-PL181128118',
'TSH-PL181128120', 'TSH-PL181128122', 'TSH-PL181128123', 'TSH-PL181128124', 'TSH-PL181128125',
'TSH-PL181128126', 'TSH-PL181128127', 'TSH-PL181205002', 'TSH-PL181205003', 'TSH-PL181205005',
'TSH-PL181205006', 'TSH-PL181205008', 'TSH-PL181205009', 'TSH-PL181205013', 'TSH-PL181205014',
'TSH-PL181205016', 'TSH-PL181205020', 'TSH-PL181205022', 'TSH-PL181205024', 'TSH-PL181205026',
'TSH-PL181205027', 'TSH-PL181205028', 'TSH-PL181205030', 'TSH-PL181205033', 'TSH-PL181205035',
'TSH-PL181205040', 'TSH-PL181205041', 'TSH-PL181205042', 'TSH-PL181205043', 'TSH-PL181205044',
'TSH-PL181205045', 'TSH-PL181205046', 'TSH-PL181205047', 'TSH-PL181205048', 'TSH-PL181205049',
'TSH-PL181205051', 'TSH-PL181205053', 'TSH-PL181205054', 'TSH-PL181205055', 'TSH-PL181205057',
'TSH-PL181205058', 'TSH-PL181205059', 'TSH-PL181205060', 'TSH-PL181205061', 'TSH-PL181205062',
'TSH-PL181205063', 'TSH-PL181205064', 'TSH-PL181205065', 'TSH-PL181205066', 'TSH-PL181205068',
'TSH-PL181205069', 'TSH-PL181205072', 'TSH-PL181205074', 'TSH-PL181205075', 'TSH-PL181205076',
'TSH-PL181205077', 'TSH-PL181205078', 'TSH-PL181205079', 'TSH-PL181205081', 'TSH-PL181205082',
'TSH-PL181205083', 'TSH-PL181205085', 'TSH-PL181205086', 'TSH-PL181205087', 'TSH-PL181205088',
'TSH-PL181205090', 'TSH-PL181205091', 'TSH-PL181205093', 'TSH-PL181205095', 'TSH-PL181205096',
'TSH-PL181205097', 'TSH-PL181205098', 'TSH-PL181205099', 'TSH-PL181205102', 'TSH-PL181205103',
'TSH-PL181205104', 'TSH-PL181205107', 'TSH-PL181205108', 'TSH-PL181205110', 'TSH-PL181205113',
'TSH-PL181205115', 'TSH-PL181205116', 'TSH-PL181205117', 'TSH-PL181205121', 'TSH-PL181205122',
'TSH-PL181205125', 'TSH-PL181205128', 'TSH-PL181205129', 'TSH-PL181205140', 'TSH-PL181205141',
'TSH-PL181205142', 'TSH-PL181205143', 'TSH-PL181205144', 'TSH-PL181205145', 'TSH-PL181205146',
'TSH-PL181205147', 'TSH-PL181205148', 'TSH-PL181205149', 'TSH-PL181205150', 'TSH-PL181205151',
'TSH-PL181205152', 'TSH-PL181205155', 'TSH-PL181205156', 'TSH-PL181205157', 'TSH-PL181205158',
'TSH-PL181205160', 'TSH-PL181205161', 'TSH-PL181205162', 'TSH-PL181205164', 'TSH-PL181205165',
'TSH-PL181205166', 'TSH-PL180309082', 'TSH-PL180316013', 'TSH-PL180316029', 'TSH-PL180316075',
'TSH-PL180316102', 'TSH-PL180316112', 'TSH-PL180316134', 'TSH-PL180316138', 'TSH-PL180316175',
'TSH-PL180316178', 'TSH-PL180316200', 'TSH-PL180323029', 'TSH-PL180323032', 'TSH-PL180323053',
'TSH-PL180323068', 'TSH-PL180323074', 'TSH-PL180323088', 'TSH-PL180323097', 'TSH-PL180323103',
'TSH-PL180323115', 'TSH-PL180323120', 'TSH-PL180323160', 'TSH-PL180323175', 'TSH-PL180410058',
'TSH-PL180410070', 'TSH-PL180410081', 'TSH-PL180410148', 'TSH-PL180410161', 'TSH-PL180417017',
'TSH-PL180417023', 'TSH-PL180417045', 'TSH-PL180417077', 'TSH-PL180417104', 'TSH-PL180417111',
'TSH-PL180417124', 'TSH-PL180417162', 'TSH-PL180417169', 'TSH-PL180417199', 'TSH-PL180424042',
'TSH-PL180424088', 'TSH-PL180424138', 'TSH-PL180703012', 'TSH-PL180703127', 'TSH-PL180703134',
'TSH-PL180703141', 'TSH-PL180703168', 'TSH-PL180706117', 'TSH-PL181128004', 'TSH-PL181128006',
'TSH-PL181128013', 'TSH-PL181128018', 'TSH-PL181128020', 'TSH-PL181128031', 'TSH-PL181128037',
'TSH-PL181128067', 'TSH-PL181128071', 'TSH-PL181128078', 'TSH-PL181128084', 'TSH-PL181128095',
'TSH-PL181128097', 'TSH-PL181128098', 'TSH-PL181128105', 'TSH-PL181128106', 'TSH-PL181128108',
'TSH-PL181128113', 'TSH-PL181128116', 'TSH-PL181205001', 'TSH-PL181205004', 'TSH-PL181205007',
'TSH-PL181205010', 'TSH-PL181205011', 'TSH-PL181205012', 'TSH-PL181205015', 'TSH-PL181205018',
'TSH-PL181205019', 'TSH-PL181205023', 'TSH-PL181205031', 'TSH-PL181205032', 'TSH-PL181205036',
'TSH-PL181205037', 'TSH-PL181205052', 'TSH-PL181205056', 'TSH-PL181205067', 'TSH-PL181205070',
'TSH-PL181205071', 'TSH-PL181205073', 'TSH-PL181205080', 'TSH-PL181205084', 'TSH-PL181205089',
'TSH-PL181205092', 'TSH-PL181205094', 'TSH-PL181205100', 'TSH-PL181205101', 'TSH-PL181205106',
'TSH-PL181205112', 'TSH-PL181205118', 'TSH-PL181205119', 'TSH-PL181205120', 'TSH-PL181205123',
'TSH-PL181205124', 'TSH-PL181205153', 'TSH-PL181205154', 'TSH-PL181205159', 'TSH-PL181205163']
```

In [6]:
```
healthy_samples = ['TSH-PL180309087', 'TSH-PL180316003', 'TSH-PL180316005', 'TSH-PL180316016',
'TSH-PL180316017', 'TSH-PL180316018', 'TSH-PL180316022', 'TSH-PL180316024', 'TSH-PL180316027',
'TSH-PL180316033', 'TSH-PL180316037', 'TSH-PL180316038', 'TSH-PL180316039', 'TSH-PL180316049',
'TSH-PL180316061', 'TSH-PL180316065', 'TSH-PL180316079', 'TSH-PL180316092', 'TSH-PL180316095',
'TSH-PL180316099', 'TSH-PL180316101', 'TSH-PL180316106', 'TSH-PL180316115', 'TSH-PL180316119',
'TSH-PL180316126', 'TSH-PL180316129', 'TSH-PL180316157', 'TSH-PL180316161', 'TSH-PL180316163',
'TSH-PL180316164', 'TSH-PL180316173', 'TSH-PL180316174', 'TSH-PL180316181', 'TSH-PL180316183',
'TSH-PL180316187', 'TSH-PL180316190', 'TSH-PL180316195', 'TSH-PL180316196', 'TSH-PL180316198',
'TSH-PL180323034', 'TSH-PL180323038', 'TSH-PL180323051', 'TSH-PL180323052', 'TSH-PL180323054',
'TSH-PL180323059', 'TSH-PL180323065', 'TSH-PL180323067', 'TSH-PL180323072', 'TSH-PL180323073',
'TSH-PL180323091', 'TSH-PL180323106', 'TSH-PL180323107', 'TSH-PL180323109', 'TSH-PL180323110',
'TSH-PL180323111', 'TSH-PL180323119', 'TSH-PL180323121', 'TSH-PL180323124', 'TSH-PL180323139',
'TSH-PL180323141', 'TSH-PL180323142', 'TSH-PL180323151', 'TSH-PL180323155', 'TSH-PL180323166',
'TSH-PL180410003', 'TSH-PL180410023', 'TSH-PL180410043', 'TSH-PL180410049', 'TSH-PL180410059',
'TSH-PL180410063', 'TSH-PL180410076', 'TSH-PL180410087', 'TSH-PL180410115', 'TSH-PL180410116',
'TSH-PL180410133', 'TSH-PL180410140', 'TSH-PL180410144', 'TSH-PL180410160', 'TSH-PL180410162',
'TSH-PL180410198', 'TSH-PL180417003', 'TSH-PL180417007', 'TSH-PL180417035', 'TSH-PL180417060',
'TSH-PL180417074', 'TSH-PL180417087', 'TSH-PL180417095', 'TSH-PL180417101', 'TSH-PL180417109',
'TSH-PL180417117', 'TSH-PL180417135', 'TSH-PL180417138', 'TSH-PL180417143', 'TSH-PL180417152',
'TSH-PL180417156', 'TSH-PL180417165', 'TSH-PL180417170', 'TSH-PL180417179', 'TSH-PL180417180',
'TSH-PL180417181', 'TSH-PL180417200', 'TSH-PL180424003', 'TSH-PL180424030', 'TSH-PL180424036',
'TSH-PL180424058', 'TSH-PL180424090', 'TSH-PL180424097', 'TSH-PL180424104', 'TSH-PL180424115',
'TSH-PL180424146', 'TSH-PL180424149', 'TSH-PL180424155', 'TSH-PL180424162', 'TSH-PL180424166',
'TSH-PL180424171', 'TSH-PL180703037', 'TSH-PL180703136', 'TSH-PL180703153', 'TSH-PL180703157',
'TSH-PL180703185', 'TSH-PL180706034', 'TSH-PL180706037', 'TSH-PL180706095', 'TSH-PL180706096',
'TSH-PL180706104', 'TSH-PL180706113', 'TSH-PL180706118', 'TSH-PL180706145', 'TSH-PL180706147',
'TSH-PL180706150', 'TSH-PL180706153', 'TSH-PL180706156', 'TSH-PL181128001', 'TSH-PL181128003',
'TSH-PL181128005', 'TSH-PL181128007', 'TSH-PL181128008', 'TSH-PL181128009', 'TSH-PL181128011',
'TSH-PL181128012', 'TSH-PL181128014', 'TSH-PL181128015', 'TSH-PL181128019', 'TSH-PL181128021',
'TSH-PL181128022', 'TSH-PL181128023', 'TSH-PL181128025', 'TSH-PL181128027', 'TSH-PL181128029',
'TSH-PL181128030', 'TSH-PL181128033', 'TSH-PL181128035', 'TSH-PL181128036', 'TSH-PL181128038',
'TSH-PL181128039', 'TSH-PL181128040', 'TSH-PL181128041', 'TSH-PL181128042', 'TSH-PL181128043',
'TSH-PL181128045', 'TSH-PL181128047', 'TSH-PL181128048', 'TSH-PL181128049', 'TSH-PL181128050',
'TSH-PL181128051', 'TSH-PL181128054', 'TSH-PL181128055', 'TSH-PL181128056', 'TSH-PL181128057',
'TSH-PL181128058', 'TSH-PL181128061', 'TSH-PL181128062', 'TSH-PL181128064', 'TSH-PL181128065',
'TSH-PL181128066', 'TSH-PL181128070', 'TSH-PL181128073', 'TSH-PL181128077', 'TSH-PL181128080',
'TSH-PL181128082', 'TSH-PL181128083', 'TSH-PL181128085', 'TSH-PL181128086', 'TSH-PL181128093',
'TSH-PL181128096', 'TSH-PL181128099', 'TSH-PL181128101', 'TSH-PL181128102', 'TSH-PL181128103',
'TSH-PL181128104', 'TSH-PL181128107', 'TSH-PL181128109', 'TSH-PL181128110', 'TSH-PL181128111',
'TSH-PL181128112', 'TSH-PL181128114', 'TSH-PL181128115', 'TSH-PL181128117', 'TSH-PL181128118',
'TSH-PL181128120', 'TSH-PL181128122', 'TSH-PL181128123', 'TSH-PL181128124', 'TSH-PL181128125',
'TSH-PL181128126', 'TSH-PL181128127', 'TSH-PL181205002', 'TSH-PL181205003', 'TSH-PL181205005',
'TSH-PL181205006', 'TSH-PL181205008', 'TSH-PL181205009', 'TSH-PL181205013', 'TSH-PL181205014',
'TSH-PL181205016', 'TSH-PL181205020', 'TSH-PL181205022', 'TSH-PL181205024', 'TSH-PL181205026',
'TSH-PL181205027', 'TSH-PL181205028', 'TSH-PL181205030', 'TSH-PL181205033', 'TSH-PL181205035',
'TSH-PL181205040', 'TSH-PL181205041', 'TSH-PL181205042', 'TSH-PL181205043', 'TSH-PL181205044',
'TSH-PL181205045', 'TSH-PL181205046', 'TSH-PL181205047', 'TSH-PL181205048', 'TSH-PL181205049',
'TSH-PL181205051', 'TSH-PL181205053', 'TSH-PL181205054', 'TSH-PL181205055', 'TSH-PL181205057',
'TSH-PL181205058', 'TSH-PL181205059', 'TSH-PL181205060', 'TSH-PL181205061', 'TSH-PL181205062',
'TSH-PL181205063', 'TSH-PL181205064', 'TSH-PL181205065', 'TSH-PL181205066', 'TSH-PL181205068',
'TSH-PL181205069', 'TSH-PL181205072', 'TSH-PL181205074', 'TSH-PL181205075', 'TSH-PL181205076',
'TSH-PL181205077', 'TSH-PL181205078', 'TSH-PL181205079', 'TSH-PL181205081', 'TSH-PL181205082',
'TSH-PL181205083', 'TSH-PL181205085', 'TSH-PL181205086', 'TSH-PL181205087', 'TSH-PL181205088',
'TSH-PL181205090', 'TSH-PL181205091', 'TSH-PL181205093', 'TSH-PL181205095', 'TSH-PL181205096',
'TSH-PL181205097', 'TSH-PL181205098', 'TSH-PL181205099', 'TSH-PL181205102', 'TSH-PL181205103',
'TSH-PL181205104', 'TSH-PL181205107', 'TSH-PL181205108', 'TSH-PL181205110', 'TSH-PL181205113',
'TSH-PL181205115', 'TSH-PL181205116', 'TSH-PL181205117', 'TSH-PL181205121', 'TSH-PL181205122',
'TSH-PL181205125', 'TSH-PL181205128', 'TSH-PL181205129', 'TSH-PL181205140', 'TSH-PL181205141',
'TSH-PL181205142', 'TSH-PL181205143', 'TSH-PL181205144', 'TSH-PL181205145', 'TSH-PL181205146',
'TSH-PL181205147', 'TSH-PL181205148', 'TSH-PL181205149', 'TSH-PL181205150', 'TSH-PL181205151',
'TSH-PL181205152', 'TSH-PL181205155', 'TSH-PL181205156', 'TSH-PL181205157', 'TSH-PL181205158',
'TSH-PL181205160', 'TSH-PL181205161', 'TSH-PL181205162', 'TSH-PL181205164', 'TSH-PL181205165',
'TSH-PL181205166', 'TSH-PL180309082', 'TSH-PL180316013', 'TSH-PL180316029', 'TSH-PL180316075',
'TSH-PL180316102', 'TSH-PL180316112', 'TSH-PL180316134', 'TSH-PL180316138', 'TSH-PL180316175',
'TSH-PL180316178', 'TSH-PL180316200', 'TSH-PL180323029', 'TSH-PL180323032', 'TSH-PL180323053',
'TSH-PL180323068', 'TSH-PL180323074', 'TSH-PL180323088', 'TSH-PL180323097', 'TSH-PL180323103',
'TSH-PL180323115', 'TSH-PL180323120', 'TSH-PL180323160', 'TSH-PL180323175', 'TSH-PL180410058',
'TSH-PL180410070', 'TSH-PL180410081', 'TSH-PL180410148', 'TSH-PL180410161', 'TSH-PL180417017',
'TSH-PL180417023', 'TSH-PL180417045', 'TSH-PL180417077', 'TSH-PL180417104', 'TSH-PL180417111',
'TSH-PL180417124', 'TSH-PL180417162', 'TSH-PL180417169', 'TSH-PL180417199', 'TSH-PL180424042',
'TSH-PL180424088', 'TSH-PL180424138', 'TSH-PL180703012', 'TSH-PL180703127', 'TSH-PL180703134',
'TSH-PL180703141', 'TSH-PL180703168', 'TSH-PL180706117', 'TSH-PL181128004', 'TSH-PL181128006',
'TSH-PL181128013', 'TSH-PL181128018', 'TSH-PL181128020', 'TSH-PL181128031', 'TSH-PL181128037',
'TSH-PL181128067', 'TSH-PL181128071', 'TSH-PL181128078', 'TSH-PL181128084', 'TSH-PL181128095',
'TSH-PL181128097', 'TSH-PL181128098', 'TSH-PL181128105', 'TSH-PL181128106', 'TSH-PL181128108',
'TSH-PL181128113', 'TSH-PL181128116', 'TSH-PL181205001', 'TSH-PL181205004', 'TSH-PL181205007',
'TSH-PL181205010', 'TSH-PL181205011', 'TSH-PL181205012', 'TSH-PL181205015', 'TSH-PL181205018',
'TSH-PL181205019', 'TSH-PL181205023', 'TSH-PL181205031', 'TSH-PL181205032', 'TSH-PL181205036',
'TSH-PL181205037', 'TSH-PL181205052', 'TSH-PL181205056', 'TSH-PL181205067', 'TSH-PL181205070',
'TSH-PL181205071', 'TSH-PL181205073', 'TSH-PL181205080', 'TSH-PL181205084', 'TSH-PL181205089',
'TSH-PL181205092', 'TSH-PL181205094', 'TSH-PL181205100', 'TSH-PL181205101', 'TSH-PL181205106',
'TSH-PL181205112', 'TSH-PL181205118', 'TSH-PL181205119', 'TSH-PL181205120', 'TSH-PL181205123',
'TSH-PL181205124', 'TSH-PL181205153', 'TSH-PL181205154', 'TSH-PL181205159', 'TSH-PL181205163']

post_diagnosis_samples_colon = ['TSH-PL180510139', 'TSH-PL180510140', 'TSH-PL180510146', 'TSH-PL180510147', 'TSH-PL180510162', 'TSH-PL181205175', 'TSH-PL180510143']
post_diagnosis_samples_liver = ['TSH-PL180510085', 'TSH-PL180510089', 'TSH-PL180510092', 'TSH-PL180510095', 'TSH-PL180510101', 'TSH-PL180510104', 'TSH-PL180510105', 'TSH-PL180510107', 'TSH-PL180510112', 'TSH-PL180510121', 'TSH-PL180510122', 'TSH-PL180510125', 'TSH-PL181205173', 'TSH-PL180510088', 'TSH-PL180510090', 'TSH-PL180510098', 'TSH-PL180510100', 'TSH-PL180510103', 'TSH-PL180510108', 'TSH-PL180510109', 'TSH-PL180510113', 'TSH-PL180510117', 'TSH-PL180510124']
post_diagnosis_samples_esophagus = ['TSH-PL171221101', 'TSH-PL171221112', 'TSH-PL171221149', 'TSH-PL171221150', 'TSH-PL171221160', 'TSH-PL171221172', 'TSH-PL171221179', 'TSH-PL171221180',
'TSH-PL180508001', 'TSH-PL180508004', 'TSH-PL180508005', 'TSH-PL180508006', 'TSH-PL180508008',
'TSH-PL180508009', 'TSH-PL180508011', 'TSH-PL180508012', 'TSH-PL180508013', 'TSH-PL180508014',
'TSH-PL180508015', 'TSH-PL180508019', 'TSH-PL180508020', 'TSH-PL180508021', 'TSH-PL180508022',
'TSH-PL180508025', 'TSH-PL180508030', 'TSH-PL180508031', 'TSH-PL180508033', 'TSH-PL180508034',
'TSH-PL180508036', 'TSH-PL180508037', 'TSH-PL180508038', 'TSH-PL180508039', 'TSH-PL180508040',
'TSH-PL180508041', 'TSH-PL180508042', 'TSH-PL180508043', 'TSH-PL180508049', 'TSH-PL180508050',
'TSH-PL180508060', 'TSH-PL180508061', 'TSH-PL180508064', 'TSH-PL180508068', 'TSH-PL180508076',
'TSH-PL180508078', 'TSH-PL180508083', 'TSH-PL180508086', 'TSH-PL180508089', 'TSH-PL180510044',
'TSH-PL180510061', 'TSH-PL181205168', 'TSH-PL181205170', 'TSH-PL171221151', 'TSH-PL180508003',
'TSH-PL180508007', 'TSH-PL180508023', 'TSH-PL180508026', 'TSH-PL180508028', 'TSH-PL180508029',
'TSH-PL180508032', 'TSH-PL180508044', 'TSH-PL180508052', 'TSH-PL180508065', 'TSH-PL180508067',
'TSH-PL180508088', 'TSH-PL181205172', 'TSH-PL181205174', 'TSH-PL171221153', 'TSH-PL171221113']
post_diagnosis_samples_lung = ['TSH-PL180510005', 'TSH-PL180510006', 'TSH-PL180510007', 'TSH-PL180510008', 'TSH-PL180510010', 'TSH-PL180510011', 'TSH-PL180510012', 'TSH-PL180510014', 'TSH-PL180510015', 'TSH-PL180510016', 'TSH-PL180510017', 'TSH-PL180510018', 'TSH-PL180510019', 'TSH-PL180510021', 'TSH-PL180510023', 'TSH-PL180510024', 'TSH-PL180510025', 'TSH-PL180510026', 'TSH-PL180510030', 'TSH-PL180510031', 'TSH-PL180510033', 'TSH-PL180510038', 'TSH-PL180510040', 'TSH-PL180510047', 'TSH-PL180510050', 'TSH-PL180510052', 'TSH-PL180510053', 'TSH-PL180510054', 'TSH-PL180510055', 'TSH-PL180510057', 'TSH-PL180510063', 'TSH-PL180510066', 'TSH-PL180510071',
'TSH-PL180510072', 'TSH-PL180510073', 'TSH-PL180510074', 'TSH-PL180510075', 'TSH-PL180510076',
'TSH-PL180510077', 'TSH-PL180510078', 'TSH-PL180510079', 'TSH-PL180510080', 'TSH-PL181205182',
'TSH-PL180510002', 'TSH-PL180510004', 'TSH-PL180510013', 'TSH-PL180510020', 'TSH-PL180510022',
'TSH-PL180510032', 'TSH-PL180510034', 'TSH-PL180510035', 'TSH-PL180510043', 'TSH-PL180510048',
'TSH-PL180510068', 'TSH-PL181205177', 'TSH-PL181205179']
post_diagnosis_samples_stomach = ['TSH-PL180504001', 'TSH-PL180504002', 'TSH-PL180504003', 'TSH-PL180504004', 'TSH-PL180504005', 'TSH-PL180504009', 'TSH-PL180504010', 'TSH-PL180504011', 'TSH-PL180504013', 'TSH-PL180504014', 'TSH-PL180504015', 'TSH-PL180504016', 'TSH-PL180504017', 'TSH-PL180504018', 'TSH-PL180504019', 'TSH-PL180504021', 'TSH-PL180504022', 'TSH-PL180504027',
'TSH-PL180504028', 'TSH-PL180504030', 'TSH-PL180504033', 'TSH-PL180504034', 'TSH-PL180504041',
'TSH-PL180504042', 'TSH-PL180504044', 'TSH-PL180504046', 'TSH-PL180504047', 'TSH-PL180504049',
'TSH-PL180504050', 'TSH-PL180504053', 'TSH-PL180504054', 'TSH-PL180504058', 'TSH-PL180504061',
'TSH-PL180504062', 'TSH-PL180504063', 'TSH-PL180504065', 'TSH-PL180504068', 'TSH-PL180504070',
'TSH-PL180504074', 'TSH-PL180504081', 'TSH-PL180504083', 'TSH-PL180504087', 'TSH-PL180504088',
'TSH-PL180504089', 'TSH-PL180504091', 'TSH-PL180504094', 'TSH-PL180504097', 'TSH-PL180504101',
'TSH-PL180504104', 'TSH-PL180504108', 'TSH-PL180504111', 'TSH-PL180504118', 'TSH-PL180504006',
'TSH-PL180504012', 'TSH-PL180504024', 'TSH-PL180504026', 'TSH-PL180504029', 'TSH-PL180504037',
'TSH-PL180504043', 'TSH-PL180504045', 'TSH-PL180504051', 'TSH-PL180504052', 'TSH-PL180504055',
'TSH-PL180504057', 'TSH-PL180504064', 'TSH-PL180504076', 'TSH-PL180504084', 'TSH-PL180504110',
'TSH-PL181205169']

pre_diagnosis_samples_colon = ['TSH-PL180123001', 'TSH-PL180123010', 'TSH-PL180513013', 'TSH-PL180513014', 'TSH-PL180513020', 'TSH-PL180513027', 'TSH-PL180513029', 'TSH-PL180513030', 'TSH-PL180513034', 'TSH-PL180513037', 'TSH-PL180513049', 'TSH-PL180513062', 'TSH-PL180513077', 'TSH-PL180513078', 'TSH-PL180513085', 'TSH-PL180513114', 'TSH-PL180516003', 'TSH-PL180516011', 'TSH-PL180516012', 'TSH-PL180516014', 'TSH-PL180516018', 'TSH-PL180516021', 'TSH-PL180516028', 'TSH-PL180516039', 'TSH-PL180516044', 'TSH-PL180516053', 'TSH-PL180516057', 'TSH-PL180513001', 'TSH-PL180513002', 'TSH-PL180513064', 'TSH-PL180513116', 'TSH-PL180516015', 'TSH-PL180516017', 'TSH-PL180516026', 'TSH-PL180516033']
pre_diagnosis_samples_liver = ['TSH-PL180123014', 'TSH-PL180123031', 'TSH-PL180513006', 'TSH-PL180513011', 'TSH-PL180513017', 'TSH-PL180513021', 'TSH-PL180513026', 'TSH-PL180513041', 'TSH-PL180513043', 'TSH-PL180513071', 'TSH-PL180513088', 'TSH-PL180513095', 'TSH-PL180513098', 'TSH-PL180513108', 'TSH-PL180513109', 'TSH-PL180513111', 'TSH-PL180513117', 'TSH-PL180516006', 'TSH-PL180516038', 'TSH-PL180516040', 'TSH-PL180516051', 'TSH-PL180516054', 'TSH-PL180516063', 'TSH-PL180123004', 'TSH-PL180123005', 'TSH-PL180513009', 'TSH-PL180513033', 'TSH-PL180513090', 'TSH-PL180516025']
pre_diagnosis_samples_esophagus = ['TSH-PL180123006', 'TSH-PL180123007', 'TSH-PL180123008', 'TSH-PL180123016', 'TSH-PL180123025', 'TSH-PL180513004', 'TSH-PL180513005', 'TSH-PL180513016', 'TSH-PL180513051', 'TSH-PL180513068', 'TSH-PL180513072', 'TSH-PL180513075', 'TSH-PL180513080',
'TSH-PL180513081', 'TSH-PL180513091', 'TSH-PL180513093', 'TSH-PL180513097', 'TSH-PL180513100',
'TSH-PL180513101', 'TSH-PL180513110', 'TSH-PL180513115', 'TSH-PL180513118', 'TSH-PL180513121',
'TSH-PL180513122', 'TSH-PL180513124', 'TSH-PL180513128', 'TSH-PL180513129', 'TSH-PL180516013',
'TSH-PL180516020', 'TSH-PL180516030', 'TSH-PL180516031', 'TSH-PL180516045', 'TSH-PL180516049',
'TSH-PL180516059', 'TSH-PL180123024', 'TSH-PL180123032', 'TSH-PL180513028', 'TSH-PL180513032',
'TSH-PL180513058', 'TSH-PL180513083', 'TSH-PL180513096', 'TSH-PL180516005', 'TSH-PL180516047',
'TSH-PL180516050', 'TSH-PL180516061']
pre_diagnosis_samples_lung = ['TSH-PL180123013', 'TSH-PL180123021', 'TSH-PL180123022', 'TSH-PL180123029', 'TSH-PL180123030', 'TSH-PL180513007', 'TSH-PL180513012', 'TSH-PL180513023', 'TSH-PL180513024', 'TSH-PL180513036', 'TSH-PL180513040', 'TSH-PL180513045', 'TSH-PL180513055', 'TSH-PL180513056', 'TSH-PL180513057', 'TSH-PL180513067', 'TSH-PL180513069', 'TSH-PL180513079', 'TSH-PL180513084', 'TSH-PL180513087', 'TSH-PL180513092', 'TSH-PL180513106', 'TSH-PL180516001', 'TSH-PL180516002', 'TSH-PL180516004', 'TSH-PL180516016', 'TSH-PL180516023', 'TSH-PL180516032', 'TSH-PL180516055', 'TSH-PL180516056', 'TSH-PL180516062', 'TSH-PL180123002', 'TSH-PL180123012', 'TSH-PL180513010', 'TSH-PL180513019', 'TSH-PL180513022', 'TSH-PL180513031', 'TSH-PL180513035',
'TSH-PL180513044', 'TSH-PL180513086', 'TSH-PL180513112', 'TSH-PL180516029', 'TSH-PL180516034',
'TSH-PL180516046', 'TSH-PL180516048', 'TSH-PL180516058', 'TSH-PL180516060']
pre_diagnosis_samples_stomach = ['TSH-PL180123017', 'TSH-PL180123018', 'TSH-PL180123027', 'TSH-PL180123033', 'TSH-PL180513025', 'TSH-PL180513038', 'TSH-PL180513039', 'TSH-PL180513050', 'TSH-PL180513060', 'TSH-PL180513066', 'TSH-PL180513070', 'TSH-PL180513073', 'TSH-PL180513076', 'TSH-PL180513089', 'TSH-PL180513099', 'TSH-PL180513102', 'TSH-PL180513104', 'TSH-PL180513119', 'TSH-PL180513123', 'TSH-PL180513127', 'TSH-PL180516007', 'TSH-PL180516008', 'TSH-PL180516009',
'TSH-PL180516019', 'TSH-PL180516027', 'TSH-PL180516036', 'TSH-PL180516042', 'TSH-PL180516043',
'TSH-PL180513003', 'TSH-PL180513018', 'TSH-PL180513103', 'TSH-PL180513105', 'TSH-PL180513125',
'TSH-PL180516010', 'TSH-PL180516052']
```

BioChain Tissue Samples

In [7]:
```
healthy_lung_tissue = ['BC_Lung_ND1', 'BC_Lung_ND2', 'BC_Lung_ND3', 'BC_Lung_ND4', 'BC_Lung_ND5', 'BC_Lung_ND6', 'BC_Lung_ND7', 'BC_Lung_ND8']
healthy_colon_tissue = ['BC_CRC_ND1', 'BC_CRC_ND2', 'BC_CRC_ND3', 'BC_CRC_ND4', 'BC_CRC_ND5', 'BC_CRC_ND6', 'BC_CRC_ND7', 'BC_CRC_ND8']
healthy_stomach_tissue = ['BC_Stomach_ND1', 'BC_Stomach_ND2', 'BC_Stomach_ND3', 'BC_Stomach_ND4', 'BC_Stomach_ND5', 'BC_Stomach_ND6', 'BC_Stomach_ND7', 'BC_Stomach_ND8']
healthy_breast_tissue = ['BC_Breast_ND1', 'BC_Breast_ND2', 'BC_Breast_ND3', 'BC_Breast_ND4', 'BC_Breast_ND5', 'BC_Breast_ND6', 'BC_Breast_ND7', 'BC_Breast_ND8']

cancer_lung_tissue = ['BC_Lung_TD1', 'BC_Lung_TD10', 'BC_Lung_TD11', 'BC_Lung_TD12', 'BC_Lung_TD13', 'BC_Lung_TD14', 'BC_Lung_TD15', 'BC_Lung_TD16', 'BC_Lung_TD17', 'BC_Lung_TD18', 'BC_Lung_TD19', 'BC_Lung_TD2', 'BC_Lung_TD20', 'BC_Lung_TD21', 'BC_Lung_TD22', 'BC_Lung_TD23', 'BC_Lung_TD24', 'BC_Lung_TD25', 'BC_Lung_TD26', 'BC_Lung_TD27', 'BC_Lung_TD28', 'BC_Lung_TD29', 'BC_Lung_TD3', 'BC_Lung_TD30', 'BC_Lung_TD31', 'BC_Lung_TD32', 'BC_Lung_TD33', 'BC_Lung_TD34', 'BC_Lung_TD35', 'BC_Lung_TD36', 'BC_Lung_TD37', 'BC_Lung_TD38', 'BC_Lung_TD39', 'BC_Lung_TD4', 'BC_Lung_TD40', 'BC_Lung_TD5', 'BC_Lung_TD6', 'BC_Lung_TD7', 'BC_Lung_TD8', 'BC_Lung_TD9']
cancer_colon_tissue = ['BC_CRC_TD1', 'BC_CRC_TD10', 'BC_CRC_TD11', 'BC_CRC_TD12', 'BC_CRC_TD13', 'BC_CRC_TD14', 'BC_CRC_TD15', 'BC_CRC_TD16', 'BC_CRC_TD17', 'BC_CRC_TD18', 'BC_CRC_TD19', 'BC_CRC_TD2', 'BC_CRC_TD20', 'BC_CRC_TD21', 'BC_CRC_TD22', 'BC_CRC_TD23', 'BC_CRC_TD24', 'BC_CRC_TD25', 'BC_CRC_TD26', 'BC_CRC_TD27', 'BC_CRC_TD28', 'BC_CRC_TD29', 'BC_CRC_TD3', 'BC_CRC_TD30', 'BC_CRC_TD31', 'BC_CRC_TD32', 'BC_CRC_TD33', 'BC_CRC_TD34', 'BC_CRC_TD35', 'BC_CRC_TD36', 'BC_CRC_TD37', 'BC_CRC_TD38', 'BC_CRC_TD39', 'BC_CRC_TD4', 'BC_CRC_TD40', 'BC_CRC_TD5', 'BC_CRC_TD6', 'BC_CRC_TD7', 'BC_CRC_TD8', 'BC_CRC_TD9']
cancer_stomach_tissue = ['BC_Stomach_TD1', 'BC_Stomach_TD10', 'BC_Stomach_TD11', 'BC_Stomach_TD12', 'BC_Stomach_TD13', 'BC_Stomach_TD14', 'BC_Stomach_TD15', 'BC_Stomach_TD16', 'BC_Stomach_TD17', 'BC_Stomach_TD18', 'BC_Stomach_TD19', 'BC_Stomach_TD2', 'BC_Stomach_TD20', 'BC_Stomach_TD21', 'BC_Stomach_TD22', 'BC_Stomach_TD23', 'BC_Stomach_TD24', 'BC_Stomach_TD25', 'BC_Stomach_TD26', 'BC_Stomach_TD27', 'BC_Stomach_TD28', 'BC_Stomach_TD29', 'BC_Stomach_TD3', 'BC_Stomach_TD30', 'BC_Stomach_TD31', 'BC_Stomach_TD32', 'BC_Stomach_TD33', 'BC_Stomach_TD34', 'BC_Stomach_TD35', 'BC_Stomach_TD36', 'BC_Stomach_TD37', 'BC_Stomach_TD38', 'BC_Stomach_TD39', 'BC_Stomach_TD4', 'BC_Stomach_TD40', 'BC_Stomach_TD5', 'BC_Stomach_TD6', 'BC_Stomach_TD7', 'BC_Stomach_TD8', 'BC_Stomach_TD9']
cancer_breast_tissue = ['BC_Breast_TD1', 'BC_Breast_TD10', 'BC_Breast_TD11', 'BC_Breast_TD12', 'BC_Breast_TD13', 'BC_Breast_TD14', 'BC_Breast_TD15', 'BC_Breast_TD16', 'BC_Breast_TD17', 'BC_Breast_TD18', 'BC_Breast_TD19', 'BC_Breast_TD2', 'BC_Breast_TD20', 'BC_Breast_TD21', 'BC_Breast_TD22', 'BC_Breast_TD23', 'BC_Breast_TD24', 'BC_Breast_TD25', 'BC_Breast_TD26', 'BC_Breast_TD27', 'BC_Breast_TD28', 'BC_Breast_TD29', 'BC_Breast_TD3', 'BC_Breast_TD30', 'BC_Breast_TD31', 'BC_Breast_TD32', 'BC_Breast_TD33', 'BC_Breast_TD34', 'BC_Breast_TD35', 'BC_Breast_TD36', 'BC_Breast_TD37', 'BC_Breast_TD38', 'BC_Breast_TD39', 'BC_Breast_TD4', 'BC_Breast_TD40', 'BC_Breast_TD5', 'BC_Breast_TD6', 'BC_Breast_TD7', 'BC_Breast_TD8', 'BC_Breast_TD9']

all_tissue_samples = healthy_lung_tissue+healthy_colon_tissue+healthy_stomach_tissue+healthy_breast_tissue+cancer_lung_tissue+cancer_colon_tissue+cancer_stomach_tissue+cancer_breast_tissue
```

Load Data

Let's load a data matrix listing the average methylation across each target region for each sample.

In [8]:
```
amf_df_orig = pd.read_csv('SupplementaryData5.tsv', sep="\t", header=0, index_col=0)
```

Marker Selection

Let's first remove markers that are not covered in all plasma samples, so we don't have to deal with any missing values.

In [9]:
```
amf_df = amf_df_orig.copy()
amf_df = amf_df.loc[amf_df[healthy_samples+post_diagnosis_samples+pre_diagnosis_samples].dropna(how='any').index]
```

Next, let's choose markers that separate healthy tissue from cancer tissue.

For each tissue type we purchased from BioChain, we will use a T-test to identify target regions that separate normal tissue from cancer tissue (applying a Benjamini-Hochberg multiple testing correction).

In [10]:
```
tissue_t_tests = pd.concat(
    [
        pd.Series(amf_df.index),
        pd.Series(scipy.stats.ttest_ind(amf_df.loc[:, healthy_colon_tissue].T,
                                        amf_df.loc[:, cancer_colon_tissue].T,
                                        axis=0, equal_var=True, nan_policy='omit').pvalue),
        pd.Series(scipy.stats.ttest_ind(amf_df.loc[:, healthy_lung_tissue].T,
                                        amf_df.loc[:, cancer_lung_tissue].T,
                                        axis=0, equal_var=True, nan_policy='omit').pvalue),
        pd.Series(scipy.stats.ttest_ind(amf_df.loc[:, healthy_stomach_tissue].T,
                                        amf_df.loc[:, cancer_stomach_tissue].T,
                                        axis=0, equal_var=True, nan_policy='omit').pvalue),
        pd.Series(scipy.stats.ttest_ind(amf_df.loc[:, healthy_breast_tissue].T,
                                        amf_df.loc[:, cancer_breast_tissue].T,
                                        axis=0, equal_var=True, nan_policy='omit').pvalue),
    ], axis=1
)

tissue_t_tests.columns = ['Locus',
                          'ColonM', 'LungM', 'StomachM', 'BreastM',
                         ]
tissue_t_tests = tissue_t_tests.set_index('Locus')

# Benjamini-Hochberg Correction
from statsmodels.stats.multitest import multipletests
_, pvals_corrected, _, _ = multipletests(tissue_t_tests.min(axis=1).fillna(1).values, alpha=0.05, method='fdr_bh')

tissue_t_tests_min_by_marker = tissue_t_tests.loc[pvals_corrected <= 0.05].iloc[:, :4].min(axis=1).copy()
```

In [11]:
```
print len(tissue_t_tests_min_by_marker)
```

```
477
```

We can then use these target regions in our plasma samples to build a classifier.

Divide Samples Into Leave-In and Leave-Out

Let's first split the Taizhou samples into two sets: leave-in (1/2) and leave-out (1/2). The leave-in set will be used to build a model.

We want to make sure that equal proportions of healthy/cancer samples are included in the leave-in/leave-out set. We also want balance within each type of cancer (so we don't end up with no samples for one cancer type in the leave-out set, for example).

In [12]:
```
random.seed(128)

# Healthy
random.shuffle(healthy_samples)
# Post-Diagnosis
random.shuffle(post_diagnosis_samples_colon)
random.shuffle(post_diagnosis_samples_liver)
random.shuffle(post_diagnosis_samples_esophagus)
random.shuffle(post_diagnosis_samples_lung)
random.shuffle(post_diagnosis_samples_stomach)
# Pre-Diagnosis
random.shuffle(pre_diagnosis_samples_colon)
random.shuffle(pre_diagnosis_samples_liver)
random.shuffle(pre_diagnosis_samples_esophagus)
random.shuffle(pre_diagnosis_samples_lung)
random.shuffle(pre_diagnosis_samples_stomach)

negative_leavein = list(healthy_samples[:int(len(healthy_samples)/2)])
negative_leaveout = list(healthy_samples[int(len(healthy_samples)/2):])

positive_post_leavein = post_diagnosis_samples_colon[:int(len(post_diagnosis_samples_colon)/2)] + post_diagnosis_samples_liver[:int(len(post_diagnosis_samples_liver)/2)] + post_diagnosis_samples_esophagus[:int(len(post_diagnosis_samples_esophagus)/2)] + post_diagnosis_samples_lung[:int(len(post_diagnosis_samples_lung)/2)] + post_diagnosis_samples_stomach[:int(len(post_diagnosis_samples_stomach)/2)]
positive_post_leaveout = post_diagnosis_samples_colon[int(len(post_diagnosis_samples_colon)/2):] + post_diagnosis_samples_liver[int(len(post_diagnosis_samples_liver)/2):] + post_diagnosis_samples_esophagus[int(len(post_diagnosis_samples_esophagus)/2):] + post_diagnosis_samples_lung[int(len(post_diagnosis_samples_lung)/2):] + post_diagnosis_samples_stomach[int(len(post_diagnosis_samples_stomach)/2):]

positive_pre_leavein = pre_diagnosis_samples_colon[:int(len(pre_diagnosis_samples_colon)/2)] + pre_diagnosis_samples_liver[:int(len(pre_diagnosis_samples_liver)/2)] + pre_diagnosis_samples_esophagus[:int(len(pre_diagnosis_samples_esophagus)/2)] + pre_diagnosis_samples_lung[:int(len(pre_diagnosis_samples_lung)/2)] + pre_diagnosis_samples_stomach[:int(len(pre_diagnosis_samples_stomach)/2)]
positive_pre_leaveout = pre_diagnosis_samples_colon[int(len(pre_diagnosis_samples_colon)/2):] + pre_diagnosis_samples_liver[int(len(pre_diagnosis_samples_liver)/2):] + pre_diagnosis_samples_esophagus[int(len(pre_diagnosis_samples_esophagus)/2):] + pre_diagnosis_samples_lung[int(len(pre_diagnosis_samples_lung)/2):] + pre_diagnosis_samples_stomach[int(len(pre_diagnosis_samples_stomach)/2):]
```

Build a Logistic Regression Classifier

Now, let's build an LR classifier from the leave-in set. We will use the method described in Teschendorff et al, Nature Materials (2019). Briefly:

1. We will randomly split the leave-in set into 50% training, 50% test.
2. We will train an LR classifier on the training set, and compute the results on the test set.
3. We will repeat steps 1 and 2 using 1000 different random splits.
4. We will then compute the ensemble performance of these 1000 models across the ten separate test sets, and report this as the "leave-in set" accuracy.
5. We will then apply all 1000 models to the "leave-out set," compute the ensemble performance, and report this as the "leave-out set" accuracy.

Let's build each LR classifier using 1000 different random splits:

In [13]:
```
negative_test_list = []
# positive_post_test_list = []
positive_pre_test_list = []
z_prob_list = []
model_list = []
clf_list = []
intercept_list = []

for random_state in range(1000):
    random.seed(random_state)

    # Set up LR classifier that will automatically fit the best C value using the training set
    clf = LogisticRegressionCV(penalty='l1', solver='liblinear', random_state=random_state,
                               Cs=[1.0, 5.0, 10.0, 50.0, 100.0], cv=3)

    # Split the leave-in set into training and test set
    negative = list(negative_leavein)
    positive_post = list(positive_post_leavein)
    positive_pre = list(positive_pre_leavein)
    random.shuffle(negative)
    random.shuffle(positive_post)
    random.shuffle(positive_pre)
    negative_training, negative_test = negative[:1*int(len(negative)/2)], negative[int(1*len(negative)/2):]
    positive_post_training, positive_post_test = positive_post[:1*int(len(positive_post)/2)], positive_post[int(1*len(positive_post)/2):]
    positive_pre_training, positive_pre_test = positive_pre[:1*int(len(positive_pre)/2)], positive_pre[int(1*len(positive_pre)/2):]
    # positive_training = positive_post_training+positive_pre_training
    positive_training = positive_pre_training
    # positive_test = positive_post_test+positive_pre_test
    positive_test = positive_pre_test

    # Build the model and set parameters using the training set
    x = amf_df.loc[tissue_t_tests_min_by_marker.index, negative_training+positive_training].transpose()
    y = ['Healthy']*len(negative_training) + ['Cancer']*len(positive_training)
    clf.fit(x,y)
    model_list.append(clf)
    clf_list.append(clf.coef_[0])
    intercept_list.append(clf.intercept_[0])

    # Compute the accuracy of this classifier on the leave-in and leave-out sets
    z = pd.Series(clf.predict(amf_df.loc[tissue_t_tests_min_by_marker.index, healthy_samples+pre_diagnosis_samples].transpose()),
                  index=healthy_samples+pre_diagnosis_samples)
    z_prob = pd.Series(clf.predict_proba(amf_df.loc[tissue_t_tests_min_by_marker.index, healthy_samples+pre_diagnosis_samples].transpose())[:, 0],
                       index=healthy_samples+pre_diagnosis_samples)

    # Store the results
    negative_test_list.append(negative_test)
    # positive_post_test_list.append(positive_post_test)
    positive_pre_test_list.append(positive_pre_test)
    z_prob_list.append(z_prob)
```

Next, let's compute the ensemble performance across the leave-in test sets and the leave-out sets.

In [14]:
```
ensemble_score_leavein = pd.concat([z_prob_list[index].loc[negative_test_list[index]+positive_pre_test_list[index]]
                                    for index in range(1000)],
                                   axis=1, sort=True).mean(axis=1)
ensemble_score_leaveout = pd.concat([z_prob_list[index].loc[negative_leaveout+positive_pre_leaveout]
                                     for index in range(1000)],
                                    axis=1, sort=True).mean(axis=1)
```

We can now compute accuracy metrics for the two sets; as per Teschendorff et al 2019, we will choose the model cutoff using the leave-in set.

Leave-In Set Accuracy

Let's choose a cutoff that aims for ~95% specificity in the leave-in set, and compute the associated sensitivity.

In [15]:
```
sns.set_style('whitegrid')

# y_true = ['Healthy']*len(negative_leavein) + ['Cancer']*len(positive_post_leavein)
# y_score = ensemble_score_leavein.loc[negative_leavein+positive_post_leavein]
# cur_fpr, cur_tpr, cur_cutoff = roc_curve(y_true, y_score, pos_label='Cancer')
# cur_auc = auc(cur_fpr, cur_tpr)
# plt.plot(cur_fpr, cur_tpr, label=r'Post-Diagnosis ROC (AUC = %0.2f)' % cur_auc)

y_true = ['Healthy']*len(negative_leavein) + ['Cancer']*len(positive_pre_leavein)
y_score = ensemble_score_leavein.loc[negative_leavein+positive_pre_leavein]
cur_fpr, cur_tpr, cur_cutoff = roc_curve(y_true, y_score, pos_label='Cancer')
cur_auc = auc(cur_fpr, cur_tpr)
plt.plot(cur_fpr, cur_tpr, label=r'Pre-Diagnosis ROC (AUC = %0.2f)' % cur_auc)

plt.xlim([-0.0, 1.0])
plt.ylim([-0.0, 1.0])
plt.xticks([0, 0.05, 0.1, 0.2, 0.3, 0.4, 0.5, 0.6, 0.7, 0.8, 0.9, 1.0])
plt.yticks([0, 0.05, 0.1, 0.2, 0.3, 0.4, 0.5, 0.6, 0.7, 0.8, 0.9, 1.0])
plt.xlabel('False Positive Rate')
plt.ylabel('True Positive Rate')
plt.legend(loc="lower right");

print "Leave-In Results"
print "Specificity: %s" % (sum(ensemble_score_leavein.loc[negative_leavein] < 0.33593) / len(negative_leavein))
# print "Post-Diagnosis Sensitivity: %s" % (sum(ensemble_score_leavein.loc[positive_post_leavein] >= 0.33593) / len(positive_post_leavein))
print "Pre-Diagnosis Sensitivity: %s" % (sum(ensemble_score_leavein.loc[positive_pre_leavein] >= 0.33593) / len(positive_pre_leavein))

plt.figure()
sns.distplot(ensemble_score_leavein.loc[negative_leavein])
# sns.distplot(ensemble_score_leavein.loc[positive_post_leavein])
sns.distplot(ensemble_score_leavein.loc[positive_pre_leavein])
# plt.legend(['Healthy', 'Post-Diagnosis', 'Pre-Diagnosis']);
plt.legend(['Healthy', 'Pre-Diagnosis']);
plt.xlim(0, 1)
```

```
Leave-In Results
Specificity: 0.946859903382
Pre-Diagnosis Sensitivity: 0.967741935484
```

Out[15]: (0, 1)

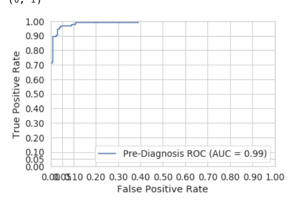

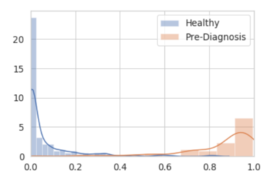

Leave-Out Set Accuracy

Let's compute the leave-out set sensitivity and specificity using the previously chosen cutoff.

In [16]:
```
sns.set_style('whitegrid')

#y_true = ['Healthy']*len(negative_leaveout) + ['Cancer']*len(positive_post_leaveout)
#y_score = ensemble_score_leaveout.loc[negative_leaveout+positive_post_leaveout]
#cur_fpr, cur_tpr, cur_cutoff = roc_curve(y_true, y_score, pos_label='Cancer')
#cur_auc = auc(cur_fpr, cur_tpr)
#plt.plot(cur_fpr, cur_tpr, label=r'Post-Diagnosis ROC (AUC = %0.2f)' % cur_auc)

y_true = ['Healthy']*len(negative_leaveout) + ['Cancer']*len(positive_pre_leaveout)
y_score = ensemble_score_leaveout.loc[negative_leaveout+positive_pre_leaveout]
cur_fpr, cur_tpr, cur_cutoff = roc_curve(y_true, y_score, pos_label='Cancer')
cur_auc = auc(cur_fpr, cur_tpr)
plt.plot(cur_fpr, cur_tpr, label=r'Pre-Diagnosis ROC (AUC = %0.2f)' % cur_auc)

plt.xlim([-0.0, 1.0])
plt.ylim([-0.0, 1.0])
plt.xticks([0, 0.05, 0.1, 0.2, 0.3, 0.4, 0.5, 0.6, 0.7, 0.8, 0.9, 1.0])
plt.yticks([0, 0.05, 0.1, 0.2, 0.3, 0.4, 0.5, 0.6, 0.7, 0.8, 0.9, 1.0])
plt.xlabel('False Positive Rate')
plt.ylabel('True Positive Rate')
plt.legend(loc="lower right");

print "Leave-Out Results"
print "Specificity: %s" % (sum(ensemble_score_leaveout.loc[negative_leaveout] < 0.33593) / len(negative_leaveout))
# print "Post-Diagnosis Sensitivity: %s" % (sum(ensemble_score_leaveout.loc[positive_post_leaveout] >= 0.33593) / len(positive_post_leaveout))
print "Pre-Diagnosis Sensitivity: %s" % (sum(ensemble_score_leaveout.loc[positive_pre_leaveout] >= 0.33593) / len(positive_pre_leaveout))

plt.figure()
sns.distplot(ensemble_score_leaveout.loc[negative_leaveout])
#sns.distplot(ensemble_score_leaveout.loc[positive_post_leaveout])
sns.distplot(ensemble_score_leaveout.loc[positive_pre_leaveout])
plt.legend(['Healthy', 'Post-Diagnosis', 'Pre-Diagnosis']);
plt.legend(['Healthy', 'Pre-Diagnosis']);
plt.xlim(0, 1)
```

```
Leave-Out Results
Specificity: 0.961352657005
Pre-Diagnosis Sensitivity: 0.948979591837
```

Out[16]: (0, 1)

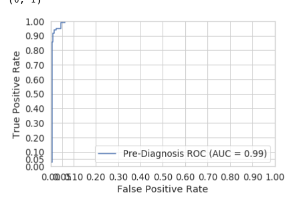

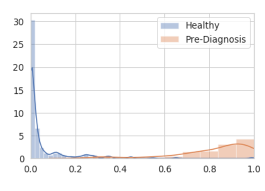

As per Teschendorff et al 2019, the accuracy is similar for the leave-in and leave-out data sets.

Leave-out set accuracy was identical to that of Supplementary Note 1 (a model utilizing both pre-diagnosis and post-diagnosis samples).

Supplementary Note 4.
Code utilized for analytical limit of detection analysis. In spike-in samples, PanSeer assay was able to detect methylation changes associated with cancer down to the 0.1% level.

Supplementary Note 4

In [1]:
```
from __future__ import division
import pandas as pd
import numpy as np
import os
import glob
```

In [2]:
```
%matplotlib inline
import matplotlib.pyplot as plt
import seaborn as sns
```

Sample Info

A set of spike-in samples consisting of HT29 genomic DNA spiked at various percentages into mixed DNA from several healthy plasma samples were processed using the PanSeer assay.

In [3]:
```
HT29_0percent = ['HT29_0percent_1', 'HT29_0percent_2', 'HT29_0percent_3', 'HT29_0percent_4', '
HT29_0percent_5', 'HT29_0percent_6']
HT29_0p1percent = ['HT29_0p1percent_1', 'HT29_0p1percent_2', 'HT29_0p1percent_3', 'HT29_0p1per
cent_4', 'HT29_0p1percent_5', 'HT29_0p1percent_6']
HT29_0p5percent = ['HT29_0p5percent_1', 'HT29_0p5percent_2', 'HT29_0p5percent_3', 'HT29_0p5per
cent_4', 'HT29_0p5percent_5', 'HT29_0p5percent_6']
HT29_1percent = ['HT29_1percent_1', 'HT29_1percent_2', 'HT29_1percent_3', 'HT29_1percent_4', '
HT29_1percent_5', 'HT29_1percent_6']
HT29_5percent = ['HT29_5percent_1', 'HT29_5percent_2', 'HT29_5percent_3', 'HT29_5percent_4', '
HT29_5percent_5', 'HT29_5percent_6']
HT29_10percent = ['HT29_10percent_1', 'HT29_10percent_2', 'HT29_10percent_3', 'HT29_10percent_
4', 'HT29_10percent_5', 'HT29_10percent_6']
HT29_100percent = ['HT29_100percent_1', 'HT29_100percent_2', 'HT29_100percent_3', 'HT29_100per
cent_4', 'HT29_100percent_5']
```

Load Data

Let's load a data matrix listing the methylation level across each CpG site for each sample.

In [6]:
```
amf_df = pd.read_csv('DataS2.tsv', sep="\t", header=0, index_col=0)
```

Cutoff Selection

Let's use three healthy plasma samples to identify the background level of methylation for each CpG site.

In [9]:
```
training_samples = [HT29_0percent[1], HT29_0percent[3], HT29_0percent[5]]
cutoffs = amf_df[training_samples].mean(axis=1) + amf_df[training_samples].std(axis=1)*3
```

Let's also identify CpG sites that have high methylation in the tissue samples.

In [11]:
```
high_in_tissue = amf_df[amf_df[HT29_100percent].mean(axis=1) > 0.6].index
```

CpG Site Counting

Let's count the number of CpG sites that are above the cutoff in each sample.

In [29]:
```
cpg_count_df = {}
for x in [HT29_0percent[0], HT29_0percent[2], HT29_0percent[4]]:
    cpg_count_df[x] = [0, sum(amf_df.loc[high_in_tissue, x] > cutoffs.loc[high_in_tissue])]
for x in HT29_0p1percent:
    cpg_count_df[x] = [0.1, sum(amf_df.loc[high_in_tissue, x] > cutoffs.loc[high_in_tissue])]
for x in HT29_0p5percent:
    cpg_count_df[x] = [0.5, sum(amf_df.loc[high_in_tissue, x] > cutoffs.loc[high_in_tissue])]
for x in HT29_1percent:
    cpg_count_df[x] = [1, sum(amf_df.loc[high_in_tissue, x] > cutoffs.loc[high_in_tissue])]
for x in HT29_5percent:
    cpg_count_df[x] = [5, sum(amf_df.loc[high_in_tissue, x] > cutoffs.loc[high_in_tissue])]
for x in HT29_10percent:
    cpg_count_df[x] = [10, sum(amf_df.loc[high_in_tissue, x] > cutoffs.loc[high_in_tissue])]

cpg_count_df = pd.DataFrame(cpg_count_df).T
cpg_count_df.columns = ['Spike-In %', 'Number of CpG Sites']
cpg_count_df = cpg_count_df.sort_values('Spike-In %')
```

Plot Spike-In Counts

In [31]:
```
sns.swarmplot(x='Spike-In %', y='Number of CpG Sites', data=cpg_count_df)
sns.despine()
```

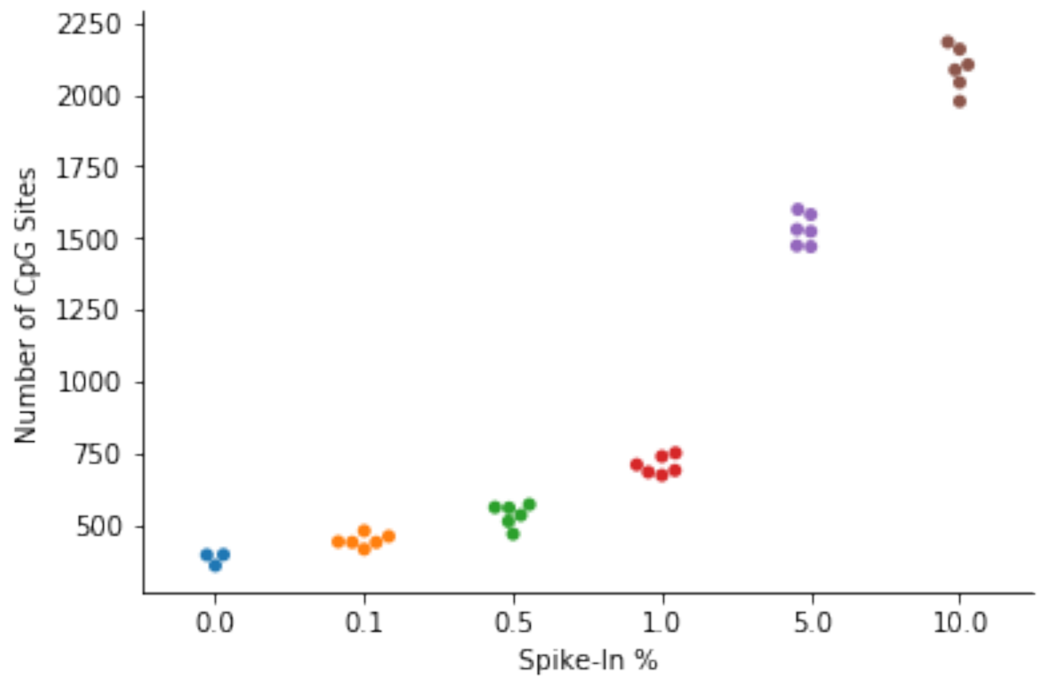

All spike-in samples could be detected.

Supplementary Note 5.
Code utilized to derive and implement an alternate Logistic Regression classifier that utilizes only genomic regions with higher read depth.A leave-in set of samples was used to build the classifier, and a leave-out set of samples was utilized to validate the classifier.

Supplementary Note 5

This analysis is similar to that of Supplementary Note 1; however, in this case all low-depth regions were excluded.

The leave-out set sensitivity and specificity were similar to Supplementary Note 1.

In [2]:
```
from __future__ import division
import sys
import os
import glob
import random
```

In [3]:
```
import pandas as pd
import numpy as np
import scipy
from sklearn.linear_model import LogisticRegression, LogisticRegressionCV
from sklearn.metrics import roc_curve, auc
from statsmodels.stats.multitest import multipletests
```

In [4]:
```
%matplotlib inline
import seaborn as sns
import matplotlib.pyplot as plt
```

In [5]:
```
sns.set()
sns.set_context('notebook', font_scale=1.2)
sns.set_style('white')
```

Sample Info

For this study, we processed two sets of samples; one set of plasma samples from the Taizhou Longitudinal Study, and a second set of commercially purchsed normal and cancer tissue samples from BioChain.

Taizhou Longitudinal Study

In [6]:
```
pre_diagnosis_samples = ['TSH-PL180123001', 'TSH-PL180123006', 'TSH-PL180123007', 'TSH-PL18012
3008', 'TSH-PL180123010', 'TSH-PL180123013', 'TSH-PL180123014', 'TSH-PL180123016', 'TSH-PL1801
23017', 'TSH-PL180123018', 'TSH-PL180123021', 'TSH-PL180123022', 'TSH-PL180123025', 'TSH-PL180
123027', 'TSH-PL180123029', 'TSH-PL180123030', 'TSH-PL180123031', 'TSH-PL180123033', 'TSH-PL18
0513004', 'TSH-PL180513005', 'TSH-PL180513006', 'TSH-PL180513007', 'TSH-PL180513011', 'TSH-PL1
80513012', 'TSH-PL180513013', 'TSH-PL180513014', 'TSH-PL180513016', 'TSH-PL180513017', 'TSH-PL
180513020', 'TSH-PL180513021', 'TSH-PL180513023', 'TSH-PL180513024', 'TSH-PL180513025', 'TSH-P
L180513026', 'TSH-PL180513027', 'TSH-PL180513029', 'TSH-PL180513030', 'TSH-PL180513034', 'TSH-
PL180513036', 'TSH-PL180513037', 'TSH-PL180513038', 'TSH-PL180513039', 'TSH-PL180513040', 'TSH
-PL180513041', 'TSH-PL180513043', 'TSH-PL180513045', 'TSH-PL180513049', 'TSH-PL180513050', 'TS
H-PL180513051', 'TSH-PL180513055', 'TSH-PL180513056', 'TSH-PL180513057', 'TSH-PL180513060', 'T
SH-PL180513062', 'TSH-PL180513066', 'TSH-PL180513067', 'TSH-PL180513068', 'TSH-PL180513069', '
TSH-PL180513070', 'TSH-PL180513071', 'TSH-PL180513072', 'TSH-PL180513073', 'TSH-PL180513075',
'TSH-PL180513076', 'TSH-PL180513077', 'TSH-PL180513078', 'TSH-PL180513079', 'TSH-PL180513080',
'TSH-PL180513081', 'TSH-PL180513084', 'TSH-PL180513085', 'TSH-PL180513087', 'TSH-PL180513088',
'TSH-PL180513089', 'TSH-PL180513091', 'TSH-PL180513092', 'TSH-PL180513093', 'TSH-PL180513095',
'TSH-PL180513097', 'TSH-PL180513098', 'TSH-PL180513099', 'TSH-PL180513100', 'TSH-PL180513101',
'TSH-PL180513102', 'TSH-PL180513104', 'TSH-PL180513106', 'TSH-PL180513108', 'TSH-PL180513109',
'TSH-PL180513110', 'TSH-PL180513111', 'TSH-PL180513114', 'TSH-PL180513115', 'TSH-PL180513117',
'TSH-PL180513118', 'TSH-PL180513119', 'TSH-PL180513121', 'TSH-PL180513122', 'TSH-PL180513123',
'TSH-PL180513124', 'TSH-PL180513127', 'TSH-PL180513128', 'TSH-PL180513129', 'TSH-PL180516001',
'TSH-PL180516002', 'TSH-PL180516003', 'TSH-PL180516004', 'TSH-PL180516006', 'TSH-PL180516007',
'TSH-PL180516008', 'TSH-PL180516009', 'TSH-PL180516011', 'TSH-PL180516012', 'TSH-PL180516013',
'TSH-PL180516014', 'TSH-PL180516016', 'TSH-PL180516018', 'TSH-PL180516019', 'TSH-PL180516020',
'TSH-PL180516021', 'TSH-PL180516023', 'TSH-PL180516027', 'TSH-PL180516028', 'TSH-PL180516030',
'TSH-PL180516031', 'TSH-PL180516032', 'TSH-PL180516036', 'TSH-PL180516038', 'TSH-PL180516039',
'TSH-PL180516040', 'TSH-PL180516042', 'TSH-PL180516043', 'TSH-PL180516044', 'TSH-PL180516045',
'TSH-PL180516049', 'TSH-PL180516051', 'TSH-PL180516053', 'TSH-PL180516054', 'TSH-PL180516055',
'TSH-PL180516056', 'TSH-PL180516057', 'TSH-PL180516059', 'TSH-PL180516062', 'TSH-PL180516063',
'TSH-PL180123002', 'TSH-PL180123004', 'TSH-PL180123005', 'TSH-PL180123012', 'TSH-PL180123024',
'TSH-PL180123032', 'TSH-PL180513001', 'TSH-PL180513002', 'TSH-PL180513003', 'TSH-PL180513009',
'TSH-PL180513010', 'TSH-PL180513018', 'TSH-PL180513019', 'TSH-PL180513022', 'TSH-PL180513028',
'TSH-PL180513031', 'TSH-PL180513032', 'TSH-PL180513033', 'TSH-PL180513035', 'TSH-PL180513044',
'TSH-PL180513058', 'TSH-PL180513064', 'TSH-PL180513083', 'TSH-PL180513086', 'TSH-PL180513090',
'TSH-PL180513096', 'TSH-PL180513103', 'TSH-PL180513105', 'TSH-PL180513112', 'TSH-PL180513116',
'TSH-PL180513125', 'TSH-PL180516005', 'TSH-PL180516010', 'TSH-PL180516015', 'TSH-PL180516017',
'TSH-PL180516025', 'TSH-PL180516026', 'TSH-PL180516029', 'TSH-PL180516033', 'TSH-PL180516034',
'TSH-PL180516046', 'TSH-PL180516047', 'TSH-PL180516048', 'TSH-PL180516050', 'TSH-PL180516052',
'TSH-PL180516058', 'TSH-PL180516060', 'TSH-PL180516061']
post_diagnosis_samples = ['TSH-PL171221101', 'TSH-PL171221112', 'TSH-PL171221149', 'TSH-PL1712
21150', 'TSH-PL171221160', 'TSH-PL171221172', 'TSH-PL171221179', 'TSH-PL171221180', 'TSH-PL180
504001', 'TSH-PL180504002', 'TSH-PL180504003', 'TSH-PL180504004', 'TSH-PL180504005', 'TSH-PL18
0504009', 'TSH-PL180504010', 'TSH-PL180504011', 'TSH-PL180504013', 'TSH-PL180504014', 'TSH-PL1
80504015', 'TSH-PL180504016', 'TSH-PL180504017', 'TSH-PL180504018', 'TSH-PL180504019', 'TSH-PL
180504021', 'TSH-PL180504022', 'TSH-PL180504027', 'TSH-PL180504028', 'TSH-PL180504030', 'TSH-P
L180504033', 'TSH-PL180504034', 'TSH-PL180504041', 'TSH-PL180504042', 'TSH-PL180504044', 'TSH-
PL180504046', 'TSH-PL180504047', 'TSH-PL180504049', 'TSH-PL180504050', 'TSH-PL180504053', 'TSH
-PL180504054', 'TSH-PL180504058', 'TSH-PL180504061', 'TSH-PL180504062', 'TSH-PL180504063', 'TS
H-PL180504065', 'TSH-PL180504068', 'TSH-PL180504070', 'TSH-PL180504074', 'TSH-PL180504081', 'T
SH-PL180504083', 'TSH-PL180504087', 'TSH-PL180504088', 'TSH-PL180504089', 'TSH-PL180504091', '
TSH-PL180504094', 'TSH-PL180504097', 'TSH-PL180504101', 'TSH-PL180504104', 'TSH-PL180504108',
'TSH-PL180504111', 'TSH-PL180504118', 'TSH-PL180508001', 'TSH-PL180508004', 'TSH-PL180508005',
'TSH-PL180508006', 'TSH-PL180508008', 'TSH-PL180508009', 'TSH-PL180508011', 'TSH-PL180508012',
'TSH-PL180508013', 'TSH-PL180508014', 'TSH-PL180508015', 'TSH-PL180508019', 'TSH-PL180508020',
'TSH-PL180508021', 'TSH-PL180508022', 'TSH-PL180508025', 'TSH-PL180508030', 'TSH-PL180508031',
'TSH-PL180508033', 'TSH-PL180508034', 'TSH-PL180508036', 'TSH-PL180508037', 'TSH-PL180508038',
'TSH-PL180508039', 'TSH-PL180508040', 'TSH-PL180508041', 'TSH-PL180508042', 'TSH-PL180508043',
'TSH-PL180508049', 'TSH-PL180508050', 'TSH-PL180508060', 'TSH-PL180508061', 'TSH-PL180508064',
'TSH-PL180508068', 'TSH-PL180508076', 'TSH-PL180508078', 'TSH-PL180508083', 'TSH-PL180508086',
'TSH-PL180508089', 'TSH-PL180510005', 'TSH-PL180510006', 'TSH-PL180510007', 'TSH-PL180510008',
'TSH-PL180510010', 'TSH-PL180510011', 'TSH-PL180510012', 'TSH-PL180510014', 'TSH-PL180510015',
'TSH-PL180510016', 'TSH-PL180510017', 'TSH-PL180510018', 'TSH-PL180510019', 'TSH-PL180510021',
'TSH-PL180510023', 'TSH-PL180510024', 'TSH-PL180510025', 'TSH-PL180510026', 'TSH-PL180510030',
'TSH-PL180510031', 'TSH-PL180510033', 'TSH-PL180510038', 'TSH-PL180510040', 'TSH-PL180510044',
'TSH-PL180510047', 'TSH-PL180510050', 'TSH-PL180510052', 'TSH-PL180510053', 'TSH-PL180510054',
'TSH-PL180510055', 'TSH-PL180510057', 'TSH-PL180510061', 'TSH-PL180510063', 'TSH-PL180510066',
'TSH-PL180510071', 'TSH-PL180510072', 'TSH-PL180510073', 'TSH-PL180510074', 'TSH-PL180510075',
'TSH-PL180510076', 'TSH-PL180510077', 'TSH-PL180510078', 'TSH-PL180510079', 'TSH-PL180510080',
'TSH-PL180510085', 'TSH-PL180510089', 'TSH-PL180510092', 'TSH-PL180510095', 'TSH-PL180510101',
'TSH-PL180510104', 'TSH-PL180510105', 'TSH-PL180510107', 'TSH-PL180510112', 'TSH-PL180510121',
'TSH-PL180510122', 'TSH-PL180510125', 'TSH-PL180510139', 'TSH-PL180510140', 'TSH-PL180510146',
'TSH-PL180510147', 'TSH-PL180510162', 'TSH-PL181205168', 'TSH-PL181205170', 'TSH-PL181205173',
'TSH-PL181205175', 'TSH-PL181205182', 'TSH-PL171221151', 'TSH-PL180504006', 'TSH-PL180504012',
'TSH-PL180504024', 'TSH-PL180504026', 'TSH-PL180504029', 'TSH-PL180504037', 'TSH-PL180504043',
'TSH-PL180504045', 'TSH-PL180504051', 'TSH-PL180504052', 'TSH-PL180504055', 'TSH-PL180504057',
'TSH-PL180504064', 'TSH-PL180504076', 'TSH-PL180504084', 'TSH-PL180504110', 'TSH-PL180508003',
'TSH-PL180508007', 'TSH-PL180508023', 'TSH-PL180508026', 'TSH-PL180508028', 'TSH-PL180508029',
'TSH-PL180508032', 'TSH-PL180508044', 'TSH-PL180508052', 'TSH-PL180508065', 'TSH-PL180508067',
'TSH-PL180508088', 'TSH-PL180510002', 'TSH-PL180510004', 'TSH-PL180510013', 'TSH-PL180510020',
'TSH-PL180510022', 'TSH-PL180510032', 'TSH-PL180510034', 'TSH-PL180510035', 'TSH-PL180510043',
'TSH-PL180510048', 'TSH-PL180510068', 'TSH-PL180510088', 'TSH-PL180510090', 'TSH-PL180510098',
'TSH-PL180510100', 'TSH-PL180510103', 'TSH-PL180510108', 'TSH-PL180510109', 'TSH-PL180510113',
'TSH-PL180510117', 'TSH-PL180510124', 'TSH-PL180510143', 'TSH-PL181205169', 'TSH-PL181205172',
'TSH-PL181205174', 'TSH-PL181205177', 'TSH-PL181205179', 'TSH-PL171221153', 'TSH-PL171221113']
healthy_samples = ['TSH-PL180309087', 'TSH-PL180316003', 'TSH-PL180316005', 'TSH-PL180316016',
'TSH-PL180316017', 'TSH-PL180316018', 'TSH-PL180316022', 'TSH-PL180316024', 'TSH-PL180316027',
'TSH-PL180316033', 'TSH-PL180316037', 'TSH-PL180316038', 'TSH-PL180316039', 'TSH-PL180316049',
'TSH-PL180316061', 'TSH-PL180316065', 'TSH-PL180316079', 'TSH-PL180316092', 'TSH-PL180316095',
'TSH-PL180316099', 'TSH-PL180316101', 'TSH-PL180316106', 'TSH-PL180316115', 'TSH-PL180316119',
'TSH-PL180316126', 'TSH-PL180316129', 'TSH-PL180316157', 'TSH-PL180316161', 'TSH-PL180316163',
'TSH-PL180316164', 'TSH-PL180316173', 'TSH-PL180316174', 'TSH-PL180316181', 'TSH-PL180316183',
'TSH-PL180316187', 'TSH-PL180316190', 'TSH-PL180316195', 'TSH-PL180316196', 'TSH-PL180316198',
'TSH-PL180323034', 'TSH-PL180323038', 'TSH-PL180323051', 'TSH-PL180323052', 'TSH-PL180323054',
'TSH-PL180323059', 'TSH-PL180323065', 'TSH-PL180323067', 'TSH-PL180323072', 'TSH-PL180323073',
'TSH-PL180323091', 'TSH-PL180323106', 'TSH-PL180323107', 'TSH-PL180323109', 'TSH-PL180323110',
'TSH-PL180323111', 'TSH-PL180323119', 'TSH-PL180323121', 'TSH-PL180323124', 'TSH-PL180323139',
'TSH-PL180323141', 'TSH-PL180323142', 'TSH-PL180323151', 'TSH-PL180323155', 'TSH-PL180323166',
'TSH-PL180410003', 'TSH-PL180410023', 'TSH-PL180410043', 'TSH-PL180410049', 'TSH-PL180410059',
'TSH-PL180410063', 'TSH-PL180410076', 'TSH-PL180410087', 'TSH-PL180410115', 'TSH-PL180410116',
'TSH-PL180410133', 'TSH-PL180410140', 'TSH-PL180410144', 'TSH-PL180410160', 'TSH-PL180410162',
'TSH-PL180410198', 'TSH-PL180417003', 'TSH-PL180417007', 'TSH-PL180417035', 'TSH-PL180417060',
'TSH-PL180417074', 'TSH-PL180417087', 'TSH-PL180417095', 'TSH-PL180417101', 'TSH-PL180417109',
'TSH-PL180417117', 'TSH-PL180417135', 'TSH-PL180417138', 'TSH-PL180417143', 'TSH-PL180417152',
'TSH-PL180417156', 'TSH-PL180417165', 'TSH-PL180417170', 'TSH-PL180417179', 'TSH-PL180417180',
'TSH-PL180417181', 'TSH-PL180417200', 'TSH-PL180424003', 'TSH-PL180424030', 'TSH-PL180424036',
'TSH-PL180424058', 'TSH-PL180424090', 'TSH-PL180424097', 'TSH-PL180424104', 'TSH-PL180424115',
'TSH-PL180424146', 'TSH-PL180424149', 'TSH-PL180424155', 'TSH-PL180424162', 'TSH-PL180424166',
'TSH-PL180424171', 'TSH-PL180703037', 'TSH-PL180703136', 'TSH-PL180703153', 'TSH-PL180703157',
'TSH-PL180703185', 'TSH-PL180706034', 'TSH-PL180706037', 'TSH-PL180706095', 'TSH-PL180706096',
'TSH-PL180706104', 'TSH-PL180706113', 'TSH-PL180706118', 'TSH-PL180706145', 'TSH-PL180706147',
'TSH-PL180706150', 'TSH-PL180706153', 'TSH-PL180706156', 'TSH-PL181128001', 'TSH-PL181128003',
'TSH-PL181128005', 'TSH-PL181128007', 'TSH-PL181128008', 'TSH-PL181128009', 'TSH-PL181128011',
'TSH-PL181128012', 'TSH-PL181128014', 'TSH-PL181128015', 'TSH-PL181128019', 'TSH-PL181128021',
'TSH-PL181128022', 'TSH-PL181128023', 'TSH-PL181128025', 'TSH-PL181128027', 'TSH-PL181128029',
'TSH-PL181128030', 'TSH-PL181128033', 'TSH-PL181128035', 'TSH-PL181128036', 'TSH-PL181128038',
'TSH-PL181128039', 'TSH-PL181128040', 'TSH-PL181128041', 'TSH-PL181128042', 'TSH-PL181128043',
'TSH-PL181128045', 'TSH-PL181128047', 'TSH-PL181128048', 'TSH-PL181128049', 'TSH-PL181128050',
'TSH-PL181128051', 'TSH-PL181128054', 'TSH-PL181128055', 'TSH-PL181128056', 'TSH-PL181128057',
'TSH-PL181128058', 'TSH-PL181128061', 'TSH-PL181128062', 'TSH-PL181128064', 'TSH-PL181128065',
'TSH-PL181128066', 'TSH-PL181128070', 'TSH-PL181128073', 'TSH-PL181128077', 'TSH-PL181128080',
'TSH-PL181128082', 'TSH-PL181128083', 'TSH-PL181128085', 'TSH-PL181128086', 'TSH-PL181128093',
'TSH-PL181128096', 'TSH-PL181128099', 'TSH-PL181128101', 'TSH-PL181128102', 'TSH-PL181128103',
'TSH-PL181128104', 'TSH-PL181128107', 'TSH-PL181128109', 'TSH-PL181128110', 'TSH-PL181128111',
'TSH-PL181128112', 'TSH-PL181128114', 'TSH-PL181128115', 'TSH-PL181128117', 'TSH-PL181128118',
'TSH-PL181128120', 'TSH-PL181128122', 'TSH-PL181128123', 'TSH-PL181128124', 'TSH-PL181128125',
'TSH-PL181128126', 'TSH-PL181128127', 'TSH-PL181205002', 'TSH-PL181205003', 'TSH-PL181205005',
'TSH-PL181205006', 'TSH-PL181205008', 'TSH-PL181205009', 'TSH-PL181205013', 'TSH-PL181205014',
'TSH-PL181205016', 'TSH-PL181205020', 'TSH-PL181205022', 'TSH-PL181205024', 'TSH-PL181205026',
'TSH-PL181205027', 'TSH-PL181205028', 'TSH-PL181205030', 'TSH-PL181205033', 'TSH-PL181205035',
'TSH-PL181205040', 'TSH-PL181205041', 'TSH-PL181205042', 'TSH-PL181205043', 'TSH-PL181205044',
'TSH-PL181205045', 'TSH-PL181205046', 'TSH-PL181205047', 'TSH-PL181205048', 'TSH-PL181205049',
'TSH-PL181205051', 'TSH-PL181205053', 'TSH-PL181205054', 'TSH-PL181205055', 'TSH-PL181205057',
'TSH-PL181205058', 'TSH-PL181205059', 'TSH-PL181205060', 'TSH-PL181205061', 'TSH-PL181205062',
'TSH-PL181205063', 'TSH-PL181205064', 'TSH-PL181205065', 'TSH-PL181205066', 'TSH-PL181205068',
'TSH-PL181205069', 'TSH-PL181205072', 'TSH-PL181205074', 'TSH-PL181205075', 'TSH-PL181205076',
'TSH-PL181205077', 'TSH-PL181205078', 'TSH-PL181205079', 'TSH-PL181205081', 'TSH-PL181205082',
'TSH-PL181205083', 'TSH-PL181205085', 'TSH-PL181205086', 'TSH-PL181205087', 'TSH-PL181205088',
'TSH-PL181205090', 'TSH-PL181205091', 'TSH-PL181205093', 'TSH-PL181205095', 'TSH-PL181205096',
'TSH-PL181205097', 'TSH-PL181205098', 'TSH-PL181205099', 'TSH-PL181205102', 'TSH-PL181205103',
'TSH-PL181205104', 'TSH-PL181205107', 'TSH-PL181205108', 'TSH-PL181205110', 'TSH-PL181205113',
'TSH-PL181205115', 'TSH-PL181205116', 'TSH-PL181205117', 'TSH-PL181205121', 'TSH-PL181205122',
'TSH-PL181205125', 'TSH-PL181205128', 'TSH-PL181205129', 'TSH-PL181205140', 'TSH-PL181205141',
'TSH-PL181205142', 'TSH-PL181205143', 'TSH-PL181205144', 'TSH-PL181205145', 'TSH-PL181205146',
'TSH-PL181205147', 'TSH-PL181205148', 'TSH-PL181205149', 'TSH-PL181205150', 'TSH-PL181205151',
'TSH-PL181205152', 'TSH-PL181205155', 'TSH-PL181205156', 'TSH-PL181205157', 'TSH-PL181205158',
'TSH-PL181205160', 'TSH-PL181205161', 'TSH-PL181205162', 'TSH-PL181205164', 'TSH-PL181205165',
'TSH-PL181205166', 'TSH-PL180309082', 'TSH-PL180316013', 'TSH-PL180316029', 'TSH-PL180316075',
'TSH-PL180316102', 'TSH-PL180316112', 'TSH-PL180316134', 'TSH-PL180316138', 'TSH-PL180316175',
'TSH-PL180316178', 'TSH-PL180316200', 'TSH-PL180323029', 'TSH-PL180323032', 'TSH-PL180323053',
'TSH-PL180323068', 'TSH-PL180323074', 'TSH-PL180323088', 'TSH-PL180323097', 'TSH-PL180323103',
'TSH-PL180323115', 'TSH-PL180323120', 'TSH-PL180323160', 'TSH-PL180323175', 'TSH-PL180410058',
'TSH-PL180410070', 'TSH-PL180410081', 'TSH-PL180410148', 'TSH-PL180410161', 'TSH-PL180417017',
'TSH-PL180417023', 'TSH-PL180417045', 'TSH-PL180417077', 'TSH-PL180417104', 'TSH-PL180417111',
'TSH-PL180417124', 'TSH-PL180417162', 'TSH-PL180417169', 'TSH-PL180417199', 'TSH-PL180424042',
'TSH-PL180424088', 'TSH-PL180424138', 'TSH-PL180703012', 'TSH-PL180703127', 'TSH-PL180703134',
'TSH-PL180703141', 'TSH-PL180703168', 'TSH-PL180706117', 'TSH-PL181128004', 'TSH-PL181128006',
'TSH-PL181128013', 'TSH-PL181128018', 'TSH-PL181128020', 'TSH-PL181128031', 'TSH-PL181128037',
'TSH-PL181128067', 'TSH-PL181128071', 'TSH-PL181128078', 'TSH-PL181128084', 'TSH-PL181128095',
'TSH-PL181128097', 'TSH-PL181128098', 'TSH-PL181128105', 'TSH-PL181128106', 'TSH-PL181128108',
'TSH-PL181128113', 'TSH-PL181128116', 'TSH-PL181205001', 'TSH-PL181205004', 'TSH-PL181205007',
'TSH-PL181205010', 'TSH-PL181205011', 'TSH-PL181205012', 'TSH-PL181205015', 'TSH-PL181205018',
'TSH-PL181205019', 'TSH-PL181205023', 'TSH-PL181205031', 'TSH-PL181205032', 'TSH-PL181205036',
'TSH-PL181205037', 'TSH-PL181205052', 'TSH-PL181205056', 'TSH-PL181205067', 'TSH-PL181205070',
'TSH-PL181205071', 'TSH-PL181205073', 'TSH-PL181205080', 'TSH-PL181205084', 'TSH-PL181205089',
'TSH-PL181205092', 'TSH-PL181205094', 'TSH-PL181205100', 'TSH-PL181205101', 'TSH-PL181205106',
'TSH-PL181205112', 'TSH-PL181205118', 'TSH-PL181205119', 'TSH-PL181205120', 'TSH-PL181205123',
'TSH-PL181205124', 'TSH-PL181205153', 'TSH-PL181205154', 'TSH-PL181205159', 'TSH-PL181205163']
```

In [7]:
```
healthy_samples = ['TSH-PL180309087', 'TSH-PL180316003', 'TSH-PL180316005', 'TSH-PL180316016',
'TSH-PL180316017', 'TSH-PL180316018', 'TSH-PL180316022', 'TSH-PL180316024', 'TSH-PL180316027',
'TSH-PL180316033', 'TSH-PL180316037', 'TSH-PL180316038', 'TSH-PL180316039', 'TSH-PL180316049',
'TSH-PL180316061', 'TSH-PL180316065', 'TSH-PL180316079', 'TSH-PL180316092', 'TSH-PL180316095',
'TSH-PL180316099', 'TSH-PL180316101', 'TSH-PL180316106', 'TSH-PL180316115', 'TSH-PL180316119',
'TSH-PL180316126', 'TSH-PL180316129', 'TSH-PL180316157', 'TSH-PL180316161', 'TSH-PL180316163',
'TSH-PL180316164', 'TSH-PL180316173', 'TSH-PL180316174', 'TSH-PL180316181', 'TSH-PL180316183',
'TSH-PL180316187', 'TSH-PL180316190', 'TSH-PL180316195', 'TSH-PL180316196', 'TSH-PL180316198',
'TSH-PL180323034', 'TSH-PL180323038', 'TSH-PL180323051', 'TSH-PL180323052', 'TSH-PL180323054',
'TSH-PL180323059', 'TSH-PL180323065', 'TSH-PL180323067', 'TSH-PL180323072', 'TSH-PL180323073',
'TSH-PL180323091', 'TSH-PL180323106', 'TSH-PL180323107', 'TSH-PL180323109', 'TSH-PL180323110',
'TSH-PL180323111', 'TSH-PL180323119', 'TSH-PL180323121', 'TSH-PL180323124', 'TSH-PL180323139',
'TSH-PL180323141', 'TSH-PL180323142', 'TSH-PL180323151', 'TSH-PL180323155', 'TSH-PL180323166',
'TSH-PL180410003', 'TSH-PL180410023', 'TSH-PL180410043', 'TSH-PL180410049', 'TSH-PL180410059',
'TSH-PL180410063', 'TSH-PL180410076', 'TSH-PL180410087', 'TSH-PL180410115', 'TSH-PL180410116',
'TSH-PL180410133', 'TSH-PL180410140', 'TSH-PL180410144', 'TSH-PL180410160', 'TSH-PL180410162',
'TSH-PL180410198', 'TSH-PL180417003', 'TSH-PL180417007', 'TSH-PL180417035', 'TSH-PL180417060',
'TSH-PL180417074', 'TSH-PL180417087', 'TSH-PL180417095', 'TSH-PL180417101', 'TSH-PL180417109',
'TSH-PL180417117', 'TSH-PL180417135', 'TSH-PL180417138', 'TSH-PL180417143', 'TSH-PL180417152',
'TSH-PL180417156', 'TSH-PL180417165', 'TSH-PL180417170', 'TSH-PL180417179', 'TSH-PL180417180',
'TSH-PL180417181', 'TSH-PL180417200', 'TSH-PL180424003', 'TSH-PL180424030', 'TSH-PL180424036',
'TSH-PL180424058', 'TSH-PL180424090', 'TSH-PL180424097', 'TSH-PL180424104', 'TSH-PL180424115',
'TSH-PL180424146', 'TSH-PL180424149', 'TSH-PL180424155', 'TSH-PL180424162', 'TSH-PL180424166',
'TSH-PL180424171', 'TSH-PL180703037', 'TSH-PL180703136', 'TSH-PL180703153', 'TSH-PL180703157',
'TSH-PL180703185', 'TSH-PL180706034', 'TSH-PL180706037', 'TSH-PL180706095', 'TSH-PL180706096',
'TSH-PL180706104', 'TSH-PL180706113', 'TSH-PL180706118', 'TSH-PL180706145', 'TSH-PL180706147',
'TSH-PL180706150', 'TSH-PL180706153', 'TSH-PL180706156', 'TSH-PL181128001', 'TSH-PL181128003',
'TSH-PL181128005', 'TSH-PL181128007', 'TSH-PL181128008', 'TSH-PL181128009', 'TSH-PL181128011',
'TSH-PL181128012', 'TSH-PL181128014', 'TSH-PL181128015', 'TSH-PL181128019', 'TSH-PL181128021',
'TSH-PL181128022', 'TSH-PL181128023', 'TSH-PL181128025', 'TSH-PL181128027', 'TSH-PL181128029',
'TSH-PL181128030', 'TSH-PL181128033', 'TSH-PL181128035', 'TSH-PL181128036', 'TSH-PL181128038',
'TSH-PL181128039', 'TSH-PL181128040', 'TSH-PL181128041', 'TSH-PL181128042', 'TSH-PL181128043',
'TSH-PL181128045', 'TSH-PL181128047', 'TSH-PL181128048', 'TSH-PL181128049', 'TSH-PL181128050',
'TSH-PL181128051', 'TSH-PL181128054', 'TSH-PL181128055', 'TSH-PL181128056', 'TSH-PL181128057',
'TSH-PL181128058', 'TSH-PL181128061', 'TSH-PL181128062', 'TSH-PL181128064', 'TSH-PL181128065',
'TSH-PL181128066', 'TSH-PL181128070', 'TSH-PL181128073', 'TSH-PL181128077', 'TSH-PL181128080',
'TSH-PL181128082', 'TSH-PL181128083', 'TSH-PL181128085', 'TSH-PL181128086', 'TSH-PL181128093',
'TSH-PL181128096', 'TSH-PL181128099', 'TSH-PL181128101', 'TSH-PL181128102', 'TSH-PL181128103',
'TSH-PL181128104', 'TSH-PL181128107', 'TSH-PL181128109', 'TSH-PL181128110', 'TSH-PL181128111',
'TSH-PL181128112', 'TSH-PL181128114', 'TSH-PL181128115', 'TSH-PL181128117', 'TSH-PL181128118',
'TSH-PL181128120', 'TSH-PL181128122', 'TSH-PL181128123', 'TSH-PL181128124', 'TSH-PL181128125',
'TSH-PL181128126', 'TSH-PL181128127', 'TSH-PL181205002', 'TSH-PL181205003', 'TSH-PL181205005',
'TSH-PL181205006', 'TSH-PL181205008', 'TSH-PL181205009', 'TSH-PL181205013', 'TSH-PL181205014',
'TSH-PL181205016', 'TSH-PL181205020', 'TSH-PL181205022', 'TSH-PL181205024', 'TSH-PL181205026',
'TSH-PL181205027', 'TSH-PL181205028', 'TSH-PL181205030', 'TSH-PL181205033', 'TSH-PL181205035',
'TSH-PL181205040', 'TSH-PL181205041', 'TSH-PL181205042', 'TSH-PL181205043', 'TSH-PL181205044',
'TSH-PL181205045', 'TSH-PL181205046', 'TSH-PL181205047', 'TSH-PL181205048', 'TSH-PL181205049',
'TSH-PL181205051', 'TSH-PL181205053', 'TSH-PL181205054', 'TSH-PL181205055', 'TSH-PL181205057',
'TSH-PL181205058', 'TSH-PL181205059', 'TSH-PL181205060', 'TSH-PL181205061', 'TSH-PL181205062',
'TSH-PL181205063', 'TSH-PL181205064', 'TSH-PL181205065', 'TSH-PL181205066', 'TSH-PL181205068',
'TSH-PL181205069', 'TSH-PL181205072', 'TSH-PL181205074', 'TSH-PL181205075', 'TSH-PL181205076',
'TSH-PL181205077', 'TSH-PL181205078', 'TSH-PL181205079', 'TSH-PL181205081', 'TSH-PL181205082',
'TSH-PL181205083', 'TSH-PL181205085', 'TSH-PL181205086', 'TSH-PL181205087', 'TSH-PL181205088',
'TSH-PL181205090', 'TSH-PL181205091', 'TSH-PL181205093', 'TSH-PL181205095', 'TSH-PL181205096',
'TSH-PL181205097', 'TSH-PL181205098', 'TSH-PL181205099', 'TSH-PL181205102', 'TSH-PL181205103',
'TSH-PL181205104', 'TSH-PL181205107', 'TSH-PL181205108', 'TSH-PL181205110', 'TSH-PL181205113',
'TSH-PL181205115', 'TSH-PL181205116', 'TSH-PL181205117', 'TSH-PL181205121', 'TSH-PL181205122',
'TSH-PL181205125', 'TSH-PL181205128', 'TSH-PL181205129', 'TSH-PL181205140', 'TSH-PL181205141',
'TSH-PL181205142', 'TSH-PL181205143', 'TSH-PL181205144', 'TSH-PL181205145', 'TSH-PL181205146',
'TSH-PL181205147', 'TSH-PL181205148', 'TSH-PL181205149', 'TSH-PL181205150', 'TSH-PL181205151',
'TSH-PL181205152', 'TSH-PL181205155', 'TSH-PL181205156', 'TSH-PL181205157', 'TSH-PL181205158',
'TSH-PL181205160', 'TSH-PL181205161', 'TSH-PL181205162', 'TSH-PL181205164', 'TSH-PL181205165',
'TSH-PL181205166', 'TSH-PL180309082', 'TSH-PL180316013', 'TSH-PL180316029', 'TSH-PL180316075',
'TSH-PL180316102', 'TSH-PL180316112', 'TSH-PL180316134', 'TSH-PL180316138', 'TSH-PL180316175',
'TSH-PL180316178', 'TSH-PL180316200', 'TSH-PL180323029', 'TSH-PL180323032', 'TSH-PL180323053',
'TSH-PL180323068', 'TSH-PL180323074', 'TSH-PL180323088', 'TSH-PL180323097', 'TSH-PL180323103',
'TSH-PL180323115', 'TSH-PL180323120', 'TSH-PL180323160', 'TSH-PL180323175', 'TSH-PL180410058',
'TSH-PL180410070', 'TSH-PL180410081', 'TSH-PL180410148', 'TSH-PL180410161', 'TSH-PL180417017',
'TSH-PL180417023', 'TSH-PL180417045', 'TSH-PL180417077', 'TSH-PL180417104', 'TSH-PL180417111',
'TSH-PL180417124', 'TSH-PL180417162', 'TSH-PL180417169', 'TSH-PL180417199', 'TSH-PL180424042',
'TSH-PL180424088', 'TSH-PL180424138', 'TSH-PL180703012', 'TSH-PL180703127', 'TSH-PL180703134',
'TSH-PL180703141', 'TSH-PL180703168', 'TSH-PL180706117', 'TSH-PL181128004', 'TSH-PL181128006',
'TSH-PL181128013', 'TSH-PL181128018', 'TSH-PL181128020', 'TSH-PL181128031', 'TSH-PL181128037',
'TSH-PL181128067', 'TSH-PL181128071', 'TSH-PL181128078', 'TSH-PL181128084', 'TSH-PL181128095',
'TSH-PL181128097', 'TSH-PL181128098', 'TSH-PL181128105', 'TSH-PL181128106', 'TSH-PL181128108',
'TSH-PL181128113', 'TSH-PL181128116', 'TSH-PL181205001', 'TSH-PL181205004', 'TSH-PL181205007',
'TSH-PL181205010', 'TSH-PL181205011', 'TSH-PL181205012', 'TSH-PL181205015', 'TSH-PL181205018',
'TSH-PL181205019', 'TSH-PL181205023', 'TSH-PL181205031', 'TSH-PL181205032', 'TSH-PL181205036',
'TSH-PL181205037', 'TSH-PL181205052', 'TSH-PL181205056', 'TSH-PL181205067', 'TSH-PL181205070',
'TSH-PL181205071', 'TSH-PL181205073', 'TSH-PL181205080', 'TSH-PL181205084', 'TSH-PL181205089',
'TSH-PL181205092', 'TSH-PL181205094', 'TSH-PL181205100', 'TSH-PL181205101', 'TSH-PL181205106',
'TSH-PL181205112', 'TSH-PL181205118', 'TSH-PL181205119', 'TSH-PL181205120', 'TSH-PL181205123',
'TSH-PL181205124', 'TSH-PL181205153', 'TSH-PL181205154', 'TSH-PL181205159', 'TSH-PL181205163']

post_diagnosis_samples_colon = ['TSH-PL180510139', 'TSH-PL180510140', 'TSH-PL180510146', 'TSH-
PL180510147', 'TSH-PL180510162', 'TSH-PL181205175', 'TSH-PL180510143']
post_diagnosis_samples_liver = ['TSH-PL180510085', 'TSH-PL180510089', 'TSH-PL180510092', 'TSH-
PL180510095', 'TSH-PL180510101', 'TSH-PL180510104', 'TSH-PL180510105', 'TSH-PL180510107', 'TSH
-PL180510112', 'TSH-PL180510121', 'TSH-PL180510122', 'TSH-PL180510125', 'TSH-PL181205173', 'TS
H-PL180510088', 'TSH-PL180510090', 'TSH-PL180510098', 'TSH-PL180510100', 'TSH-PL180510103', 'T
SH-PL180510108', 'TSH-PL180510109', 'TSH-PL180510113', 'TSH-PL180510117', 'TSH-PL180510124']
post_diagnosis_samples_esophagus = ['TSH-PL171221101', 'TSH-PL171221112', 'TSH-PL171221149', '
TSH-PL171221150', 'TSH-PL171221160', 'TSH-PL171221172', 'TSH-PL171221179', 'TSH-PL171221180',
'TSH-PL180508001', 'TSH-PL180508004', 'TSH-PL180508005', 'TSH-PL180508006', 'TSH-PL180508008',
'TSH-PL180508009', 'TSH-PL180508011', 'TSH-PL180508012', 'TSH-PL180508013', 'TSH-PL180508014',
'TSH-PL180508015', 'TSH-PL180508019', 'TSH-PL180508020', 'TSH-PL180508021', 'TSH-PL180508022',
'TSH-PL180508025', 'TSH-PL180508030', 'TSH-PL180508031', 'TSH-PL180508033', 'TSH-PL180508034',
'TSH-PL180508036', 'TSH-PL180508037', 'TSH-PL180508038', 'TSH-PL180508039', 'TSH-PL180508040',
'TSH-PL180508041', 'TSH-PL180508042', 'TSH-PL180508043', 'TSH-PL180508049', 'TSH-PL180508050',
'TSH-PL180508060', 'TSH-PL180508061', 'TSH-PL180508064', 'TSH-PL180508068', 'TSH-PL180508076',
'TSH-PL180508078', 'TSH-PL180508083', 'TSH-PL180508086', 'TSH-PL180508089', 'TSH-PL180510044',
'TSH-PL180510061', 'TSH-PL181205168', 'TSH-PL181205170', 'TSH-PL171221151', 'TSH-PL180508003',
'TSH-PL180508007', 'TSH-PL180508023', 'TSH-PL180508026', 'TSH-PL180508028', 'TSH-PL180508029',
'TSH-PL180508032', 'TSH-PL180508044', 'TSH-PL180508052', 'TSH-PL180508065', 'TSH-PL180508067',
'TSH-PL180508088', 'TSH-PL181205172', 'TSH-PL181205174', 'TSH-PL171221153', 'TSH-PL171221113']
post_diagnosis_samples_lung = ['TSH-PL180510005', 'TSH-PL180510006', 'TSH-PL180510007', 'TSH-P
L180510008', 'TSH-PL180510010', 'TSH-PL180510011', 'TSH-PL180510012', 'TSH-PL180510014', 'TSH-
PL180510015', 'TSH-PL180510016', 'TSH-PL180510017', 'TSH-PL180510018', 'TSH-PL180510019', 'TSH
-PL180510021', 'TSH-PL180510023', 'TSH-PL180510024', 'TSH-PL180510025', 'TSH-PL180510026', 'TS
H-PL180510030', 'TSH-PL180510031', 'TSH-PL180510033', 'TSH-PL180510038', 'TSH-PL180510040', 'T
SH-PL180510047', 'TSH-PL180510050', 'TSH-PL180510052', 'TSH-PL180510053', 'TSH-PL180510054', '
TSH-PL180510055', 'TSH-PL180510057', 'TSH-PL180510063', 'TSH-PL180510066', 'TSH-PL180510071',
'TSH-PL180510072', 'TSH-PL180510073', 'TSH-PL180510074', 'TSH-PL180510075', 'TSH-PL180510076',
'TSH-PL180510077', 'TSH-PL180510078', 'TSH-PL180510079', 'TSH-PL180510080', 'TSH-PL181205182',
'TSH-PL180510002', 'TSH-PL180510004', 'TSH-PL180510013', 'TSH-PL180510020', 'TSH-PL180510022',
'TSH-PL180510032', 'TSH-PL180510034', 'TSH-PL180510035', 'TSH-PL180510043', 'TSH-PL180510048',
'TSH-PL180510068', 'TSH-PL181205177', 'TSH-PL181205179']
post_diagnosis_samples_stomach = ['TSH-PL180504001', 'TSH-PL180504002', 'TSH-PL180504003', 'TS
H-PL180504004', 'TSH-PL180504005', 'TSH-PL180504009', 'TSH-PL180504010', 'TSH-PL180504011', 'T
SH-PL180504013', 'TSH-PL180504014', 'TSH-PL180504015', 'TSH-PL180504016', 'TSH-PL180504017', '
TSH-PL180504018', 'TSH-PL180504019', 'TSH-PL180504021', 'TSH-PL180504022', 'TSH-PL180504027',
'TSH-PL180504028', 'TSH-PL180504030', 'TSH-PL180504033', 'TSH-PL180504034', 'TSH-PL180504041',
'TSH-PL180504042', 'TSH-PL180504044', 'TSH-PL180504046', 'TSH-PL180504047', 'TSH-PL180504049',
'TSH-PL180504050', 'TSH-PL180504053', 'TSH-PL180504054', 'TSH-PL180504058', 'TSH-PL180504061',
'TSH-PL180504062', 'TSH-PL180504063', 'TSH-PL180504065', 'TSH-PL180504068', 'TSH-PL180504070',
'TSH-PL180504074', 'TSH-PL180504081', 'TSH-PL180504083', 'TSH-PL180504087', 'TSH-PL180504088',
'TSH-PL180504089', 'TSH-PL180504091', 'TSH-PL180504094', 'TSH-PL180504097', 'TSH-PL180504101',
'TSH-PL180504104', 'TSH-PL180504108', 'TSH-PL180504111', 'TSH-PL180504118', 'TSH-PL180504006',
'TSH-PL180504012', 'TSH-PL180504024', 'TSH-PL180504026', 'TSH-PL180504029', 'TSH-PL180504037',
'TSH-PL180504043', 'TSH-PL180504045', 'TSH-PL180504051', 'TSH-PL180504052', 'TSH-PL180504055',
'TSH-PL180504057', 'TSH-PL180504064', 'TSH-PL180504076', 'TSH-PL180504084', 'TSH-PL180504110',
'TSH-PL181205169']

pre_diagnosis_samples_colon = ['TSH-PL180123001', 'TSH-PL180123010', 'TSH-PL180513013', 'TSH-P
L180513014', 'TSH-PL180513020', 'TSH-PL180513027', 'TSH-PL180513029', 'TSH-PL180513030', 'TSH-
PL180513034', 'TSH-PL180513037', 'TSH-PL180513049', 'TSH-PL180513062', 'TSH-PL180513077', 'TSH
-PL180513078', 'TSH-PL180513085', 'TSH-PL180513114', 'TSH-PL180516003', 'TSH-PL180516011', 'TS
H-PL180516012', 'TSH-PL180516014', 'TSH-PL180516018', 'TSH-PL180516021', 'TSH-PL180516028', 'T
SH-PL180516039', 'TSH-PL180516044', 'TSH-PL180516053', 'TSH-PL180516057', 'TSH-PL180513001', '
TSH-PL180513002', 'TSH-PL180513064', 'TSH-PL180513116', 'TSH-PL180516015', 'TSH-PL180516017',
'TSH-PL180516026', 'TSH-PL180516033']
pre_diagnosis_samples_liver = ['TSH-PL180123014', 'TSH-PL180123031', 'TSH-PL180513006', 'TSH-P
L180513011', 'TSH-PL180513017', 'TSH-PL180513021', 'TSH-PL180513026', 'TSH-PL180513041', 'TSH-
PL180513043', 'TSH-PL180513071', 'TSH-PL180513088', 'TSH-PL180513095', 'TSH-PL180513098', 'TSH
-PL180513108', 'TSH-PL180513109', 'TSH-PL180513111', 'TSH-PL180513117', 'TSH-PL180516006', 'TS
H-PL180516038', 'TSH-PL180516040', 'TSH-PL180516051', 'TSH-PL180516054', 'TSH-PL180516063', 'T
SH-PL180123004', 'TSH-PL180123005', 'TSH-PL180513009', 'TSH-PL180513033', 'TSH-PL180513090', '
TSH-PL180516025']
pre_diagnosis_samples_esophagus = ['TSH-PL180123006', 'TSH-PL180123007', 'TSH-PL180123008', 'T
SH-PL180123016', 'TSH-PL180123025', 'TSH-PL180513004', 'TSH-PL180513005', 'TSH-PL180513016', '
TSH-PL180513051', 'TSH-PL180513068', 'TSH-PL180513072', 'TSH-PL180513075', 'TSH-PL180513080',
'TSH-PL180513081', 'TSH-PL180513091', 'TSH-PL180513093', 'TSH-PL180513097', 'TSH-PL180513100',
'TSH-PL180513101', 'TSH-PL180513110', 'TSH-PL180513115', 'TSH-PL180513118', 'TSH-PL180513121',
'TSH-PL180513122', 'TSH-PL180513124', 'TSH-PL180513128', 'TSH-PL180513129', 'TSH-PL180516013',
'TSH-PL180516020', 'TSH-PL180516030', 'TSH-PL180516031', 'TSH-PL180516045', 'TSH-PL180516049',
'TSH-PL180516059', 'TSH-PL180123024', 'TSH-PL180123032', 'TSH-PL180513028', 'TSH-PL180513032',
'TSH-PL180513058', 'TSH-PL180513083', 'TSH-PL180513096', 'TSH-PL180516005', 'TSH-PL180516047',
'TSH-PL180516050', 'TSH-PL180516061']
pre_diagnosis_samples_lung = ['TSH-PL180123013', 'TSH-PL180123021', 'TSH-PL180123022', 'TSH-PL
180123029', 'TSH-PL180123030', 'TSH-PL180513007', 'TSH-PL180513012', 'TSH-PL180513023', 'TSH-P
L180513024', 'TSH-PL180513036', 'TSH-PL180513040', 'TSH-PL180513045', 'TSH-PL180513055', 'TSH-
PL180513056', 'TSH-PL180513057', 'TSH-PL180513067', 'TSH-PL180513069', 'TSH-PL180513079', 'TSH
-PL180513084', 'TSH-PL180513087', 'TSH-PL180513092', 'TSH-PL180513106', 'TSH-PL180516001', 'TS
H-PL180516002', 'TSH-PL180516004', 'TSH-PL180516016', 'TSH-PL180516023', 'TSH-PL180516032', 'T
SH-PL180516055', 'TSH-PL180516056', 'TSH-PL180516062', 'TSH-PL180123002', 'TSH-PL180123012', '
TSH-PL180513010', 'TSH-PL180513019', 'TSH-PL180513022', 'TSH-PL180513031', 'TSH-PL180513035',
'TSH-PL180513044', 'TSH-PL180513086', 'TSH-PL180513112', 'TSH-PL180516029', 'TSH-PL180516034',
'TSH-PL180516046', 'TSH-PL180516048', 'TSH-PL180516058', 'TSH-PL180516060']
pre_diagnosis_samples_stomach = ['TSH-PL180123017', 'TSH-PL180123018', 'TSH-PL180123027', 'TSH
-PL180123033', 'TSH-PL180513025', 'TSH-PL180513038', 'TSH-PL180513039', 'TSH-PL180513050', 'TS
H-PL180513060', 'TSH-PL180513066', 'TSH-PL180513070', 'TSH-PL180513073', 'TSH-PL180513076', 'T
SH-PL180513089', 'TSH-PL180513099', 'TSH-PL180513102', 'TSH-PL180513104', 'TSH-PL180513119', '
TSH-PL180513123', 'TSH-PL180513127', 'TSH-PL180516007', 'TSH-PL180516008', 'TSH-PL180516009',
'TSH-PL180516019', 'TSH-PL180516027', 'TSH-PL180516036', 'TSH-PL180516042', 'TSH-PL180516043',
'TSH-PL180513003', 'TSH-PL180513018', 'TSH-PL180513103', 'TSH-PL180513105', 'TSH-PL180513125',
'TSH-PL180516010', 'TSH-PL180516052']
```

BioChain Tissue Samples

In [8]:
```
healthy_lung_tissue = ['BC_Lung_ND1', 'BC_Lung_ND2', 'BC_Lung_ND3', 'BC_Lung_ND4', 'BC_Lung_ND
5', 'BC_Lung_ND6', 'BC_Lung_ND7', 'BC_Lung_ND8']
healthy_colon_tissue = ['BC_CRC_ND1', 'BC_CRC_ND2', 'BC_CRC_ND3', 'BC_CRC_ND4', 'BC_CRC_ND5',
'BC_CRC_ND6', 'BC_CRC_ND7', 'BC_CRC_ND8']
healthy_stomach_tissue = ['BC_Stomach_ND1', 'BC_Stomach_ND2', 'BC_Stomach_ND3', 'BC_Stomach_ND
4', 'BC_Stomach_ND5', 'BC_Stomach_ND6', 'BC_Stomach_ND7', 'BC_Stomach_ND8']
healthy_breast_tissue = ['BC_Breast_ND1', 'BC_Breast_ND2', 'BC_Breast_ND3', 'BC_Breast_ND4', '
BC_Breast_ND5', 'BC_Breast_ND6', 'BC_Breast_ND7', 'BC_Breast_ND8']

cancer_lung_tissue = ['BC_Lung_TD1', 'BC_Lung_TD10', 'BC_Lung_TD11', 'BC_Lung_TD12', 'BC_Lung_
TD13', 'BC_Lung_TD14', 'BC_Lung_TD15', 'BC_Lung_TD16', 'BC_Lung_TD17', 'BC_Lung_TD18', 'BC_Lun
g_TD19', 'BC_Lung_TD2', 'BC_Lung_TD20', 'BC_Lung_TD21', 'BC_Lung_TD22', 'BC_Lung_TD23', 'BC_Lu
ng_TD24', 'BC_Lung_TD25', 'BC_Lung_TD26', 'BC_Lung_TD27', 'BC_Lung_TD28', 'BC_Lung_TD29', 'BC_
Lung_TD3', 'BC_Lung_TD30', 'BC_Lung_TD31', 'BC_Lung_TD32', 'BC_Lung_TD33', 'BC_Lung_TD34', 'BC
_Lung_TD35', 'BC_Lung_TD36', 'BC_Lung_TD37', 'BC_Lung_TD38', 'BC_Lung_TD39', 'BC_Lung_TD4', 'B
C_Lung_TD40', 'BC_Lung_TD5', 'BC_Lung_TD6', 'BC_Lung_TD7', 'BC_Lung_TD8', 'BC_Lung_TD9']
cancer_colon_tissue = ['BC_CRC_TD1', 'BC_CRC_TD10', 'BC_CRC_TD11', 'BC_CRC_TD12', 'BC_CRC_TD13
', 'BC_CRC_TD14', 'BC_CRC_TD15', 'BC_CRC_TD16', 'BC_CRC_TD17', 'BC_CRC_TD18', 'BC_CRC_TD19', '
BC_CRC_TD2', 'BC_CRC_TD20', 'BC_CRC_TD21', 'BC_CRC_TD22', 'BC_CRC_TD23', 'BC_CRC_TD24', 'BC_CR
C_TD25', 'BC_CRC_TD26', 'BC_CRC_TD27', 'BC_CRC_TD28', 'BC_CRC_TD29', 'BC_CRC_TD3', 'BC_CRC_TD3
0', 'BC_CRC_TD31', 'BC_CRC_TD32', 'BC_CRC_TD33', 'BC_CRC_TD34', 'BC_CRC_TD35', 'BC_CRC_TD36',
'BC_CRC_TD37', 'BC_CRC_TD38', 'BC_CRC_TD39', 'BC_CRC_TD4', 'BC_CRC_TD40', 'BC_CRC_TD5', 'BC_CR
C_TD6', 'BC_CRC_TD7', 'BC_CRC_TD8', 'BC_CRC_TD9']
cancer_stomach_tissue = ['BC_Stomach_TD1', 'BC_Stomach_TD10', 'BC_Stomach_TD11', 'BC_Stomach_T
D12', 'BC_Stomach_TD13', 'BC_Stomach_TD14', 'BC_Stomach_TD15', 'BC_Stomach_TD16', 'BC_Stomach_
TD17', 'BC_Stomach_TD18', 'BC_Stomach_TD19', 'BC_Stomach_TD2', 'BC_Stomach_TD20', 'BC_Stomach_
TD21', 'BC_Stomach_TD22', 'BC_Stomach_TD23', 'BC_Stomach_TD24', 'BC_Stomach_TD25', 'BC_Stomach
_TD26', 'BC_Stomach_TD27', 'BC_Stomach_TD28', 'BC_Stomach_TD29', 'BC_Stomach_TD3', 'BC_Stomach
_TD30', 'BC_Stomach_TD31', 'BC_Stomach_TD32', 'BC_Stomach_TD33', 'BC_Stomach_TD34', 'BC_Stomac
h_TD35', 'BC_Stomach_TD36', 'BC_Stomach_TD37', 'BC_Stomach_TD38', 'BC_Stomach_TD39', 'BC_Stoma
ch_TD4', 'BC_Stomach_TD40', 'BC_Stomach_TD5', 'BC_Stomach_TD6', 'BC_Stomach_TD7', 'BC_Stomach_
TD8', 'BC_Stomach_TD9']
cancer_breast_tissue = ['BC_Breast_TD1', 'BC_Breast_TD10', 'BC_Breast_TD11', 'BC_Breast_TD12',
'BC_Breast_TD13', 'BC_Breast_TD14', 'BC_Breast_TD15', 'BC_Breast_TD16', 'BC_Breast_TD17', 'BC_
Breast_TD18', 'BC_Breast_TD19', 'BC_Breast_TD2', 'BC_Breast_TD20', 'BC_Breast_TD21', 'BC_Breas
t_TD22', 'BC_Breast_TD23', 'BC_Breast_TD24', 'BC_Breast_TD25', 'BC_Breast_TD26', 'BC_Breast_TD
27', 'BC_Breast_TD28', 'BC_Breast_TD29', 'BC_Breast_TD3', 'BC_Breast_TD30', 'BC_Breast_TD31',
'BC_Breast_TD32', 'BC_Breast_TD33', 'BC_Breast_TD34', 'BC_Breast_TD35', 'BC_Breast_TD36', 'BC_
Breast_TD37', 'BC_Breast_TD38', 'BC_Breast_TD39', 'BC_Breast_TD4', 'BC_Breast_TD40', 'BC_Breas
t_TD5', 'BC_Breast_TD6', 'BC_Breast_TD7', 'BC_Breast_TD8', 'BC_Breast_TD9']

all_tissue_samples = healthy_lung_tissue+healthy_colon_tissue+healthy_stomach_tissue+healthy_b
reast_tissue+cancer_lung_tissue+cancer_colon_tissue+cancer_stomach_tissue+cancer_breast_tissue
```

Load Data

Let's load a data matrix listing the average methylation across each target region for each sample.

In [9]:
```
amf_df_orig = pd.read_csv('SupplementaryData5.tsv', sep="\t", header=0, index_col=0)
```

Let's also load the coverage data.

In [10]:
```
cov_df_orig = pd.read_csv('SupplementaryData7.tsv', sep="\t", header=0, index_col=0)
```

Marker Selection

Let's first remove markers that are not covered in all plasma samples, so we don't have to deal with any missing values.

In [23]:
```
amf_df = amf_df_orig.copy()
amf_df = amf_df.loc[amf_df[healthy_samples+post_diagnosis_samples+pre_diagnosis_samples].dropn
a(how='any').index]
```

Let's also remove any low-depth regions.

In [24]:
```
amf_df = amf_df[cov_df_orig.loc[amf_df.index, healthy_samples+post_diagnosis_samples+pre_diagn
osis_samples].min(axis=1) > 10]
```

Next, let's choose markers that separate healthy tissue from cancer tissue.

For each tissue type we purchased from BioChain, we will use a T-test to identify target regions that separate normal tissue from cancer tissue (applying a Benjamini-Hochberg multiple testing correction).

In [26]:
```
tissue_t_tests = pd.concat(
    [
        pd.Series(amf_df.index),
        pd.Series(scipy.stats.ttest_ind(amf_df.loc[:, healthy_colon_tissue].T,
                                        amf_df.loc[:, cancer_colon_tissue].T,
                                        axis=0, equal_var=True, nan_policy='omit').pvalue),
        pd.Series(scipy.stats.ttest_ind(amf_df.loc[:, healthy_lung_tissue].T,
                                        amf_df.loc[:, cancer_lung_tissue].T,
                                        axis=0, equal_var=True, nan_policy='omit').pvalue),
        pd.Series(scipy.stats.ttest_ind(amf_df.loc[:, healthy_stomach_tissue].T,
                                        amf_df.loc[:, cancer_stomach_tissue].T,
                                        axis=0, equal_var=True, nan_policy='omit').pvalue),
        pd.Series(scipy.stats.ttest_ind(amf_df.loc[:, healthy_breast_tissue].T,
                                        amf_df.loc[:, cancer_breast_tissue].T,
                                        axis=0, equal_var=True, nan_policy='omit').pvalue),
    ], axis=1
)

tissue_t_tests.columns = ['Locus',
                          'ColonM', 'LungM', 'StomachM', 'BreastM',
                         ]
tissue_t_tests = tissue_t_tests.set_index('Locus')

# Benjamini-Hochberg Correction
from statsmodels.stats.multitest import multipletests
_, pvals_corrected, _, _ = multipletests(tissue_t_tests.min(axis=1).fillna(1).values, alpha=0.
05, method='fdr_bh')

tissue_t_tests_min_by_marker = tissue_t_tests.loc[pvals_corrected <= 0.05].iloc[:, :4].min(axi
s=1).copy()
```

In [27]:
```
print len(tissue_t_tests_min_by_marker)
```

```
317
```

We can then use these target regions in our plasma samples to build a classifier.

Divide Samples Into Leave-In and Leave-Out

Let's first split the Taizhou samples into two sets: leave-in (1/2) and leave-out (1/2). The leave-in set will be used to build a model.

We want to make sure that equal proportions of healthy/cancer samples are included in the leave-in/leave-out set. We also want balance within each type of cancer (so we don't end up with no samples for one cancer type in the leave-out set, for example).

In [28]:
```
random.seed(128)

# Healthy
random.shuffle(healthy_samples)
# Post-Diagnosis
random.shuffle(post_diagnosis_samples_colon)
random.shuffle(post_diagnosis_samples_liver)
random.shuffle(post_diagnosis_samples_esophagus)
random.shuffle(post_diagnosis_samples_lung)
random.shuffle(post_diagnosis_samples_stomach)
# Pre-Diagnosis
random.shuffle(pre_diagnosis_samples_colon)
random.shuffle(pre_diagnosis_samples_liver)
random.shuffle(pre_diagnosis_samples_esophagus)
random.shuffle(pre_diagnosis_samples_lung)
random.shuffle(pre_diagnosis_samples_stomach)

negative_leavein = list(healthy_samples[:int(len(healthy_samples)/2)])
negative_leaveout = list(healthy_samples[int(len(healthy_samples)/2):])

positive_post_leavein = post_diagnosis_samples_colon[:int(len(post_diagnosis_samples_colon)/2)
] + post_diagnosis_samples_liver[:int(len(post_diagnosis_samples_liver)/2)] + post_diagnosis_s
amples_esophagus[:int(len(post_diagnosis_samples_esophagus)/2)] + post_diagnosis_samples_lung[
:int(len(post_diagnosis_samples_lung)/2)] + post_diagnosis_samples_stomach[:int(len(post_diagn
osis_samples_stomach)/2)]
positive_post_leaveout = post_diagnosis_samples_colon[int(len(post_diagnosis_samples_colon)/2)
:] + post_diagnosis_samples_liver[int(len(post_diagnosis_samples_liver)/2):] + post_diagnosis_
samples_esophagus[int(len(post_diagnosis_samples_esophagus)/2):] + post_diagnosis_samples_lung
[int(len(post_diagnosis_samples_lung)/2):] + post_diagnosis_samples_stomach[int(len(post_diagn
osis_samples_stomach)/2):]

positive_pre_leavein = pre_diagnosis_samples_colon[:int(len(pre_diagnosis_samples_colon)/2)] +
pre_diagnosis_samples_liver[:int(len(pre_diagnosis_samples_liver)/2)] + pre_diagnosis_samples_
esophagus[:int(len(pre_diagnosis_samples_esophagus)/2)] + pre_diagnosis_samples_lung[:int(len(
pre_diagnosis_samples_lung)/2)] + pre_diagnosis_samples_stomach[:int(len(pre_diagnosis_samples
_stomach)/2)]
positive_pre_leaveout = pre_diagnosis_samples_colon[int(len(pre_diagnosis_samples_colon)/2):]
+ pre_diagnosis_samples_liver[int(len(pre_diagnosis_samples_liver)/2):] + pre_diagnosis_sample
s_esophagus[int(len(pre_diagnosis_samples_esophagus)/2):] + pre_diagnosis_samples_lung[int(len
(pre_diagnosis_samples_lung)/2):] + pre_diagnosis_samples_stomach[int(len(pre_diagnosis_sample
s_stomach)/2):]
```

Build a Logistic Regression Classifier

Now, let's build an LR classifier from the leave-in set. We will use the method described in Teschendorff et al, Nature Materials (2019). Briefly:

1. We will randomly split the leave-in set into 50% training, 50% test.
2. We will train an LR classifier on the training set, and compute the results on the test set.
3. We will repeat steps 1 and 2 using 1000 different random splits.
4. We will then compute the ensemble performance of these 1000 models across the ten separate test sets, and report this as the "leave-in set" accuracy.
5. We will then apply all 1000 models to the "leave-out set," compute the ensemble performance, and report this as the "leave-out set" accuracy.

Let's build each LR classifier using 1000 different random splits:

In [29]:
```
negative_test_list = []
positive_post_test_list = []
positive_pre_test_list = []
z_prob_list = []

for random_state in range(1000):
    random.seed(random_state)

    # Set up LR classifier that will automatically fit the best C value using the training set
    clf = LogisticRegressionCV(penalty='l1', solver='liblinear', random_state=random_state,
                               Cs=[1.0, 5.0, 10.0, 50.0, 100.0], cv=3)

    # Split the leave-in set into training and test set
    negative = list(negative_leavein)
    positive_post = list(positive_post_leavein)
    positive_pre = list(positive_pre_leavein)
    random.shuffle(negative)
    random.shuffle(positive_post)
    random.shuffle(positive_pre)
    negative_training, negative_test = negative[:1*int(len(negative)/2)], negative[int(1*len(n
egative)/2):]
    positive_post_training, positive_post_test = positive_post[:1*int(len(positive_post)/2)],
positive_post[int(1*len(positive_post)/2):]
    positive_pre_training, positive_pre_test = positive_pre[:1*int(len(positive_pre)/2)], posi
tive_pre[int(1*len(positive_pre)/2):]
    positive_training = positive_post_training+positive_pre_training
    positive_test = positive_post_test+positive_pre_test

    # Build the model and set parameters using the training set
    x = amf_df.loc[tissue_t_tests_min_by_marker.index, negative_training+positive_training].tr
anspose()
    y = ['Healthy']*len(negative_training) + ['Cancer']*len(positive_training)
    clf.fit(x,y)

    # Compute the accuracy of this classifier on the leave-in and leave-out sets
    z = pd.Series(clf.predict(amf_df.loc[tissue_t_tests_min_by_marker.index, healthy_samples+p
ost_diagnosis_samples+pre_diagnosis_samples].transpose()),
                  index=healthy_samples+post_diagnosis_samples+pre_diagnosis_samples)
    z_prob = pd.Series(clf.predict_proba(amf_df.loc[tissue_t_tests_min_by_marker.index, health
y_samples+post_diagnosis_samples+pre_diagnosis_samples].transpose())[:, 0],
                       index=healthy_samples+post_diagnosis_samples+pre_diagnosis_samples)

    # Store the results
    negative_test_list.append(negative_test)
    positive_post_test_list.append(positive_post_test)
    positive_pre_test_list.append(positive_pre_test)
    z_prob_list.append(z_prob)
```

Next, let's compute the ensemble performance across the leave-in test sets and the leave-out sets.

In [30]:
```
ensemble_score_leavein = pd.concat([z_prob_list[index].loc[negative_test_list[index]+positive_
pre_test_list[index]+positive_post_test_list[index]]
                                    for index in range(1000)],
                                   axis=1).mean(axis=1)
ensemble_score_leaveout = pd.concat([z_prob_list[index].loc[negative_leaveout+positive_post_le
aveout+positive_pre_leaveout]
                                     for index in range(1000)],
                                    axis=1).mean(axis=1)
```

We can now compute accuracy metrics for the two sets; as per Teschendorff et al 2019, we will choose the model cutoff using the leave-in set.

Leave-In Set Accuracy

Let's choose a cutoff that aims for ~95% specificity in the leave-in set, and compute the associated sensitivity.

In [56]:
```
sns.set_style('whitegrid')

y_true = ['Healthy']*len(negative_leavein) + ['Cancer']*len(positive_post_leavein)
y_score = ensemble_score_leavein.loc[negative_leavein+positive_post_leavein]
cur_fpr, cur_tpr, cur_cutoff = roc_curve(y_true, y_score, pos_label='Cancer')
cur_auc = auc(cur_fpr, cur_tpr)
plt.plot(cur_fpr, cur_tpr, label=r'Post-Diagnosis ROC (AUC = %0.2f)' % cur_auc)

y_true = ['Healthy']*len(negative_leavein) + ['Cancer']*len(positive_pre_leavein)
y_score = ensemble_score_leavein.loc[negative_leavein+positive_pre_leavein]
cur_fpr, cur_tpr, cur_cutoff = roc_curve(y_true, y_score, pos_label='Cancer')
cur_auc = auc(cur_fpr, cur_tpr)
plt.plot(cur_fpr, cur_tpr, label=r'Pre-Diagnosis ROC (AUC = %0.2f)' % cur_auc)

plt.xlim([-0.0, 1.0])
plt.ylim([-0.0, 1.0])
plt.xticks([0, 0.05, 0.1, 0.2, 0.3, 0.4, 0.5, 0.6, 0.7, 0.8, 0.9, 1.0])
plt.yticks([0, 0.05, 0.1, 0.2, 0.3, 0.4, 0.5, 0.6, 0.7, 0.8, 0.9, 1.0])
plt.xlabel('False Positive Rate')
plt.ylabel('True Positive Rate')
plt.legend(loc="lower right");

print "Leave-In Results"
print "Specificity: %s" % (sum(ensemble_score_leavein.loc[negative_leavein] < 0.576) / len(neg
ative_leavein))
print "Post-Diagnosis Sensitivity: %s" % (sum(ensemble_score_leavein.loc[positive_post_leavein
] >= 0.576) / len(positive_post_leavein))
print "Pre-Diagnosis Sensitivity: %s" % (sum(ensemble_score_leavein.loc[positive_pre_leavein]
>= 0.576) / len(positive_pre_leavein))

plt.figure()
sns.distplot(ensemble_score_leavein.loc[negative_leavein])
sns.distplot(ensemble_score_leavein.loc[positive_post_leavein])
sns.distplot(ensemble_score_leavein.loc[positive_pre_leavein])
plt.legend(['Healthy', 'Post-Diagnosis', 'Pre-Diagnosis']);
plt.xlim(0, 1)
```

```
Leave-In Results
Specificity: 0.932367149758
Post-Diagnosis Sensitivity: 0.881818181818
Pre-Diagnosis Sensitivity: 0.94623655914
```

Out[56]: (0, 1)

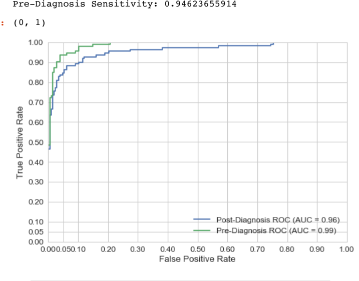

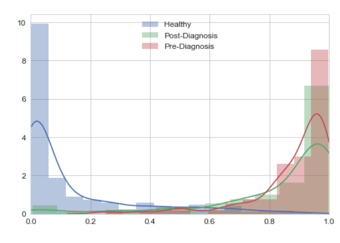

Leave-Out Set Accuracy

Let's compute the leave-out set sensitivity and specificity using the previously chosen cutoff.

In [55]:
```
sns.set_style('whitegrid')

y_true = ['Healthy']*len(negative_leaveout) + ['Cancer']*len(positive_post_leaveout)
y_score = ensemble_score_leaveout.loc[negative_leaveout+positive_post_leaveout]
cur_fpr, cur_tpr, cur_cutoff = roc_curve(y_true, y_score, pos_label='Cancer')
cur_auc = auc(cur_fpr, cur_tpr)
plt.plot(cur_fpr, cur_tpr, label=r'Post-Diagnosis ROC (AUC = %0.2f)' % cur_auc)

y_true = ['Healthy']*len(negative_leaveout) + ['Cancer']*len(positive_pre_leaveout)
y_score = ensemble_score_leaveout.loc[negative_leaveout+positive_pre_leaveout]
cur_fpr, cur_tpr, cur_cutoff = roc_curve(y_true, y_score, pos_label='Cancer')
cur_auc = auc(cur_fpr, cur_tpr)
plt.plot(cur_fpr, cur_tpr, label=r'Pre-Diagnosis ROC (AUC = %0.2f)' % cur_auc)

plt.xlim([-0.0, 1.0])
plt.ylim([-0.0, 1.0])
plt.xticks([0, 0.05, 0.1, 0.2, 0.3, 0.4, 0.5, 0.6, 0.7, 0.8, 0.9, 1.0])
plt.yticks([0, 0.05, 0.1, 0.2, 0.3, 0.4, 0.5, 0.6, 0.7, 0.8, 0.9, 1.0])
plt.xlabel('False Positive Rate')
plt.ylabel('True Positive Rate')
plt.legend(loc="lower right");

print "Leave-Out Results"
print "Specificity: %s" % (sum(ensemble_score_leaveout.loc[negative_leaveout] < 0.576) / len(n
egative_leaveout))
print "Post-Diagnosis Sensitivity: %s" % (sum(ensemble_score_leaveout.loc[positive_post_leaveo
ut] >= 0.576) / len(positive_post_leaveout))
print "Pre-Diagnosis Sensitivity: %s" % (sum(ensemble_score_leaveout.loc[positive_pre_leaveout
] >= 0.576) / len(positive_pre_leaveout))

plt.figure()
sns.distplot(ensemble_score_leaveout.loc[negative_leaveout])
sns.distplot(ensemble_score_leaveout.loc[positive_post_leaveout])
sns.distplot(ensemble_score_leaveout.loc[positive_pre_leaveout])
plt.legend(['Healthy', 'Post-Diagnosis', 'Pre-Diagnosis']);
plt.xlim(0, 1)
```

```
Leave-Out Results
Specificity: 0.946859903382
Post-Diagnosis Sensitivity: 0.83185840708
Pre-Diagnosis Sensitivity: 0.928571428571
```

Out[55]: (0, 1)

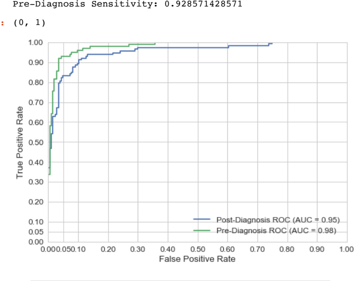

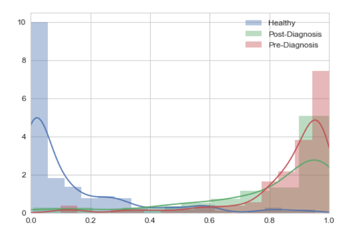

As per Teschendorff et al 2019, the accuracy is similar for the leave-in and leave-out data sets.

Supplementary Note 6.
Code utilized to derive and implement an alternate Logistic Regression classifier that utilizes only genomic regions that show concordant methylation patterns in cancer tissue and cancer plasma. A leave-in set of samples was used to build the classifier, and a leave-out set of samples was utilized to validate the classifier.

Supplementary Note 6

This analysis is similar to that of Supplementary Note 1; however, in this case all regions not showing concordant methylation between tissue and plasma were excluded.

The leave-out set sensitivity and specificity were similar to Supplementary Note 1

In [1]:
```python
from __future__ import division
import sys
import os
import glob
import random
```

In [2]:
```python
import pandas as pd
import numpy as np
import scipy
from sklearn.linear_model import LogisticRegression, LogisticRegressionCV
from sklearn.metrics import roc_curve, auc
from statsmodels.stats.multitest import multipletests
```

In [3]:
```python
%matplotlib inline
import seaborn as sns
import matplotlib.pyplot as plt
```

In [4]:
```python
sns.set()
sns.set_context('notebook', font_scale=1.2)
sns.set_style('white')
```

Sample Info

For this study, we processed two sets of samples; one set of plasma samples from the Taizhou Longitudinal Study, and a second set of commercially purchsed normal and cancer tissue samples from BioChain.

Taizhou Longitudinal Study

In [5]:
```python
pre_diagnosis_samples = ['TSH-PL180123001', 'TSH-PL180123006', 'TSH-PL180123007', 'TSH-PL18012
3008', 'TSH-PL180123010', 'TSH-PL180123013', 'TSH-PL180123014', 'TSH-PL180123016', 'TSH-PL1801
23017', 'TSH-PL180123018', 'TSH-PL180123021', 'TSH-PL180123022', 'TSH-PL180123025', 'TSH-PL180
123027', 'TSH-PL180123029', 'TSH-PL180123030', 'TSH-PL180123031', 'TSH-PL180123033', 'TSH-PL18
0513004', 'TSH-PL180513005', 'TSH-PL180513006', 'TSH-PL180513007', 'TSH-PL180513011', 'TSH-PL1
80513012', 'TSH-PL180513013', 'TSH-PL180513014', 'TSH-PL180513016', 'TSH-PL180513017', 'TSH-PL
180513020', 'TSH-PL180513021', 'TSH-PL180513023', 'TSH-PL180513024', 'TSH-PL180513025', 'TSH-P
L180513026', 'TSH-PL180513027', 'TSH-PL180513029', 'TSH-PL180513030', 'TSH-PL180513034', 'TSH-
PL180513036', 'TSH-PL180513037', 'TSH-PL180513038', 'TSH-PL180513039', 'TSH-PL180513040', 'TSH
-PL180513041', 'TSH-PL180513043', 'TSH-PL180513045', 'TSH-PL180513049', 'TSH-PL180513050', 'TS
H-PL180513051', 'TSH-PL180513055', 'TSH-PL180513056', 'TSH-PL180513057', 'TSH-PL180513060', 'T
SH-PL180513062', 'TSH-PL180513066', 'TSH-PL180513067', 'TSH-PL180513068', 'TSH-PL180513069', '
TSH-PL180513070', 'TSH-PL180513071', 'TSH-PL180513072', 'TSH-PL180513073', 'TSH-PL180513075',
'TSH-PL180513076', 'TSH-PL180513077', 'TSH-PL180513078', 'TSH-PL180513079', 'TSH-PL180513080',
'TSH-PL180513081', 'TSH-PL180513084', 'TSH-PL180513085', 'TSH-PL180513087', 'TSH-PL180513088',
'TSH-PL180513089', 'TSH-PL180513091', 'TSH-PL180513092', 'TSH-PL180513093', 'TSH-PL180513095',
'TSH-PL180513097', 'TSH-PL180513098', 'TSH-PL180513099', 'TSH-PL180513100', 'TSH-PL180513101',
'TSH-PL180513102', 'TSH-PL180513104', 'TSH-PL180513106', 'TSH-PL180513108', 'TSH-PL180513109',
'TSH-PL180513110', 'TSH-PL180513111', 'TSH-PL180513114', 'TSH-PL180513115', 'TSH-PL180513117',
'TSH-PL180513118', 'TSH-PL180513119', 'TSH-PL180513121', 'TSH-PL180513122', 'TSH-PL180513123',
'TSH-PL180513124', 'TSH-PL180513127', 'TSH-PL180513128', 'TSH-PL180513129', 'TSH-PL180516001',
'TSH-PL180516002', 'TSH-PL180516003', 'TSH-PL180516004', 'TSH-PL180516006', 'TSH-PL180516007',
'TSH-PL180516008', 'TSH-PL180516009', 'TSH-PL180516011', 'TSH-PL180516012', 'TSH-PL180516013',
'TSH-PL180516014', 'TSH-PL180516016', 'TSH-PL180516018', 'TSH-PL180516019', 'TSH-PL180516020',
'TSH-PL180516021', 'TSH-PL180516023', 'TSH-PL180516027', 'TSH-PL180516028', 'TSH-PL180516030',
'TSH-PL180516031', 'TSH-PL180516032', 'TSH-PL180516036', 'TSH-PL180516038', 'TSH-PL180516039',
'TSH-PL180516040', 'TSH-PL180516042', 'TSH-PL180516043', 'TSH-PL180516044', 'TSH-PL180516045',
'TSH-PL180516049', 'TSH-PL180516051', 'TSH-PL180516053', 'TSH-PL180516054', 'TSH-PL180516055',
'TSH-PL180516056', 'TSH-PL180516057', 'TSH-PL180516059', 'TSH-PL180516062', 'TSH-PL180516063',
'TSH-PL180123002', 'TSH-PL180123004', 'TSH-PL180123005', 'TSH-PL180123012', 'TSH-PL180123024',
'TSH-PL180123032', 'TSH-PL180513001', 'TSH-PL180513002', 'TSH-PL180513003', 'TSH-PL180513009',
'TSH-PL180513010', 'TSH-PL180513018', 'TSH-PL180513019', 'TSH-PL180513022', 'TSH-PL180513028',
'TSH-PL180513031', 'TSH-PL180513032', 'TSH-PL180513033', 'TSH-PL180513035', 'TSH-PL180513044',
'TSH-PL180513058', 'TSH-PL180513064', 'TSH-PL180513083', 'TSH-PL180513086', 'TSH-PL180513090',
'TSH-PL180513096', 'TSH-PL180513103', 'TSH-PL180513105', 'TSH-PL180513112', 'TSH-PL180513116',
'TSH-PL180513125', 'TSH-PL180516005', 'TSH-PL180516010', 'TSH-PL180516015', 'TSH-PL180516017',
'TSH-PL180516025', 'TSH-PL180516026', 'TSH-PL180516029', 'TSH-PL180516033', 'TSH-PL180516034',
'TSH-PL180516046', 'TSH-PL180516047', 'TSH-PL180516048', 'TSH-PL180516050', 'TSH-PL180516052',
'TSH-PL180516058', 'TSH-PL180516060', 'TSH-PL180516061']
post_diagnosis_samples = ['TSH-PL171221101', 'TSH-PL171221112', 'TSH-PL171221149', 'TSH-PL1712
21150', 'TSH-PL171221160', 'TSH-PL171221172', 'TSH-PL171221179', 'TSH-PL171221180', 'TSH-PL180
504001', 'TSH-PL180504002', 'TSH-PL180504003', 'TSH-PL180504004', 'TSH-PL180504005', 'TSH-PL18
0504009', 'TSH-PL180504010', 'TSH-PL180504011', 'TSH-PL180504013', 'TSH-PL180504014', 'TSH-PL1
80504015', 'TSH-PL180504016', 'TSH-PL180504017', 'TSH-PL180504018', 'TSH-PL180504019', 'TSH-PL
180504021', 'TSH-PL180504022', 'TSH-PL180504027', 'TSH-PL180504028', 'TSH-PL180504030', 'TSH-P
L180504033', 'TSH-PL180504034', 'TSH-PL180504041', 'TSH-PL180504042', 'TSH-PL180504044', 'TSH-
PL180504046', 'TSH-PL180504047', 'TSH-PL180504049', 'TSH-PL180504050', 'TSH-PL180504053', 'TSH
-PL180504054', 'TSH-PL180504058', 'TSH-PL180504061', 'TSH-PL180504062', 'TSH-PL180504063', 'TS
H-PL180504065', 'TSH-PL180504068', 'TSH-PL180504070', 'TSH-PL180504074', 'TSH-PL180504081', 'T
SH-PL180504083', 'TSH-PL180504087', 'TSH-PL180504088', 'TSH-PL180504089', 'TSH-PL180504091', '
TSH-PL180504094', 'TSH-PL180504097', 'TSH-PL180504101', 'TSH-PL180504104', 'TSH-PL180504108',
'TSH-PL180504111', 'TSH-PL180504118', 'TSH-PL180508001', 'TSH-PL180508004', 'TSH-PL180508005',
'TSH-PL180508006', 'TSH-PL180508008', 'TSH-PL180508009', 'TSH-PL180508011', 'TSH-PL180508012',
'TSH-PL180508013', 'TSH-PL180508014', 'TSH-PL180508015', 'TSH-PL180508019', 'TSH-PL180508020',
'TSH-PL180508021', 'TSH-PL180508022', 'TSH-PL180508025', 'TSH-PL180508030', 'TSH-PL180508031',
'TSH-PL180508033', 'TSH-PL180508034', 'TSH-PL180508036', 'TSH-PL180508037', 'TSH-PL180508038',
'TSH-PL180508039', 'TSH-PL180508040', 'TSH-PL180508041', 'TSH-PL180508042', 'TSH-PL180508043',
'TSH-PL180508049', 'TSH-PL180508050', 'TSH-PL180508060', 'TSH-PL180508061', 'TSH-PL180508064',
'TSH-PL180508068', 'TSH-PL180508076', 'TSH-PL180508078', 'TSH-PL180508083', 'TSH-PL180508086',
'TSH-PL180508089', 'TSH-PL180510005', 'TSH-PL180510006', 'TSH-PL180510007', 'TSH-PL180510008',
'TSH-PL180510010', 'TSH-PL180510011', 'TSH-PL180510012', 'TSH-PL180510014', 'TSH-PL180510015',
'TSH-PL180510016', 'TSH-PL180510017', 'TSH-PL180510018', 'TSH-PL180510019', 'TSH-PL180510021',
'TSH-PL180510023', 'TSH-PL180510024', 'TSH-PL180510025', 'TSH-PL180510026', 'TSH-PL180510030',
'TSH-PL180510031', 'TSH-PL180510033', 'TSH-PL180510038', 'TSH-PL180510040', 'TSH-PL180510044',
'TSH-PL180510047', 'TSH-PL180510050', 'TSH-PL180510052', 'TSH-PL180510053', 'TSH-PL180510054',
'TSH-PL180510055', 'TSH-PL180510057', 'TSH-PL180510061', 'TSH-PL180510063', 'TSH-PL180510066',
'TSH-PL180510071', 'TSH-PL180510072', 'TSH-PL180510073', 'TSH-PL180510074', 'TSH-PL180510075',
'TSH-PL180510076', 'TSH-PL180510077', 'TSH-PL180510078', 'TSH-PL180510079', 'TSH-PL180510080',
'TSH-PL180510085', 'TSH-PL180510089', 'TSH-PL180510092', 'TSH-PL180510095', 'TSH-PL180510101',
'TSH-PL180510104', 'TSH-PL180510105', 'TSH-PL180510107', 'TSH-PL180510112', 'TSH-PL180510121',
'TSH-PL180510122', 'TSH-PL180510125', 'TSH-PL180510139', 'TSH-PL180510140', 'TSH-PL180510146',
'TSH-PL180510147', 'TSH-PL180510162', 'TSH-PL181205168', 'TSH-PL181205170', 'TSH-PL181205173',
'TSH-PL181205175', 'TSH-PL181205182', 'TSH-PL171221151', 'TSH-PL180504006', 'TSH-PL180504012',
'TSH-PL180504024', 'TSH-PL180504026', 'TSH-PL180504029', 'TSH-PL180504037', 'TSH-PL180504043',
'TSH-PL180504045', 'TSH-PL180504051', 'TSH-PL180504052', 'TSH-PL180504055', 'TSH-PL180504057',
'TSH-PL180504064', 'TSH-PL180504076', 'TSH-PL180504084', 'TSH-PL180504110', 'TSH-PL180508003',
'TSH-PL180508007', 'TSH-PL180508023', 'TSH-PL180508026', 'TSH-PL180508028', 'TSH-PL180508029',
'TSH-PL180508032', 'TSH-PL180508044', 'TSH-PL180508052', 'TSH-PL180508065', 'TSH-PL180508067',
'TSH-PL180508088', 'TSH-PL180510002', 'TSH-PL180510004', 'TSH-PL180510013', 'TSH-PL180510020',
'TSH-PL180510022', 'TSH-PL180510032', 'TSH-PL180510034', 'TSH-PL180510035', 'TSH-PL180510043',
'TSH-PL180510048', 'TSH-PL180510068', 'TSH-PL180510088', 'TSH-PL180510090', 'TSH-PL180510098',
'TSH-PL180510100', 'TSH-PL180510103', 'TSH-PL180510108', 'TSH-PL180510109', 'TSH-PL180510113',
'TSH-PL180510117', 'TSH-PL180510124', 'TSH-PL180510143', 'TSH-PL181205169', 'TSH-PL181205172',
'TSH-PL181205174', 'TSH-PL181205177', 'TSH-PL181205179', 'TSH-PL171221153', 'TSH-PL171221113']
healthy_samples = ['TSH-PL180309087', 'TSH-PL180316003', 'TSH-PL180316005', 'TSH-PL180316016',
'TSH-PL180316017', 'TSH-PL180316018', 'TSH-PL180316022', 'TSH-PL180316024', 'TSH-PL180316027',
'TSH-PL180316033', 'TSH-PL180316037', 'TSH-PL180316038', 'TSH-PL180316039', 'TSH-PL180316049',
'TSH-PL180316061', 'TSH-PL180316065', 'TSH-PL180316079', 'TSH-PL180316092', 'TSH-PL180316095',
'TSH-PL180316099', 'TSH-PL180316101', 'TSH-PL180316106', 'TSH-PL180316115', 'TSH-PL180316119',
'TSH-PL180316126', 'TSH-PL180316129', 'TSH-PL180316157', 'TSH-PL180316161', 'TSH-PL180316163',
'TSH-PL180316164', 'TSH-PL180316173', 'TSH-PL180316174', 'TSH-PL180316181', 'TSH-PL180316183',
'TSH-PL180316187', 'TSH-PL180316190', 'TSH-PL180316195', 'TSH-PL180316196', 'TSH-PL180316198',
'TSH-PL180323034', 'TSH-PL180323038', 'TSH-PL180323051', 'TSH-PL180323052', 'TSH-PL180323054',
'TSH-PL180323059', 'TSH-PL180323065', 'TSH-PL180323067', 'TSH-PL180323072', 'TSH-PL180323073',
'TSH-PL180323091', 'TSH-PL180323106', 'TSH-PL180323107', 'TSH-PL180323109', 'TSH-PL180323110',
'TSH-PL180323111', 'TSH-PL180323119', 'TSH-PL180323121', 'TSH-PL180323124', 'TSH-PL180323139',
'TSH-PL180323141', 'TSH-PL180323142', 'TSH-PL180323151', 'TSH-PL180323155', 'TSH-PL180323166',
'TSH-PL180410003', 'TSH-PL180410023', 'TSH-PL180410043', 'TSH-PL180410049', 'TSH-PL180410059',
'TSH-PL180410063', 'TSH-PL180410076', 'TSH-PL180410087', 'TSH-PL180410115', 'TSH-PL180410116',
'TSH-PL180410133', 'TSH-PL180410140', 'TSH-PL180410144', 'TSH-PL180410160', 'TSH-PL180410162',
'TSH-PL180410198', 'TSH-PL180417003', 'TSH-PL180417007', 'TSH-PL180417035', 'TSH-PL180417060',
'TSH-PL180417074', 'TSH-PL180417087', 'TSH-PL180417095', 'TSH-PL180417101', 'TSH-PL180417109',
'TSH-PL180417117', 'TSH-PL180417135', 'TSH-PL180417138', 'TSH-PL180417143', 'TSH-PL180417152',
'TSH-PL180417156', 'TSH-PL180417165', 'TSH-PL180417170', 'TSH-PL180417179', 'TSH-PL180417180',
'TSH-PL180417181', 'TSH-PL180417200', 'TSH-PL180424003', 'TSH-PL180424030', 'TSH-PL180424036',
'TSH-PL180424058', 'TSH-PL180424090', 'TSH-PL180424097', 'TSH-PL180424104', 'TSH-PL180424115',
'TSH-PL180424146', 'TSH-PL180424149', 'TSH-PL180424155', 'TSH-PL180424162', 'TSH-PL180424166',
'TSH-PL180424171', 'TSH-PL180703037', 'TSH-PL180703136', 'TSH-PL180703153', 'TSH-PL180703157',
'TSH-PL180703185', 'TSH-PL180706034', 'TSH-PL180706037', 'TSH-PL180706095', 'TSH-PL180706096',
'TSH-PL180706104', 'TSH-PL180706113', 'TSH-PL180706118', 'TSH-PL180706145', 'TSH-PL180706147',
'TSH-PL180706150', 'TSH-PL180706153', 'TSH-PL180706156', 'TSH-PL181128001', 'TSH-PL181128003',
'TSH-PL181128005', 'TSH-PL181128007', 'TSH-PL181128008', 'TSH-PL181128009', 'TSH-PL181128011',
'TSH-PL181128012', 'TSH-PL181128014', 'TSH-PL181128015', 'TSH-PL181128019', 'TSH-PL181128021',
'TSH-PL181128022', 'TSH-PL181128023', 'TSH-PL181128025', 'TSH-PL181128027', 'TSH-PL181128029',
'TSH-PL181128030', 'TSH-PL181128033', 'TSH-PL181128035', 'TSH-PL181128036', 'TSH-PL181128038',
'TSH-PL181128039', 'TSH-PL181128040', 'TSH-PL181128041', 'TSH-PL181128042', 'TSH-PL181128043',
'TSH-PL181128045', 'TSH-PL181128047', 'TSH-PL181128048', 'TSH-PL181128049', 'TSH-PL181128050',
'TSH-PL181128051', 'TSH-PL181128054', 'TSH-PL181128055', 'TSH-PL181128056', 'TSH-PL181128057',
'TSH-PL181128058', 'TSH-PL181128061', 'TSH-PL181128062', 'TSH-PL181128064', 'TSH-PL181128065',
'TSH-PL181128066', 'TSH-PL181128070', 'TSH-PL181128073', 'TSH-PL181128077', 'TSH-PL181128080',
'TSH-PL181128082', 'TSH-PL181128083', 'TSH-PL181128085', 'TSH-PL181128086', 'TSH-PL181128093',
'TSH-PL181128096', 'TSH-PL181128099', 'TSH-PL181128101', 'TSH-PL181128102', 'TSH-PL181128103',
'TSH-PL181128104', 'TSH-PL181128107', 'TSH-PL181128109', 'TSH-PL181128110', 'TSH-PL181128111',
'TSH-PL181128112', 'TSH-PL181128114', 'TSH-PL181128115', 'TSH-PL181128117', 'TSH-PL181128118',
'TSH-PL181128120', 'TSH-PL181128122', 'TSH-PL181128123', 'TSH-PL181128124', 'TSH-PL181128125',
'TSH-PL181128126', 'TSH-PL181128127', 'TSH-PL181205002', 'TSH-PL181205003', 'TSH-PL181205005',
'TSH-PL181205006', 'TSH-PL181205008', 'TSH-PL181205009', 'TSH-PL181205013', 'TSH-PL181205014',
'TSH-PL181205016', 'TSH-PL181205020', 'TSH-PL181205022', 'TSH-PL181205024', 'TSH-PL181205026',
'TSH-PL181205027', 'TSH-PL181205028', 'TSH-PL181205030', 'TSH-PL181205033', 'TSH-PL181205035',
'TSH-PL181205040', 'TSH-PL181205041', 'TSH-PL181205042', 'TSH-PL181205043', 'TSH-PL181205044',
'TSH-PL181205045', 'TSH-PL181205046', 'TSH-PL181205047', 'TSH-PL181205048', 'TSH-PL181205049',
'TSH-PL181205051', 'TSH-PL181205053', 'TSH-PL181205054', 'TSH-PL181205055', 'TSH-PL181205057',
'TSH-PL181205058', 'TSH-PL181205059', 'TSH-PL181205060', 'TSH-PL181205061', 'TSH-PL181205062',
'TSH-PL181205063', 'TSH-PL181205064', 'TSH-PL181205065', 'TSH-PL181205066', 'TSH-PL181205068',
'TSH-PL181205069', 'TSH-PL181205072', 'TSH-PL181205074', 'TSH-PL181205075', 'TSH-PL181205076',
'TSH-PL181205077', 'TSH-PL181205078', 'TSH-PL181205079', 'TSH-PL181205081', 'TSH-PL181205082',
'TSH-PL181205083', 'TSH-PL181205085', 'TSH-PL181205086', 'TSH-PL181205087', 'TSH-PL181205088',
'TSH-PL181205090', 'TSH-PL181205091', 'TSH-PL181205093', 'TSH-PL181205095', 'TSH-PL181205096',
'TSH-PL181205097', 'TSH-PL181205098', 'TSH-PL181205099', 'TSH-PL181205102', 'TSH-PL181205103',
'TSH-PL181205104', 'TSH-PL181205107', 'TSH-PL181205108', 'TSH-PL181205110', 'TSH-PL181205113',
'TSH-PL181205115', 'TSH-PL181205116', 'TSH-PL181205117', 'TSH-PL181205121', 'TSH-PL181205122',
'TSH-PL181205125', 'TSH-PL181205128', 'TSH-PL181205129', 'TSH-PL181205140', 'TSH-PL181205141',
'TSH-PL181205142', 'TSH-PL181205143', 'TSH-PL181205144', 'TSH-PL181205145', 'TSH-PL181205146',
'TSH-PL181205147', 'TSH-PL181205148', 'TSH-PL181205149', 'TSH-PL181205150', 'TSH-PL181205151',
'TSH-PL181205152', 'TSH-PL181205155', 'TSH-PL181205156', 'TSH-PL181205157', 'TSH-PL181205158',
'TSH-PL181205160', 'TSH-PL181205161', 'TSH-PL181205162', 'TSH-PL181205164', 'TSH-PL181205165',
'TSH-PL181205166', 'TSH-PL180309082', 'TSH-PL180316013', 'TSH-PL180316029', 'TSH-PL180316075',
'TSH-PL180316102', 'TSH-PL180316112', 'TSH-PL180316134', 'TSH-PL180316138', 'TSH-PL180316175',
'TSH-PL180316178', 'TSH-PL180316200', 'TSH-PL180323029', 'TSH-PL180323032', 'TSH-PL180323053',
'TSH-PL180323068', 'TSH-PL180323074', 'TSH-PL180323088', 'TSH-PL180323097', 'TSH-PL180323103',
'TSH-PL180323115', 'TSH-PL180323120', 'TSH-PL180323160', 'TSH-PL180323175', 'TSH-PL180410058',
'TSH-PL180410070', 'TSH-PL180410081', 'TSH-PL180410148', 'TSH-PL180410161', 'TSH-PL180417017',
'TSH-PL180417023', 'TSH-PL180417045', 'TSH-PL180417077', 'TSH-PL180417104', 'TSH-PL180417111',
'TSH-PL180417124', 'TSH-PL180417162', 'TSH-PL180417169', 'TSH-PL180417199', 'TSH-PL180424042',
'TSH-PL180424088', 'TSH-PL180424138', 'TSH-PL180703012', 'TSH-PL180703127', 'TSH-PL180703134',
'TSH-PL180703141', 'TSH-PL180703168', 'TSH-PL180706117', 'TSH-PL181128004', 'TSH-PL181128006',
'TSH-PL181128013', 'TSH-PL181128018', 'TSH-PL181128020', 'TSH-PL181128031', 'TSH-PL181128037',
'TSH-PL181128067', 'TSH-PL181128071', 'TSH-PL181128078', 'TSH-PL181128084', 'TSH-PL181128095',
'TSH-PL181128097', 'TSH-PL181128098', 'TSH-PL181128105', 'TSH-PL181128106', 'TSH-PL181128108',
'TSH-PL181128113', 'TSH-PL181128116', 'TSH-PL181205001', 'TSH-PL181205004', 'TSH-PL181205007',
'TSH-PL181205010', 'TSH-PL181205011', 'TSH-PL181205012', 'TSH-PL181205015', 'TSH-PL181205018',
'TSH-PL181205019', 'TSH-PL181205023', 'TSH-PL181205031', 'TSH-PL181205032', 'TSH-PL181205036',
'TSH-PL181205037', 'TSH-PL181205052', 'TSH-PL181205056', 'TSH-PL181205067', 'TSH-PL181205070',
'TSH-PL181205071', 'TSH-PL181205073', 'TSH-PL181205080', 'TSH-PL181205084', 'TSH-PL181205089',
'TSH-PL181205092', 'TSH-PL181205094', 'TSH-PL181205100', 'TSH-PL181205101', 'TSH-PL181205106',
'TSH-PL181205112', 'TSH-PL181205118', 'TSH-PL181205119', 'TSH-PL181205120', 'TSH-PL181205123',
'TSH-PL181205124', 'TSH-PL181205153', 'TSH-PL181205154', 'TSH-PL181205159', 'TSH-PL181205163']
```

In [6]:
```python
healthy_samples = ['TSH-PL180309087', 'TSH-PL180316003', 'TSH-PL180316005', 'TSH-PL180316016',
'TSH-PL180316017', 'TSH-PL180316018', 'TSH-PL180316022', 'TSH-PL180316024', 'TSH-PL180316027',
'TSH-PL180316033', 'TSH-PL180316037', 'TSH-PL180316038', 'TSH-PL180316039', 'TSH-PL180316049',
'TSH-PL180316061', 'TSH-PL180316065', 'TSH-PL180316079', 'TSH-PL180316092', 'TSH-PL180316095',
'TSH-PL180316099', 'TSH-PL180316101', 'TSH-PL180316106', 'TSH-PL180316115', 'TSH-PL180316119',
'TSH-PL180316126', 'TSH-PL180316129', 'TSH-PL180316157', 'TSH-PL180316161', 'TSH-PL180316163',
'TSH-PL180316164', 'TSH-PL180316173', 'TSH-PL180316174', 'TSH-PL180316181', 'TSH-PL180316183',
'TSH-PL180316187', 'TSH-PL180316190', 'TSH-PL180316195', 'TSH-PL180316196', 'TSH-PL180316198',
'TSH-PL180323034', 'TSH-PL180323038', 'TSH-PL180323051', 'TSH-PL180323052', 'TSH-PL180323054',
'TSH-PL180323059', 'TSH-PL180323065', 'TSH-PL180323067', 'TSH-PL180323072', 'TSH-PL180323073',
'TSH-PL180323091', 'TSH-PL180323106', 'TSH-PL180323107', 'TSH-PL180323109', 'TSH-PL180323110',
'TSH-PL180323111', 'TSH-PL180323119', 'TSH-PL180323121', 'TSH-PL180323124', 'TSH-PL180323139',
'TSH-PL180323141', 'TSH-PL180323142', 'TSH-PL180323151', 'TSH-PL180323155', 'TSH-PL180323166',
'TSH-PL180410003', 'TSH-PL180410023', 'TSH-PL180410043', 'TSH-PL180410049', 'TSH-PL180410059',
'TSH-PL180410063', 'TSH-PL180410076', 'TSH-PL180410087', 'TSH-PL180410115', 'TSH-PL180410116',
'TSH-PL180410133', 'TSH-PL180410140', 'TSH-PL180410144', 'TSH-PL180410160', 'TSH-PL180410162',
'TSH-PL180410198', 'TSH-PL180417003', 'TSH-PL180417007', 'TSH-PL180417035', 'TSH-PL180417060',
'TSH-PL180417074', 'TSH-PL180417087', 'TSH-PL180417095', 'TSH-PL180417101', 'TSH-PL180417109',
'TSH-PL180417117', 'TSH-PL180417135', 'TSH-PL180417138', 'TSH-PL180417143', 'TSH-PL180417152',
'TSH-PL180417156', 'TSH-PL180417165', 'TSH-PL180417170', 'TSH-PL180417179', 'TSH-PL180417180',
'TSH-PL180417181', 'TSH-PL180417200', 'TSH-PL180424003', 'TSH-PL180424030', 'TSH-PL180424036',
'TSH-PL180424058', 'TSH-PL180424090', 'TSH-PL180424097', 'TSH-PL180424104', 'TSH-PL180424115',
'TSH-PL180424146', 'TSH-PL180424149', 'TSH-PL180424155', 'TSH-PL180424162', 'TSH-PL180424166',
'TSH-PL180424171', 'TSH-PL180703037', 'TSH-PL180703136', 'TSH-PL180703153', 'TSH-PL180703157',
'TSH-PL180703185', 'TSH-PL180706034', 'TSH-PL180706037', 'TSH-PL180706095', 'TSH-PL180706096',
'TSH-PL180706104', 'TSH-PL180706113', 'TSH-PL180706118', 'TSH-PL180706145', 'TSH-PL180706147',
'TSH-PL180706150', 'TSH-PL180706153', 'TSH-PL180706156', 'TSH-PL181128001', 'TSH-PL181128003',
'TSH-PL181128005', 'TSH-PL181128007', 'TSH-PL181128008', 'TSH-PL181128009', 'TSH-PL181128011',
'TSH-PL181128012', 'TSH-PL181128014', 'TSH-PL181128015', 'TSH-PL181128019', 'TSH-PL181128021',
'TSH-PL181128022', 'TSH-PL181128023', 'TSH-PL181128025', 'TSH-PL181128027', 'TSH-PL181128029',
'TSH-PL181128030', 'TSH-PL181128033', 'TSH-PL181128035', 'TSH-PL181128036', 'TSH-PL181128038',
'TSH-PL181128039', 'TSH-PL181128040', 'TSH-PL181128041', 'TSH-PL181128042', 'TSH-PL181128043',
'TSH-PL181128045', 'TSH-PL181128047', 'TSH-PL181128048', 'TSH-PL181128049', 'TSH-PL181128050',
'TSH-PL181128051', 'TSH-PL181128054', 'TSH-PL181128055', 'TSH-PL181128056', 'TSH-PL181128057',
'TSH-PL181128058', 'TSH-PL181128061', 'TSH-PL181128062', 'TSH-PL181128064', 'TSH-PL181128065',
'TSH-PL181128066', 'TSH-PL181128070', 'TSH-PL181128073', 'TSH-PL181128077', 'TSH-PL181128080',
'TSH-PL181128082', 'TSH-PL181128083', 'TSH-PL181128085', 'TSH-PL181128086', 'TSH-PL181128093',
'TSH-PL181128096', 'TSH-PL181128099', 'TSH-PL181128101', 'TSH-PL181128102', 'TSH-PL181128103',
'TSH-PL181128104', 'TSH-PL181128107', 'TSH-PL181128109', 'TSH-PL181128110', 'TSH-PL181128111',
'TSH-PL181128112', 'TSH-PL181128114', 'TSH-PL181128115', 'TSH-PL181128117', 'TSH-PL181128118',
'TSH-PL181128120', 'TSH-PL181128122', 'TSH-PL181128123', 'TSH-PL181128124', 'TSH-PL181128125',
'TSH-PL181128126', 'TSH-PL181128127', 'TSH-PL181205002', 'TSH-PL181205003', 'TSH-PL181205005',
'TSH-PL181205006', 'TSH-PL181205008', 'TSH-PL181205009', 'TSH-PL181205013', 'TSH-PL181205014',
'TSH-PL181205016', 'TSH-PL181205020', 'TSH-PL181205022', 'TSH-PL181205024', 'TSH-PL181205026',
'TSH-PL181205027', 'TSH-PL181205028', 'TSH-PL181205030', 'TSH-PL181205033', 'TSH-PL181205035',
'TSH-PL181205040', 'TSH-PL181205041', 'TSH-PL181205042', 'TSH-PL181205043', 'TSH-PL181205044',
'TSH-PL181205045', 'TSH-PL181205046', 'TSH-PL181205047', 'TSH-PL181205048', 'TSH-PL181205049',
'TSH-PL181205051', 'TSH-PL181205053', 'TSH-PL181205054', 'TSH-PL181205055', 'TSH-PL181205057',
'TSH-PL181205058', 'TSH-PL181205059', 'TSH-PL181205060', 'TSH-PL181205061', 'TSH-PL181205062',
'TSH-PL181205063', 'TSH-PL181205064', 'TSH-PL181205065', 'TSH-PL181205066', 'TSH-PL181205068',
'TSH-PL181205069', 'TSH-PL181205072', 'TSH-PL181205074', 'TSH-PL181205075', 'TSH-PL181205076',
'TSH-PL181205077', 'TSH-PL181205078', 'TSH-PL181205079', 'TSH-PL181205081', 'TSH-PL181205082',
'TSH-PL181205083', 'TSH-PL181205085', 'TSH-PL181205086', 'TSH-PL181205087', 'TSH-PL181205088',
'TSH-PL181205090', 'TSH-PL181205091', 'TSH-PL181205093', 'TSH-PL181205095', 'TSH-PL181205096',
'TSH-PL181205097', 'TSH-PL181205098', 'TSH-PL181205099', 'TSH-PL181205102', 'TSH-PL181205103',
'TSH-PL181205104', 'TSH-PL181205107', 'TSH-PL181205108', 'TSH-PL181205110', 'TSH-PL181205113',
'TSH-PL181205115', 'TSH-PL181205116', 'TSH-PL181205117', 'TSH-PL181205121', 'TSH-PL181205122',
'TSH-PL181205125', 'TSH-PL181205128', 'TSH-PL181205129', 'TSH-PL181205140', 'TSH-PL181205141',
'TSH-PL181205142', 'TSH-PL181205143', 'TSH-PL181205144', 'TSH-PL181205145', 'TSH-PL181205146',
'TSH-PL181205147', 'TSH-PL181205148', 'TSH-PL181205149', 'TSH-PL181205150', 'TSH-PL181205151',
'TSH-PL181205152', 'TSH-PL181205155', 'TSH-PL181205156', 'TSH-PL181205157', 'TSH-PL181205158',
'TSH-PL181205160', 'TSH-PL181205161', 'TSH-PL181205162', 'TSH-PL181205164', 'TSH-PL181205165',
'TSH-PL181205166', 'TSH-PL180309082', 'TSH-PL180316013', 'TSH-PL180316029', 'TSH-PL180316075',
'TSH-PL180316102', 'TSH-PL180316112', 'TSH-PL180316134', 'TSH-PL180316138', 'TSH-PL180316175',
'TSH-PL180316178', 'TSH-PL180316200', 'TSH-PL180323029', 'TSH-PL180323032', 'TSH-PL180323053',
'TSH-PL180323068', 'TSH-PL180323074', 'TSH-PL180323088', 'TSH-PL180323097', 'TSH-PL180323103',
'TSH-PL180323115', 'TSH-PL180323120', 'TSH-PL180323160', 'TSH-PL180323175', 'TSH-PL180410058',
'TSH-PL180410070', 'TSH-PL180410081', 'TSH-PL180410148', 'TSH-PL180410161', 'TSH-PL180417017',
'TSH-PL180417023', 'TSH-PL180417045', 'TSH-PL180417077', 'TSH-PL180417104', 'TSH-PL180417111',
'TSH-PL180417124', 'TSH-PL180417162', 'TSH-PL180417169', 'TSH-PL180417199', 'TSH-PL180424042',
'TSH-PL180424088', 'TSH-PL180424138', 'TSH-PL180703012', 'TSH-PL180703127', 'TSH-PL180703134',
'TSH-PL180703141', 'TSH-PL180703168', 'TSH-PL180706117', 'TSH-PL181128004', 'TSH-PL181128006',
'TSH-PL181128013', 'TSH-PL181128018', 'TSH-PL181128020', 'TSH-PL181128031', 'TSH-PL181128037',
'TSH-PL181128067', 'TSH-PL181128071', 'TSH-PL181128078', 'TSH-PL181128084', 'TSH-PL181128095',
'TSH-PL181128097', 'TSH-PL181128098', 'TSH-PL181128105', 'TSH-PL181128106', 'TSH-PL181128108',
'TSH-PL181128113', 'TSH-PL181128116', 'TSH-PL181205001', 'TSH-PL181205004', 'TSH-PL181205007',
'TSH-PL181205010', 'TSH-PL181205011', 'TSH-PL181205012', 'TSH-PL181205015', 'TSH-PL181205018',
'TSH-PL181205019', 'TSH-PL181205023', 'TSH-PL181205031', 'TSH-PL181205032', 'TSH-PL181205036',
'TSH-PL181205037', 'TSH-PL181205052', 'TSH-PL181205056', 'TSH-PL181205067', 'TSH-PL181205070',
'TSH-PL181205071', 'TSH-PL181205073', 'TSH-PL181205080', 'TSH-PL181205084', 'TSH-PL181205089',
'TSH-PL181205092', 'TSH-PL181205094', 'TSH-PL181205100', 'TSH-PL181205101', 'TSH-PL181205106',
'TSH-PL181205112', 'TSH-PL181205118', 'TSH-PL181205119', 'TSH-PL181205120', 'TSH-PL181205123',
'TSH-PL181205124', 'TSH-PL181205153', 'TSH-PL181205154', 'TSH-PL181205159', 'TSH-PL181205163']

post_diagnosis_samples_colon = ['TSH-PL180510139', 'TSH-PL180510140', 'TSH-PL180510146', 'TSH-
PL180510147', 'TSH-PL180510162', 'TSH-PL181205175', 'TSH-PL180510143']
post_diagnosis_samples_liver = ['TSH-PL180510085', 'TSH-PL180510089', 'TSH-PL180510092', 'TSH-
PL180510095', 'TSH-PL180510101', 'TSH-PL180510104', 'TSH-PL180510105', 'TSH-PL180510107', 'TSH
-PL180510112', 'TSH-PL180510121', 'TSH-PL180510122', 'TSH-PL180510125', 'TSH-PL181205173', 'TS
H-PL180510088', 'TSH-PL180510090', 'TSH-PL180510098', 'TSH-PL180510100', 'TSH-PL180510103', 'T
SH-PL180510108', 'TSH-PL180510109', 'TSH-PL180510113', 'TSH-PL180510117', 'TSH-PL180510124']
post_diagnosis_samples_esophagus = ['TSH-PL171221101', 'TSH-PL171221112', 'TSH-PL171221149', '
TSH-PL171221150', 'TSH-PL171221160', 'TSH-PL171221172', 'TSH-PL171221179', 'TSH-PL171221180',
'TSH-PL180508001', 'TSH-PL180508004', 'TSH-PL180508005', 'TSH-PL180508006', 'TSH-PL180508008',
'TSH-PL180508009', 'TSH-PL180508011', 'TSH-PL180508012', 'TSH-PL180508013', 'TSH-PL180508014',
'TSH-PL180508015', 'TSH-PL180508019', 'TSH-PL180508020', 'TSH-PL180508021', 'TSH-PL180508022',
'TSH-PL180508025', 'TSH-PL180508030', 'TSH-PL180508031', 'TSH-PL180508033', 'TSH-PL180508034',
'TSH-PL180508036', 'TSH-PL180508037', 'TSH-PL180508038', 'TSH-PL180508039', 'TSH-PL180508040',
'TSH-PL180508041', 'TSH-PL180508042', 'TSH-PL180508043', 'TSH-PL180508049', 'TSH-PL180508050',
'TSH-PL180508060', 'TSH-PL180508061', 'TSH-PL180508064', 'TSH-PL180508068', 'TSH-PL180508076',
'TSH-PL180508078', 'TSH-PL180508083', 'TSH-PL180508086', 'TSH-PL180508089', 'TSH-PL180510044',
'TSH-PL180510061', 'TSH-PL181205168', 'TSH-PL181205170', 'TSH-PL171221151', 'TSH-PL180508003',
'TSH-PL180508007', 'TSH-PL180508023', 'TSH-PL180508026', 'TSH-PL180508028', 'TSH-PL180508029',
'TSH-PL180508032', 'TSH-PL180508044', 'TSH-PL180508052', 'TSH-PL180508065', 'TSH-PL180508067',
'TSH-PL180508088', 'TSH-PL181205172', 'TSH-PL181205174', 'TSH-PL171221153', 'TSH-PL171221113']
post_diagnosis_samples_lung = ['TSH-PL180510005', 'TSH-PL180510006', 'TSH-PL180510007', 'TSH-P
L180510008', 'TSH-PL180510010', 'TSH-PL180510011', 'TSH-PL180510012', 'TSH-PL180510014', 'TSH-
PL180510015', 'TSH-PL180510016', 'TSH-PL180510017', 'TSH-PL180510018', 'TSH-PL180510019', 'TSH
-PL180510021', 'TSH-PL180510023', 'TSH-PL180510024', 'TSH-PL180510025', 'TSH-PL180510026', 'TS
H-PL180510030', 'TSH-PL180510031', 'TSH-PL180510033', 'TSH-PL180510038', 'TSH-PL180510040', 'T
SH-PL180510047', 'TSH-PL180510050', 'TSH-PL180510052', 'TSH-PL180510053', 'TSH-PL180510054', '
TSH-PL180510055', 'TSH-PL180510057', 'TSH-PL180510063', 'TSH-PL180510066', 'TSH-PL180510071',
'TSH-PL180510072', 'TSH-PL180510073', 'TSH-PL180510074', 'TSH-PL180510075', 'TSH-PL180510076',
'TSH-PL180510077', 'TSH-PL180510078', 'TSH-PL180510079', 'TSH-PL180510080', 'TSH-PL181205182',
'TSH-PL180510002', 'TSH-PL180510004', 'TSH-PL180510013', 'TSH-PL180510020', 'TSH-PL180510022',
'TSH-PL180510032', 'TSH-PL180510034', 'TSH-PL180510035', 'TSH-PL180510043', 'TSH-PL180510048',
'TSH-PL180510068', 'TSH-PL181205177', 'TSH-PL181205179']
post_diagnosis_samples_stomach = ['TSH-PL180504001', 'TSH-PL180504002', 'TSH-PL180504003', 'TS
H-PL180504004', 'TSH-PL180504005', 'TSH-PL180504009', 'TSH-PL180504010', 'TSH-PL180504011', 'T
SH-PL180504013', 'TSH-PL180504014', 'TSH-PL180504015', 'TSH-PL180504016', 'TSH-PL180504017', '
TSH-PL180504018', 'TSH-PL180504019', 'TSH-PL180504021', 'TSH-PL180504022', 'TSH-PL180504027',
'TSH-PL180504028', 'TSH-PL180504030', 'TSH-PL180504033', 'TSH-PL180504034', 'TSH-PL180504041',
'TSH-PL180504042', 'TSH-PL180504044', 'TSH-PL180504046', 'TSH-PL180504047', 'TSH-PL180504049',
'TSH-PL180504050', 'TSH-PL180504053', 'TSH-PL180504054', 'TSH-PL180504058', 'TSH-PL180504061',
'TSH-PL180504062', 'TSH-PL180504063', 'TSH-PL180504065', 'TSH-PL180504068', 'TSH-PL180504070',
'TSH-PL180504074', 'TSH-PL180504081', 'TSH-PL180504083', 'TSH-PL180504087', 'TSH-PL180504088',
'TSH-PL180504089', 'TSH-PL180504091', 'TSH-PL180504094', 'TSH-PL180504097', 'TSH-PL180504101',
'TSH-PL180504104', 'TSH-PL180504108', 'TSH-PL180504111', 'TSH-PL180504118', 'TSH-PL180504006',
'TSH-PL180504012', 'TSH-PL180504024', 'TSH-PL180504026', 'TSH-PL180504029', 'TSH-PL180504037',
'TSH-PL180504043', 'TSH-PL180504045', 'TSH-PL180504051', 'TSH-PL180504052', 'TSH-PL180504055',
'TSH-PL180504057', 'TSH-PL180504064', 'TSH-PL180504076', 'TSH-PL180504084', 'TSH-PL180504110',
'TSH-PL181205169']

pre_diagnosis_samples_colon = ['TSH-PL180123001', 'TSH-PL180123010', 'TSH-PL180513013', 'TSH-P
L180513014', 'TSH-PL180513020', 'TSH-PL180513027', 'TSH-PL180513029', 'TSH-PL180513030', 'TSH-
PL180513034', 'TSH-PL180513037', 'TSH-PL180513049', 'TSH-PL180513062', 'TSH-PL180513077', 'TSH
-PL180513078', 'TSH-PL180513085', 'TSH-PL180513114', 'TSH-PL180516003', 'TSH-PL180516011', 'TS
H-PL180516012', 'TSH-PL180516014', 'TSH-PL180516018', 'TSH-PL180516021', 'TSH-PL180516028', 'T
SH-PL180516039', 'TSH-PL180516044', 'TSH-PL180516053', 'TSH-PL180516057', 'TSH-PL180513001', '
TSH-PL180513002', 'TSH-PL180513064', 'TSH-PL180513116', 'TSH-PL180516015', 'TSH-PL180516017',
'TSH-PL180516026', 'TSH-PL180516033']
pre_diagnosis_samples_liver = ['TSH-PL180123014', 'TSH-PL180123031', 'TSH-PL180513006', 'TSH-P
L180513011', 'TSH-PL180513017', 'TSH-PL180513021', 'TSH-PL180513026', 'TSH-PL180513041', 'TSH-
PL180513043', 'TSH-PL180513071', 'TSH-PL180513088', 'TSH-PL180513095', 'TSH-PL180513098', 'TSH
-PL180513108', 'TSH-PL180513109', 'TSH-PL180513111', 'TSH-PL180513117', 'TSH-PL180516006', 'TS
H-PL180516038', 'TSH-PL180516040', 'TSH-PL180516051', 'TSH-PL180516054', 'TSH-PL180516063', 'T
SH-PL180123004', 'TSH-PL180123005', 'TSH-PL180513009', 'TSH-PL180513033', 'TSH-PL180513090', '
TSH-PL180516025']
pre_diagnosis_samples_esophagus = ['TSH-PL180123006', 'TSH-PL180123007', 'TSH-PL180123008', 'T
SH-PL180123016', 'TSH-PL180123025', 'TSH-PL180513004', 'TSH-PL180513005', 'TSH-PL180513016', '
TSH-PL180513051', 'TSH-PL180513068', 'TSH-PL180513072', 'TSH-PL180513075', 'TSH-PL180513080',
'TSH-PL180513081', 'TSH-PL180513091', 'TSH-PL180513093', 'TSH-PL180513097', 'TSH-PL180513100',
'TSH-PL180513101', 'TSH-PL180513110', 'TSH-PL180513115', 'TSH-PL180513118', 'TSH-PL180513121',
'TSH-PL180513122', 'TSH-PL180513124', 'TSH-PL180513128', 'TSH-PL180513129', 'TSH-PL180516013',
'TSH-PL180516020', 'TSH-PL180516030', 'TSH-PL180516031', 'TSH-PL180516045', 'TSH-PL180516049',
'TSH-PL180516059', 'TSH-PL180123024', 'TSH-PL180123032', 'TSH-PL180513028', 'TSH-PL180513032',
'TSH-PL180513058', 'TSH-PL180513083', 'TSH-PL180513096', 'TSH-PL180516005', 'TSH-PL180516047',
'TSH-PL180516050', 'TSH-PL180516061']
pre_diagnosis_samples_lung = ['TSH-PL180123013', 'TSH-PL180123021', 'TSH-PL180123022', 'TSH-PL
180123029', 'TSH-PL180123030', 'TSH-PL180513007', 'TSH-PL180513012', 'TSH-PL180513023', 'TSH-P
L180513024', 'TSH-PL180513036', 'TSH-PL180513040', 'TSH-PL180513045', 'TSH-PL180513055', 'TSH-
PL180513056', 'TSH-PL180513057', 'TSH-PL180513067', 'TSH-PL180513069', 'TSH-PL180513079', 'TSH
-PL180513084', 'TSH-PL180513087', 'TSH-PL180513092', 'TSH-PL180513106', 'TSH-PL180516001', 'TS
H-PL180516002', 'TSH-PL180516004', 'TSH-PL180516016', 'TSH-PL180516023', 'TSH-PL180516032', 'T
SH-PL180516055', 'TSH-PL180516056', 'TSH-PL180516062', 'TSH-PL180123002', 'TSH-PL180123012', '
TSH-PL180513010', 'TSH-PL180513019', 'TSH-PL180513022', 'TSH-PL180513031', 'TSH-PL180513035',
'TSH-PL180513044', 'TSH-PL180513086', 'TSH-PL180513112', 'TSH-PL180516029', 'TSH-PL180516034',
'TSH-PL180516046', 'TSH-PL180516048', 'TSH-PL180516058', 'TSH-PL180516060']
pre_diagnosis_samples_stomach = ['TSH-PL180123017', 'TSH-PL180123018', 'TSH-PL180123027', 'TSH
-PL180123033', 'TSH-PL180513025', 'TSH-PL180513038', 'TSH-PL180513039', 'TSH-PL180513050', 'TS
H-PL180513060', 'TSH-PL180513066', 'TSH-PL180513070', 'TSH-PL180513073', 'TSH-PL180513076', 'T
SH-PL180513089', 'TSH-PL180513099', 'TSH-PL180513102', 'TSH-PL180513104', 'TSH-PL180513119', '
TSH-PL180513123', 'TSH-PL180513127', 'TSH-PL180516007', 'TSH-PL180516008', 'TSH-PL180516009',
'TSH-PL180516019', 'TSH-PL180516027', 'TSH-PL180516036', 'TSH-PL180516042', 'TSH-PL180516043',
'TSH-PL180513003', 'TSH-PL180513018', 'TSH-PL180513103', 'TSH-PL180513105', 'TSH-PL180513125',
'TSH-PL180516010', 'TSH-PL180516052']
```

BioChain Tissue Samples

In [7]:
```python
healthy_lung_tissue = ['BC_Lung_ND1', 'BC_Lung_ND2', 'BC_Lung_ND3', 'BC_Lung_ND4', 'BC_Lung_ND
5', 'BC_Lung_ND6', 'BC_Lung_ND7', 'BC_Lung_ND8']
healthy_colon_tissue = ['BC_CRC_ND1', 'BC_CRC_ND2', 'BC_CRC_ND3', 'BC_CRC_ND4', 'BC_CRC_ND5',
'BC_CRC_ND6', 'BC_CRC_ND7', 'BC_CRC_ND8']
healthy_stomach_tissue = ['BC_Stomach_ND1', 'BC_Stomach_ND2', 'BC_Stomach_ND3', 'BC_Stomach_ND
4', 'BC_Stomach_ND5', 'BC_Stomach_ND6', 'BC_Stomach_ND7', 'BC_Stomach_ND8']
healthy_breast_tissue = ['BC_Breast_ND1', 'BC_Breast_ND2', 'BC_Breast_ND3', 'BC_Breast_ND4', '
BC_Breast_ND5', 'BC_Breast_ND6', 'BC_Breast_ND7', 'BC_Breast_ND8']

cancer_lung_tissue = ['BC_Lung_TD1', 'BC_Lung_TD10', 'BC_Lung_TD11', 'BC_Lung_TD12', 'BC_Lung_
TD13', 'BC_Lung_TD14', 'BC_Lung_TD15', 'BC_Lung_TD16', 'BC_Lung_TD17', 'BC_Lung_TD18', 'BC_Lun
g_TD19', 'BC_Lung_TD2', 'BC_Lung_TD20', 'BC_Lung_TD21', 'BC_Lung_TD22', 'BC_Lung_TD23', 'BC_Lu
ng_TD24', 'BC_Lung_TD25', 'BC_Lung_TD26', 'BC_Lung_TD27', 'BC_Lung_TD28', 'BC_Lung_TD29', 'BC_
Lung_TD3', 'BC_Lung_TD30', 'BC_Lung_TD31', 'BC_Lung_TD32', 'BC_Lung_TD33', 'BC_Lung_TD34', 'BC
_Lung_TD35', 'BC_Lung_TD36', 'BC_Lung_TD37', 'BC_Lung_TD38', 'BC_Lung_TD39', 'BC_Lung_TD4', 'B
C_Lung_TD40', 'BC_Lung_TD5', 'BC_Lung_TD6', 'BC_Lung_TD7', 'BC_Lung_TD8', 'BC_Lung_TD9']
cancer_colon_tissue = ['BC_CRC_TD1', 'BC_CRC_TD10', 'BC_CRC_TD11', 'BC_CRC_TD12', 'BC_CRC_TD13
', 'BC_CRC_TD14', 'BC_CRC_TD15', 'BC_CRC_TD16', 'BC_CRC_TD17', 'BC_CRC_TD18', 'BC_CRC_TD19', '
BC_CRC_TD2', 'BC_CRC_TD20', 'BC_CRC_TD21', 'BC_CRC_TD22', 'BC_CRC_TD23', 'BC_CRC_TD24', 'BC_CR
C_TD25', 'BC_CRC_TD26', 'BC_CRC_TD27', 'BC_CRC_TD28', 'BC_CRC_TD29', 'BC_CRC_TD3', 'BC_CRC_TD3
0', 'BC_CRC_TD31', 'BC_CRC_TD32', 'BC_CRC_TD33', 'BC_CRC_TD34', 'BC_CRC_TD35', 'BC_CRC_TD36',
'BC_CRC_TD37', 'BC_CRC_TD38', 'BC_CRC_TD39', 'BC_CRC_TD4', 'BC_CRC_TD40', 'BC_CRC_TD5', 'BC_CR
C_TD6', 'BC_CRC_TD7', 'BC_CRC_TD8', 'BC_CRC_TD9']
cancer_stomach_tissue = ['BC_Stomach_TD1', 'BC_Stomach_TD10', 'BC_Stomach_TD11', 'BC_Stomach_T
D12', 'BC_Stomach_TD13', 'BC_Stomach_TD14', 'BC_Stomach_TD15', 'BC_Stomach_TD16', 'BC_Stomach_
TD17', 'BC_Stomach_TD18', 'BC_Stomach_TD19', 'BC_Stomach_TD2', 'BC_Stomach_TD20', 'BC_Stomach_
TD21', 'BC_Stomach_TD22', 'BC_Stomach_TD23', 'BC_Stomach_TD24', 'BC_Stomach_TD25', 'BC_Stomach
_TD26', 'BC_Stomach_TD27', 'BC_Stomach_TD28', 'BC_Stomach_TD29', 'BC_Stomach_TD3', 'BC_Stomach
_TD30', 'BC_Stomach_TD31', 'BC_Stomach_TD32', 'BC_Stomach_TD33', 'BC_Stomach_TD34', 'BC_Stomac
h_TD35', 'BC_Stomach_TD36', 'BC_Stomach_TD37', 'BC_Stomach_TD38', 'BC_Stomach_TD39', 'BC_Stoma
ch_TD4', 'BC_Stomach_TD40', 'BC_Stomach_TD5', 'BC_Stomach_TD6', 'BC_Stomach_TD7', 'BC_Stomach_
TD8', 'BC_Stomach_TD9']
cancer_breast_tissue = ['BC_Breast_TD1', 'BC_Breast_TD10', 'BC_Breast_TD11', 'BC_Breast_TD12',
'BC_Breast_TD13', 'BC_Breast_TD14', 'BC_Breast_TD15', 'BC_Breast_TD16', 'BC_Breast_TD17', 'BC_
Breast_TD18', 'BC_Breast_TD19', 'BC_Breast_TD2', 'BC_Breast_TD20', 'BC_Breast_TD21', 'BC_Breas
t_TD22', 'BC_Breast_TD23', 'BC_Breast_TD24', 'BC_Breast_TD25', 'BC_Breast_TD26', 'BC_Breast_TD
27', 'BC_Breast_TD28', 'BC_Breast_TD29', 'BC_Breast_TD3', 'BC_Breast_TD30', 'BC_Breast_TD31',
'BC_Breast_TD32', 'BC_Breast_TD33', 'BC_Breast_TD34', 'BC_Breast_TD35', 'BC_Breast_TD36', 'BC_
Breast_TD37', 'BC_Breast_TD38', 'BC_Breast_TD39', 'BC_Breast_TD4', 'BC_Breast_TD40', 'BC_Breas
t_TD5', 'BC_Breast_TD6', 'BC_Breast_TD7', 'BC_Breast_TD8', 'BC_Breast_TD9']

all_tissue_samples = healthy_lung_tissue+healthy_colon_tissue+healthy_stomach_tissue+healthy_b
reast_tissue+cancer_lung_tissue+cancer_colon_tissue+cancer_stomach_tissue+cancer_breast_tissue
```

Load Data

Let's load a data matrix listing the average methylation across each target region for each sample.

In [8]:
```python
amf_df_orig = pd.read_csv('SupplementaryData5.tsv', sep="\t", header=0, index_col=0)
```

Marker Selection

Let's first remove markers that are not covered in all plasma samples, so we don't have to deal with any missing values.

In [9]:
```python
amf_df = amf_df_orig.copy()
amf_df = amf_df.loc[amf_df[healthy_samples+post_diagnosis_samples+pre_diagnosis_samples].dropn
a(how='any').index]
```

Next, let's choose markers that separate healthy tissue from cancer tissue.

For each tissue type we purchased from BioChain, we will use a T-test to identify target regions that separate normal tissue from cancer tissue (applying a Benjamini-Hochberg multiple testing correction).

In [10]:
```python
tissue_t_tests = pd.concat(
    [
        pd.Series(amf_df.index),
        pd.Series(scipy.stats.ttest_ind(amf_df.loc[:, healthy_colon_tissue].T,
                                        amf_df.loc[:, cancer_colon_tissue].T,
                                        axis=0, equal_var=True, nan_policy='omit').pvalue),
        pd.Series(scipy.stats.ttest_ind(amf_df.loc[:, healthy_lung_tissue].T,
                                        amf_df.loc[:, cancer_lung_tissue].T,
                                        axis=0, equal_var=True, nan_policy='omit').pvalue),
        pd.Series(scipy.stats.ttest_ind(amf_df.loc[:, healthy_stomach_tissue].T,
                                        amf_df.loc[:, cancer_stomach_tissue].T,
                                        axis=0, equal_var=True, nan_policy='omit').pvalue),
        pd.Series(scipy.stats.ttest_ind(amf_df.loc[:, healthy_breast_tissue].T,
                                        amf_df.loc[:, cancer_breast_tissue].T,
                                        axis=0, equal_var=True, nan_policy='omit').pvalue),
    ], axis=1
)

tissue_t_tests.columns = ['Locus',
                          'ColonM', 'LungM', 'StomachM', 'BreastM',
                         ]
tissue_t_tests = tissue_t_tests.set_index('Locus')

# Benjamini-Hochberg Correction
from statsmodels.stats.multitest import multipletests
_, pvals_corrected, _, _ = multipletests(tissue_t_tests.min(axis=1).fillna(1).values, alpha=0.
05, method='fdr_bh')

tissue_t_tests_min_by_marker = tissue_t_tests.loc[pvals_corrected <= 0.05].iloc[:, :4].min(axi
s=1).copy()
```

In [11]:
```python
print len(tissue_t_tests_min_by_marker)
```

```
477
```

We can then use these target regions in our plasma samples to build a classifier.

Divide Samples Into Leave-In and Leave-Out

Let's first split the Taizhou samples into two sets: leave-in (1/2) and leave-out (1/2). The leave-in set will be used to build a model.

We want to make sure that equal proportions of healthy/cancer samples are included in the leave-in/leave-out set. We also want balance within each type of cancer (so we don't end up with no samples for one cancer type in the leave-out set, for example).

In [16]:
```python
random.seed(128)

# Healthy
random.shuffle(healthy_samples)
# Post-Diagnosis
random.shuffle(post_diagnosis_samples_colon)
random.shuffle(post_diagnosis_samples_liver)
random.shuffle(post_diagnosis_samples_esophagus)
random.shuffle(post_diagnosis_samples_lung)
random.shuffle(post_diagnosis_samples_stomach)
# Pre-Diagnosis
random.shuffle(pre_diagnosis_samples_colon)
random.shuffle(pre_diagnosis_samples_liver)
random.shuffle(pre_diagnosis_samples_esophagus)
random.shuffle(pre_diagnosis_samples_lung)
random.shuffle(pre_diagnosis_samples_stomach)

negative_leavein = list(healthy_samples[:int(len(healthy_samples)/2)])
negative_leaveout = list(healthy_samples[int(len(healthy_samples)/2):])

positive_post_leavein = post_diagnosis_samples_colon[:int(len(post_diagnosis_samples_colon)/2)
] + post_diagnosis_samples_liver[:int(len(post_diagnosis_samples_liver)/2)] + post_diagnosis_s
amples_esophagus[:int(len(post_diagnosis_samples_esophagus)/2)] + post_diagnosis_samples_lung[
:int(len(post_diagnosis_samples_lung)/2)] + post_diagnosis_samples_stomach[:int(len(post_diagn
osis_samples_stomach)/2)]
positive_post_leaveout = post_diagnosis_samples_colon[int(len(post_diagnosis_samples_colon)/2)
:] + post_diagnosis_samples_liver[int(len(post_diagnosis_samples_liver)/2):] + post_diagnosis_
samples_esophagus[int(len(post_diagnosis_samples_esophagus)/2):] + post_diagnosis_samples_lung
[int(len(post_diagnosis_samples_lung)/2):] + post_diagnosis_samples_stomach[int(len(post_diagn
osis_samples_stomach)/2):]

positive_pre_leavein = pre_diagnosis_samples_colon[:int(len(pre_diagnosis_samples_colon)/2)] +
pre_diagnosis_samples_liver[:int(len(pre_diagnosis_samples_liver)/2)] + pre_diagnosis_samples_
esophagus[:int(len(pre_diagnosis_samples_esophagus)/2)] + pre_diagnosis_samples_lung[:int(len(
pre_diagnosis_samples_lung)/2)] + pre_diagnosis_samples_stomach[:int(len(pre_diagnosis_samples
_stomach)/2)]
positive_pre_leaveout = pre_diagnosis_samples_colon[int(len(pre_diagnosis_samples_colon)/2):]
+ pre_diagnosis_samples_liver[int(len(pre_diagnosis_samples_liver)/2):] + pre_diagnosis_sample
s_esophagus[int(len(pre_diagnosis_samples_esophagus)/2):] + pre_diagnosis_samples_lung[int(len
(pre_diagnosis_samples_lung)/2):] + pre_diagnosis_samples_stomach[int(len(pre_diagnosis_sample
s_stomach)/2):]
```

Let's next further filter the markers using the leave-in samples to require the same direction of change as the cancer tissues.

In [18]:
```python
healthy_plasma_mean = amf_df.loc[tissue_t_tests_min_by_marker.index, negative_leavein].mean(ax
is=1)
cancer_plasma_mean = amf_df.loc[tissue_t_tests_min_by_marker.index, positive_post_leavein+posi
tive_pre_leavein].mean(axis=1)

healthy_tissue_mean = amf_df.loc[tissue_t_tests_min_by_marker.index, healthy_lung_tissue+healt
hy_colon_tissue+healthy_stomach_tissue+healthy_breast_tissue].mean(axis=1)
cancer_tissue_mean = amf_df.loc[tissue_t_tests_min_by_marker.index, cancer_lung_tissue+cancer_
colon_tissue+cancer_stomach_tissue+cancer_breast_tissue].mean(axis=1)
```

In [28]:
```python
tissue_t_tests_min_by_marker = tissue_t_tests_min_by_marker[(
        (healthy_plasma_mean <= cancer_plasma_mean) & (healthy_tissue_mean <= cancer_tissue_me
an)
        |
        (healthy_plasma_mean > cancer_plasma_mean) & (healthy_tissue_mean > cancer_tissue_mean
)
    )]
```

Build a Logistic Regression Classifier

Now, let's build an LR classifier from the leave-in set. We will use the method described in Teschendorff et al, Nature Materials (2019). Briefly:

1. We will randomly split the leave-in set into 50% training, 50% test.
2. We will train an LR classifier on the training set, and compute the results on the test set.
3. We will repeat steps 1 and 2 using 1000 different random splits.
4. We will then compute the ensemble performance of these 1000 models across the ten separate test sets, and report this as the "leave-in set" accuracy.
5. We will then apply all 1000 models to the "leave-out set," compute the ensemble performance, and report this as the "leave-out set" accuracy.

Let's build each LR classifier using 1000 different random splits:

In [29]:
```python
negative_test_list = []
positive_post_test_list = []
positive_pre_test_list = []
z_prob_list = []

for random_state in range(1000):
    random.seed(random_state)

    # Set up LR classifier that will automatically fit the best C value using the training set
    clf = LogisticRegressionCV(penalty='l1', solver='liblinear', random_state=random_state,
                               Cs=[1.0, 5.0, 10.0, 50.0, 100.0], cv=3)

    # Split the leave-in set into training and test set
    negative = list(negative_leavein)
    positive_post = list(positive_post_leavein)
    positive_pre = list(positive_pre_leavein)
    random.shuffle(negative)
    random.shuffle(positive_post)
    random.shuffle(positive_pre)
    negative_training, negative_test = negative[:1*int(len(negative)/2)], negative[int(1*len(n
egative)/2):]
    positive_post_training, positive_post_test = positive_post[:1*int(len(positive_post)/2)],
positive_post[int(1*len(positive_post)/2):]
    positive_pre_training, positive_pre_test = positive_pre[:1*int(len(positive_pre)/2)], posi
tive_pre[int(1*len(positive_pre)/2):]
    positive_training = positive_post_training+positive_pre_training
    positive_test = positive_post_test+positive_pre_test

    # Build the model and set parameters using the training set
    x = amf_df.loc[tissue_t_tests_min_by_marker.index, negative_training+positive_training].tr
anspose()
    y = ['Healthy']*len(negative_training) + ['Cancer']*len(positive_training)
    clf.fit(x,y)

    # Compute the accuracy of this classifier on the leave-in and leave-out sets
    z = pd.Series(clf.predict(amf_df.loc[tissue_t_tests_min_by_marker.index, healthy_samples+p
ost_diagnosis_samples+pre_diagnosis_samples].transpose()),
                  index=healthy_samples+post_diagnosis_samples+pre_diagnosis_samples)
    z_prob = pd.Series(clf.predict_proba(amf_df.loc[tissue_t_tests_min_by_marker.index, health
y_samples+post_diagnosis_samples+pre_diagnosis_samples].transpose())[:, 0],
                       index=healthy_samples+post_diagnosis_samples+pre_diagnosis_samples)

    # Store the results
    negative_test_list.append(negative_test)
    positive_post_test_list.append(positive_post_test)
    positive_pre_test_list.append(positive_pre_test)
    z_prob_list.append(z_prob)
```

Next, let's compute the ensemble performance across the leave-in test sets and the leave-out sets.

In [30]:
```python
ensemble_score_leavein = pd.concat([z_prob_list[index].loc[negative_test_list[index]+positive_
pre_test_list[index]+positive_post_test_list[index]]
                                    for index in range(1000)],
                                   axis=1).mean(axis=1)
ensemble_score_leaveout = pd.concat([z_prob_list[index].loc[negative_leaveout+positive_post_le
aveout+positive_pre_leaveout]
                                     for index in range(1000)],
                                    axis=1).mean(axis=1)
```

We can now compute accuracy metrics for the two sets; as per Teschendorff et al 2019, we will choose the model cutoff using the leave-in set.

Leave-In Set Accuracy

Let's choose a cutoff that aims for ~95% specificity in the leave-in set, and compute the associated sensitivity.

In [53]:
```python
sns.set_style('whitegrid')

y_true = ['Healthy']*len(negative_leavein) + ['Cancer']*len(positive_post_leavein)
y_score = ensemble_score_leavein.loc[negative_leavein+positive_post_leavein]
cur_fpr, cur_tpr, cur_cutoff = roc_curve(y_true, y_score, pos_label='Cancer')
cur_auc = auc(cur_fpr, cur_tpr)
plt.plot(cur_fpr, cur_tpr, label=r'Post-Diagnosis ROC (AUC = %0.2f)' % cur_auc)

y_true = ['Healthy']*len(negative_leavein) + ['Cancer']*len(positive_pre_leavein)
y_score = ensemble_score_leavein.loc[negative_leavein+positive_pre_leavein]
cur_fpr, cur_tpr, cur_cutoff = roc_curve(y_true, y_score, pos_label='Cancer')
cur_auc = auc(cur_fpr, cur_tpr)
plt.plot(cur_fpr, cur_tpr, label=r'Pre-Diagnosis ROC (AUC = %0.2f)' % cur_auc)

plt.xlim([-0.0, 1.0])
plt.ylim([-0.0, 1.0])
plt.xticks([0, 0.05, 0.1, 0.2, 0.3, 0.4, 0.5, 0.6, 0.7, 0.8, 0.9, 1.0])
plt.yticks([0, 0.05, 0.1, 0.2, 0.3, 0.4, 0.5, 0.6, 0.7, 0.8, 0.9, 1.0])
plt.xlabel('False Positive Rate')
plt.ylabel('True Positive Rate')
plt.legend(loc="lower right");

print "Leave-In Results"
print "Specificity: %s" % (sum(ensemble_score_leavein.loc[negative_leavein] < 0.655) / len(neg
ative_leavein))
print "Post-Diagnosis Sensitivity: %s" % (sum(ensemble_score_leavein.loc[positive_post_leavein
] >= 0.655) / len(positive_post_leavein))
print "Pre-Diagnosis Sensitivity: %s" % (sum(ensemble_score_leavein.loc[positive_pre_leavein]
>= 0.655) / len(positive_pre_leavein))

plt.figure()
sns.distplot(ensemble_score_leavein.loc[negative_leavein])
sns.distplot(ensemble_score_leavein.loc[positive_post_leavein])
sns.distplot(ensemble_score_leavein.loc[positive_pre_leavein])
plt.legend(['Healthy', 'Post-Diagnosis', 'Pre-Diagnosis']);
plt.xlim(0, 1)
```

```
Leave-In Results
Specificity: 0.946859903382
Post-Diagnosis Sensitivity: 0.845454545455
Pre-Diagnosis Sensitivity: 0.870967741935
```

Out[53]: (0, 1)

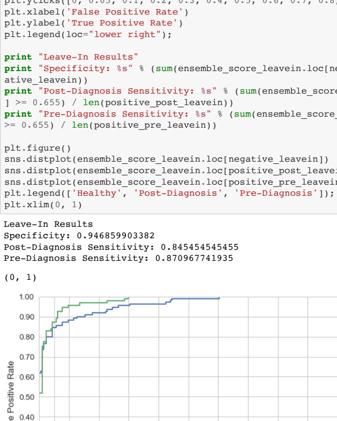

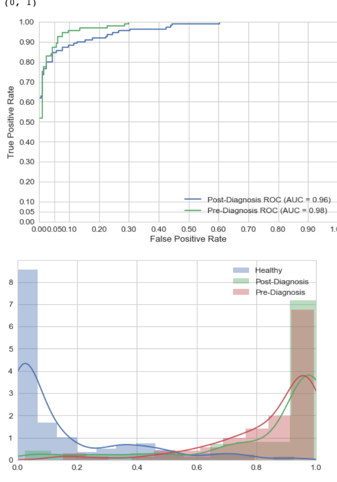

Leave-Out Set Accuracy

Let's compute the leave-out set sensitivity and specificity using the previously chosen cutoff.

In [54]:
```python
sns.set_style('whitegrid')

y_true = ['Healthy']*len(negative_leaveout) + ['Cancer']*len(positive_post_leaveout)
y_score = ensemble_score_leaveout.loc[negative_leaveout+positive_post_leaveout]
cur_fpr, cur_tpr, cur_cutoff = roc_curve(y_true, y_score, pos_label='Cancer')
cur_auc = auc(cur_fpr, cur_tpr)
plt.plot(cur_fpr, cur_tpr, label=r'Post-Diagnosis ROC (AUC = %0.2f)' % cur_auc)

y_true = ['Healthy']*len(negative_leaveout) + ['Cancer']*len(positive_pre_leaveout)
y_score = ensemble_score_leaveout.loc[negative_leaveout+positive_pre_leaveout]
cur_fpr, cur_tpr, cur_cutoff = roc_curve(y_true, y_score, pos_label='Cancer')
cur_auc = auc(cur_fpr, cur_tpr)
plt.plot(cur_fpr, cur_tpr, label=r'Pre-Diagnosis ROC (AUC = %0.2f)' % cur_auc)

plt.xlim([-0.0, 1.0])
plt.ylim([-0.0, 1.0])
plt.xticks([0, 0.05, 0.1, 0.2, 0.3, 0.4, 0.5, 0.6, 0.7, 0.8, 0.9, 1.0])
plt.yticks([0, 0.05, 0.1, 0.2, 0.3, 0.4, 0.5, 0.6, 0.7, 0.8, 0.9, 1.0])
plt.xlabel('False Positive Rate')
plt.ylabel('True Positive Rate')
plt.legend(loc="lower right");

print "Leave-Out Results"
print "Specificity: %s" % (sum(ensemble_score_leaveout.loc[negative_leaveout] < 0.655) / len(n
egative_leaveout))
print "Post-Diagnosis Sensitivity: %s" % (sum(ensemble_score_leaveout.loc[positive_post_leaveo
ut] >= 0.655) / len(positive_post_leaveout))
print "Pre-Diagnosis Sensitivity: %s" % (sum(ensemble_score_leaveout.loc[positive_pre_leaveout
] >= 0.655) / len(positive_pre_leaveout))

plt.figure()
sns.distplot(ensemble_score_leaveout.loc[negative_leaveout])
sns.distplot(ensemble_score_leaveout.loc[positive_post_leaveout])
sns.distplot(ensemble_score_leaveout.loc[positive_pre_leaveout])
plt.legend(['Healthy', 'Post-Diagnosis', 'Pre-Diagnosis']);
plt.xlim(0, 1)
```

```
Leave-Out Results
Specificity: 0.951690821256
Post-Diagnosis Sensitivity: 0.849557522124
Pre-Diagnosis Sensitivity: 0.897959183673
```

Out[54]: (0, 1)

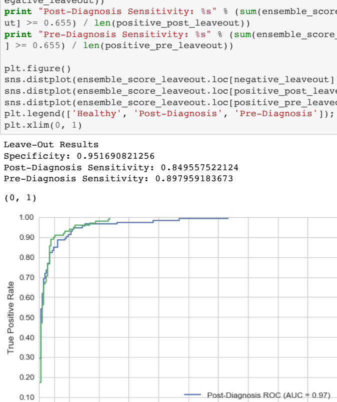

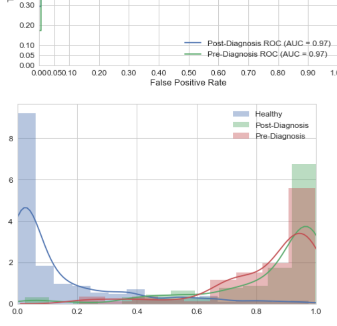

As per Teschendorff et al 2019, the accuracy is similar for the leave-in and leave-out data sets.

Compare T-Statistics for Tissue and Plasma

Let's also generate a plot comparing the t-statistics for tissue and plasma samples.

In [62]:
```
t_value_df = pd.concat(
    [
        pd.Series(amf_df.index),
        pd.Series(scipy.stats.ttest_ind(amf_df.loc[:, healthy_colon_tissue].T,
                                        amf_df.loc[:, cancer_colon_tissue].T,
                                        axis=0, equal_var=True, nan_policy='omit').statistic),
        pd.Series(scipy.stats.ttest_ind(amf_df.loc[:, healthy_lung_tissue].T,
                                        amf_df.loc[:, cancer_lung_tissue].T,
                                        axis=0, equal_var=True, nan_policy='omit').statistic),
        pd.Series(scipy.stats.ttest_ind(amf_df.loc[:, healthy_stomach_tissue].T,
                                        amf_df.loc[:, cancer_stomach_tissue].T,
                                        axis=0, equal_var=True, nan_policy='omit').statistic),
        pd.Series(scipy.stats.ttest_ind(amf_df.loc[:, healthy_breast_tissue].T,
                                        amf_df.loc[:, cancer_breast_tissue].T,
                                        axis=0, equal_var=True, nan_policy='omit').statistic),
        pd.Series(scipy.stats.ttest_ind(amf_df.loc[:, healthy_samples].T,
                                        amf_df.loc[:, post_diagnosis_samples].T,
                                        axis=0, equal_var=True, nan_policy='omit').statistic),
        pd.Series(scipy.stats.ttest_ind(amf_df.loc[:, healthy_samples].T,
                                        amf_df.loc[:, pre_diagnosis_samples].T,
                                        axis=0, equal_var=True, nan_policy='omit').statistic),
    ], axis=1
)

t_value_df.columns = ['Locus',
                      'ColonM', 'LungM', 'StomachM', 'BreastM', 'PostDiagnosisM', 'PreDiagnosi
sM'
                     ]
t_value_df = t_value_df.set_index('Locus')
```

When plotting these t-statistics, due to the differences in both **scale of methylation changes** (i.e. difference of means) and **variability of methylation changes** (i.e. standard deviation), **the t-statistics did not show a very clear pattern.**

This is because tissue and plasma samples have a very different DNA composition, making a direct comparison of t-statistics difficult.

However, even when limiting analyzed markers to the bottom left and top right quadrants of these plots (i.e. markers that behave concordantly between tissue and plasma), high sensitivity and specificity can still be achieved.

In [65]:
```
sns.pairplot(t_value_df)
```

Out[65]: <seaborn.axisgrid.PairGrid at 0x119bbd4d0>

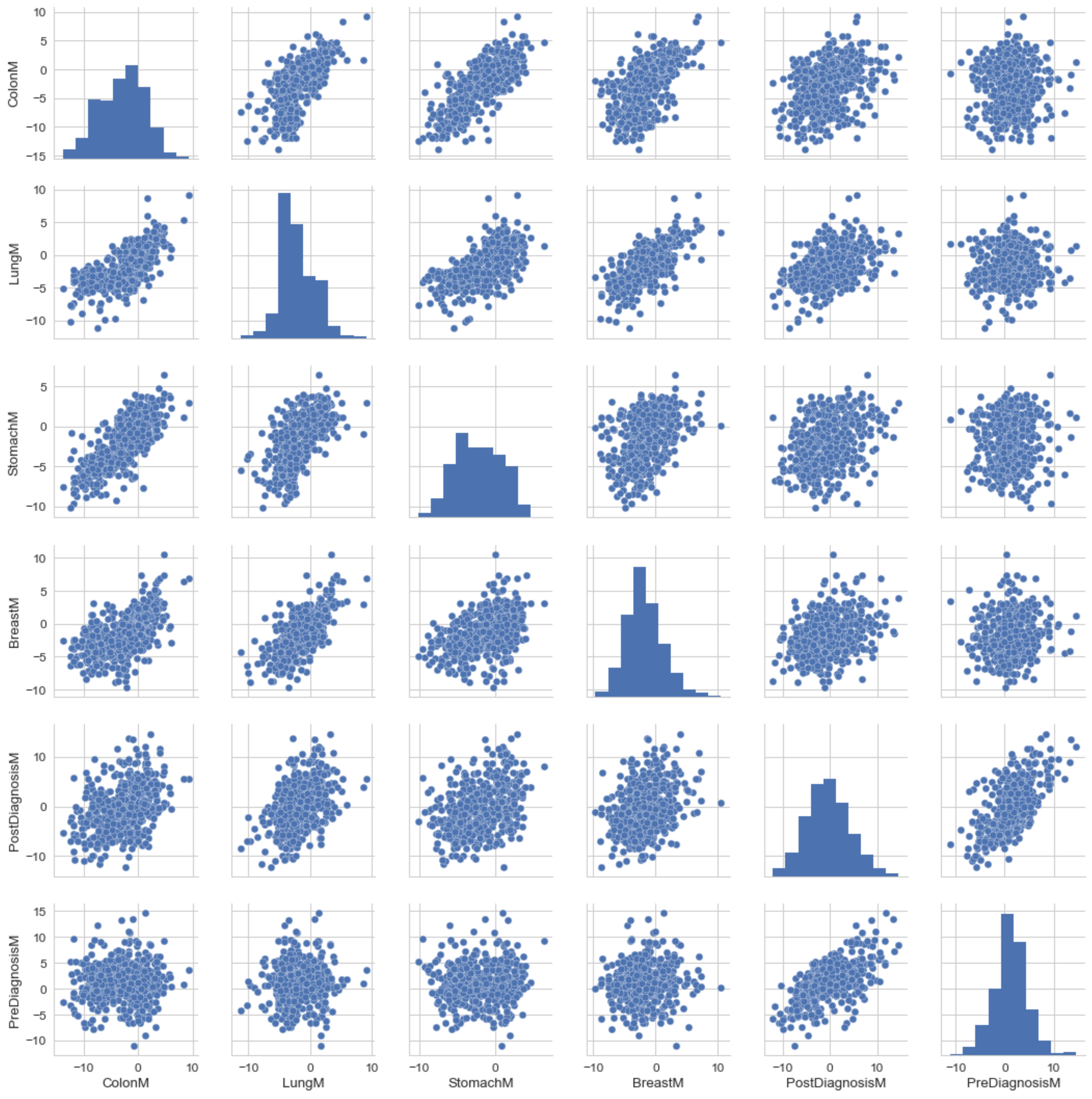

Supplement: Supplementary file 1 — Supplementary Information [file 41467_2020_17316_MOESM1_ESM.pdf]
